# Europe-wide reassessment of *Dictyocoela* (Microsporidia) infecting native and invasive amphipods (Crustacea): molecular versus ultrastructural traits

**DOI:** 10.1038/s41598-018-26879-3

**Published:** 2018-06-12

**Authors:** Karolina Bacela-Spychalska, Piotr Wróblewski, Tomasz Mamos, Michał Grabowski, Thierry Rigaud, Remi Wattier, Tomasz Rewicz, Alicja Konopacka, Mykola Ovcharenko

**Affiliations:** 1 Department of Invertebrate Zoology and Hydrobiology, University of Lodz, 12/16 Banacha Street, 90-237 Lodz, Poland; 2 Witold Stefański Institute of Parasitology of the Polish Academy of Sciences, 51/55 Twarda Street, 00-818 Warsaw, Poland; 3 Laboratoire Biogéosciences, UMR CNRS 6282, Université de Bourgogne Franche Comté, 6 boulevard Gabriel, 21000 Dijon, France; 4 Institute of Biology and Environmental Protection, Pomeranian Academy High School, 22b Arciszewskiego Street, 76-200 Słupsk, Poland

## Abstract

Microsporidia are common parasites infecting animals and protists. They are specifically common pathogens of amphipods (Crustacea, Malacostraca), with *Dictyocoela* spp. being particularly frequent and highly prevalent, exhibiting a range of phenotypic and ecological effects. Until now, seven species of *Dictyocoela* were defined, predominantly based on the genetic distance. However, neither the taxonomic status of this provisionally erected genus (based on eight novel sequences and one micrograph of the spore), nor its internal phylogenetic relationships have been clearly revealed. The formal description of the genus and of most of the putative species are still lacking. Here we aimed to fill this gap and performed both ultrastructural and molecular studies (based on SSU, ITS and partial LSU) using different species delimitation methods. As a consensus of these results and following conservative data interpretation, we propose to distinguish five species infecting gammarid hosts, and to keep the names introduced by the authors of the type sequences: *Dictyocoela duebenum*, *D. muelleri*, *D. berillonum* and *D. roeselum*. We provide full descriptions of these species. Moreover, thanks to our extensive sampling, we extend the known host and geographic range of these Microsporidia.

## Introduction

Microsporidia are obligate intracellular parasites of animals and protists, and are related to the Fungi. They are particularly common pathogens of gammarid amphipods (Crustacea, Malacostraca), where they show a great taxonomic/phylogenetic diversity^1,2^, but also a variety of transmission strategies; as well as phenotypic and ecological effects^3^. *Nosema granulosis* and *Dictyocoela* spp. are usually vertically-transmitted^4,5^, and have the peculiar ability to induce sex ratio distortion in infected populations by feminizing males^6^. Other parasites, such as those belonging to the genus *Cucumispora*, exhibit a direct virulence on their gammarid hosts and are associated with horizontal transmission^7,8^. Microsporidia can therefore impact the key ecological roles of gammarids in ecosystem functioning^9^, either directly or indirectly.

The majority of recent reports on microsporidian infections in gammarids is based solely on molecular methods. In many cases, the phylogenetic position of the parasite, their phenotypic effects, and their transmission modes are not fully understood^2,10,11^. Among these, *Dictyocoela* spp. have been found in numerous gammarid species, frequently at high prevalence^2,5,6,12,13^. However, until now, neither the taxonomic status of Microsporidia belonging to the genus *Dictyocoela*, nor the phylogenetic relationships within this microsporidian clade have been clearly defined. Indeed, Terry *et al*.^5^ provisionally described the genus based on eight novel sequences of the small subunit ribosomal RNA gene (SSU rDNA) that formed a discrete clade that grouped outside of those microsporidians infecting fish. The tubular network that fills the sporophorous vesicle containing the developing parasite spores was described as an ultrastructural feature characteristic to this group. The molecular distinctness of the *Dictyocoela* clade has been suggested by many other authors^7,10,14–19^. However, the formal description of the genus as recommended by Stentiford *et al*.^16^, is lacking. Additionally, some structures such as: the isofilar polar filament; bipartite lamellar polaroplast; three-layered exospore; and tubular inclusions in the episporontal space, also suggest an ultrastructural identity of *Dictyocoela* with *Thelohania* spp., creating a confusion in their taxonomy^1,20–23^. The taxonomic conundrum involving *Thelohania*-like Microsporidia infecting amphipods is not new: discrepancies between morphological and molecular data led to a taxonomical reappraisal for a parasite infecting *Gammarus duebeni celticus*. This parasite was formerly assigned to *Thelohania* (*T. muelleri*), but is now placed in the genus *Pleistophora*^24^. Therefore, the existing confusion between *Thelohania* and *Dictyocoela* genus necessitates further clarification.

In addition, the existence of “species” within the genus *Dictyocoela* is unclear. Originally, Terry *et al*.^5^ distinguished six species under the criterion that SSU rDNA sequences differed by less than 1% of nucleotides would belong to a single species. There are now 139 rDNA sequences placed within the *Dictyocoela* that were isolated from 18 amphipod host species, registered in GenBank (June 2017). In total, names for eight species have been suggested: *Dictyocoela berillonum, D. cavimanum, D. deshayesum, D. duebenum*, *D. gammarellum, D. muelleri, D. roeselum*^5,6,10,12^ and *D. diporeiae*^19^. Transmission electron microscopy (TEM) data are only available for the spore of the microsporidium named *D. muelleri*, isolated from *Gammarus duebeni celticus*^5^, and for *D. diporeiae*, isolated from *Diporeia* sp.^19^; this later species being the only one with sufficient genetic and morphological description. For other “species” the molecular species delimitation was very difficult based on SSU rDNA sequence alone, as there are numerous sequences already available for comparison, which fill the discontinuities in genetic distance. For example, Ironside and Alexander^13^ used “*Dictyocoela* sp.” for their newly-obtained sequences. On the other hand, Grabner *et al*.^2^ defined the “*D*. *duebenum/muelleri* species complex”, clearly separated from the *D. berillonum* clade. Finally, Wilkinson *et al*.^12^, suggested that the species delimitation within the *Dictyocoela* group may be less reliable than first thought. A potential problem is that half of the SSU rDNA sequences available in GenBank are relatively short, and do not exceed 600 bp in length. Since utility of a short fragment of SSU rDNA is questioned in defining species limits by many authors^25^, full sequences of the SSU region, as well as ITS and partial LSU, are required to improve phylogenetic resolution^26^.

In addition to controversies upon the taxonomy and phylogenetic relationships of this parasite group, the known host and geographic range seems to be incomplete. In previous studies, amphipods were sampled mainly in North-Western Europe, with only a few species sampled from Central Europe^2,5,12^. A recent study focused on the highly endemic amphipod community in the Siberian Baikal Lake basin^27^. Among the potential European hosts of *Dictyocoela* the Ponto-Caspian species are of particular interest for further investigations. Several are highly invasive species spreading throughout Central and Western Europe during the last few decades^28^. They are known as vectors of microsporidian diseases in their new invaded ranges^2,18,29^; however, to date, *Dictyocoela* parasites have been evidenced in too few Ponto-Caspian gammarids to get a broader picture of an infection pattern.

To sum-up, it appears surprising that, given the number of *Dictyocoela* records produced up to now and the potential effects they have on the host biology and ecology, the validity of the genus itself and the relationship between the putative species and the host range has not yet been followed up by a solid assessment of morphological and ultrastructural characters in combination with molecular data, as recommended by Stentiford *et al*.^16^. Our study attempts to fill this gap. To do so, we first sampled a broad set of gammarid species, focusing on the Ponto-Caspian species in their native and colonized range. Second, we sequenced SSU, ITS and partial LSU rDNA of detected parasites, to compare them with the sequences available in GenBank, and applied several methods of molecular species delimitation to understand the diversity patterns within this parasite group. Finally, we confronted ultrastructure and molecular traits of four species of *Dictyocoela*, for which inconsistencies in species identity have been highlighted in previous studies^2,6,12^.

## Results

**Figure 1:**
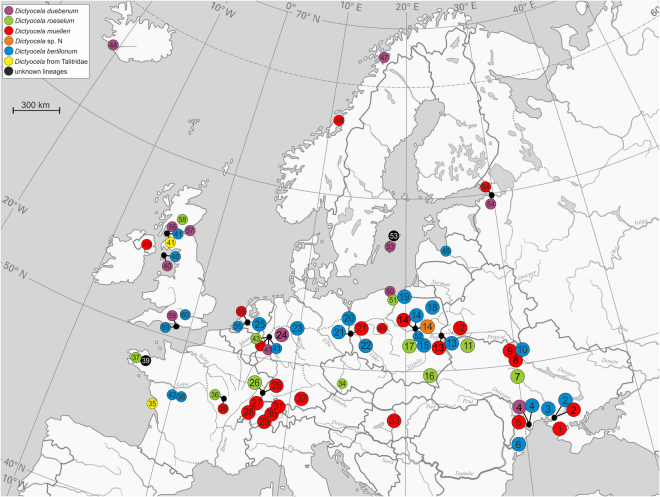
Records of *Dictyocoela* in Europe: big circles – from this study (site numbers 1–33), small circles – GenBank data (site numbers 34–62). Numbers correspond to locations as in Supplementary Tables S1 and S2. Different clades of *Dictyocoela* are indicated by colours as in Figs 1 and 2, Supplementary Fig. S1.

Among the 3862 specimens of gammarid hosts (15 species) collected from 95 sampling sites (Supplementary Table S1), we found *Dictyocoela* spp. in 33 sites (Fig. 1), infecting twelve species (Supplementary Table S1, Supplementary Fig. S1b), including: crustaceans of Ponto-Caspian; Mediterranean; and Atlantic-Boreal origins. The prevalence of *Dictyocoela* infections ranged from 0.6% to 26.1%. In total, we obtained 108 sequences covering SSU rDNA (length 1306–1320 bp) from 33 localities (Fig. 1). A subset of 85 specimens from 29 sites were sequenced to provide a length of 1753–1767 bp (full SSU, ITS and partial LSU rDNA) (Supplementary Table S2).

### Phylogenetic relationships, host range and molecular species delimitation of *Dictyocoela*

Based on the alignment consisting of the 108 sequences produced in this study and the 108 *Dictyocoela* sequences deposited in GenBank covering a 5′ fragment of 524 bp long SSU rDNA, we distinguished 83 haplotypes (Supplementary Table S2). All the *Dictyocoela* haplotypes constituted a large well-supported monophyletic group (Supplementary Fig. S1a). Within this group, we can distinguish seven well-supported clades, yet without resolved mutual phylogenetic relationships. Five of these clades grouped sequences together with the type sequences of already suggested *Dictyocoela* species^5,6^. These clades are intermingled with short singular branches (haplotypes S1–S18), including the type sequence of *D. muelleri* (haplotype S4 = AJ438955). The full description of these clades is provided along with the Supplementary Fig. S1.

**Figure 2:**
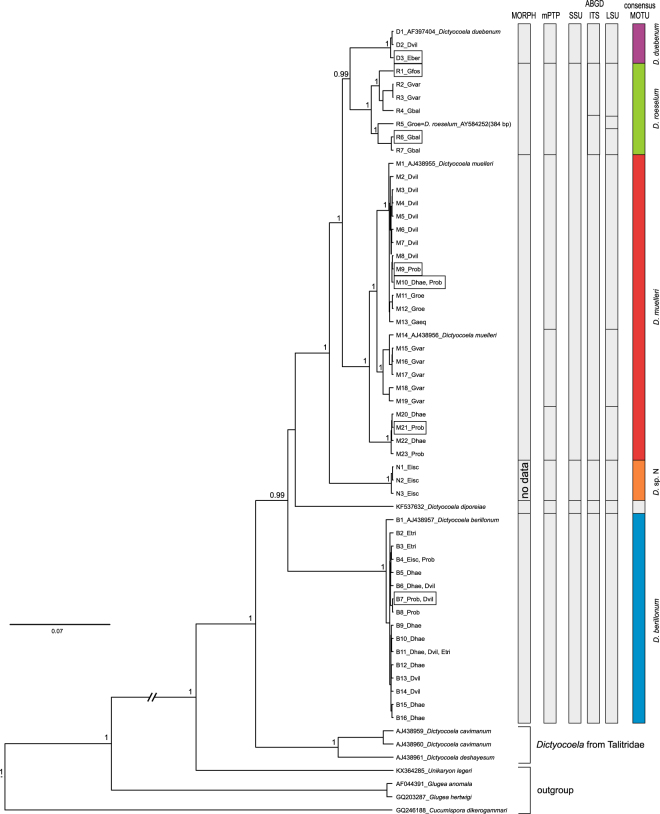
Bayesian phylogenetic reconstruction based on a 1833 bp long alignment of combined SSU, ITS and partial LSU rDNA. Above the branches the Bayesian posterior probabilities for major clades higher than 0.75 are given. Bars annotated on the right represents results of the species delimitation based on morphology and molecular (mPTP and ABGD) methods as well as consensus species delimitation. In frames, the individuals for which the ultrastructural analysis was done are indicated.

Using the combined three markers SSU, ITS and partial LSU (56 *Dictyocoela* haplotypes), the resolution of the *Dictyocoela* phylogeny was improved (Fig. 2) and only the position of *D. diporeiae* remained unresolved. This tree allowed us to define new clades that may be compared to those obtained with the SSU short sequences (Supplementary Fig. S1a). The microsporidian sequences found in talitrid amphipods grouped together to form a sister clade to all the other *Dictyocoela* lineages infecting gammarids and the pontoporeid *Diporeia* sp. Another outer monophyletic group clustered our newly-obtained sequences with *D. berillonum* type sequence^5^, forming the *D. berillonum* clade, which was characterized by very low haplotype diversity (Table 1) and identified almost solely in the Ponto-Caspian hosts. We were able to identify the *D. duebenum* and *D. roeselum* clades (equivalents of D and R clades showed in Supplementary Fig. S1a) gathering sequences similar to the type sequences of these two named *Dictyocoela* (haplotypes D1 and R5, respectively) (Fig. 2). The use of the combined three markers resulted in emergence of the *D. muelleri* clade (Fig. 2), which included sequences with an unresolved phylogenetic position where short SSU fragments were used (Supplementary Fig. S1a: haplotypes S1–S13, clades M and L). Four of these haplotypes clustered in clade B2 in the Bayesian tree of Wilkinson *et al*.^12^. This large *D. muelleri* clade showed the highest haplotypic diversity (Table 1) and included parasites from seven gammarid species: four Ponto-Caspian invaders (*D. haemobaphes, D. villosus, D. bispinosus, P. robustoides*); the Mediterranean/Black Sea *Gammarus aequicauda*; the Balkan *G. roeselii*; the Pontic *G. varsoviensis*; the Western-European *G. duebeni*; and the widespread *G. pulex*. This tree also highlighted a highly supported clade grouping the Microsporidia that infect *Echinogammarus ischnus* from Poland (Fig. 2). We called this clade “*Dictyocoela* sp. N”.

**Table 1:** Genetic characteristics of discriminated *Dictyocoela* species based on SSU (1306–1320 bp), ITS (33–39 bp) and partial LSU (ca 430 bp) rDNA.

species	N	SSU			ITS			LSU		
		H	Hd (SD)	π (SD)	H	Hd (SD)	π (SD)	H	Hd (SD)	π (SD)
*D. duebenum*	4	3	0.833 (0.222)	0.0021 (0.0009)	1	0.0	0.0	2	0.667 (0.314)	0.0016 (0.0007)
*D. roeselum*	9	6	0.917 (0.073)	0.0098 (0.0011)	6	0.917 (0.073)	0.2378 (0.0248)	5	0.889 (0.071)	0.0250 (0.0033)
*D. muelleri*	27	16	0.920 (0.001)	0.0060 (0.0008)	7	0.803 (0.003)	0.0769 (0.0097)	12	0.927 (0.028)	0.0301 (0.0040)
*D*. sp. N	8	1	0.0	0.0	1	0.0	0.0	3	0.464 (0.200)	0.0022 (0.0011)
*D. diporeiae*	1	1	na	na	1	na	na	1	na	na
*D. berillonum*	41	10	0.721 (0.062)	0.0012 (0.0002)	3	0.298 (0.083)	0.04630 (0.0145)	4	0.233 (0.086)	0.0006 (0.0002)

N – number of sequences; H – number of haplotypes, Hd – haplotype diversity; SD – standard deviation; π – nucleotide diversity.

The phylogenies reconstructed separately based on the complete SSU and the LSU markers (Supplementary Fig. S2), both provided the support for the clades described above, but showed also that the combination of the two markers is needed to resolve the deeper part of the phylogeny (Supplementary Fig. S2).

The molecular species delimitation based on the mPTP method defined eight molecular species among the *Dictyocoela* sequences. The distance-based approach (ABGD) divided our dataset into 5 to 10 molecular species, depending on the marker (Fig. 2). These species sets were identical over a wide range of prior maximal distance P values (SSU: 0.0010–0.0129, ITS: 0.0010–0.0599, and LSU: 0.0010–0.0399), a range of relative gap widths (from 0.1 to 5) and two distance metrics (p, K2P). The discrepancies between the mPTP and the ABGD method based on LSU resulted in subdividing the *D. muelleri* and *D. roeselum* clades into 3 molecular species each, while based on ITS *D. roeselum* was divided into two species. In contrary, ABGD based on the SSU marker did not find a barcode gap between *D*. *muelleri* and *D*. *roeselum* clades.

Based on the above results, we selected representatives of the major clades and, if possible, subclades, for further ultrastructural analyses. It was not possible for the new *Dictyocoela* sp. N clade (Fig. 2), because these sequences were obtained from material preserved only in ETOH 96%. Thus, the combination of molecular data, light microscopy data and ultrastructural data were carried out for seven specimens (Fig. 2).

### Clinical signs

**Figure 3:**
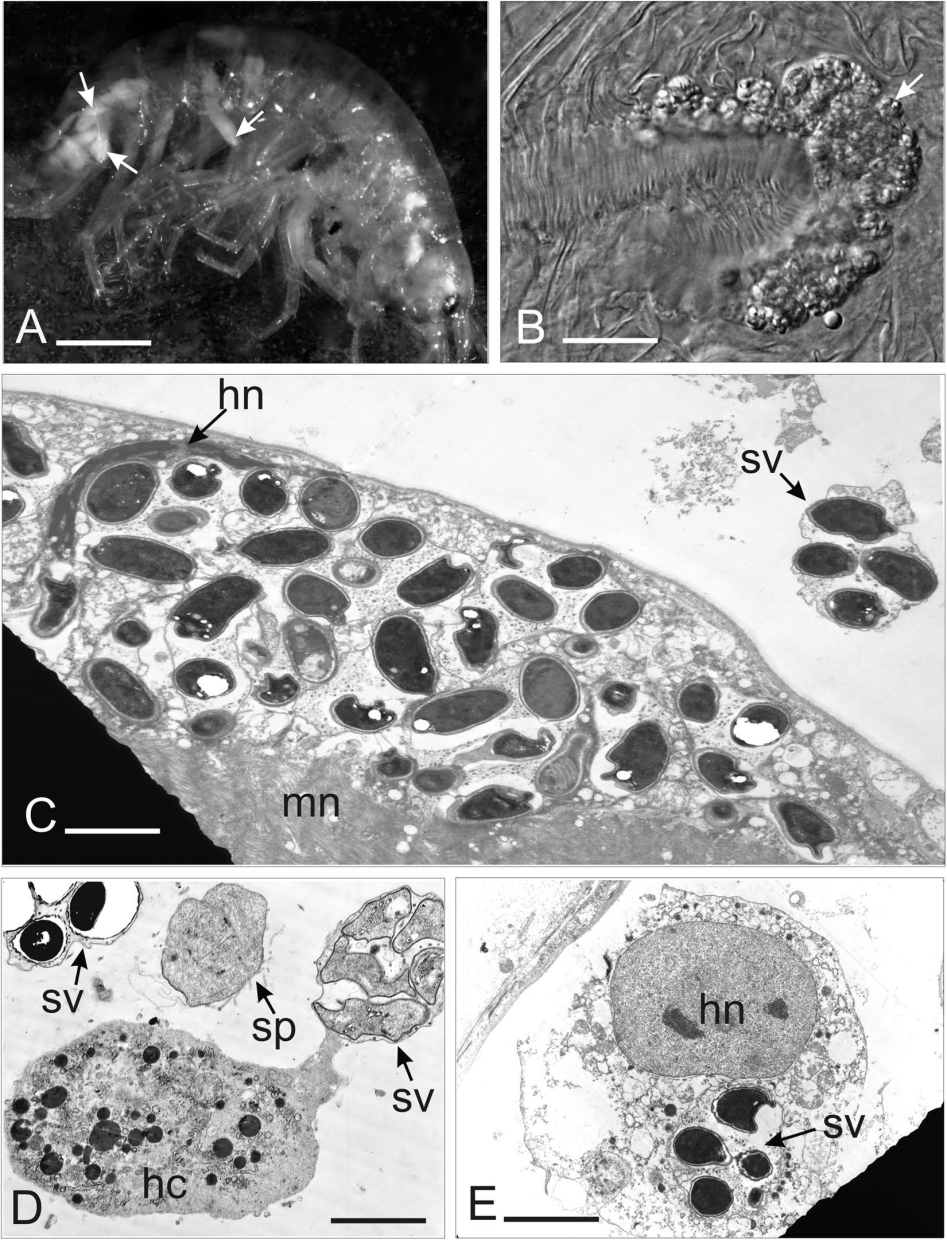
Pathogenicity of *Dictyocoela* spp.: (**A**) *Gammarus varsoviensis* infected with *Dictyocoela muelleri*. Pale-colored musculature indicates microsporidian infection (arrow). (**B**) Infected abdominal muscles (arrow) of the same specimens (Nomarski contrast). (**C**) Ultrathin section of muscle cell of *Pontogammarus robustoides* infected with *D. berillonum*. Sporophorous vesicles are located in the sarcoplasm neighboring myofibrils (mn). Deformation of host nucleus (hn) is observed. Single sporophorous vesicle (sv) is located outside the muscle cell in haemolymph. (**D**) Diplokaryotic sporont (sp) and sporophorous vesicles (sv) of *D. muelleri*, located in hemolymph of *Dikerogammarus haemobaphes*. Sporophorous vesicles (sv) adhering to haemocyte (hc) are visible. (**E**). Sporophorous vesicle of *Dictyocoela berillonum* (arrow) inside the cytoplasm of uninucleate haemocyte of *P. robustoides*. Single nucleus of haemocyte (hn) contains two nucleoli. Scale bars: 2.0 mm (**A**), 0.2 mm (**B**); 3.5 µm (**C**); 2.0 µm (**D**); 4.5 µm (**E**).

Most of the inspected specimens lacked evidence of gross infection/septicemia. Nevertheless, some hosts showed irregular stripes of whitish color, and the dissection revealed presence of numerous microsporidian spores and developmental stages filling the muscles (Fig. 3A,B). Infection was never found in the gut wall. These clinical signs were observed in *G. varsoviensis* and *D. haemobaphes* infected with *D. muelleri* and in *P. robustoides* infected with *D. berillonum*. Sporogonial stages and spores occurred within the cytoplasm of host cells (Fig. 3C,E). Sporogonial stages were found also floating separately in the hemolymph (Fig. 3C) or adhering to hemocytes (Fig. 3D). Sporophorous vesicles were occasionally registered inside the cytoplasm of hemocytes (Fig. 3E). Merogonial stages and sporophorous vesicles containing sporogonial stages and spores were located in direct contact with the host cell cytoplasm (Fig. 3C,E). A host nucleus distortion was recorded in substantially infected muscle cells (Fig. 3C).

### Light Microscope Observations

**Figure 4:**
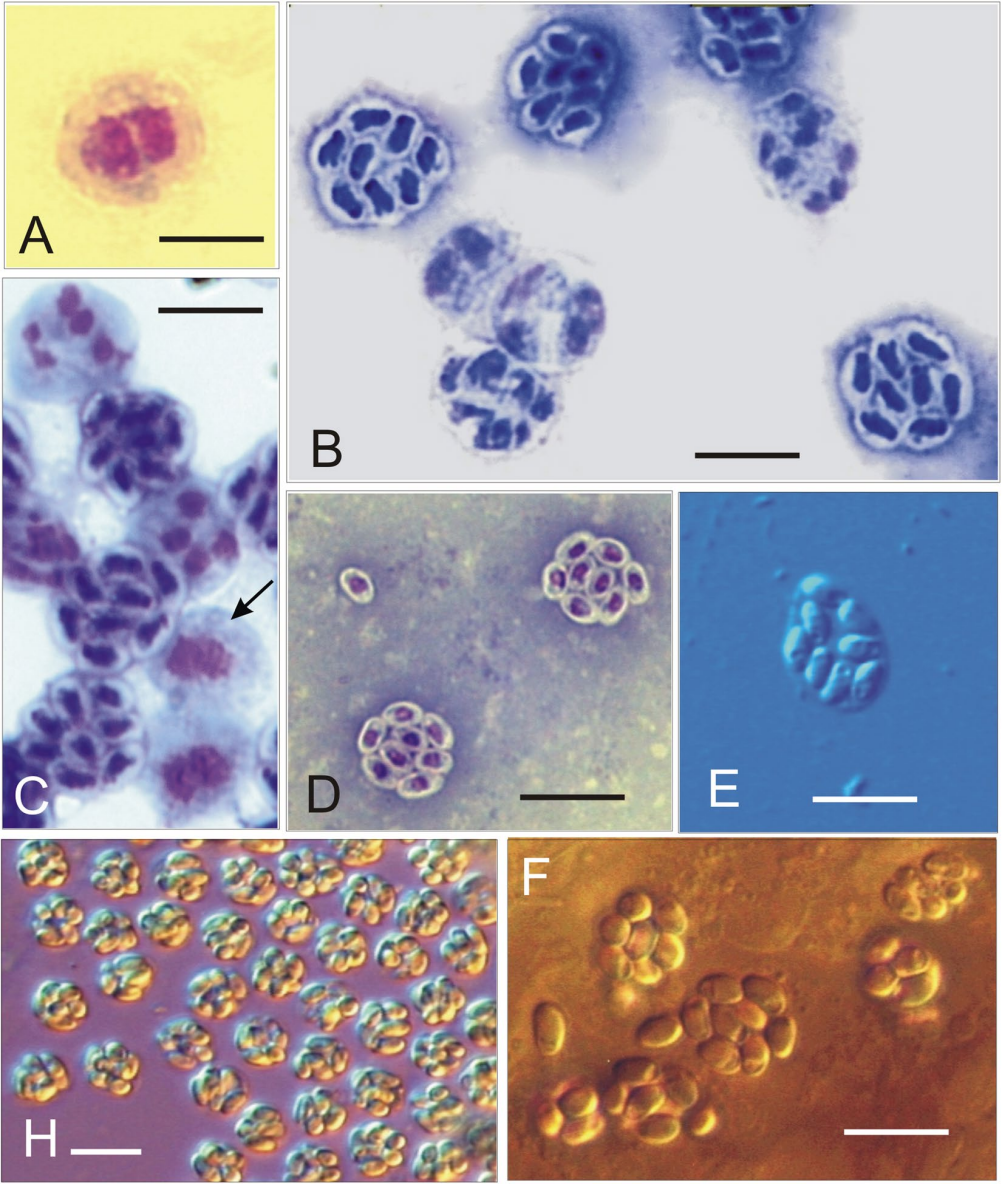
Light micrographs of Giemsa-stained (**A**–**D**) and fresh (**E,H,F**) smears. (**A**) Diplokaryotic merozoite of *Dictyocoela muelleri* from *Pontogammarus robustoides* (**B**) Sporogonial plasmodia and mature octospores of *Dictyocoela roeselum* from *Gammarus balcanicus*. (**C**) Merogonial plasmodium with two diplokaryotic nuclei (arrow), sporonts with four and eight nuclei (beginning of second sporontal division) and mature octospores of *D. berillonum* from *P. robustoides*. (**D)** Mature spores of *D. muelleri* from *D. haemobaphes*. (**E,F**) Live spores of *D. roeselum* from *G. balcanicus* (**E**), *D*. *muelleri* from *P. robustoides* (**H**), and *D. duebenum* from *Echinogammarus berilloni* (**F**). Scale bars: 4.9 µm (**D**); 5.2 µm (**B,C**); 5.8 µm (**E,F**); 6.5 µm (**H**).

All features presented hereafter are valid for all the *Dictyocoela* studied here, otherwise the clade specific feature are specified. The earliest developmental stages of the parasite observed on the Giemsa-stained slides were single diplokaryotic merozoites (Fig. 4A), and ribbon-like merogonial plasmodia (Fig. 4C). Sporogony occurred within sporophorous vesicles (Fig. 4B,C). The sporogonial stages, identified in stained smears, were characterized by sporogonial plasmodia with two to eight nuclei, and sporophorous vesicles containing eight uninucleate sporoblasts. Immature and mature octospores were consistently present in all smears prepared (Fig. 4B–F). Sporophorous vesicles were circular in shape. The live spores were ovoid to plum shaped, lightly irregular with equally rounded ends about 3 × 2 μm in size (Fig. 4E,F). Giemsa stained spores were ovoid to pyriform (Fig. 4B–D). A small posterior vacuole was visible close to the posterior part of the stained spores (Fig. 4D). Spores differed in sizes among the Microsporidia defined as separate species via molecular tools (Table 2).

**Table 2:** Morphological characteristics of 4 candidate and one described by Winters and Faisal (2014)*. *Dictyocoela* species. Haplo – haplotype name as shown on Fig. 2/Supplementary Fig. 1a; Site – site number as in Fig. 1 and Supplementary Table S2; SE – standard error; PC – number of polar filament coils.

Sequence abbreviations	Host	Haplo	Site	Live spore sizes ± SE	Giemsa stained spore sizes	PC	Sporophorous vesicle inclusions
***D. duebenum***							
DEB2	*Echinogammarus. berilloni*	D3/SD1	26	No data	2.73 ± 0.29 × 1.47 ± 0.18 µm (n = 120)	9–10	Rare tubules 80–100 nm in diameter and loosely packed electron dense filaments 40–50 nm in diameter
***D. roeselum***							
GbOv2	*Gammarus balcanicus*	R6/SR6	17	3.78 ± 0.31 × 2.15 ± 0.24 µm (n = 23)	2.68 ± 0.37 × 1.66 ± 0.33 µm (n = 576)	7–8	Rare microtubules about 70 nm in diameter, rare electron dense filaments and granules
GfOv	*G. fossarum*	R1/SR7	18	3.11 ± 0.45 × 1.85 ± 0.25 µm (n = 141)	2.77 ± 0.26 × 1.75 ± 0.16 µm (n = 70)	7–8	Rare microtubules 70–80 nm in diameter, rare granules
***D. muelleri***							
ProbM33	*Pontogammarus robustoides*	S3/M9	6	3.18 ± 0.29 × 2.03 ± 0.19 µm (n = 125)	2.47 ± 0.30 × 1.52 ± 0.22 µm (n = 117)	11–12	Microtubules and numerous granular aggregates about 40–60 nm in diameter
PrOv1	*P. robustoides*	M21/SMB2	15	No data	2.35 ± 0.28 × 1.39 ± 0.16 µm (n = 46)	11–12	Microtubules and numerous granular aggregates 40–50 nm in diameter
DhaeM22	*Dikerogammarus haemobaphes*	M10/S4	2	3.31 ± 0.37 × 1.96 ± 0.30 µm (n = 120)	2.94 ± 0.27 × 1.63 ± 0.19 µm (n = 120)	12–13	Microtubules and numerous granular aggregates about 40 nm in diameter
***D. berillonum***							
ProbM32	*P. robustoides*	B7/SB1	11	3.00 ± 0.29 × 1.69 ± 0.17 µm (n = 123)	2.34 ± 0.26 × 1.42 ± 0.16 µm (n = 98)	5–6	Not numerous tubules 70–100 nm in diameter and granular aggregates
D. diporeiae*							
	*Diporeia* sp.	Ddip		No data	1.99 ± 0.09 × 1.19 ± 0.05 µm	8	No data

### Ultrastructure

**Figure 5:**
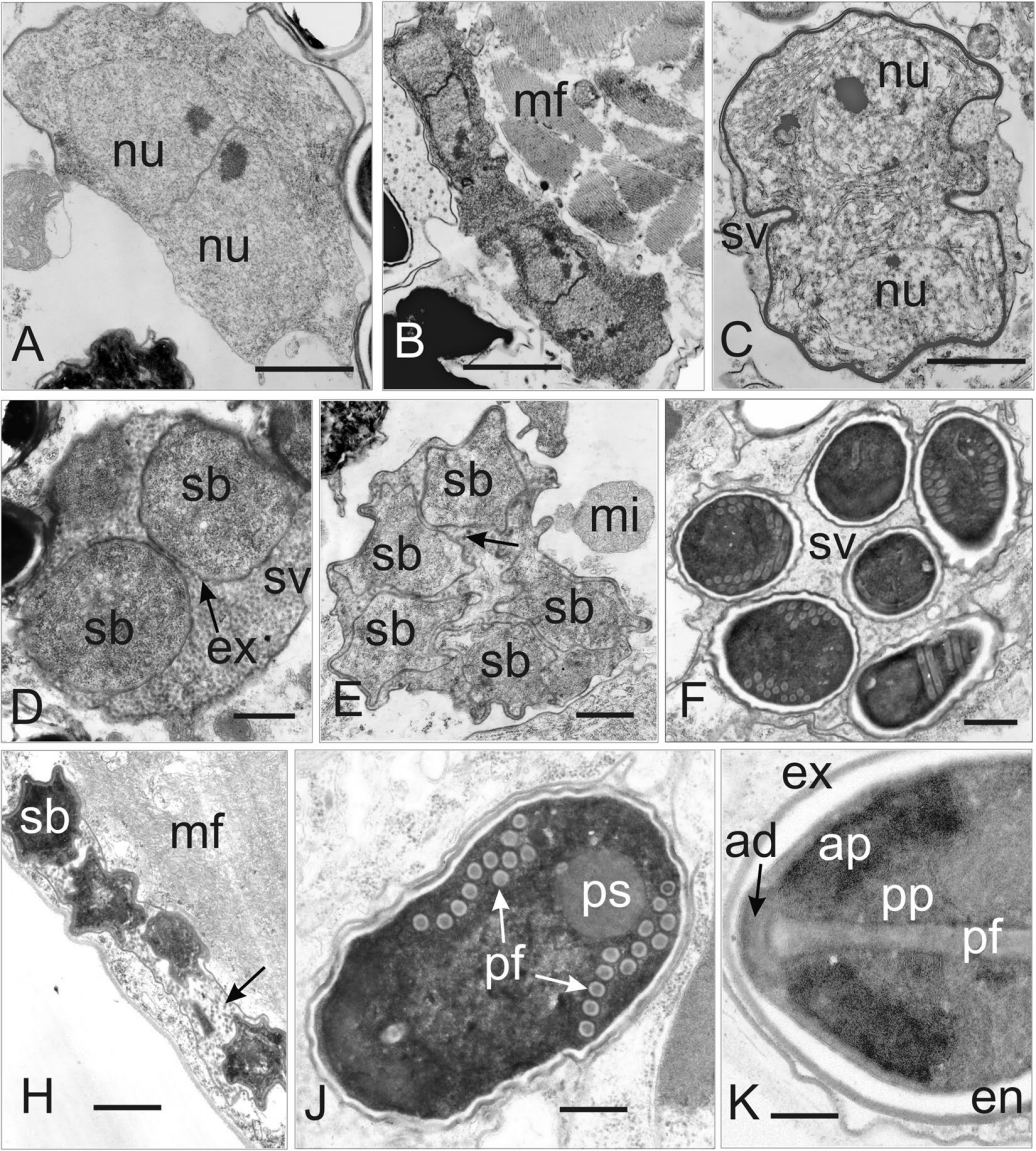
Ultrastructure of the developmental stages of *Dictyocoela muelleri* from *Pontogammarus robustoides* (**A–E,H,J**) and *Dikerogammarus haemobaphes* (**F,K**). (**A**) Diplokaryotic merozoite. The cytoplasm is homogenously granular. A few cisternae of rough cytoplasmic reticulum are visible. (**B**) Merogonial plasmodium neighboring myofibrils (mf). Dividing presporont with two diplokarya. (**C**) Beginning of meiotical division of the sporont market by detaching the two parts of the diplokaryon from each other. The separation of the sporophorous vesicle membrane (sv) is visible. (**D**) Part of the sporophorous vesicle (SV) with uninucleate sporoblasts (sb). The sporoblast mother cells surrounded by granular secretory material. Episporontal cover (arrow) mark the start of future exospore (ex) development. (**E**) Ultrathin section of sporophorous vesicle containing young uninucleate sporoblasts (sb). Granular material, forming tubular episporontal inclusions is arrowed. (**F**) Sporophorous vesicle (sv) with mature spores and tubular inclusions. (**H**) Lateral section of the sporophorous vesicle containing late sporoblasts (sb). The space between the sporoblasts is filled with granular and tubular inclusions (arrow). (**J**) Immature spore with 11–12 polar filament coils (pf) and posterosome (ps). Thin layer of electron-lucent endospore and complete exospore are clearly visible. (**K**) Longitudinal section of the anterior part of the mature spore showing the endospore (en), exospore (ex), manubrial part of polar filament (pf), fine lamellar anterior polaroplast, wide lamellar posterior polaroplast (pp), and anchoring disc (ad). Scale bars: 200 nm (**K**); 500 nm (**A**,**C**,**D**); 600 nm (**J**); 700 nm (**B**); 800 nm (**E**,**F**); 1.5 nm (**H**).

**Figure 6:**
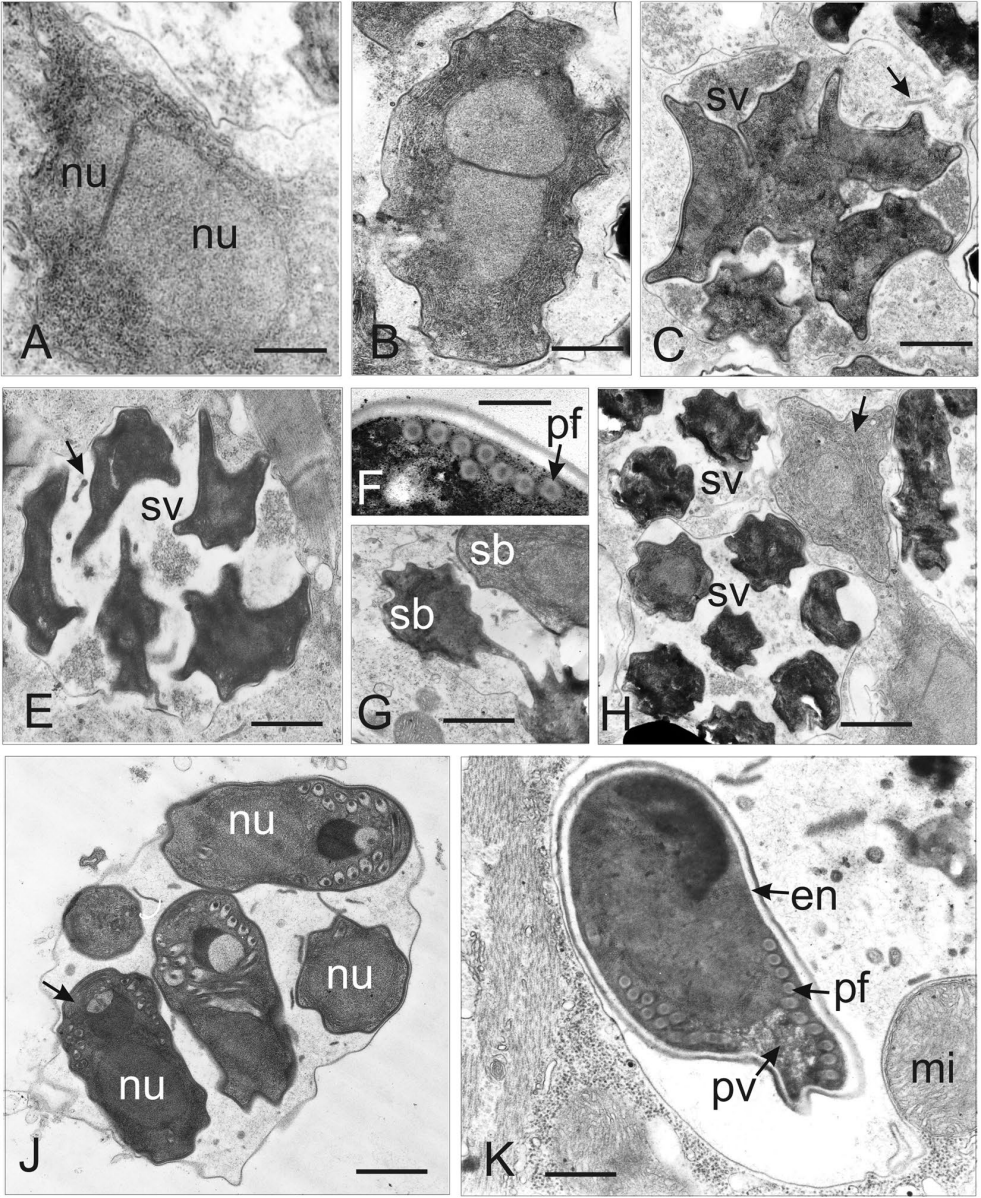
Ultrastructure of developmental stages of *D*. *roeselum* from *Gammarus balcanicus* (**A**–**F**) and *D. duebenum* from *Echinogammarus berilloni* (G–K). (**A**) Oblique section of merozoite showing two attached nuclei (nu) and homogenously granular cytoplasm. (**B**) Diplokaryotic presporont with cytoplasm containing numerous cysternae of the endoplasmic reticulum. The cell is coated with electron dense material. (**C**) Sporophorous vesicle (sv) with divided sporogonal plasmodium. Episporontal space contains not numerous tubules (arrow) and granular substance. (**E**) Sporophorous vesicle (sv) with separated late sporoblasts, rare microtubules (arrow) and aggregated granules. (**F**) Part of mature spores showing eight coils of isofilar polar filament (pf). (**G**) Part of sporophorous vesicle with rare inclusions. Divided and separated sporoblasts (sb) are visible. (**H**) Early merozoite, late merogonal stages (presporont) and sporophorous vesicles (sv) containing late sporoblasts are presented. The presporont is arrowed. (**J**) Sporophorous vesicle with immature spores (arrow) and rare loosely packed inclusions are presented. The dense Golgi-derived substance fills the core of the polar filament and forms a large globe shaped inclusion. (**K**) Section through the posterior part of mature uninucleate spore. Polar filament (pf), posterior vacuole (pv) and spore wall ultrastructure with exospore (e) and endospore (en) are visible. Sporophorous vesicle contains rare tubules 70–100 nm in diameter and eletron dense filaments 40–50 nm in thickness. Scale bars: 100 nm (**B**); 300 nm (**A,F**); 800 nm (**K**); 1.2 µm (**C,E,G,J**); 1.5 µm (**H**).

No differences between the seven analyzed specimens have been observed during merogony and early sporogony. The merogonial stages displayed as rounded or elongated merozoites approximately 1.5 × 3.2 μm in size (Figs 5A and 6A,B,H). They possessed two nuclei (nu) in diplokaryotic arrangement. The cytoplasm of the early merozoites was homogenously granular. A few cisternae of rough cytoplasmic reticulum were occasionally registered in cytoplasm (Figs 5A and 6H). Two granular nuclei arranged as a diplokaryon measured 1.2–1.5 μm in diameter and occupied more than a half of the cell volume. Merozoites were multiplicated by binary division of diplokaryotic cells. Pairs of diplokaryotic nuclei (nu) have separated prior to division of the cytoplasm (Fig. 5B). The first observations of partial thickening of the cell surface were reported during merogony. Finally, the presporont surface was covered with electron-dense granular material about 30 nm thick (Figs 5B and 6B,H).

**Figure 7:**
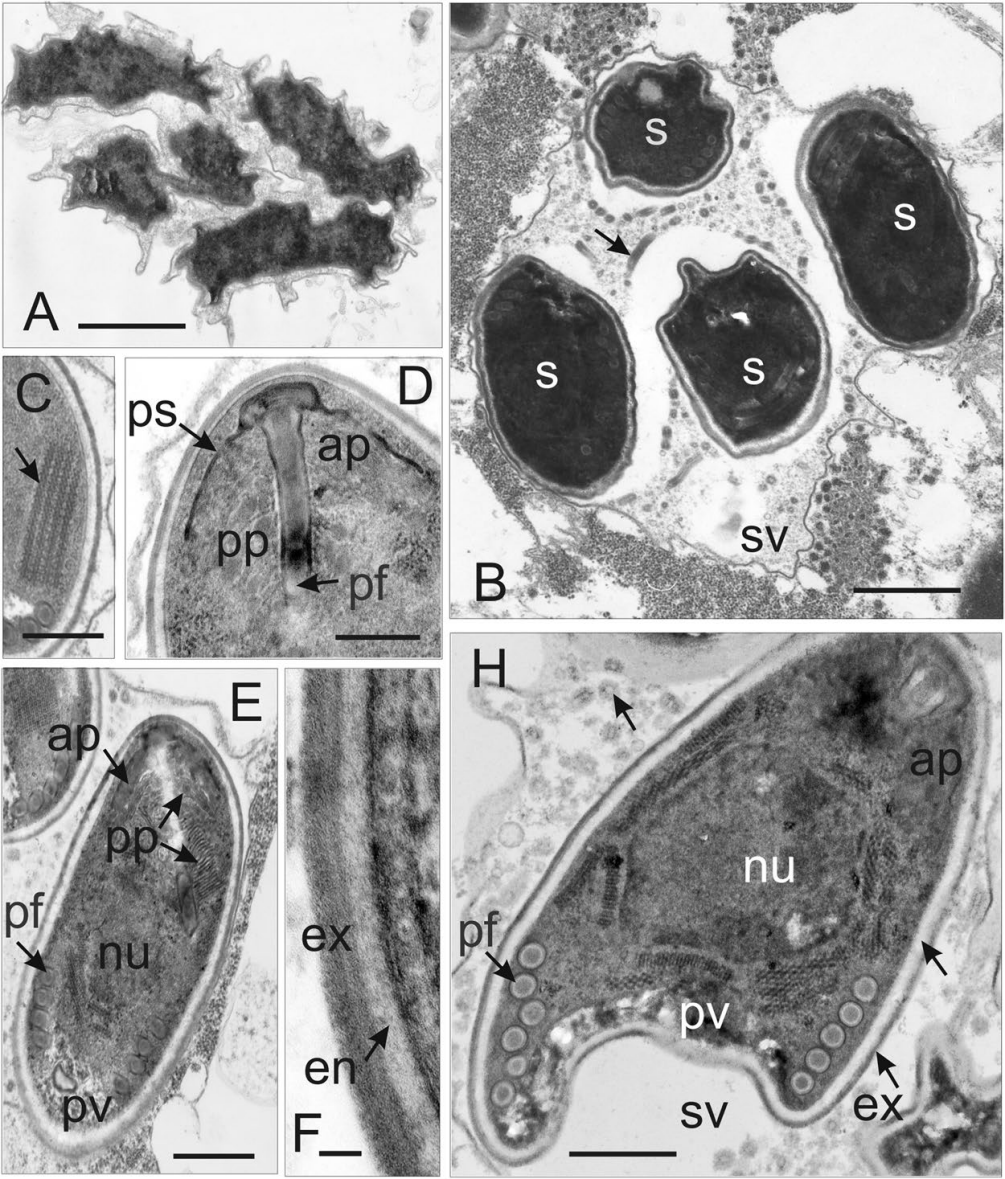
Late sporogonial stages of *Dictyocoela berillonum* from *Pontogammarus robustiodes*. (**A**,**B**) Section of the sporophorous vesicles (sv) containing sporoblasts (**A**) and spores (**B**). The sv space is filled with thin granular material and not numerous septate tubules (arrow). (**C**) Part of immature spore with polyribosomes (arrow). (**D**) Anterior part of immature spore with anchoring apparatus, polar sac (ps), manubrial part of polar filament (pf), thin lamellar anterior polaroplast (ap), and cysterns of posterior polaroplast (pp). The spore wall is narrowed at the apical pole. (**E**) Anterior part of mature spore with lamellar posterior polaroplast (pp) and posterior vacuole (pv). Tubular inclusions, structure of the spore wall and coiled part of polar filament (pf) are visible. (**F**) Ultrastructure of the spore wall. The plasmalemma, endospore (en) and two layers of exospore (ex) are presented. (**H**) Oblique section of mature spore. Anterior polaroplast (ap), single nucleus (nu), 5–6 coils of the polar filament (pf), posterior vacuole (pv), and inclusions (arrow) inside sporophorous vacuole (sv) are displayed. Scale bars: 40 nm (**F**); 300 nm (**D**); 400 nm (**H**); 500 nm (**C**,**E**); 1.5 µm (**A**,**B**).

The sporophorous vesicle (SV) was separated around the sporont coat. Detaching the two parts of the diplokaryon from each other, and marked beginning of meiotical division of the sporont (Fig. 5C). The resulting sporogonal plasmodium contains four separated nuclei. Each of the nuclei gives rise to the formation of four uninucleate sporoblasts. The sporoblast mother cells were surrounded by thin granular secretory material of the episporontal space. The episporontal cover marks the start of future exospore (ex) development (Fig. 5C). Each sporoblast underwent binary division. Finally, eight uninucleate sporoblasts were produced within persistent SV (Fig. 6H). Plasmotomy of the divided sporogonal plasmodium was not permanently synchronous (Fig. 6C). Sometimes, the SV contained uninucleate and divided sporoblasts of different maturity (Fig. 6B). In most cases, synchronous separation of the plasmodium was observed and the SV contained sporoblasts of the same degree of development (Figs 5E,H and 7A,E,H). Sporophorous vesicles contained granular material, which developed to form episporontal inclusions (Figs 5E,H and 6C**–**J). Episporontal inclusions of Microsporidia differed in size and shape among different molecular parasite species (Table 2). Mature spores also differed among the analyzed species. Spores were uninucleate (Fig. 7H), thin walled, and filled by ribosomes arranged in a crystalline pattern as polyribosomes (Fig. 7C). The spore wall consisted of an electron-lucid endospore (47–60 nm thick) and a two-layered exospore (45–47 nm thick) (Fig. 7F). The isofilar polar filament (pf) crossed the polaroplast (Figs 5K and 7E) and was regularly coiled into 5 to 13 turns, depending on species, tightly arranged in one row (Figs 5J, 6E,K and 7H) (Table 2). Spores possessed a small posterior vacuole (PV) filled with a granular substance (Figs 6K and 7E,H). A rounded posterosome (PS) was sometimes seen in immature spores (Fig. 5J).

As a consensus of molecular and morphological results, and following conservative data interpretation, we propose to distinguish five *Dictyocoela* spp. that infect gammarids, which should hold the following species names after Terry *et al*.^5^, including: *D. duebenum*; *D. muelleri*; *D. berillonum*; and Haine *et al*.^6^ for *D. roeselum*. The last candidate species (clade *Dictyocoela* sp. N) must remain unnamed as there are no morphological data available to allow a full species description. We also support *D. diporeiae* as a separate species. This division is also confirmed by the within and between clades p-distance, counted separately on the three markers used, where on each occasion the within p-distance is at least two times lower than between the closest relative clade (Supplementary Table 3). The BOLD analysis of diagnostic characters revealed from 3 up to 21 diagnostic characters for the 5 molecular species of *Dictyocoela* distinguished in this study (Supplementary Table 4).

## Discussion

The combination of morphological and genetic data allowed us to provide the full description of the genus *Dictyocoela*, after the original suggestion by Terry *et al*.^5^, as well as to elucidate the phylogenetic relationships between the different proposed species within this genus. Our study, by showing wide host range of some *Dictyocoela* lineages and presence of the infection in muscle tissue, provided further information on the possible horizontal transmission mode in *Dictyocoela* lineages, which is in addition with the vertical transmission which has been previously presented as obligatory^5^.

### *Dictyocoela* confirmed as a genus and resolution of the conundrum with *Thelohania* spp. infecting amphipods

As noted in the introduction, some gammarid-infecting Microsporidia share the same morphological characteristics (uninucleate spores, bipartite polaroplast and posterior vacuole filled with granules, sporophorous vesicle filled with microtubules), and were either assigned to the genus *Dictycoela*^5^ or to *Thelohania*^1,21–23^. None of the *Thelohania* recorded in gammarid crustaceans were examined with molecular methods, contrary to the *Dictyocoela* that form a monophyletic clade^2,5,6,10,12–15,29^. Terry *et al*.^5^ included a *Thelohania* sequence in their phylogenetic tree (infecting red fire ants), and this parasite clustered in the clade that was the most distant from the *Dictyocoela* group. Vossbrinck and Debrunner-Vossbrinck^14^ presented similar results. Adding to the conundrum, many other studies showed that the *Thelohania* genus is paraphyletic and needs major revision^30,31^.

Our molecular data confirms that all ribosomal (and ITS) sequences belong to a single microsporidian clade, the one originally noticed by Terry *et al*.^5^. None of the Microsporidia sequences obtained from gammarids in this study were found to be related to *Thelohania* parasites, as has been observed by previous studies^2,5,32^. The genetic K2P distance between our *Dictyocoela* SSU rDNA sequences and the closest available *Thelohania* sequence (*T. butleri*, GenBank accession number: DQ417114) was 18.6%, while in the case of the other *Thelohania* spp. infecting crustaceans the distance exceeded 44% (*T. parastaci*, AF294780; *T. contejeani*, AF49259436). All morphological descriptions made on individuals for which the sequences are available, were in accordance with both previous descriptions of *Dictyocoela* and *Thelohania* from gammarids (presence of tubular inclusions within sporophorous vesicles; production of octospores with double layered exospore; bipartite lamellar polaroplast). Some variations in the number of polar filament turns, microtubule size and quantity of granular aggregates were nevertheless observed. However, sporogony of all inspected *Dictyocoela* species underwent binary division of each of the four early sporoblasts, contrary to the series of three binary divisions observed in *Thelohania* parasites that infect other crustacean species, such as: *T. butleri*; *T. giardi*; *T. contejeani*^30,33,34^. We therefore propose that most, if not all, microsporidian infections previously described in gammarids as *Thelohania* spp. should now be considered as *Dictyocoela* spp.

### Full support for species subdivisions within the *Dictyocoela*

Terry *et al*.^5^ distinguished six species within their proposed *Dictyocoela* genus based on a 1% dissimilarity in their SSU sequences. However, also using the SSU marker, Wilkinson *et al*.^12^ and Grabner *et al*.^2^ noted no clear evidence for the ‘species’ separation of *D. muelleri* from *D. duebenum*; however, only short sequences were used by the authors.

The utility of SSU rDNA in resolving the phylogeny of the Microsporidia, particularly at the species level, has been acknowledged in many studies^17^. However, our study demonstrates the usefulness of multi-locus analysis, which has already been suggested as an informative tool for species delimitation^35^, where relatively short SSU sequences did not provide a clear phylogenetic signal^2,12^. In the case of *D. muelleri* sequences, the phylogenetic relationship was unresolved by using only the short fragment of the SSU. By combining three markers (but also based on full SSU), these sequences were grouped into a well-supported *D. muelleri* clade, which was confirmed by morphological data. Even if the relationships among the main lineages of *Dictyocoela* infecting gammarids could not have been resolved, the major clades were constant whatever the marker used: full SSU or partial LSU (Supplementary Fig. S2).

Based on: (i) additional sequences; (ii) longer sequences in the parasite’s ribosomal region; (iii) results of several molecular species delimitation methods; and (iv) the use of molecular and morphological results in combination, we distinguished five major clades that can be treated as candidate species within the *Dictyocoela*, in addition to the already described *D. diporeiae*^19^. The four candidate species that are fully described here are: *D. duebenum*; *D. muelleri*; *D. berillonum*; and *D. roeselum*. Detailed arguments for these descriptions are provided below. We will not discuss the systematic position of *Dictyocoela* spp. infecting talitrid hosts, because we did not detect any parasite in our study that clustered within this clade, and because no morphological data are currently available.

A comparison of the single electron micrograph that illustrated the first provisional description of *Dictyocoela muelleri*^5^ with our ultrastructural analysis of the Microsporidia grouped within *D. muelleri* clade confirms their identity. Thus, based on molecular and morphological data we are able to assess that all these parasite observations belong to *D. muelleri*. The results of mPTP and ABGD on LSU suggested that three species may be recognized within the *D. muelleri* clade. However, this was not supported by the morphological analysis, where all the qualitative characteristics of the parasites belonging to these sub-clades were identified as the same, and their quantitative characteristics were seen to overlap (Table 2). This could be an indication of an ongoing diversification process, which may be a result of the relatively wide host range observed for this parasite (Supplementary Fig. S2 and Table S2).

In contrast, the *D. berillonum* clade is genetically homogenous (Table 1). The sequence (haplotype B7, Fig. 2) obtained from *P. robustoides* sampled in the Kievski Reservoir (Ukraine), which is studied here ultrastructurally, showed 99.5% similarity to the type sequence of *D. berillonum* (AJ438957) deposited by Terry *et al*.^5^. Thus, we propose to use the ultrastructural data we obtained in the present study as a valid description of *D. berillonum*. Similarly, the morphological description made in this study for the *Dictyocoela* obtained from *Echinogammarus berilloni*, which was collected from the Aa River (Germany) (haplotype D3, Fig. 2) showed 99.7% identity to the type sequence of *Dictyocoela duebenum* (AF397404)^5^, and provided reason to authorize *D. duebenum* as a valid species and allow full species description.

The SSU haplotypes R1 and R6 (Fig. 2), from *Gammarus fossarum* and *G. balcanicus*, respectively, were morphologically analyzed and found to be very similar (99.1% and 99.2% of identity, respectively) to the haplotype R5, which was identical to the sequence AY584252 (384 bp) found in *G. roeselii* and provisionally called *Dictyocoela roeselum* by Haine *et al*.^6^. We use the morphological features of these parasites as a valid description of *D. roeselum* herein.

Our results appear to be in contrast with those of Wilkinson *et al*.^12^ and Grabner *et al*.^2^. These authors highlighted a lack of clear evidence for the separation of *D. muelleri* from *D. duebenum*. In our analysis we obtained a good support for all clades, the species delimitation methods confirmed these clades as candidate species (Fig. 2), and morphological features gave support for such discrimination. Differences in the length of the analyzed sequences may explain these differences. In the study made by Wilkinson *et al*.^12^ the length of the alignment based on SSU was at best 520 bp, which might explain the unresolved phylogeny. In addition to this potential problem with short sequences, a high number of sequences recently obtained from amphipod-infecting Microsporidia from the Baikal Lake, an ancient and highly isolated watershed, could bias the analysis (see Supplementary Fig. S1a). The taxonomic summaries for these species are provided in Supplementary Discussion 1.

### Geographic and host ranges: suggested patterns for *Dictyocoela* infections

Our results extend the known geographic and host ranges of *Dictyocela* spp. The geographic distribution and host range of *D. muelleri*, *D. duebenum* and *D. roeselum* does not show any pattern. The presence of *D. muelleri* in the Ponto-Caspian gammarids sampled in their native range (Danube and Dnieper deltas), shows that these Microsporidia are not restricted to *Gammarus* sp. native to Western Europe. These records disagree with the hypothesis of a recent host shift from native gammarids to invaders in the colonized area, as suggested by Wattier *et al*.^29^ and Wilkinson *et al*.^12^. Nevertheless, a spillover of the *Dictyocoela muelleri* or *D. duebenum* between native local gammarids and the invasive non-native species from the Ponto-Caspian cannot be excluded. We found muscle infections in many amphipods found to be positive for *Dictyocoela*. This supports the suggestions of Wilkinson *et al*.^12^ and Grabner *et al*.^2^ that the horizontal transmission of these parasites is possible, in addition to the vertical transmission observed in some *Dictyocoela* lineages^5,6,36^, thus making a host shift event possible.

The Baikal Lake basin is particularly interesting. The *D. duebenum* parasites infecting *Gmelinoides fasciatus* and other Baikalian amphipods (SD3-13, Supplementary Fig. S1a) are rarely clustering together with *Gammarus lacustris* sampled in the lake vicinity (SD14, 16–18, 26, 29), with some exceptions (sequence SD16 from *Gm. fasciatus*; sequences SD 23, 24 from *Eulimnogammarus cyaneus*). This result conforms with observations by Ironside and Wilkinson^27^, who suggest that an exchange of Microsporidia between Baikalian and non-Baikalian hosts have occurred in the past, but are relatively rare.

The only lineage that may show some degree of host limitation is *D. berillonum*. In our study, this parasite was found exclusively in Ponto-Caspian hosts (Supplementary Fig. S1b). Wilkinson *et al*.^12^ and Grabner *et al*.^2^ also found this parasite to infect Ponto-Caspian hosts outside their native range. Recently, Etxabe *et al*.^36^ recorded a high prevalence of *D. berillonum* infecting the Ponto-Caspian invader *D. haemobaphes*, a recent colonizer of UK freshwaters. Interestingly, they recorded the same (and most common) haplotype as recorded by Grabner *et al*.^2^, and in our study (SB1, Fig. 2). In the light of these results from central and western Europe, the distribution of the *D. berillonum* lineage appears to be found exclusively in Ponto-Caspian host species and could be a result of successful co-invasion of the parasites alongside the host, as suggested by Grabner *et al*.^2^. However, to contradict this hypothesis, the first records of this parasite species came from several non-Ponto-Caspian amphipod species: they were frequent in *Echinogammarus marinus* in UK and in *E. berilloni* in France, and more sporadic in *Gammarus duebeni duebeni, G. tigrinus*, and *Melita palmata*^5^. All the SSU sequences of these parasites were a single haplotype in all 5 host species. It should be noted that *Echinogammarus* hosts share a phylogenetic proximity with Ponto-Caspian gammarids^37^ and therefore, an ancestral infection of these host groups cannot be dismissed. In addition, a more recent spillover on ecologically closely-related hosts (*Melita* or *Gammarus*) is not impossible to consider.

The biogeography of *Dictyocoela* spp. appears to be complex and poorly understood. Globally, our findings highlight the lack of knowledge about the dispersal ability and the specificity of Microsporidia that infect gammarids. It cannot be excluded that spores disperse freely within the water current and may be transmitted not only through direct contact between hosts but via environmental methods. The spores of fish-infecting Microsporidia can be infectious even after one year of being outside their host’s body in the aquatic environment^38^. The presence of vectors or reservoir species may also explain the successful spread of some particular lineages between populations of different crustacean hosts^39^. Even if some authors suggest high host specificity for many microsporidia^40^, there are Microsporidia infecting many, sometimes unrelated, species^11^. Vossbrinck *et al*.^41^ reported *Octosporea muscaedomesticae* to infect seven species of calyptratae flies, and Solter *et al*.^42^ reported *Cystosporoenes* sp. infecting 19 lepidopteran species. Also, *Pleistophora muelleri* infecting *Gammarus duebeni* is suspected to use fish as intermediate or reservoir host^43^. The newest methods of species identification based on environmental DNA metabarcoding could help to test the dispersal abilities of Microsporidia in the aquatic ecosystem, and identify possible vectors or reservoir species^44^.

Another possibility for explaining the puzzling distribution of *Dictyocoela* in their amphipod hosts is to consider that these Microsporidia diversified before the host species did. They could have different (co-)evolutionary histories within the different diversifying host clades, but the precise evolution could be masked by frequent host shifts (at both the ecological and evolutionary time scales). An interesting study has been made in *Gammarus duebeni* across its natural range^10^, supporting the hypothesis that there could have been co-radiations of different Microsporidia in this host species, even if no clear relationship was established within the host phylogeny. Contrastingly, links between microsporidian infections and ecological features were also found, the parasites of animals living in freshwater belonged to different clades than those of hosts living in brackish water. We believe that only detailed studies including complete histories of host and parasite (co-)diversifications can help to understand the *Dictyocoela* infection pattern in gammarids.

## Material and Methods

### Sampling and light microscopy

Our study is based on material from 3,862 individual amphipods, collected during several field expeditions between 2000 and 2012 from 95 sites located in 15 countries, which altogether cover a large area of Western, Central and Eastern Europe (Supplementary Table S1). The collection consisted of two sample sets: 1) samples from which material was brought fresh to the laboratory in order to preserve correctly for light and electron microscopy analysis, as well as the subsequent molecular analysis and (1,015 individuals from 33 sites); 2) samples preserved in ethanol on site (2,847 individuals from 62 sites). Such a strategy was used as a compromise due to logistic constraints that prevent keeping and housing live material collected in remote places for a prolonged time. The animals were collected using standard kick sampling procedure. Gammarids were sorted out in the field, transported to the laboratory either fresh or directly stored in 96% ethanol and identified to species level. All the analysed material is kept in the collections of the Department of Invertebrate Zoology and Hydrobiology, University of Lodz, and in the Laboratory of Biogeosciences, University Bourgogne Franche-Comte, Dijon.

Individuals of freshly collected gammarids were dissected, and pieces of tissues were fixed in 96% ethanol for molecular analyses. Animals with a visible white coloration (possible signs of microsporidian infection) were used to prepare the light-microscope slides. First, the infected tissues were smeared on microscope slides. Then the smears were air dried, fixed in methanol and stained with Giemsa-stain solution. Both living and methanol-fixed Giemsa-stained spores were observed and measured under an Olympus BX50F4 microscope using the attached camera and PC computer with the Analysis Pro 2.11 software.

Material directly preserved in 96% ethanol was used to reveal the geographic distribution of *Dictyocoela* spp. All these individuals were screened with molecular methods for the presence of Microsporidia.

### Molecular analysis

#### DNA extraction, amplification and sequencing of microsporidian SSU, ITS and partial LSU rDNA

Microsporidian DNA was co-extracted with host DNA. Around 4 mm^3^ of the host tissue including muscles and gonads was homogenized in a 1.5 ml tube containing 200 ml of Queen’s lysis buffer^45^ using a plastic pestle and incubated overnight at 55 °C with 5 ml of proteinase K (20 mg/ml). DNA was then extracted based on a standard phenol-chloroform method^46^. Air-dried DNA pellets were re-suspended in 100 ml of TE buffer, pH 8.00. The microsporidian SSU rDNA, ITS and partial LSU rDNA operon was amplified as two overlapping amplicons using the Microsporidia specific primer pairs: V1f (forward) (5′-CACCAGGTTGATTCTGCCTGAC-3′^45^) with MC3r (reverse) (5′-GATAACGACGGGCGGTGTGTACAA-3′^7^ and HG4f (5′-GCGGCTTAATTTGACTCAAC-3′^47^) with 580r (reverse) (5′*-*GGTCCGTGTTTCAAGACGG-3′^48^). A negative control containing no DNA was included in each set of PCR reactions. The PCR conditions were as follows: an initial denaturing step at 94 °C for 2 min was followed by 35 cycles of 94 °C for 20 s, 55 °C for 45 s and 65 °C for 1 min 50 s, final extension at 65 °C for 5 min. The PCR product was visualized on the 2% agarose gel in order to identify the infected individuals. All the PCR products were purified with exonuclease I (Burlington, Canada) and FastAP alkaline phosphatase (Fermentas) treatment and sequenced directly with the BigDye technology by Macrogen Inc., the Netherlands. All PCR products were sequenced in both directions using the primers mentioned above. The microsporidian sequences were edited using BioEdit software^49^. Combining sequencing data from the two PCR products allowed us to analyze the full SSU rDNA (ca 1300 bp), full ITS (ca 40 bp) and partial LSU rDNA (ca 430 bp).

To complete our molecular dataset with *Dictyocoela* sequences available in GenBank, we performed a BLAST search^50^ using all our sequences as queries. All the resulting sequences without any undetermined nucleotides that showed at least 90% similarity to the query were included in the dataset for further analyses.

#### Phylogeny reconstruction

The sequences were aligned in the MAFFT 7 software^51^ using the E-INS-i algorithm (alignments available in Supplementary material). Prior to any phylogenetic analyses presented below, we identified the number of haplotypes in DnaSp^52^, and determined the model of sequence evolution for each dataset with bModelTest^53^ in BEAST 2.4.6^54^.

First, an alignment of partial SSU rDNA (524 bp) combined our 108 newly produced sequences with the 108 *Dictyocoela* sequences available in GenBank (Supplementary Table S2). As an outgroup, we used four GenBank-obtained sequences of aquatic Microsporidia placed, similarly to *Dictyocoela*, in the Class Marinosporidia (AF044391, GQ203287, GQ246188, KX364285)^7,14,26^. This first dataset was used to (1) show relationship of our sequences with as much as possible *Dictyocoela* sequences available in GenBank, taking into account that the majority of deposited sequences are relatively short; (2) evaluate the ability of short SSU rDNA sequences to produce resolved phylogeny at a shallow taxonomic level. We performed Bayesian and Maximum Likelihood (ML) analyses. The preferred model of nucleotide substitution was General Time Reversible (GTR) with gamma-distributed rate heterogeneity (G) and a significant proportion of invariable sites (I). The ML analyses were run in MEGA7^55^ with 1,000 bootstrap replicates implemented as a test for robustness of the tree topology. The Bayesian phylogeny was reconstructed using MrBayes software^56^. Four heated chains, two million iterations long, and sampled every 500 iterations. The runs obtained satisfactory Effective Sampling Sizes (ESS > 200) and the potential scale reduction factor values equaled 1 for all parameters. The 50% majority-rule consensus tree was constructed after removal of 10% burn-in phase.

Second, we combined our sequences of SSU rDNA, ITS and partial LSU rDNA (48 haplotypes) and aligned them with the type sequences of Terry *et al*.^5^ as well as with the sequence of *D. diporeiae* (KF537632). This analysis aimed at producing the phylogenetic tree with the highest-possible topology resolution. The Bayesian phylogeny reconstruction was set up based on partitions, one for each marker, and substitution models were set as follows: SSU: General Time-Reversible (GTR) model of evolution with gamma-distributed rate heterogeneity (G) and a proportion of invariable sites (I) GTR + G + I, ITS: Jukes-Cantor (JC69) + G, LSU: Tamura-Nei (TN93) + G + I. Additionally, the Bayesian phylogeny was reconstructed using the same evolution models for SSU rDNA and LSU rDNA in order to compare the tree topologies and verify if the long SSU fragment could offer better resolution than shorter ones or if the partial LSU alone provides enough phylogenetic information. The Bayesian phylogeny reconstructions were performed in BEAST 2.4.6^54^ using 50 million iterations of Markov Chain Monte Carlo (MCMC) and sampled every 5,000 MCMC iterations and Birth-Death model as a prior tree. For each data set, four independent runs were performed. Runs were analyzed using Tracer 1.6^57^ and attained the Effective Sampling Sizes (ESS) > 200. To generate the final cladogram, the trees from separate runs were combined using LogCombiner and then annotated with TreeAnnotator, in BEAST 2.4.6 package^54^.

#### Molecular species delimitation

To explore the number of molecular operational taxonomic units (MOTUs) that may represent potential species within the *Dictyocoela* clade, we applied the tree-based phylogenetic approach using the Multi-rate Poisson Tree Processor (mPTP) proposed by Kapli *et al*.^58^. The mPTP method accounts for different levels of intraspecific genetic diversity deriving from differences in either the evolutionary history or number of lineages sampled within each species. The program uses the Akaike Information Criterion to select the best-fitting tree to input the number of species. As the input tree, we used the Bayesian maximum clade credibility phylogeny based on the combined three markers. The delimitation was carried out using mPTP webserver: http://mptp.h-its.org.

In addition, we performed the distance-based barcode-gap approach using the Automatic Barcode Gap Discovery (ABGD) software^59^. The ABGD method is based upon pairwise distance measures and is restricted to a single *locus*, thus we performed this analysis separately for the SSU, ITS and LSU. ABGD was used with a set of values of relative gap width (X ranging from 0.1 to 5) and two distance metrics (p, K2P), with the other parameters at default values.

#### Genetic diversity of Dictyocoela lineages

The DNA sequence polymorphism parameters, i.e. haplotype diversity (Hd) and nucleotide diversity (π), were calculated by using DnaSP 5.10.01^52^. The mean molecular distances between and within clades identified in our dataset were calculated with MEGA7^55^.

#### Detection of molecular diagnostic features

We applied discrete nucleotide substitutions as molecular descriptive characters used in formal species descriptions, following Grabowski *et al*.^60^. Diagnostic character analysis was first performed in BOLD (www.boldsystems.org)^61^ based on p-distance and performed for the combined markers obtained in our study (SSU, ITS and partial LSU, ca 1780 bp). The analysis identifies consensus nucleotide sequences from each MOTU and compares them to those sequences from other groups to eventually characterize each consensus nucleotide.

### Transmission electron microscopy

The ultrastructure was analyzed for seven specimens representing main clades identified on the phylogenetic trees (see Results). Samples of infected tissues were fixed in a 2.5% (v/v) glutaraldehyde in a 0.2 M sodium cacodylate buffer (pH 7.4) for 3–7 days. After washing and post-fixation in 2.0% (w/v) osmium tetroxide in the same buffer for 1 h at 4 °C, the pieces were dehydrated in a graded series of ethanol and acetone and embedded in Epon-Araldite solution using a standard procedure^62^. Blocks of embedded tissues were sectioned with an LKB III ultra-microtome. Semithin sections (0.7 µm thick) were stained with methylene blue. Ultrathin sections were then mounted on copper grids. After contrasting with uranyl acetate (10 min) and lead citrate (15 min), the sections were examined using a JEOL 1010 electron microscope at 75–80 kV.

### Data availability

GenBank accessions of 43 haplotypes identified based on SSU, ITS and partial LSU rDNA are MG773213-MG773260.

## Electronic supplementary material

Supplementary File

Supplementary Dataset
