# Supplementary material for: Europe-wide reassessment of Dictyocoela (Microsporidia) infecting native and invasive amphipods (Crustacea): molecular versus ultrastructural traits
Source: Sci Rep. 2018 Jun 12;8:8945. doi: 10.1038/s41598-018-26879-3 (PMC5997659; doi:10.1038/s41598-018-26879-3)
Supplement: Supplementary file 1 — Supplementary File [file 41598_2018_26879_MOESM1_ESM.pdf]

Europe-wide reassessment of *Dictyocoela* (Microsporidia) infecting native and invasive amphipods (Crustacea): molecular versus ultrastructural traits

Karolina Bacela-Spychalska, Piotr Wróblewski, Tomasz Mamos, Michał Grabowski, Thierry Rigaud, Remi Wattier, Tomasz Rewicz, Alicja Konopacka, Mykola Ovcharenko

Supplementary Discussion 1. Taxonomic summary of the *Dictyocoela* genus and four *Dictyocoela* spp. described in this study.

Phylum: Microsporidia Balbiani, 1882
Family: *incertae sedis*
Genus: *Dictyocoela* Terry *et al*., 2004**.**
Merogony: In direct contact with host cell cytoplasm. Binucleate (diplokaryotic) merozoites were multiplicated by binary division.
Sporogony: A plasmodium with four nuclei gives rise to the formation of four uninucleate sporonts. Each sporont undergoes binary division. Finally eight uninucleate sporoblasts are produced within persistent sporophorous vesicles. Sporophorous vesicle contains granular material, forming microtubular inclusions. The tubular network fills the sporophorous vesicle containing parasite spores.
Spores uninucleate, thin-walled with three-layered exospore, bipartite polaroplast and posterior vacuole filled with vacuoles and granules. Anterior part of polaroplast is composed with thinnest, closely packed lamellae, posterior part contains straight, less regular lamellae or flattened tubules. The polar filament isofilar. Small subunit ribosomal DNA as well as analysis based on combined SSU, ITS, partial LSU clearly show distinctiveness and monophyly of *Dictyocoela* clade thus supports the establishment of the new genus.

Dictyocoela muelleri Terry *et al*., 2004 (type species)
Synonim: *Thelohania muelleri* (Friedrich et al. 1995).
Specific diagnosis: The live spores ovoid to plum shaped, slightly different in sizes from different host infected: 2.3 – 4.3 × 1.3 – 2.7 μm. Giemsa stained spores ovoid to pyriform: 1.9 – 3.5 × 1.0 – 2.1 μm in size. Polar filament turned into 11 – 13 coils.
The exospore 35 – 40 nm wide, endospore remains 90 – 120 nm in thickness.
Sporophorous vesicle contains granular secretory material, forming nest of microtubules 40 – 60 nm in diameter.

Molecular diagnostic features: G in position 123; C – 134; T – 1668 (alignment in S2).
Hosts: *Pontogammarus robustoides* (Sars, 1894); *Dikerogammarus haemobaphes* (Eichwald, 1841); *D. villosus* (Sowinsky, 1894); *D. bispinosus* Martynov, 1925; *Gammarus duebeni duebeni* Lilljeborg, 1852; *G. duebeni celticus* Lilljeborg, 1852; *G. varsoviensis* Jazdzewski, 1975; *G. aeqiucauda* (Martynov, 1931); *G. pulex* (L.); *G. roeselii* Gervais, 1835.
Locality: European fresh and brackish waters.
Site of infection: Muscle tissue, ovary, eggs, haemolymph.
Transmission: Vertical[5,6], probably horizontal[2,12].
Type material: Type slides unknown, type sequence: AJ438955, AJ438956[5]. Paratype material (slides and TEM blocks) from *P. robustoides* (ProbM33) and *D. haemobaphes* (DhaeM22) are deposited in slides collections of Witold Stefański Institute of Parasitology of the Polish Academy of Sciences, 51/55 Twarda Street, 00-818 Warsaw, Poland.

Dictyocoela berillonum Terry et al., 2004
Specific diagnosis: The live spores ovoid to plum shaped, 2.3 – 3.7 × 1.3 – 2.0 μm . Giemsa stained spores ovoid to pyriform: 2.4 – 3.5 × 1.3 – 2.1 μm in size. Polar filament turned into 5 – 6 coils. The exospore 35 – 40 nm wide, endospore remains 65 – 75 nm in thickness. Immature sporophorous vesicle contains granular secretory material, forming nest of not numerous tubules 70 – 110 nm in diameter.
Molecular diagnostic features: G in position 40; A – 112; C – 150; T – 336; A – 701; T – 959; A – 1005; T – 1087; A – 1128; T – 1523; A – 1578; A – 1579; T – 1580; T – 1588; C – 1621; G – 1632; A – 1672; A – 1675; G – 1677; G – 1678; T – 1779 (alignment in S2).
Hosts: *Pontogammarus robustoides* (Sars, 1894); *Dikerogammarus haemobaphes* (Eichwald, 1841); *D. villosus* (Sowinsky, 1894); *Gammarus duebeni duebeni* Lilljeborg, 1852; *G. pulex* (L.); *G. tigrinus* Sexton, 1939; *Echinogammarus berilloni* (Catta, 1878); *E. ischnus* (Stebbing, 1899); *E. marinus* (Leach, 1815); *E. trichiatus* (Martynov, 1932), *Melita palmata* (Martynov, 1932).
Locality: European fresh, marine and brackish waters.
Site of infection: Muscle tissue, ovary, eggs, haemolymph, haemocytes.
Type material: Type slides unknown, type sequence AJ438957[5].
Paratype material (slides and TEM blocks) from *P. robustoides* (ProbM32), is deposited in slides collections of Witold Stefański Institute of Parasitology of the Polish Academy of Sciences, 51/55 Twarda Street, 00-818 Warsaw, Poland.

Dictyocoella duebenum Terry et al., 2004

Synonim: *Thelohania muelleri* (Friedrich et al., 1995).

Specific diagnosis: Giemsa stained spores ovoid to pyriform: 1.9 – 3.4 × 1.1 – 2.0 µm in size. Polar filament turned into 9 – 10 coils.

The exospore 20 – 30 nm wide, endospore remains 60 – 70 nm in thickness. Immature sporophorous vesicle contains granular secretory material in the episporontal space, forming nest of loosely packed tubules 70 – 80 nm in diameter.

Molecular diagnostic features: A in position 19; T – 20; C – 22; C – 1328; G – 1513; C – 1582; A – 1594; C – 1615; A – 1649; G – 1762 (alignment in S2).

Hosts: *Echinogammarus berilloni* (Catta, 1878); *E. marinus* (Leach, 1815); *Dikerogammarus villosus* (Sowinsky, 1894); *Gammarus duebeni duebeni* Lilljeborg, 1852; *G. pulex* (L.); *G. tigrinus* Sexton, 1939; *G. roeselii* Gervais, 1835; *G. fossarum* Koch, 1836; *G. lacustris* Sars, 1864; *G. locusta* (L.), *G. pseudolimneus* Bousfield, 1958; *Acanthogammarus lappaceus* Tachteew, 2001; *Ac. victorii* (Dybowsky, 1874); *Dorogostaiskia parasitica* (Dybowsky, 1874); *Eulimnogammarus cyaneus* (Dybowsky, 1874); *Eu. verrucosus* (Gerstfeldt, 1858); *Eu. marituji* Bazikalova, 1945; *Gmelinoides fasciatus* (Stebbing, 1899); *Pallasea cancellous* Dorogostaisky, 1917.

Locality: European fresh, marine and brackish waters, Baikal

Site of infection: Muscle tissue, ovary, eggs, haemolymph, haemocytes.

Type material: Type slides unknown, type sequence AF397404[5]. Paratype material (slides and TEM bloks) from *E. berilloni* (DEB2) are deposited in the slide collection of Witold Stefański Institute of Parasitology of the Polish Academy of Sciences, 51/55 Twarda Street, 00-818 Warsaw, Poland.

Dictyocoela roeselum sp. nov

Synonymes: *Thelohania muelleri* (Larsson 1983), *Dictyocoela* sp. (e.g. GU196258) *D. duebenum* (KR871359), *Dictyocoela* sp. (*roeselum*) (AY584252)

Specific diagnosis: The live spores ovoid to plum shaped, 3.3 – 4.3 × 1.7 – 2.5 µm. Giemsa stained spores ovoid to pyriform: 1.9 – 3.6 × 1.6 – 2.4 µm in size. Polar filament turned into 7 – 8 coils. The exospore 20 – 30 nm wide, endospore remains 60 – 70 nm in thickness. Sporophorous vesicle contains granular secretory material, forming nest of rare sometimes septate microtubules about 70 nm in diameter and filamentous structures 50 – 60 nm in thickness.

Molecular diagnostic features: C in position 346; G – 446; G – 1326; G – 1670 (alignment in S2).

Hosts: *Gammarus balcanicus* Schäferna, 1922; *Gammarus duebeni celticus* Lilljeborg, 1852; *G. fossarum* Koch, 1836; *G. lacustris* Sars, 1864; *G. roeselii* Gervais, 1835; *G. pulex* (L.); *G. varsoviensis* Jażdżewski, 1975.

Locality: European fresh, marine and brackish waters.

Site of infection: Muscle tissue, ovary, eggs, haemolymph, haemocytes.

Type material: Type material (slides and TEM blocks) from *G. balcanicus* (GbOv2) are deposited in the slide collection of Witold Stefański Institute of Parasitology of the Polish Academy of Sciences, 51/55 Twarda Street, 00-818 Warsaw, Poland and sequence of SSU, ITS and partial LSU deposited in GenBank (MG773220).

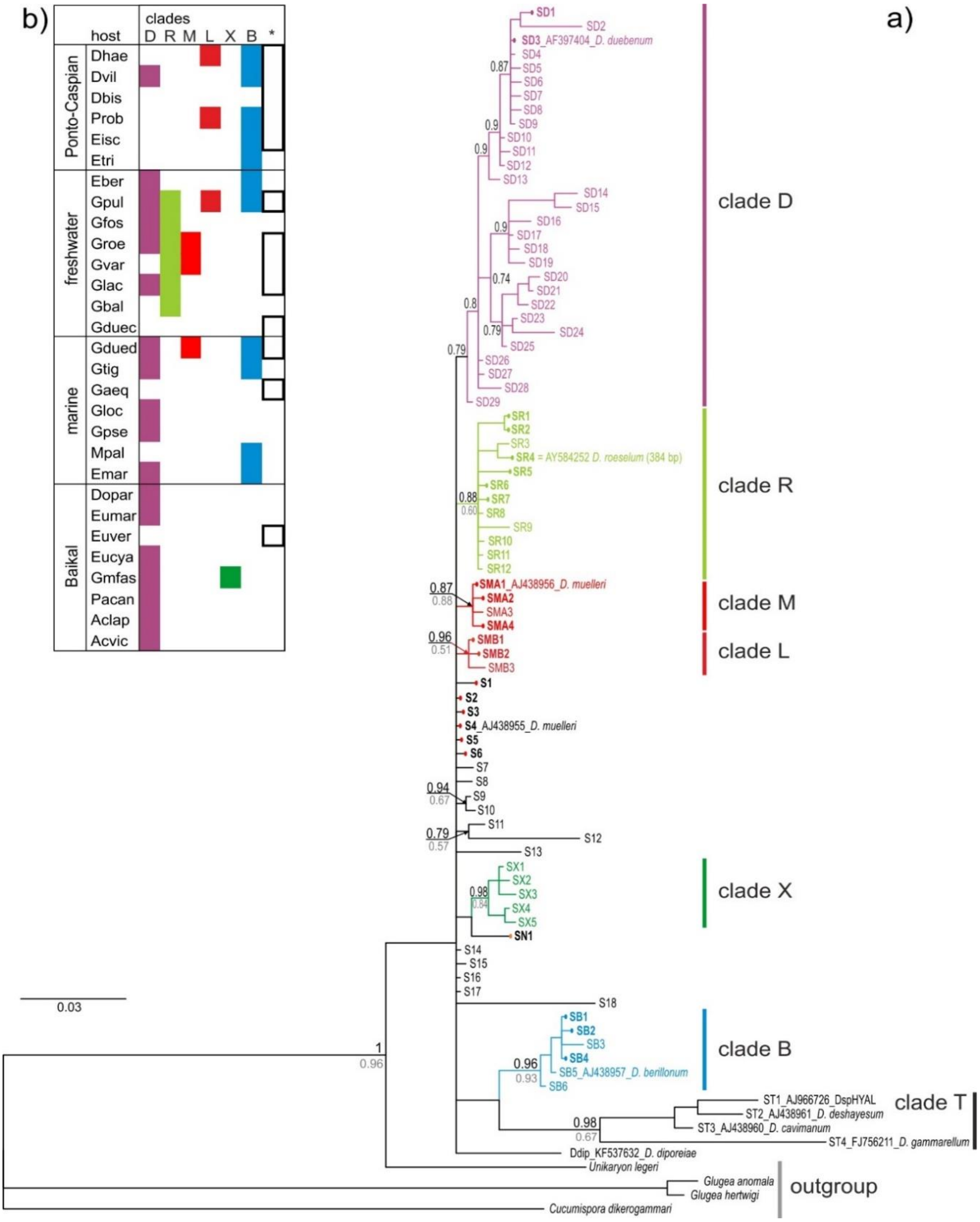

Supplementary Figure S1. a) Bayesian phylogenetic tree of *Dictyocoela* SSU rDNA haplotypes identified in the study (in bold) and published in GenBank, built on the 524 bp long alignment. The Bayesian posterior probabilities are given for the major nodes, when values are higher than 0.75. Below the branches the bootstrap values of ML analysis are given when higher than 0.5 (in grey). By colour dots at the end of the branches the clustering of particular sequence in the tree from Fig. 2 is indicated. **b)** Host range of *Dictyocoela* clades infecting gammaroidean hosts (* – lineage unknown). Host species names as follows: Dhae –

Dikerogammarus haemobaphes; Dvil – *D. villosus*; Dbis – *D. bispinosus*; Prob – *Pontogammarus robustoides*; Eisc– *Echinogammarus ischnus*; Etri – *E. trichiatus*; Eber – *E. berilloni*; Gpul – *Gammarus pulex*; Gfos – *G. fossarum*; Groe – *G. roeselii*; Gvar – *G. varsoviensis*; Glac – *Gammarus lacustris*; Gbal – *G. balcanicus*; Gduec – *Gammaraus duebeni celticus*; Gdued – *Gammarus duebeni duebeni*; Gtig – *G. tigrinus*; Gaeq – *G. aequicauda*; Gloc – *G. locusta*; Gpse – *G. pseudolimnaeus*; Mpal – *Melita palmata*; Emar – *E. marinus*; Dopar – *Dorogostaiskia parasitica*; Eumar - *Eulimnogammarus marituji*; Euver – *Eu. verrucosus*; Eucya – *Eu. cyaneus*; Gmfas – *Gmelinoides fasciatus*; Pacan – *Pallasea cancellous*; Aclap – *Acanthogammarus lappaceus*; Acvic – *A. victorii*.

All 83 *Dictyocoela* haplotypes (524 bp long fragment of SSU rDNA) constituted a large well-supported monophyletic group with seven well-supported clades, yet without resolved mutual phylogenetic relationships. These clades are intermingled with short singular branches (haplotypes S1-S18), including the one of the type sequence of *D. muelleri* (haplotype S4 = AJ438955).

Clade D grouped 29 haplotypes, including the type sequence of *D. duebenum* (haplotype SD3 = AF397404[5]). These parasites were found in 18 host species and had a wide geographic distribution in Europe, including the Ponto-Caspian region (Fig. 1), and the Baikal Lake (Supplementary Table S2). Clade R included 12 haplotypes infecting six species of *Gammarus*, with three reported for the first time as hosts of *Dictyocoela*. Haplotype SR4, found in *Gammarus roeselii*, was identical to the 384 bp long sequence (AY584252) deposited in GenBank by Haine et al.[6] provisionally named *Dictyocoela roeselum.*

Clade M grouped three haplotypes (SM2-4) closely related to the type sequence of *D. muelleri* (SM1 = AJ438956[5]). Then, the fourth small clade (L) consisted of only three sequences (SL1-3). Clade X grouped sequences retrieved only from GenBank and detected only in the Baikal host *Gmelinoides fasciatus*. Clade B clustered together 5 haplotypes close to the type sequence of *D. berillonum* (SB5 = AJ438957[5]). These microsporidia were the most common parasite in our material (over 56% off all *Dictyocoela* positive records) and they were detected only in the Ponto-Caspian hosts (61 individuals): *Dikerogammarus haemobaphes*, *D. villosus*, *Pontogammarus robustoides* and *Echinogammarus trichiatus*. Finally, clade T consisted of 4 haplotypes found almost exclusively in talitrid amphipods. None of the new sequences found in our study grouped within this clade.

The Maximum Likelihood tree did not differ substantially in the topology from the Bayesian one and most of its major clades, but the clade D, were supported by high bootstrap values.

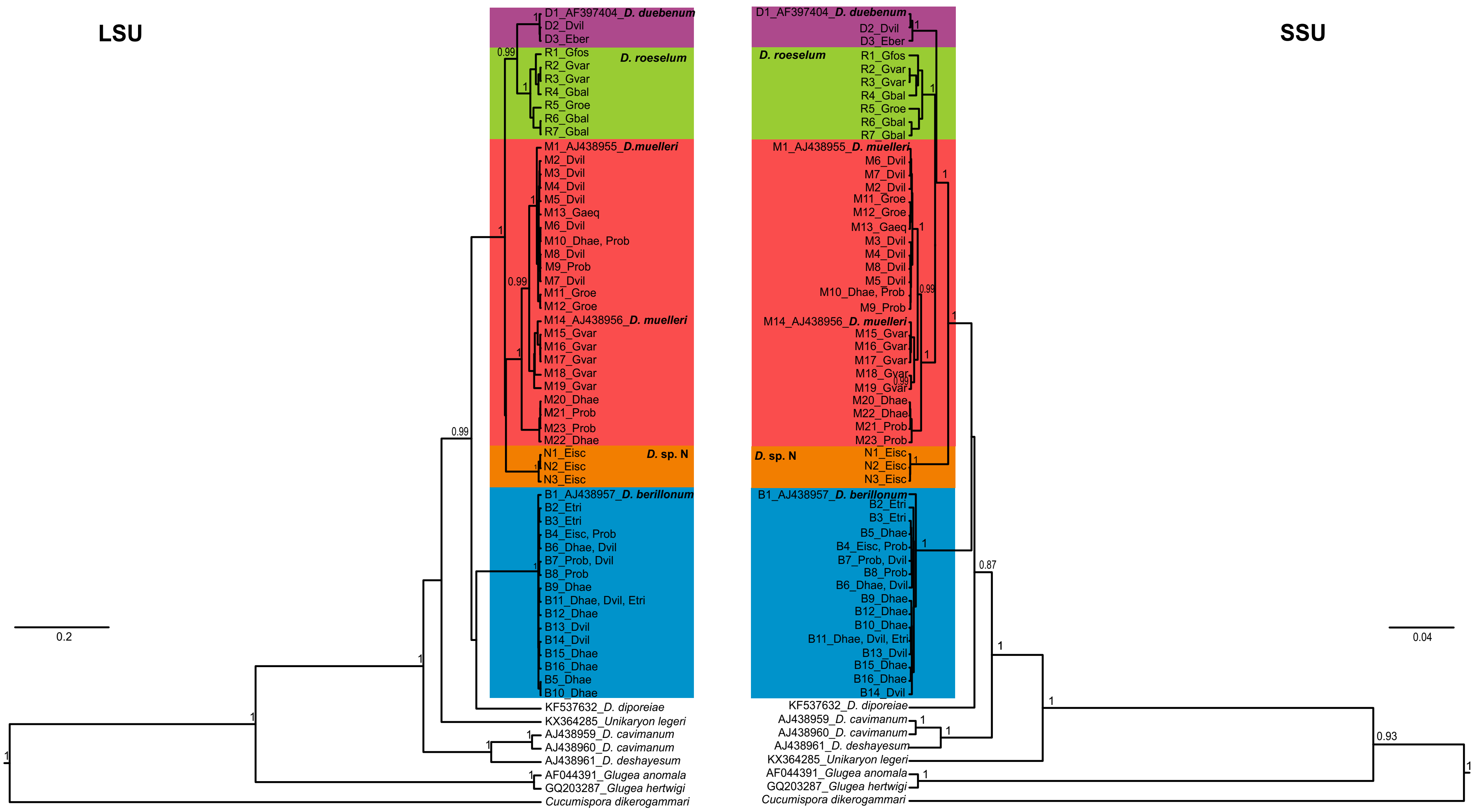

Supplementary Figure S2. Bayesian phylogeny of *Dictyocoela* spp. reconstructed using SSU rDNA (ca 1300 bp) and LSU (ca 400 bp) rDNA separately. Above the branches the Bayesian posterior probabilities for major clades higher than 0.75 are given. The colour code used for identification of major clades as in Figs. 1, 2 and Supplementary Fig. 1a.

Supplementary Data 1. Alignments used in the study: 1 – based on 524 bp long SSU rDNA fragment; 2. based on SSU, ITS and partial LSU rDNA (ca 1800 bp); 3 – used for analysis of molecular diagnostic features made in BOLD.

Alignment 1: SSU_524bp

```
>FJ820205_B1FAS1FAS1
CCAGGACCAAGGTCAGCAGCAGGCGCGAAAATTATCGAAGCCCGCC-GAGGGGCGATAGTGAGGAGACGTG-
TATAACGAAGTACGTGTAAAGAACGTGCTAATAACTGGAGGTCAAGTCTGGTGCCAGCATCCGCGGTAATTCC
AGCTCCAGGGGTGTCTATGATGATTGCTGCGATTAAAAAGTCCGTAGTCAAGCTGACTG-
ACTTGCCTGCAATG-
TGATTGATTAAAGAACGAGCAGGGCTAGGAAAGCAGAGAATTAGGAGCGACCGAGGGCTAGAGTATTGAATGG
CGAGAGGTGAAATTTGATGACCCATTCAGGAGTGACAAAGGCGAAGGCACTAGTCAAGGGCGAATCCGATGAT
CAAGGACGTAGGCTAGAGTTTCGAAAACGATTAGAGACCGGAGTAGTTCTAGTAGTAAACTATGCCGACGCCG
TGGTATTA------ATAT-TAGTATTGCGGAAGAGAAATC--AAGT-AAGGCTTT-
GGGGAGAGTACGCGCGCAAGCGATAAATT
>FJ820211_B3FAS1FAS2
CCAGGACCAAGGTCAGCAGCAGGCGCGAAAATTATCGAAGCCCGCC-GAGGGGCGATAGTGAGGAGACGTG-
TATAACGAAGTACGTGTAAAGAACGTGCTAATAACTGGAGGTCAAGTCTGGTGCCAGCATCCGCGGTAATTCC
AGCTCCAGGGGTGTCTATGATGATTGCTGCGATTAAAAAGTCCGTAGTCAAGCTGACTG-
ACTTGCCTGCAATG-
TGATTGATTAAAGAACGAGCAGGGCTAGGAAAGCAGAGAATTAGGAGCGACCGAGGGCTAGAGTATTGAATGG
CGAGAGGTGAAATTTGATGACCCATTCAGGAGTGACAAAGGCGAAGGCACTAGTCAAGGGCGAATCCGATGAT
CAAGGACGTAGGCTAGAGTTTCGAAAACGATTAGAGACCGGAGTAGTTCTAGTAGTAAACTATGCCTACGCCG
TGGTATTA------ATAT-TAGTATTGCGGAAGAGAAATC--AAGT-AAGGCTTT-
GGGGAGAGTACGCGCGCAAGCGATAAATT
>FJ820216_BFAS1FAS10
CCAGGACCAAGGTCAGCAGCAGGCGCGAAAATTATCGAAGCCCGCC-GAGGGGCGATAGTGAGGAGACGTG-
TATAACGAAGTACGTGTAAAGAACGTGCTAATAAATGGAGGTCAAGTCTGGTGCCAGCATCCGCGGTAATTCC
AGCTCCAGGGGTGTCTATGATGATTGCTGCGATTAAAAAGTCCGTAGTCAAGCTGACTG-
ACTTGCCTGCAATG-
TGATTGATTAAAGAACGAGCAGGGCTAGGAAAGCAGAGAATTAGGAGCGACCGAGGGCTAGAGTATTGAATGG
CGAGAGGTGAAATTTGATGACCCATTCAGGAGTGACAAAGGCGAAGGCACTAGTCAAGGGCGAATCCGATGAT
CAAGGACGTAGGCTAGAGTTTCGAAAACGATTAGAGACCGGAGTAGTTCTAGCAGTAAACTATGCCGACACCG
TGGTATTA------ATAT-TAGTATTGCGGAAGAGAAATC--AAGT-AAGGCTTT-
GGGGAGAGTACGCGCGCAAGCGATAAATT
>FJ820214_BFAS1FAS9
CCAGGACCAAGGTCAGCAGCAGGCGCGAAAATTATCGAAGCCCGCC---AGGGCGATAGTGAGGAGACGTG-
TATAACGAAGTACGTGTAAAGAACGTGCTAATAAATGGAGGTCAAGTCTGGTGCCAGCATCCGCGGTAATTCC
AGCTCCAGGGGTGTCTATGATGATTGCTGCGATTAAAAAGTCCGTAGTCAAGCTGACTG-
ACTTGCCTGCAATG-
TGATTGATTAAAGAACGAGCAGGGCTAGGAAAGCAGAGAATTAGGAGCGACCGAGGGCTAGAGTATTGAATGG
CGAGAGGTGAAATTTGATGACCCATTCAGGAGTGACAAAGGCGAAGGCACTAGTCAAGGGCGAATCCGATGAT
CAAGGACGTAGGCTAGAGTTTCGAAAACGATTAGAGACCGGAGTAGTTCTAGCAGTAAACTATGCCGACACCG
TGGTATTA------ATAT-TAGTATTGCGGAAGAGAAATC--AAGT-AAGGCTTT-
GGGGAGAGTACGCGCGCAAGCGATAAATT
>FJ820213_BFAS1FAS3
CCAGGACCAAGGTCAGCAGCAGGCGCGAAAATTATCGAAGCCCGCC-GAGGGGCGATAGTGAGGAGACGTG-
TATAACGAAGTACGTGTAAAGAACGTGCTAATAACTGGAGGTCAAGTCTGGTGCCAGCATCCGCGGTAATTCC
AGCTCCAGGGGTGTCTATGATGATTGCTGCGATTAAAAAGTCCGTAGTCAAGCTGACTG-
ACTTGCCCGCAATG-
TGGTTGATTAAAGAACGAGCAGGGCTAGGAAAGCAGAGAATTAGGAGCGACCGAGGGCTAGAGTATTGAATGG
CGAGAGGTGAAATTTGATGACCCATTCAGGAGTGACAAAGGCGAAGGCACTAGTCAAGGGCGAATCCGATGAT
CAAGGACGTAGGCTAGAGTTTCGAAAACGATTAGAGACCGGAGTAGTTCTAGTAGTAAACTATGCCGAC-
CCGTGGTATTA------ATAT-TAGTATTGCGGAAGAGAAATC--AAGT-AAGGCTTT-
GGGGAGAGTACGCGCGCAAGCGATAAATT
>EISC_PLZWC12
```

CCAGGACCAAGGTCAGCAGCAGGCGCGAAAATTATCGAAGCCCGCC-GAGGGGCGATAGTGAGGAGACGTG-
TATAACGAAGTACGTGTAAAGAACGTGCTAACAACTGGAGGTCAAGTCTGGTGCCAGCATCCGCGGTAATTCC
AGCTCCAGGGGTGTCTATGATGATTGCTGCGATTAAAAAGTCCGTAGTCAATCTGTCTG-
ACTTGCCTGCAATG-
TGATTGATTAAAGAACGAGCAGGGCTAGGAAAGCAGAGAATTAGGAGCGACCGAGGGCTAGAGTATTGAATGG
CGAGAGGTGAAATTTGATGACCCATTCAGGAGTGACAAAGGCGAAGGCACTAGTCAAGGGCGAATCCGATGAT
CAAGGACGTAGGCTAGAGTTTCGAAAACGATTAGAGACCGGAGTAGTTCTAGCAGTAAACTATGCCGACGCCG
TGGTATGT------ATTT-ATGTATTGCGGAAGAGAAATC--AAGT-AAGGCTTT-
GGGGAGAGTACGCGCGCAAGCGATAAATT
>FJ820206_BFAS2FAS1
CCAGGACCAAGGTCAGCAGCAGGCGCGAAAATTATCGAAGCCCGCC-TAGGGGCGATAGTGAGGAGACGTG-
TATAACGAAGTACGTGTAAAGAACGTGCTAATAACTGGAGGTCAAGTCTGGTGCCAGCATCCGCGGTAATTCC
AGCTCCAGGGGTGTCTATGATGATTGCTGCGATTAAAAAGTCCGTAGTCAATCTGACTG-
ACTGGTCTGCAATG-
TGATTGATTAAAGAACGAGCAGGGTTAGGAAAGCAGAGAATTAGGAGCGACCGAGGGCTAGAGTATTGAATGG
CGGGAGGTGAAATTTGATGACCCATTCAGGAGTGACAAAGGCGAAGGCACTAGTCAAGGGCGAATCCGATGAT
CAAGGACGTGGGCTAGAGTTTCGAAAACGATTAGAGACCGGAGTAGTTCTAGCAGTAAACTATGCCGACGCCG
TGGTATCA-------GAT-TGGTATTGCGGAAGAGAAATC--AAGT-AAGGCTTT-
GGGGAGAGTACGCGCGCAAGCGATAAATT
>HQ681048_Dsp15JEI-2011
CCAGGACCAAGGTCAGCAGCAGGCGCGAAAATTATCGAAGCCCGCC-TAGGGGCGATAGTGAGGAGACGTG-
TATAACGAAGTACGTGTAAAGAACGTGCTAATAACTGGAGGTCAAGTCTGGTGCCAGCATCCGCGGTAATTCC
AGCTCCAGGGGTGTCTATGATGATTGCTGCGATTAAAAAGTCCGTAGTCAATCTGACTG-
ACTGGCCTGCAATG-
TGATTGATTAAAGAACGAGCAGGGTTAGGAAAGCAGAGAATTAGGAGCGACCGAGGGCTAGAGTATTGAATGG
CGAGAGGTGAAATTTGATGACCCATTCAGGAGTGACAAAGGCGAAGGCACTAGTCAAGGGCGAATCCGATGAT
CAAGGACGTAGGCTAGAGTTTCGAAAACGATTAGAGACCGGAGTAGTTCTAGCAGTAAACTATGCCGACGCCG
TGGTATCA-------GAT-TGGTATTGCGGAAGAGAAATC--AAGT-AAGGCTTT-
GGGGAGAGTACGCGCGCAAGCGATAAATT
>JQ673483_Ddue
CCAGGACCAAGGTCAGCAGCAGGCGCGAAAATTATCGAAGCCCGCC-TAGGGGCGATAGTGAGGAGACGTG-
TATAACGAAGTACGTGTAAAGAACGTGCTAATAACTGGAGGTCAAGTCTGGTGCCAGCATCCGCGGTAATTCC
AGCTCCAGGGGTGTCTATGATGACTGCTGCGATTAAAAAGTCCGTAGTCAATCTGACTG-
ACTGGCCTGCAATG-
TGATTGATTAAAGAACGAGCAGGGTTAGGAAAGCAGAGAATTAGGAGCGACCGAGGGCTAGAGTATTGAATGG
CGAGAGGTGAAATTTGATGACCCATTCAGGAGTGACAAAGGCGAAGGCACTAGTCAAGGGCGAATCCGATGAT
CAAGGACGTAGGCTAGAGTTTCGAAAACGATTAGAGACCGGAGTAGTTCTAGCAGTAAACTATGCCGACGCCG
TGGTATCA-------GAT-TGGTATTGCGGAAGAGAAATC--AAGT-AAGGCTTT-
GGGGAGAGTACGCGCGCAAGCGATAAATT
>HQ681045_Dsp11JEI-2011
CCAGGACCAAGGTCAGCAGCAGGCGCGAAAATTATCGAAGCCCGCC-TAGGGGCGATAGTGAGGAGACGTG-
TATAACGAAGTACGTGTAAAGAACGTGCTAATAACTGGAGGTCAAGTCTGGTGCCAGCATCCGCGGTAATTCC
AGCTCCAGGGGTGTCTATGATGATTGCTGCGATTAAAAAGTCCGTAGTCAATCTGACTG-
ACTGACCTGCAATG-
TGATTGATTAAAGAACGAGCAGGGTTAGGAAAGCAGAGAATTAGGAGCGACCGAGGGCTAGAGTATTGAATGG
CGAGAGGTGAAATTTGATGACCCATTCAGGAGTGACAAAGGCGAAGGCACTAGTCAAGGGCGAATCCGATGAT
CAAGGACGTAGGCTAGAGTTTCGAAAACGATTAGAGACCGGAGTAGTTCTAGCAGTAAACTATGCCGACGCCG
TGGTATTA-------TTT-TAGTATTGCGGAAGAGAAATC--AAGT-AAGGCTTT-
GGGGAGAGTACGCGCGCAAGCGATAAATT
>FJ756216_DspBLACVER
CCAGGACCAAGGTCAGCAGCAGGCGCGAAAATTATCGAAGCCCGCC-TAGGGGCGATAGTAAGGAGACGTG-
TATAACGAAGTACGTGTAAAGAACGTGCTAATAACTGGAGGTCAAGTCTGGTGCCAGCATCCGCGGTAATTCC
AGCTCCAGGGGTGTCTATGATGATTGCTGCGATTAAAAAGTCCGTAGTCAATCTGACTG-
ACTGACCTGCAATG-
TGATTGATTAAAGAACGAGCAGGGTTAGGAAAGCAGAGAATTAGGAGCGACCGAGGGCTAGAGTATTGAATGG
CGAGAGGTGAAATTTGATGACCCATTCAGGAGTGACAAAGGCGAAGGCACTAGTCAAGGGCGAATCCGATGAT
CAAGGACGTAGGCTAGAGTTTCGAAAACGATTAGAGACCGGAGTAGTTCTAGCAGTAAACTATGCCGACGCCG

TGGTATTA-------TTT-TAGTATTGCGGAAGAGAAATC--AAGT-AAGGCTTT-
GGGGAGAGTACGCGCGCAAGCGATAAATT
>HQ681044_Dsp10JEI-2011
CCAGGACCAAGGTCAGCAGCAGGCGCGAAAATTATCGAAGCCCGCC-TAGGGGCGATAGTGAGGAGACGTG-
TATAACGAAGTACGTGTAAAGAACGTGCTAATAACTGGAGGTCAAGTCTGGTGCCAGCATCCGCGGTAATTCC
AGCTCCAGGGGTGTCTATGATGATTGCTGCGATTAAAAAGTCCGTAGTCAATCTGACTG-
ACTGACCTGCAATG-
TGATTGATTAAAGAACGAGCAGGGTTAGGAAAGCAGAGAATTAGGAGCGACCGAGGGCTAGAGTATTGAATGG
CGAGAGGTGAAATTTGATGACCCATTCAGGAGTGACAAAGGCGAAGGCACTAGTCAAGGGCGAATCCGATGAT
CAAGGACGTAGGCTAGAGTTTCGAAAACGATTAGAGACCGGAGTAGTTCTAGCAGTAAACTATGCCGACGCCG
TGGTATCA-------TTT-TGGTATTGCGGAAGAGAAATC--AAGT-AAGGCTTT-
GGGGAGAGTACGCGCGCAAGCGATAAATT
>HQ681043_Dsp9JEI-2011
CCAGGACCAAGGTCAGCAGCAGGCGCGAAAATTATCGAAGCCCGCC-TAGGGGCGATAGTGAGGAGACGTG-
TATAACGAAGTACGTGTAAAGAACGTGCTAATAACTGGAGGTCAAGTCTGGTGCCAGCATCCGCGGTAATTCC
AGCTCCAGGGGTGTCTATGATGATTGCTGCGATTAAAAAGTCCGTAGTCAATCTGACTG-
ACTGGCCTGCAATG-
TGATTGATTAAAGAACGAGCAGGGTTAGGAAAGCAGAGAATCAGGAGCGACCGAGGGCTAGAGTATTGAATGG
CGAGAGGTGAAATTTGATGACCCATTCAGGAGTGACAAAGGCGAAGGCACTAGTCAAGGGCGAATCCGATGAT
CAAGGACGTAGGCTAGAGTTTCGAAAACGATTAGAGACCGGAGTAGTTCTAGCAGTAAACTATGCCGACGCCG
TGGTATCA-------TTT-TGGTATTGCGGAAGAGAAATC--AAGT-AAGGCTTT-
GGGGAGAGTACGCGCGCAAGCGATAAATT
>FJ756212_DspBCYACYA1
CCAGGACCAAGGTCAGCAGCAGGCGCGAAAATTATCGAAGCCCGCC-TAGGGGCGATAGTGAGGAGACGTG-
TATAACGAAGTACGTGTAAAGAACGTGCTAGTAACTGGAGGTCAAGTCTGGTGCCAGCATCCGCGGTAATTCC
AGCTCCAGGGGTGTCTATGATGATTGCTGCGATTAAAAAGTCCGTAGTCAATCTGACTG-
ACTGGCCTGCAATG-
TGATTGATTAAAGAACGAGCAGGGTTAGGAAAGCAGAGAATCAGGAGCGACCGAGGGCTAGAGTATTGAATGG
CGAGAGGTGAAATTTGATGACCCATTCAGGAGTGACAAAGGCGAAGGCACTAGTCAAGGGCGAATCCGATGAT
CAAGGACGTAGGCTAGAGTTTCGAAAACGATTAGAGACCGGAGTAGTTCTAGCAGTAAACTATGCCGACGCCG
TGGTATCA-------TTT-TGGTATTGCGGAAGAGAAATC--AAGT-AAGGCTTT-
GGGGAGAGTACGCGCGCAAGCGATAAATT
>KP091740_Ddue
CCAGGACCAAGGTCAGCAGCAGGCGCGAAAATTATCGAAGCCCGCC-TAGGGGCGATAGTGAGGAGACGTG-
TATAACGAAGTACGTGTAAAGAACGTGCTAATAACTGGAGGTCAAGTCTGGTGCCAGCATCCGCGGTAATTCC
AGCTCCAGGGGTGTCTATGATGATTGCTGCGATTAAAAAGTCCGTAGTCAATCTGACTG-
ACTGGCGTGCAATG-
TGATTGATTAAAGAACGAGCAGGGTTAGGAAAGCAGAGAATCAGGAGCGACCGAGGGCTAGAGTATTGAATGG
CGAGAGGTGAAATTTGATGACCCATTCAGGAGTGACAAAGGCGAAGGCACTAGTCAAGGGCGAATCCGATGAT
CAAGGACGTAGGCTAGAGTTTCGAAAACGATTAGAGACCGGAGTAGTTCTAGCAGTAAACTATGCCGACGCCG
TGGTATCG-------ATT-TGGTATTGCGGAAGAGAAATC--AAGT-AAGGCTTT-
GGGGAGAGTACGCGCGCAAGCGATAAATT
>KR871355_Ddue1
CCAGGACCAAGGTCAGCAGCAGGCGCGAAAATTATCGAAGCCCGCC-TAGGGGCGATAGTGAGGAGACGTG-
TATAACGAAGTACGTGTAAAGAACGTGCTAATAACTGGAGGTCAAGTCTGGTGCCAGCATCCGCGGTAATTCC
AGCTCCAGGGGTGTCTATGATGATTGCTGCGATTAAAAAGTCCGTAGTCAATCTGACTG-
ACTGGCCTGCAATG-
TGATTGATCAAAGAACGAGCAGGGTTAGGAAAGCAGAGAATCAGGAGCGACCGAGGGCTAGAGTATTGAATGG
CGAGAGGTGAAATTTGATGACCCATTCAGGAGTGACAAAGGCGAAGGCACTAGTCAAGGGCGAATCCGATGAT
CAAGGACGTAGGCTAGAGTTTCGAAAACGATTAGAGACCGGAGTATTTCTAGCAGTAAACTATGCCGACGCCG
TGGTATCG-------ATT-TGGTATTGCGGAAGAGAAATC--AAGT-AAGGCTTT-
GGGGAGAGTACGCGCGCAAGCGATAAATT
>KR871380_Ddue
CCAGGACCAAGGTCAGCAGCAGGCGCGAAAATTATCGAAGCCCGCC-TAGGGGCGATAGTGAGGAGACGTG-
TATAACGAAGTACGTGTAAAGAACGTGCTAATAACTGGAGGTCAAGTCTGGTGCCAGCATCCGCGGTAATTCC
AGCTCCAGGGGTGTCTATGATGATTGCTGCGATTAAAAAGTCCGTAGTCAATCTGACTG-
ACTGGCCTGCAATG-
TGATTGATCAAAGAACGAGCAGGGTTAGGAAAGCAGAGAATCAGGAGCGACCGAGGGCTAGAGTATTGAATGG

CGAGAGGTGAAATTTGATGACCCATTCAGGAGTGACAAAGGCGAAGGCACTAGTCAAGGGCGAATCCGATGAT
CAAGGACGTAGGCTAGAGTTTCGAAAACGATTAGAGACCGGAGTAGTTCTAGCAGTAAACTATGCCGACGCCG
TGGTATCG-------ATT-TGGTATTGCGGAAGAGAAATC--AAGT-AAGGCTTT-
GGGGAGAGTACGCGCGCAAGCGATAAATT
>FJ756213_DspBCYACYA2
CCAGGACCAAGGACGGCAGCAGGCGCGAAAATTATCGAAGCCCGCC-TAGGGGCGATAGTGAGGAGACGTG-
TACAACGAAGTACGTGTAAAGAACGTGCTAGTAACTGGAGGTCAAGTCTGGTGCCAGCATCCGCGGTAATTCC
AGCTCCAGGGGTGTCTATGATGATTGCTGCGATTAAAAAGTCCGTAGTCAATCTGACTG-
ACTGGCCTGCAATG-
TGATTGATTAAAGAACGAGCAGGGTTAGGAAAGCAGAGAATCAGGAGCGGCCGAGGGCTAGAGTATTGAATGG
CGAGAGGTGAAATTTGATGACCCATTCAGGAGTGACAAAGGCGAAGGCACTAGTCAAGGGCGAATCCGATGAT
CAAGGACGTAGGCTAGAGTTTCGAAAACGATTAGAGACCGGAGTAGTTCTAGCAGTAAACTATGCCGACGCCG
TGGTATCA-------TTT-TGGTATTGCGGAAGAGAAATC--AAGT-AAGGCTTT-
GGGGAGAGTGCGCGCGCATGCGATAAATT
>HQ681046_Dsp12JEI-2011
CCAGGACCAAGGTCAGCAGCAGGCGCGAAAATTATCGAAGCCCGCC-TAGGGGCGATAGTGAGGAGACGTG-
TATAACGAAGTACGTGTAAAGAACGTGCTAATAACTGGAGGTCAAGTCTGGTGCCAGCATCCGCGGTAATTCC
AGCTCCAGGGGTGTCTATGATGATTGCTGCGATTAAAAAGTCCGTAGTCAATCTGACTG-
ACTGACCTGCAATG-
TGATTGATTAAAGAACGAGCAGGGTTAGGAAAGCAGAGAATTAGGAGCGACCGAGGGCTAGAGTATTGAATGG
CGAGAGGTGAAATTTGATGACCCATTCAGGAGTGACAAAGGCGAAGGCACTAGTCAAGGGCGAATCCGATGAT
CAAGGACGTAGGCTAGAGTTTCGAAAACGATTAGAGACCGGAGTAGTTCTAGCAGTAAACTATGCCGACGCCG
TGGTATTA-------TTT-TAGTATTGCGGAAGAGAAATC--AA-T-AAGGCTTT-
GGGGAGAGTACGCGCGCAAGCGATAAATT
>FJ756215_DspBLACLAC2
CCAGGACCAAGGTCAGCAGCAGGCGCGAAAATTATCGAAGCCCG-C-TAGGGGCGATAGTGAGGAGACGTG-
TATAACGAAGTACGTGTAAAGAACGTGCTAATAACTGGAGGTCAAGTCTGGTGCCAGCATCCGCGGTAATTCC
AGCTCCAGGGGTGTCTATGATGATTGCTGCGATTAAAAAGTCCGTAGTCAATCTGACTG-
ACTGACCTGCAATG-
TGATTGATTAAAGAACGAGCAGGGTTAGGAAAGCAGAGAATTAGGAGCGACCGAGGGCTAGAGTATTGAATGG
CGAGAGGTGAAATTTGATGACCCATTCAGGAGTGACAAAGGCGAAGGCACTAGTCAAGGGCGAATCCGATGAT
CAAGGACGTAGGCTAGAGTTTCGAAAACGATTAGAGACCGGAGTAGTTCTAGCAGTAAACTATGCCGACGCCG
TGGTATTA-------TTT-TAGTATTGCGGAAGAGAAATC--AAGT-AAGGCTTT-
GGGGAGAGTACGCGCGCAAGCGATAAATT
>KT956055_DspGLI-1
CCAGGACCAAGGTCAGCAGCAGGCGCGAAAATTATCGAAGCCCGCC-TAGGGGCGATAGTGAGGAGACGTG-
TATAACGAAGTACGTGTAAAGAACGTGCTAATAACTGGAGGTCAAGTCTGGTGCCAGCATCCGCGGTAATTCC
AGCTCCAGGGGTGTCTATGATGATTGCTGCGATTAAAAAGTCCGTAGTCAATCTGACTG-
ACTGACCTGCAATG-
TGATTGATTAAAGAACGAGCAGGGTTAGGAAAGCAGAGAATTAGGAGCGACCGAGGGCTAGAGTATTGAATGG
CGAGAGGTGAAATTTGATGACCCATTCAGGAGTGACAAAGGCGAAGGCACTAGTCAAGGGCGAATCCGATGAT
CAAGGACGTAGGCTAGAGTTTCGAAAACGATTAGAGACCGGAGTAGTTCTAGCAGTAAACTATGCCGACGCCG
TGGTATCA-------TTT-TAGTATTGCGGAAGAGAAATC--AAGT-
AAGGCTTTGGGGGAGAGTACGCGCGCAAGCGATAAATT
>FJ820233_BFAS2FAS2
CCAGGACCAAGGTCAGCAGCAGGCGCGAAAATTATCGAAGCCCGCC-TAGGGGCGATAGTGAGGAGACGTG-
TATAACGAAGTACGTGTAAAGAACGTGCTAATAACTGGAGGTCAAGTCTGGTGCCAGCATCCGCGGTAATTCC
AGCTCCAGGGGTGTCTATGATGATTGCTGCGATTAAAAAGTCCGTAGTCAATCTGACTG-
GCTGGCCTGCAATG-
TGATTGATTAAAGAACGAGCAGGGTTAGGAAAGCAGAGAATTAGGAGCGACCGAGGGCTAGAGTATTGAATGG
CGAGAGGTGAAATTTGATGACCCATTCAGGAGTGACAAAGGCGAAGGCACTAGTCAAGGGCGAATCCGATGAT
CAAGGACGTAGGCTAGAGTTTCGAAAACGATTAGAGACCGGAGTAGTTCTAGCAGTAAACTATGCCGACGCCG
TGGTATCA------GAAT-TGGTATTGCGGAAGAGAAATC--AAGT-AAGGCTTT-
GGGGAGAGTACGCGCGCAAGCGATAAATT
>KR871358_Ddue4
CCAGGACCAAGGTCAGCAGCAGGCGCGAAAATTATCGAAGCCCGCC-TAGGGGCGATAGTGAGGAGACGTG-
TATAACGAAGTACGTGTAAAGAACGTGCTAATAACTGGAGGTCAAGTCTGGTGCCAGCATCCGCGGTAATTCC
AGCTCCAGGGGTGTCTATGATGATTGCTGCGATTAAAAAGTCCGTAGTCAATCTGACTG-

ACTGACCTGCAATG-
TGATTGATTAAAGAACGAGCAGGGTTAGGAAAGCAGAGAATTAGGAGCGACCGAGGGCTAGAGTATTGAATGG
CGAGAGGTGAAATTTGATGACCCATTCAGGAGTGACAAAGGCGAAGGCACTAGTCAAGGGCGAATCCGATGAT
CAAGGACGTAGGCTAGAGTTTCGAAAACGATTAGAGACCGGAGTAGTTCTAGCAGTAAACTATGCCGACGCCG
TGGTATCA------ACAA-TGGTATTGCGGAAGAGAAATC--AAGT-AAGGCTTT-
GGGGAGAGTACGCGCGCAAGCGATAAATT
>FJ756203_DspBLAPPAR2
CCAGGACCAAGGTCAGCAGCAGGCGCGAAAATTATCGAAGCCCGCC-TAGGGGCGATAGTGAGGAGACGTG-
TATAACGAAGTACGTGTAAAGAACGTGCTAATAACTGGAGGTCAAGTCTGGTGCCAGCATCCGCGGTAATTCC
AGCTCCAGGGGTGTCTATGATGATTGCTGCGATTAAAAAGTCCGTAGTCAATCTGACTG-
ACTGACCTGCAATG-
TGATTGATTAAAGAACGAGCAGGGTTAGGAAAGCAGAGAATTAGGAGCGACCGAGGGCTAGAGTATTGAATGG
CGAGAGGTGAAATTTGATGACCCATTCAGGAGTGACAAAGGCGAAGGCACTAGTCAAGGGCGAATCCGATGAT
CAAGGACGTAGGCTAGAGTTTCGAAAACGATTAGAGACCGGAGTAGTTCTAGCAGTAAACTATGCCGACGCCG
TGGTATCA------ACAA-TGGTATTGCGGAAGAGAAATC--AAGT-AAGGCTTT-
GGGGAGAGTACGCGCGCAAGCGAT-----
>FJ820236_B4FAS3FAS1
CCAGGACCAAGGTCAGCAGCAGGCGCGAAAATTATCGAAGCCCGCC-TAGGGGCGATAGTGAGGAGACGTG-
TATAACGAAGTACGTGTAAAGAACGTGCTAATAACTGGAGGTCAAGTCTGGTGCCAGCATCCGCGGTAATTCC
AGCTCCAGGGGTGTCTATGATGATTGCTGCGATTAAAAAGTCCGTAGTCAATCTGACTG-
ACTGACCTGCAATG-
TGATTGATTAAAGAACGAGCAGGGTTAGGAAAGCAGAGAATTAGGAGCGACCGAGGGCTAGAGTATTGAATGG
CGAGAGGTGAAATTTGATGACCCATTCAGGAGTGACAAAGGCGAAGGCACTAGTCAAGGGCGAATCCGATGAT
CAAGGACGTAGGCTAGAGTTTCGAAAACGATTAGAGACCGGAGTAGTTCTAGCAGTAAACTATGCCGACGCCG
TGGTATCA------ATAA-TGGTATTGCGGAAGAGAAATC--AAGT-AAGGCTTT-
GGGGAGAGTACGCGCGCAAGCGATAAATT
>HQ681037_Dsp2JEI-2011
CCAGGACCAAGGTCAGCAGCAGGCGCGAAAATTATCGAAGCCCGCC-TAGGGGCGATAGTGAGGAGACGTG-
TATAACGAAGTACGTGTAAAGGACGTGCTAATAACTGGAGGTCAAGTCTGGTGCCAGCATCCGCGGTAATTCC
AGCTCCAGGGGTGTCTATGATGATTGCTGCGATTAAAAAGTCCGTAGTCAATCTGACTG-
ACTGACCTGCAATG-
TGATTGATTAAAGAACGAGCAGGGTTAGGAAAGCAGAGAATTAGGAGCGACCGAGGGCTAGAGTATTGAATGG
CGAGAGGTGAAATTTGATGACCCATTCAGGAGTGACAAAGGCGAAGGCACTAGTCAAGGGCGAATCCGATGAT
CAAGGACGTAGGCTAGAGTTTCGAAAACGATTAGAGACCGGAGTAGTTCTAGCAGTAAACTATGCCGACGCCG
TGGTATCA------ACAA-TGGTATTGCGGAAGAGAAATC--AAGT-AAGGCTTT-
GGGGAGAGTACGCGCGCAAGCGATAAATT
>FJ756204_DspBLAPPAR3
CCAGGACCAAGGTCAGCAGCAGGCGCGAAAATTATCGAAGCCCGCC-TAGGGGCGATAGTGAGGAGACGTG-
TATAACGAAGTACGTGTAAAGAACGTGCTAATAACTGGAGGTCAAGTCTGGTGCCAGCATCCGCGGTAATTCC
AGCTCCAGGGGTGTCTATGATGATTGCTGCGATTAAAAAGTCCGTAGTCAATCTGACTG-
ACTGACCTGCAATG-
TGATTGATTAAAGAACGAGCAGGGTTAGGAAAGCAGAGAATTAGGAGCGACCGAGGGCTAGAGTATTGAATGG
CGAGAGGTGAAATTTGATGACCCATTCAGGAGTGACAAAGGCGAAGGCACTAGTCAAGGGCGAATCCGATGGT
CAAGGACGTAGGCTAGAGTTTCGAAAACGATTAGAGACCGGAGTAGTTCTAGCAGTAAACTATGCCGACGCCG
TGGTATCA------ACAA-TGGTATTGCGGAAGAGAAATC--AAGT-AAGGCTTT-
GGGGAGAGTACGCGCGCAAGCGATAAATT
>DEB2
CCAGGACCAAGGTCAGCAGCAGGCGCGAAAATTATCGAAGCCCGCC-TAGGGGCGATAGTGAGGAGACGTG-
TATAACGAAGTACGTGTAAAGAACGTGCTAATAACTGGAGGTCAAGTCTGGTGCCAGCATCCGCGGTAATTCC
AGCTCCAGGGGTGTCTATGATGATTGCTGCGATTAAAAAGTCCGTAGTCAATCTGACTG-
ACTGACCTGCAATG-
TGATTGATTAAAGAACGAGCAGGGTTAGGAAAGCAAAGAATTAGGAGCGACCGAGGGCTAGAGTATTGAATGG
CGAGAGGTGAAATTTGATGACCCATTCAGGAGTGACAAAGGCGAAGGCACTAGTCAAGGGCGAATCCGATGAT
CAAGGACGTAGGCTAAAGTTTCGAAAACGATTAGAGACCGGAGTAGTTCTAGCAGTAAACTATGCCGACGCCG
TGGTATCA------ACAA-TGGTATTGCGGAAGAGAAATC--AAGT-AAGGCTTT-
GGGGAGAGTACGCGCGCAAGCGATAAATT
>HQ681038_Dsp3JEI-2011

CCAGGCCCAAGGTCAGCAGCAGGCGCGAAAATTATCGAAGCCCGCC-TAGGGGCGATAGTGAGGAGACGTG-
TATAACGAAGTACGTGTAAAGAACGTGCTAATAACTGGAGGTCAAGTCTGGTGCCAGCATCCGCGGTAATTCC
AGCTCCAGGGGTGTCTATGATGATTGCTGCGATTAAAAAGTCCGTAGTCAATCTGACTG-
ACTGACCTGCAATG-
TGATTGATTAAAGAACGAGCAGGGTTAGGAAAGCAGAGAATTAGGAGCGACCGAGGGCTAGAGTATTGAATGG
CGAGAGGTGAAATTTGATGACCCATTCAGGAGTGACAAAGGCGAAGGCACTAGTCAAGGGCGAATCCGATGAT
CAAGGACGTAGGCTAGAGTTTCGAAAACGATTAGAGACCGGAGTAGTTCTAGCAGTAAACTATGCCGACGCCG
TGGTATCA------ACAA-TGGTATTGCGGAAGAGAAATC--AAGT-AAGGCTTT-
GGGGAGAGTACGCGCGCAAGCGATAAATT
>FJ756206_DspBLAPPAR5
CCAGGACCAAGGTCAGCAGCAGGCGCGAAAATTATCGAAGCCCGCC-TAGGGGCGATAGTGAGGAGACGTG-
TATAACGAAGTACGTGTAAAGAACGTGCTAATAACTGGAGGTCAAGTCTGGTGCCAGCATCCGCGGTAATTCC
AGCTCCAGGGGTGTATATGATGATTGCTGCGATTAAAAAGTCCGTAGTCAATCTGACTG-
ACTGACCTGCAATG-
TGATTGATTAAAGAACGAGCAGGGTTAGGAAAGCAGAGAATTAGGAGCGACCGAGGGCTAGAGTATTGAATGG
CGAGAGGTGAAATTTGATGACCCATTCAGGAGTGACAAAGGCGAAGGCACTAGTCAAGGGCGAATCCGATGAT
CAAGGACGTAGGCTAGAGTTTCGAAAACGATTAGAGACCGGAGTAGTTCTAGCAGTAAACTATGCCGACGCCG
TGGTATCA------ACAA-TGGTATTGCGGAAGAGAAATC--AAGT-AAGGCTTT-
GGGGAGAGTACGCGCGCAAGCGATAAATT
>FJ756208_DspBLAPPAR7
CCAGGACCAAGGTCAGCAGCAGGCGCGAAAATTATCGAAGCCCGCC-TAGGGGCGATAGTGAGGAGACGTG-
TATAACGAAGTACGTGTAAAGAACGTGCTAATAACTGGAGGTCAAGTCTGGTGCCAGCATCCGCGGTAATTCC
AGCTCCAGGGGTGTCTATGATGATGGCTGCGATTAAAAAGTCCGTAGTCAATCTGACTG-
ACTGACCTGCAATG-
TGATTGATTAAAGAACGAGCAGGGTTAGGAAAGCAGAGAATTAGGAGCGACCGAGGGCTAGAGTATTGAATGG
CGAGAGGTGAAATTTGATGACCCATTCAGGAGTGACAAAGGCGAAGGCACTAGTCAAGGGCGAATCCGATGAT
CAAGGACGTAGGCTAGAGTTTCGAAAACGATTAGAGACCGGAGTAGTTCTAGCAGTAAACTATGCCGACGCCG
TGGTATCA------ATAA-TGGTATTGCGGAAGAGAAATC--AAGT-AAGGCTTT-
GGGGAGAGTACGCGCGCAAGCGATAAATT
>HQ681041_Dsp7JEI-2011
CCAGGACCAAGGTCAGCAGCAGGCGCGAAAATTATCGAAGCCCGCC-TAGGGGCGATAGTGAGGAGACGTG-
TATAACGAAGTACGTGTAAAGAACGTGCTAATAACTGGAGGTCAAGTCTGGTGCCAGCATCCGCGGTAATTCC
AGCTCCAGGGGTGTCTATGATGATTGCTGCGATTAAAAAGTCCGTAGTCAATCTGACTG-
ACTGACCTGCAATG-
TGATTGATTAAAGAACGAGCAGGGTTAGGAAAGCAGAGAATTAGGAGCGACCGAGGGCTAGAGTATTGAATGG
CGAGAGGTCAAATTTGATGACCCATTCAGGAGTGACAAAGGCGAAGGCACTAGTCAAGGGCGAATCCGATGAT
CAAGGACGTAGGCTAGAGTTTCGAAAACGATTAGAGACCGGAGTAGTTCTAGCAGTAAACTATGCCGACGCCG
TGGTATCA------ATTA-TGGTATTGCGGAAGAGAAATC--AAGT-AAGGCTTT-
GGGGAGAGTACGCGCGCAAGCGATAAATT
>HQ681042_Dsp8JEI-2011
CCAGGACCAAGGTCAGCAGCAGGCGCGAAAATTATCGAAGCCCGCC-TAGGGGCGATAGTGAGGAGACGTG-
TATAACGAAGTACGTGTAAAGAACGTGCTAATAACTGGAGGTCAAGTCTGGTGCCAGCATCCGCGGTAATTCC
AGCTCCAGGGGTGTCTATGATGATTGCTGCGATTAAAAAGTCCGTAGTCAATCTGACTG-
ACTGACCTGCAATG-
TGATTGATTAAAGAACGAGCAGGGTTAGGAAAGCAGAGAATTAGGAGCGACCGAGGGCTAGAGTATTGAATGG
CGAGAGGTGAAATTTGATGACCCATTCAGGAGTGACAAAGGCGAAGGCACTAGTCAAGGGCGAATCCGATGAT
CAAGGACGTAGGCTAGAGTTTCGAAAACGATTAGAGACCGGAGTAGTTCTAGCAGTAAACTATGCCGACGCCG
TGGTATCA------TTTT-TGGTATTGCGGAAGAGAAATC--AAGT-AAGGCTTT-
GGGGAGAGTACGCGCGCAAGCGATAAATT
>HM991451_DspGPM1
CCAGGACCAAGGTCAGCAGCAGGCGCGAAAATTATCGAAGCCCGCC-TAGGGGCGATAGTGAGGAGACGTG-
TATAACGAAGTACGTGTAAAGAACGTGCTAATAACTGGAGGTCAAGTCTGGTGCCAGCATCCGCGGTAATTCC
AGCTCCAGGGGTGTCTATGATGATTGCTGCGATTAAAAAGTCCGTAGTCAATCTGACTG-
ACTGACCTGTAATG-
TGATTGATTAAAGAACGAACAGGGTTAGGAAAGCAGAGAATTAGGAGCGACCGAGGGCTAGAGTATTGAATGG
CGAGAGGTGAAATTTGATGACCCATTCAGGAGTGACAAAGGCGAAGGCACTAGTCAAGGGCGAATCCGATGAT
CAAGGACGTAGGCTAGAGTTTCGAAAACGATTAGAGACCGGAGTAGTTCTAGCAGTAAACTATGCCGACGCCG

TGGTATCA------AGTT-TGGTATTGCGGAAGAGAAATC--AAGT-AAGGCTTT-
GGGGAGAGTACGCGCGCAAGCGATAAATT
>KM977838_DspEM-2105
CCAGGACCAAGGTCAGCAGCAGGCGCGAAAATTATCGAAGCCCGCC-TAGGGGCGATAGTGAGGAGACGTG-
TATAACGAAGTACGTGTAAAGAACGTGCTAATAACTGGAGGTCAAGTCTGGTGCCAGCATCCGCGGTAATTCC
AGCTCCAGGGGTGTCTATGATGATTGCTGCGATTAAAAAGTCCGTAGTCAATCTGACTG-
ACTGACCTGCAATG-
TGATTGATTAAAGAACGAGCAGGGTTAGGAAAGCAGAGAATTAGGAGCGACCGAGGGCTAGAGTATTGAATGG
CGAGAGGTGAAATTTGATGACCCATTCAGGAGTGACAAAGGCGAAGGCACTAGTCAAGGGCGAATCCGATGAT
CAAGGACGTAGGCTAGAGTTTCGAAAACGATTAGAGACCGGAGTAGTTCTAGCAGTAAACTATGCCGACGCCG
TGGTATCA------ACAA-TGGTATTGCGGAAGAGAAATC--AAGT-AAGGC-TT-
GGGGAGAGTACGCGCGCAAGCGATAAATT
>FJ820237_BFAS3FAS2
CCAGGACCAAGGTCAGCAGCAGGCGCGAAAATTATCGAAGCCCGCC-TAGGGGCGATAGTGAGGAGACGTG-
TATAACGAAGTACGTGTAAAGAACGTGCTAATAACTGGAGGTCAAGTCTGGTGCCAGCATCCGCGGTAATTCC
AGCTCCAGGGGTGTCTATGATGATTGCTGCGATTAAAAAGTCCGTAGTCAATCTGACTG-
ACTGACCTGCAATG-
TGATTGATTAAAGAACGAGCAGGGTTAGGAAAGCAGAGAATTAGGAGCGACCGAGGGCTAGAGTATTGAATGG
CGAGAGGTGAAATTTGATGACCCATTCA-
GAGTGACAAAGGCGAAGGCACTAGTCAAGGGCGAATCCGATGATCAAGGACGTAGGCTAGAGTTTCGAAAACG
ATTAGAGACCGGAGTAGTTCTAGCAGTAAACTATGCCGACGCCGTGGTATCA------ATAA-
TGGTATTGCGGAAGAGAAATC--AAGT-AAGGCTTT-GGGGAGAGTACGCGCGCAAGCGATAAATT
>M56_Gvar
CCAGGACCAAGGTCAGCAGCAGGCGCGAAAATTATCGAAGCCCGCC-TAGGGGCGATAGTGAGGAGACGTG-
TATAACGAAGTACGTGTAAAGAACGTACTAATAACTGGAGGTCAAGTCTGGTGCCAGCATCCGCGGTAATTCC
AGCTCCAGGGGTGTCTATGATGATTGCTGCGATTAAAAAGTCCGTAGTCAAGCTGACTG-
ACTGACCTGCAATG-
TGATTGATTAAGGAACGAGCAGGGTTAGGAAAGCAGAGAATTAGGAGCGACCGAGGGCTAGAGTATTGAATGG
CGAGAGGTGAAATTTGATGACCCATTCAGGAGTGACAAAGGCGAAGGCACTAGTCAAGGGCGAATCCGATGAT
CAAGGACGTAGGCTAGAGTTTCGAAAACGATTAGAGACCGGAGTAGTTCTAGCAGTAAACTATGCCGACGCCG
TGATATAG------TTTG-TGGTATTGCGGAAGAGAAATC--AAGT-AAGGCTTT-
GGGGAGAGTACGCGCGCAAGCGATAAATT
>Dmul_Groe_AJ438956
CCAGGACCAAGGTCAGCAGCAGGCGCGAAAATTATCGAAGCCCGCC-TAGGGGCGATAGTGAGGAGACGTG-
TATAACGAAGTACGTGTAAAGAACGTACTAATAACTGGAGGTCAAGTCTGGTGCCAGCATCCGCGGTAATTCC
AGCTCCAGGGGTGTCTATGATGATTGCTGCGATTAAAAAGTCCGTAGTCAAGCTGCCTG-
ACTGACCTGCAATG-
TGATTGATTAAGGAACGAGCAGGGTTAGGAAAGCAGAGAATTAGGAGCGACCGAGGGCTAGAGTATTGAATGG
CGAGAGGTGAAATTTGATGACCCATTCAGGAGTGACAAAGGCGAAGGCACTAGTCAAGGGCGAATCCGATGAT
CAAGGACGTAGGCTAGAGTTTCGAAAACGATTAGAGACCGGAGTAGTTCTAGCAGTAAACTATGCCGACGCCG
TGGTATGGTTTTTTGTGG-CTGTATTGCGGAAGAGAAATC--AAGT-AAGGCTTT-
GGGGAGAGTACGCGCGCAAGCGATAAATT
>MB6
CCAGGACCAAGGTCAGCAGCAGGCGCGAAAATTATCGAAGCCCGCC-TAGGGGCGATAGTGAGGAGACGTG-
TATAACGAAGTACGTGTAAAGAACGTACTAATAACTGGAGGTCAAGTCTGGTGCCAGCATCCGCGGTAATTCC
AGCTCCAGGGGTGTCTATGATGATTGCTGCGATTAAAAAGTCCGTAGTCAAGCTGCCTG-
ACTGACCTGCAATG-
TGATTGATTAAGGAACGAGCAGGGTTAGGAAAGCAGAGAATTAGGAGCGACCGAGGGCTAGAGTATTGAATGG
CGAGAGGTGAAATTTGATGACCCATTCAGGAGTGACAAAGGCGAAGGCACTAGTCAAGGGCGAATCCGATGAT
CAAGGACGTAGGCTAGAGTTTTTGAAAACGATTAGAGACCGGAGTAGTTCTAGCAGTAAACTATGCCGACGCCG
TGGTATGGTTTTTTGTGG-CTGTATTGCGGAAGAGAAATC--AAGT-AAGGCTTT-
GGGGAGAGTACGCGCGCAAGCGATAAATT
>HQ681054_Dsp36JEI-2011
CCAGGACCAAGGTCAGCAGCAGGCGCGAAAATTATCGAAGCCCGCC-TAGGGGCGATAGTGAGGAGACGTG-
TATAACGAAGTACGTGTAAAGAACGTACTAATAACTGGAGGTCAAGTCTGGTGCCAGCATCCGTGGTAATTCC
AGCTCCAGGGGTGTCTATGATGATTGCTGCGATTAAAAAGTCCGTAGTCAAGCTGCCTG-
ACTGACCTGCAATG-
TGATTGATTAAGGAACGAGCAGGGTTAGGAAAGCAGAGAATTAGGAGCGACCGAGGGCTAGAGTATTGAATGG

CGAGAGGTGAAATTTGATGACCCATTCAGGAGTGACAAAGGCGAAGGCACTAGTCAAGGGCGAATCCGATGAT
CAAGGACGTAGGCTAGAGTTTCGAAAACGATTAGAGACCGGAGTAGTTCTAGCAGTAAACTATGCCGACGCCG
TGGTATGGTTTTTTGTGG-CTGTATTGCGGAAGAGAAATC--AAGT-AAGGCTTT-
GGGGAGAGTACGCGCGCAAGCGATAAATT
>MB5
CCAGGACCAAGGTCAGCAGCAGGCGCGAAAATTATCGAAGCCCGCC-TAGGGGCGATAGTGAGGAGACGTG-
TATAACGAAGTACGTGTAAAGAACGTACTAATAACTGGAGGTCAAGTCTGGTGCCAGCATCCGCGGTAATTCC
AGCTCCAGGGGTGTCTATGATGATTGCTGCGATTAAAAAGTCCGTAGTCAAGCTGCCTG-
ACTGACCTGCAATG-
TGATTGATTAAGGAACGAGCAGGGTTAGGAAAGCAGAGAATTAGGAGCGACCGAGGGCTAGAGTATTGAATGG
CGAGAGGTGAAATTTGATGACCCATTCAGGAGTGACAAAGGCGAAGGCACTAGTCAAGGGCGAATCCGATGAT
CAAGGACGTAGGCTAGAGTTTCGAAAACGATTAGAGACCGGAGTAGTTCTAGCAGTAAAATATGCCGACGCCG
TGGTATGGTTTTTTGTGG-CTGTATTGCGGAAGAGAAATC--AAGT-AAGGCTTT-
GGGGAGAGTACGCGCGCAAGCGATAAATT
>M48
CCAGGACCAAGGTCAGCAGCAGGCGCGAAAATTATCGAAGCCCGCC-TAGGGGCGATAGTGAGGAGACGTG-
TATAACGAAGTACGTGTAAAGAACGTACTAATAACTGGAGGTCAAGTCTGGTGCCAGCATCCGCGGTAATTCC
AGCTCCAGGGGTGTCTATGATGATTGCTGCGATTAAAAAGTCCGTAGTCAAGCTGACTG-
ACTGACCTGCAATG-
TGATTGATTAAGGAACGAGCAGGGTTAGGAAAGCAGAGAATTAGGAGCGACCGAGGGCTAGAGTATTGAATGG
CGAGAGGTGAAATTTGATGACCCATTCAGGAGTGACAAAGGCGAAGGCACTAGTCAAGGGCGAATCCGATGAT
CAAGGACGTAGGCTAGAGTTTCGAAAACGATTAGAGACCGGAGTAGTTCTAGCAGTAAACTATGCCGACGCCG
TGATATAGTTTTTTGTGG-CTGTATTGCGGAAGAGAAATC--AAGT-AAGGCTTT-
GGGGAGAGTACGCGCGCAAGCGATAAATT
>FN434090_Dmue733
CCAGGACCAAGGTCAGCAGCAGGCGCGAAAATTATCGAAGCCCGCC-TAGGGGCGATAGTGAGGAGACGTG-
TATAACGAAGTACGTGTAAAGAACGTACTAATAACTGGAGGTCAAGTCTGGTGCCAGCATCCGCGGTAATTCC
AGCTCCAGGGGTGTCTATGATGATTGCTGCGATTAAAAAGTCCGTAGTCAAGCTGACTG-
ACTGACCTGCAATG-
TGATTGATTAAGGAACGAGCAGGGTTAGGAAAGCAGAGAATTAGGAGCGACCGAGGGCTAGAGTATTGAATGG
CGAGAGGTGAAATTTGATGACCCATTCAGGAGTGACAAAGGCGAAGGCACTAGTCAAGGGCGAATCCGATGAT
CAAGGACGTAGGCTAGAGTTTCGAAAACGATTAGAGACCGGAGTAGTTCTAGCAGTAAACTATGCCGACGCCG
TGATATAGTTTTTTGTGG-CTGTGTTGCGGAAGAGAAATC--AAGT-AAGGCTTT-
GGGGAGAGTACGCGCGCAAGCGATAAATT
>KR871363_Ddue9
CCAGGACCAAGGTCAGCAGCAGGCGCGAAAATTATCGAAGCCCGCC-TAGGGGCGATAGTGAGGAGACGTG-
TATAACGAAGTACGTGTAAAGAACGTACTAATAACTGGAGGTCAAGTCTGGTGCCAGCATCCGCGGTAATTCC
AGCTCCAGGGGTGTCTATGATGATTGCTGCGATTAAAAAGTCCGTAGTCAAGCTGACTG-
ACTGACCTGCAATG-
TGATTGATTAAGGAACGAGCAGGGTTAGGAAAGCAGAGAATTAGGAGCGACCGAGGGCTAGAGTATTGAATGG
CGAGAGGTGAAATTTGATGACCCATTCAGGAGTGACAAAGGCGAAGGCACTAGTCAAGGGCGAATCCGATGAT
CAAGGACGTAGGCTAGAGTTTCGAAAACGATTAGAGACCGGAGTAGTTCTAGCAGTAAACTATGCCGACGCCG
CGATATAGTTTTTTGTGG-CTGTGTTGCGGAAGAGAAATC--AAGT-AAGGCTTT-
GGGGAGAGTACGCGCGCAAGCGATAAATT
>M33_Pr
CCAGGACCAAGGTCAGCAGCAGGCGCGAAAATTATCGAAGCCCGCC-TAGGGGCGATAGTGAGGAGACGTG-
TATAACGAAGTACGTGTAAAGAACGTACTAATAACTGGAGGTCAAGTCTGGTGCCAGCATCCGCGGTAATTCC
AGCTCCAGGGGTGTCTATGATGATTGCTGCGATTAAAAAGTCCGTAGTCAAACTGACTG-
ACTGACCTGCAATG-
TGATTGATTAAAGAACGAGCAGGGTTAGGAAAGCAGAGAATTAGGAGCGACCGAGGGCTAGAGTATTGAATGG
CGAGAGGTGAAATTTGATGACCCATTCAGGAGTGACAAAGGCGAAGGCACTAGTCAAGGGCGAATCCGATGAT
CAAGGACGTAGGCTAGAGTTTCGAAAACGATTAGAGACCGGAGTAGTTCTAGCAGTAAACTATGCCGACGCCG
TGATATTGTTTTTTGTGG-CAGTATTGCGGAAGAGAAATC--AAGT-AAAGCTTT-
GGGGAGAGTACGCGCGCAAGCGATAAATT
>M22_Dh
CCAGGACCAAGGTCAGCAGCAGGCGCGAAAATTATCGAAGCCCGCC-TAGGGGCGATAGTGAGGAGACGTG-
TATAACGAAGTACGTGTAAAGAACGTACTAATAACTGGAGGTCAAGTCTGGTGCCAGCATCCGCGGTAATTCC
AGCTCCAGGGGTGTCTATGATGATTGCTGCGATTAAAAAGTCCGTAGTCAAACTGACTG-

ACTGACCTGCAATG-
TGATTGATTAAAGAACGAGCAGGGTTAGGAAAGCAGAGAATTAGGAGCGACCGAGGGCTAGAGTATTGAATGG
CGAGAGGTGAAATTTGATGACCCATTCAGGAGTGACAAAGGCGAAGGCACTAGTCAAGGGCGAATCCGATGAT
CAAGGACGTAGGCTAGAGTTTCGAAAACGATTAGAGACCGGAGTAGTTCTAGCAGTAAACTATGCCGACGCCG
TGATATTGTTTTTTGTGG-CAGTATTGCGGAAGAGAAATC--AAGT-AAGGCTTT-
GGGGAGAGTACGCGCGCAAGCGATAAATT
>M34_Gaequi
CCAGGACCAAGGTCAGCAGCAGGCGCGAAAATTATCGAAGCCCGCC-TAGGGGCGATAGTGAGGAGACGTG-
TATAACGAAGTACGTGTAAAGAACGTACTAATAACTGGAGGTCAAGTCTGGTGCCAGCATCCGCGGTAATTCC
AGCTCCAGGGGTGTCTATGATGATTGCTGCGATTAAAAAGTCCGTAGTCAAACTGACTG-
ACTGACCTGCAATG-
TGATTGATTAAAGAACGAGCAGGGTTAGGAAAGCAGAGAATTAGGAGCGACCGAGGGCTAGAGTATTGAATGG
CGAGAGGTGAAATTTGATGACCCATTCAGGAGTGACAAAGGCGAAGGCACTAGTCAAGGGCGAATCCGATGAT
CAAGGACGTAGGCTAGAGTTTCGAAAACGATTAGAGACCGGAGTAGTTCTAGCAGTAAACTATGCCGACGCCG
TGATATTGTTTTTTGTGG-CGGTATTGCGGAAGAGAAATC--AAGT-AAGGCTTT-
GGGGAGAGTACGCGCGCAAGCGATAAATT
>GROE_ALPM41-1
CCAGGACCAAGGTCAGCAGCAGGCGCGAAAATTATCGAAGCCCGCC-TAGGGGCGATAGTGAGGAGACGTG-
TATAACGAAGTACGTGTAAAGAACGTACTAATAACTGGAGGTCAAGTCTGGTGCCAGCATCCGCGGTAATTCC
AGCTCCAGGGGTGTCTATGATGATTGCTGCGATTAAAAAGTCCGTAGTCAAACTGACTG-
ACTGACCTGCAATG-
TGATTGATTAAAGAACGAGCAGGGTTAGGAAAGCAGAGAATTAGGAGCGACCGAGGGCTAGAGTATTGAATGG
CGAGAGGTGAAATTTGATGACCCATTCAGGAGTGACAAAGGCGAAGGCACTAGTCAAGGGCGAATCCGATGAT
CAAGGACGTAGGCTAGAGTTTCGAAAACGATTAGAGACCGGAGTAGTTCTAGCAGTAAACTATGCCGACGCCG
TGATATTGTTTTTTGTGG-CGGTATTGCGGAAGAGAAATC--AAGT-AAAGCTTT-
GGGGAGAGTACGCGCGCAAGCGATAAATT
>HQ681061_Dsp44JEI-2011
CCAGGACCAAGGTCAGCAGCAGGCGCGAAAATTATCGAAGCCCGCC-TAGGGGCGATAGTGAGGAGACGTG-
TATAACGAAGTACGTGTAAAGAACGTACTAATAACTGGAGGTCAAGTCTGGTGCCAGCATCCGCGGTAATTCC
AGCTCCAGGGGTGTCTATGATGATTGCTGCGATTAAAAAGTCCGTAGTCAAACTGACTG-
ACTGACCTGCAATG-
TGATTGATTAAAGAACGAGCAGGGTTAGGAAAGCAGAGAATTAGGAGCGACCGAGGGCTAGAGTATTGAATGG
CGAGAGGTGAAATTTGATGACCCATTCAGGAGTGACAAAGGCGAGGGCACTAGTCAAGGGCGAATCCGATGAT
CAAGGACGTAGGCTAGAGTTTCGAAAACGATTAGAGACCGGAGTAGTTCTAGCAGTAAACTATGCCGACGCCG
TGACATTGTTTTTTGTGG-CAGTATTGCGGAAGAGAAATC--AAGT-AAGGCTTT-
GGGGAGAGTACGCGCGCAAGCGATAAATT
>HQ683744_Dsp31
CCAGGACCAAGGTCAGCAGCAGGCGCGAAAATTATCGAAGCCCGCC-TAGGGGCGATAGTGAGGAGACGTG-
TATAACGAAGTACGTGTAAAGCACGCACTAATAACTGGAGGTCAAGTCTGGTGCCAGCATCCGCGGTAATTCC
AGCTCCAGGGGTGTCTATGATGATTGCTGCGATTAAAAAGTCCGTAGTCAAACTGACTG-
ACTGACCTGCAATG-
TGATTGATTAAAGAACGAGCAGGGTTAGGAAAGCAGAGAATTAGGAGCGACCGAGGGCTAGAGTATTGAATGG
CGAGAGGTGAAATTTGATGACCCATTCAGGAGTGACAAAGGCGAAGGCACTAGTCAAGGGCGAATCCGATGAT
CAAGGACGTAGGCTAGAGTTTCGAAAACGATTAGAGACCGGAGTAGTTCTAGCAGTAAACTATGCCGACGCCG
TGATATTGTTTTTTGTGG-CAGTATTGCGGAAGAGAAATC--AAGT-AAGGCTTT-
GGGGAGAGTACGCGCGCAAGCGATAAATT
>DBP2
CCAGGACCAAGGTCAGCAGCAGGCGCGAAAATTATCGAAGCCCGCC-TAGGGGCGATAGTGAGGAGACGTG-
TAT-
TCGAAGTACGTGTAAAGAACGTACTAATAACTGGAGGTCAAGTCTGGTGCCAGCATCCGCGGTAATTCCAGCT
CCAGGGGTGTCTATGATGATTGCTGCGATTAAAAAGTCCGTAGTCAAGCTGACTG-ACTGACCTGCAATG-
TGATTGATTAAAGAACGAGCAGGGTTAGGAAAGCAGAGAATTAGGAGCGACCGAGGGCTAGAGTATTGAATGG
CGAGAGGTGAAATTTGATGACCCATTCAGGAGTGACAAAGGCGAAGGCACTAGTCAAGGGCGAATCCGATGAT
CAAGGACGTAGGCTAGAGTTTCGAAAACGATTAGAGACCGGAGTAGTTCTAGCAGTAAACTATGCCGACGCCG
TGATATTGTTTTTTGTGG-CGGTATTGCGGAAGAGAAATC--AAGT-AAGGCTTT-
GGGGAGAGTACGCGCGCAAGCGATAAATT
>PrOv1

CCAGGACCAAGGTCAGCAGCAGGCGCGAAAATTATCGAAGCCCGCC-TAGGGGCGATAGTGAGGAGACGTG-
TAT-
TCGAAGTACGTGTAAAGAACGTACTAATAACTGGAGGTCAAGTCTGGTGCCAGCATCCGCGGTAATTCCAGCT
CCAGGGGTGTCTATGATGATTGCTGCGATTAAAAAGTCCGTAGTCAAGCTGACTG-ACTGACCTGCAATG-
TGATTGATTAAAGAACGAGCAGGGTTAGGAAAGCAGAGAATTAGGAGCGACCGAGGGCTAGAGTATTGAATGG
CGAGAGGTGAAATTTGATGACCCATTCAGGAGTGACAAAGGCGAAGGCACTAGTCAAGGGCGAATCCGATGAT
CAAGGACGTAGGCTAGAGTTTCGAAAACGATTAGAGACCGGAGTAGTTCTAGCAGTAAACTATGCCGACGCCG
TGATATTGTTTTTTGTGG-CGGTATTGCGGAAGAGAAATC--AAGT-AAAGCTTT-
GGGGAGAGTACGCGCGCAAGCGATAAATT
>HQ681056_Dsp38JEI-2011
CCAGGACCAAGGTCAGCAGCAGGCGCGAAAATTATCGAAGCCCGCC-TAGGGGCGATAGTGAGGAGACGTG-
TAT-
TCGAAGTACGTGTAAAGAACGTACTAATAACTGGAGGTCAAGTCTGGTGCCAGCATCCGCGGTAATTCCAGCT
CCAGGGGTGTCTATGATGATTGCTGCGATTAAAAAGTCCGTAGTCAAGCTGACTG-ACTGACCTGCAATG-
TGATTGATTAAAGAACGAGCAGGGTTAGGAAAGCAGAGAATTAGGAGCGACCGAGGGCTAGAGTATTGAATGG
CGAGAGGTGAAATTTGATGACCCATTCAGGAGTGACAAAGGCGAAGGCACTAGTCAAGGGCGAATCCGATGAT
CAAGGACGTAGGCTAGAGTTTCGAAAACGATTAGAAACCGGAGTAGTTCTAGCAGTAAACTATGCCGACGCCG
TGATATTGTTTTTTGTGG-CGGTATTGCGGAAGAGAAATC--AAGT-AGGGCTTT-
GGGGAGAGTACGCGCGCAAGCGATAAATT
>HQ683742_Dsp33
CCAGGACCAAGGTCAGCACCAGGCGCGAAAATTATCGAAGCCCGCC-TAGGGGCGATAGTGAGGAGACGTG-
TATAACGAAGTACGTGTAAAG-
ACGTCCTAATACCTGGAGGTCAAGTCTGGTGCCAGCATCCGCGGTAATTCCAGCTCCAGGGGTGTCTATGATG
ATTGCTGCGATTAAAAAGTCCGTAGTCAAACTGACTG-ACTGACCTGCAATG-
TGATTGATTAAAGAACGAGCAGGGTTAGGAAAGCAGAGAATTAGGAGCGACCGAGGGCTAGAGTATTGAATGG
CGAGAGGTGAAATTTGATGACCCATTCAGGAGTGACAAAGGCGAAGGCACTAGTCAAGGGCGAATCCGATGAT
CAAGGACGTAGGCTAGAGTTTCGAAAACGATTAGAGACCGGAGTAGTTCTAGCAGTAAACTATGCCGACGCCG
TGATATTGTTTTTTGTGG-CAGTATTGCGGAAGAGAAATC--AAGT-AAGGCTTT-
GGGGAGAGTACGCGCGCAAGCGATAAATT
>JQ673482_Ddue
CCAGGACCAAGGTCAGCAGCAGGCGCGAAAATTATCGAACCCCGCC-TAGGGGCGATAGGGAGGAAACGTG-
TATAACGAAGTACGTGTAAAGAACGTGCTAATAACTGGAGGTCAAGTCTGGTGCCAGCATCCGCGGTAATTCC
AGCTCCAGGGGTGTCTATGATGATTGCTGCAATTAAAAAGTCCGAAGTCAATCTGACTG-
ACTGACCTGCAATG-
TGATTGATTAAAGAACGAGCAGGGTTAGGAAAGCAAAGAATTAGGAGCGACCGAGGGCTAGAGTATTGAATGG
CGAGAGGTGAAATTTGATGACCCATTCAGGAGTGACAAAGGCGAAGGCACTAGTCAAGGGCGAATCCAATGAT
CAAGGACGTAGGCTAGAGTTTCGAAAACAATTAGAGACCGGAGTAGTTCTAGCATTAAACTATGCCGACGCCG
TGGTATCA------ACAT-TGGTATTGCGGAAGAGAAATC--AAGT-AAGGCTTT-
GGGGAGAGTACGCGCGCAAGCGATAAATT
>M60_Gbal
CCAGGACCAAGGTCAGCAGCAGGCGCGAAAATTATCGAAGCCCGCC-TAGGGGCGATAGTGAGGAGACGTG-
TAT-
ACGAAGTACGTGTAAAGACCGTACTAATAACTGGAGGTCAAGTCTGGTGCCAGCATCCGCGGTAATTCCAGCT
CCAGGGGTGTCTATGATGATTGCTGCGATTAAAAAGTCCGTAGTCGAGCTGACTG-ACTGACCTGCAATG-
TGATTGATTAAAGAACGAGCAGGGTTAGGAAAGCAGAGGATTAGGAGCGACCGAGGGCTAGAGTATTGAATGG
CGAGAGGTGAAATTTGATGACCCATTCAGGAGTGACAAAGGCGAAGGCACTAGTCAAGGGCGAATCCGATGAT
CAAGGACGTAGGCTAGAGTTTCGAAAACGATTAGAGACCGGAGTAGTTCTAGCAGTAAACTATGCCGACGCCG
TGGTATAG------GCTA-TAGTATTGCGGAAGAGAAATC--AAGT-AAGGCTTT-
GGGGAGAGTACGCGCGCAAGCGATAAATT
>GbOv2
CCAGGACCAAGGTCAGCAGCAGGCGCGAAAATTATCGAAGCCCGCC-TAGGGGCGATAGTGAGGAGACGTG-
TAT-
ACGAAGTACGTGTAAAGACCGTACTAATAACTGGAGGTCAAGTCTGGTGCCAGCATCCGCGGTAATTCCAGCT
CCAGGGGTGTCTATGATGATTGCTGCGATTAAAAAGTCCGTAGTCGAGCTGACTG-ACTGACCTGCAATG-
TGATTGATTAAAGAACGAGCAGGGTTAGGAAAGCAGAGGATTAGGAGCGACCGAGGGCTAGAGTATTGAATGG
CGAGAGGTGAAATTTGATGACCCATTCAGGAGTGACAAAGGCGAAGGCACTAGTCAAGGGCGAATCCGATGAT
CAAGGACGTAGGCTAGAGTTTCGAAAACGATTAGAGACCGGAGTAGTTCTAGCAGTAAACTATGCCGACACCG

TGGTATTA------ATTT-TAGTATTGCGGAAGAGAAATC--AAGT-AAGGCTTT-
GGGGAGAGTACGCGCGCAAGCGATAAATT
>GFOS_GfOv
CCAGGACCAAGGTCAGCAGCAGGCGCGAAAATTATCGAAGCCCGCC-TAGGGGCGATAGTGAGGAGACGTG-
TAT-
ACGAAGTACGTGTAAAGACCGTACTAATAACTGGAGGTCAAGTCTGGTGCCAGCATCCGCGGTAATTCCAGCT
CCAGGGGTGTCTATGATGATTGCTGCGATTAAAAAGTCCGTAGTCGAGCTGACTG-ACTGACCTGCAATG-
TGATTGATTAAAGAACGAGCAGGGTTAGGAAAGCAGAGAATTAGGAGCGACCGAGGGCTAGAGTATTGAATGG
CGAGAGGTGAAATTTGATGACCCATTCAGGAGTGACAAAGGCGAAGGCACTAGTCAAGGGCGAATCCGATGAT
CAAGGACGTAGGCTAGAGTTTCGAAAACGATTAGAGACCGGAGTAGTTCTAGCAGTAAACTATGCCGACGCCG
TGGTATTT--------TT-TAGTATTGCGGAAGAGAAATC--AAGT-AAGGCTTT-
GGGGAGAGTACGCGCGCAAGCGATAAATT
>GU196258_DspGF
CCAGGACCAAGGTCAGCAGCAGGCGCGAAAATTATCGAAGCCCGCC-TAGGGGCGATAGTGAGGAGACGTG-
TAT-
ACGAAGTACGTGTAAAGACCGTACTAATAACTGGAGGTCAAGTCTGGTGCCAGCATCCGCGGTAATTCCAGCT
CCAGGGGTGTCTATGATGATTGCTGCGATTAAAAAGTCCGTAGTCGAGCTGACTG-ACTGACCTGCAATG-
TGATTGATTAAAGAACGAGCAGGGTTAGGAAAGCAGAGGATTAGGAGCGACCGAGGGCTAGAGTATTGAATGG
CGAGAGGTGAAATTTGATGACCCATTCAGGAGTGACAAAGGCGAAGGCACTAGTCAAGGGCGAATCCGATGAT
CAAGGACGTAGGCTAGAGTTTCGAAAACGATTAGAGACCGGAGTAGTTCTAGCAGTAAACTATGCCGACGCCG
TGGTATTA--------TT-TAGTATTGCGGAAGAGAAATC--AAGT-AAGGCTTT-
GGGGAGAGTACGCGCGCAAGCGATAAATT
>GU196256_DspGL
CCAGGACCAAGGTCAGCAGCAGGCGCGAAAATTATCGAAGCCCGCC-TAGGGGCGATAGTGAGGAGACGTG-
TAT-
ACGAAGTACGTGTAAAGACCGTACTAATAACTGGAGGTCAAGTCTGGTGCCAGCATCCGCGGTAATTCCAGCT
CCAGGGGTGTCTATGATGATTGCTGCGATTAAAAAGTCCGTAGTCGAGCTGACTG-ACTGACCTGCAATG-
TGATTGATTAAAGAACGAGCAGGGTTAGGAAAGCAGAGAATTAGGAGCGACCGAGGGCTAGAGTATTGAATGG
CGAGAGGTGAAATTTGATGACCCATTCAGGAGTGACAAAGGCGAAGGCACTAGTCAAGGGCGAATCCGATGAT
CAAGGACGTAGGCTAGAGTTTCGAAAACGATTAGAGACCGGAGTAGTTCTAGCAGTAAACTATGCCGACACCG
TGGTATTAGTTTT-TAAA-TAGTATTGCGGAAGAGAAATC--AAGT-AAGGCTTT-
GGGGAGAGTACGCGCGCAAGCGATAAATT
>M41_Gvar
CCAGGACCAAGGTCAGCAGCAGGCGCGAAAATTATCGAAGCCCGCC-TAGGGGCGATAGTGAGGAGACGTG-
TTA-
ACGAAGTACGTGTAAAGACCGTACTAATAACTGGAGGTCAAGTCTGGTGCCAGCATCCGCGGTAATTCCAGCT
CCAGGGGTGTCTATGATGATTGCTGCGATTAAAAAGTCCGTAGTCGAGCTGACTG-ACTGACCTGCAATG-
TGATTGATTAAAGAACGAGCAGGGTTAGGAAAGCAGAGGATTAGGAGCGACCGAGGGCTAGAGTATTGAATGG
CGAGAGGTGAAATTTGATGACCCATTCAGGAGTGACAAAGGCGAAGGCACTAGTCAAGGGCGAATCCGATGAT
CAAGGACGTAGGCTAGAGTTTCGAAAACGATTAGAGACCGGAGTAGTTCTAGCAGTAAACTATGCCGACGCCG
TGGTATTAG-----GTAT-TAGTATTGCGGAAGAGAAATC--AAGT-AAGGCTTT-
GGGGAGAGTACGCGCGCAAGCGATAAATT
>HQ681050_Dsp23JEI-2011
CCAGGACCAAGGTCAGCAGCAGGCGCGAAAATTATCGAAGCCCGCC-TAGGGGCGATAGTGAGGAGACGTG-
TAT-
ACGAAGTACGTGTAAAGACCGTACTAATAACTGGAGGTCAAGTCTGGTGCCAGCATCCGCGGTAATTCCAGCT
CCAGGGGTGTCTATGATGATTGCTGCGATTAAAAAGTCCGTAGTCGAGCTGACTG-ACTGACCTGCAATG-
TGATTGATTAAAGAACGAGCAGGGTTAGGAAAGCAGAGGATTAGGAGCGACCGAGGGCTAGAGTATTGAATGG
CGAGAGGTGAAATTTGATGACCCATTCAGGAGTGACAAAGGCGAAGGCACTAGTCAAGGGCGAATCCGATGAT
CAAGGACGTAGGCTAGAGTTTCGAAAACGATTAGAGACCGGAGTAGTTCTAGCAGTAAACTATGCCGACGCCG
TGGTATTAA-----ATAT-TAGTATTGCGGAAGAGAAATC--AAGT-AAGGCTTT-
GGGGAGAGTACGCGCGCAAGCGATAAATT
>HQ681051_Dsp25JEI-2011
CCAGGACCAAGGTCAGCAGCAGGCGCGAAAATTATCGAAGCCCGCC-CAGGGGCGATAGTGAGGAGACGTG-
TAT-
ACGAAGTACGTGTAAAGACCGTACTAATAACTGGAGGTCAAGTCTGGTGCCAGCATCCGCGGTAATTCCAGCT
CCAGGGGTGTCTATGATGATTGCTGCGATTAAAAAGTCCGTAGTCGAGCTGACTG-ACTGACCTGCAATG-
TGATTGATTAAAAGACGAGCAGGGTTAGGAAAGCAGAGAATTAGGAGCGACCGAGGGCTAGAGTATTGAATGG

CGAGAGGTGAAATTTGATGACCCATTCAGGAGTGACAAAGGCGAAGGCACTAGTCAAGGGCGAATCCGATGAT
CAAGGACGTAGGCTAGAGTTTCGAAAACGATTAGAGACCGGAGTAGTTCTAGCAGTAAACTATGCCGACACCG
TGGTATTAA-----ATTT-TAGTATTGCGGAAGAGAAATC--AAGT-AAGGCTTT-
GGGGAGAGTACGCGCGCAAGCGATAAATT
>GVAR_M40
CCAGGACCAAGGTCAGCAGCAGGCGCGAAAATTATCGAAGCCCGCC-TAGGGGCGATAGTGAGGAGACGTG-
TTA-
ACGAAGTACGTGTAAAGACCGTACTAATAACTGGAGGTCAAGTCTGGTGCCAGCATCCGCGGTAATTCCAGCT
CCAGGGGTGTCTATGATGATTGCTGCGATTAAAAAGTCCGTAGTCGAGCTGACTG-ACTGACCTGCAATG-
TGATTGATTAAAGAACGAGCAGGGTTAGGAAAGCAGAGGATTAGGAGCGACCGAGGGCTAGAGTATTGAATGG
CGAGAGGTGAAATTTGATGACCCATTCAGGAGTGACAAAGGCGAAGGCACTAGTCAAGGGCGAATCCGATGAT
CAAGGACGTAGGCTAGAGTTTCGAAAACGATTAGAGACCGGAGTAGTTCTAGCAGTAAACTATGCCGACGCCG
TGGTATTAG-----GTAT-TAGTATTGCGGAAGAGAAATC--AAGTAAAGGCTTT-
GGGGAGAGTACGCGCGCAAGCGATAAATT
>KT956061_DspGLS-1
CCAGGACCAAGGTCAGCAGCAGGCGCGAAAATTATCGAAGCCCGCC-TAGGGGCGATAGTGAGGAGACGTG-
TAT-
ACGAAGTACGTGTAAAGACCGTACTAATAACTGGAGGTCAAGTCTGGTGCCAGCATCCGCGGTAATTCCAGCT
CCAGGGGTGTCTATGATGATTGCTGCGATTAAAAAGTCCGTAGTCGAGCTGACTG-ACTGACCTGCAATG-
TGATTGATTAAAGAACGAGCAGGGTTAGGAAAGCAGAGGATTAGGAGCGACCGAGGGCTAGAGTATTGAATGG
CGAGAGGTGAAATTTGATGACCCATTCAGGAGTGACAAAGGCGAAGGCACTAGTCAAGGGCGAATCCGATGAT
CAAGGACGTAGGCTAGAGTTTCGAAAACGATTAGAGACCGGAGTAGTTCTAGCAGTAAACTATGCCGACGCCG
TGGTATTAA-----ATTT-TAGTATTGCGGAAGAGAAATC--AAGT-
AAGGCTTTGGGGGAGAGTACGCGCGCAAGCGATAAATT
>KT956062_DspGLS-2
CCAGGACCAAGGTCAGCAGCAGGCGCGAAAATTATCGAAGCCCGCC-TAGGGGCGATAGTGAGGAGACGTG-
TAT-
ACGAAGTACGTGTAAAGACCGTACTAATAACTGGAGGTCAAGTCTGGTGCCAGCATCCGCGGTAATTCCAGCT
CCAGGGGTGTCTATGATGATTGCTGCGATTAAAAAGTCCGTAGTCGAGCTGACTG-ACTGACCTGCAATG-
TGATTGATTAAAGAACGAGCAGGGTTAGGAAAGCAGAG-
ATTAGGAGCGACCGAGGGCTAGAGTATTGAATGGCGAGAGGTGAAATTTGATGACCCATTCAGGAGTGACAAA
GGCGAAGGCACTAGTCAAGGGCGAATCCGATGATCAAGGACGTAGGCTAGAGTTTCGAAAACGATTAGAGACC
GGAGTAGTTCTAGCAGTAAACTATGCCGACGCCGTGGTATTAA-----ATTT-
TAGTATTGCGGAAGAGAAATC--AAGT-AAGGCTTTGGGGGAGAGTACGCGCGCAAGCGATAAATT
>GRR24
CCAGGACCAAGGTCAGCAGCAGGCGCGAAAATTATCGAAGCCCGCC-TAGGGGCGATAGTGAGGAGACGTG-
TAT-
ACGAAGTACGTGTAAAGACCGTACTAATAACTGGAGGTCAAGTCTGGTGCCAGCATCCGCGGTAATTCCAGCT
CCAGGGGTGTCTATGATGATTGCTGCGATTAAAAAGTCCGTAGTCGAGCTGACTG-ACTGACCTGCAATG-
TGGTTGATTAAAAGACGAGCAGGGTTAGGAAAGCAGAGAATTAGGAGCGACCGAGGGCTAGAGTATTGAATGG
CGAGAGGTGAAATTTGATGACCCATTCAGGAGTGACAAAGGCGAAGGCACTAGTCAAGGGCGAATCCGATGAT
CAAGGACGTAGGCTAGAGTTTCGAAAACGATTAGAGACCGGAGTAGTTCTAGCAGTAAACTATGCCGACACCG
TGGTATTAAT----TTTT-TAGTATTGCGGAAGAGAAATC--AAGT-AAGGCTTT-
GGGGAGAGTACGCGCGCAAGCGATAAATT
>GU196257_DspGL2
CCAGGACCAAGGTCAGCAGCAGGCGCGAAAATTATCGAAGCCCGCC-GAGGGGCGATAGTGAGGAGACGTG-
TATAACGAAGTGCGTGTAAAGAACGTGCTAATAACTGGAGGTCAAGTCTGGGGCCAGCATCCGCGGTAATTCC
AGCTCCAGGGGGGTCTATGATGATTGCTGCGATTAAAAAGTCCGTAGTCAATCTGACTG-
ACTGGCCTGCAATG-
TGATTGATTAAAGAACGAGCAGGGCTAGGAAAGCAGAAAATTAGGAGCGACCGAGGGCTAGAGTATTGAATGG
CGAGAGGTGAAATTTGATGACCCATTCAGGAGTGACAAAGGCGAAGGCACTAGTCAAGGGCGAATCCGATGAT
CAAGGACGTAGGCTAGAGTTTCGAAAACGATTAGAGACCGGAGTAGTTCTAGCAGTAAACTATGCCGACGCCG
TGGTATCTAATTT-TAAC-AGGTATTGCGGAAGAGAAATC--AAGT-AAGGCTTT-
GGGGAGAGTACGCGCGCAAGCGATAAATT
>HQ681053_Dsp28JEI-2011
CCAGGACCAAGGTCAGCAGCAGGCGCGAAAATTATCGAAGCCCGCC-GAGGGGCGATAGTGAGGAGACGTG-
TATAACGAAGTGCGTGTAAAGAACGTGCTAATAACTGGAGGTCAAGTCTGGTGCCAGCATCCGCGGTAATTCC
AGCTCCAGGGGTGTCTATGATGATTGCTGCGATTAAAAAGTCCGTAGTCAATCTGACTG-

ACTGGCCTGCAATG-
TGATTGATTAAAGAACGAGCAGGGCTAGGAAAGCAGAGAATTAGGAGCGACCGAGGGCTAGAGTATTGAATGG
CGAGAGGTGAAATTTGATGACCCATTCAGGAGTGACAAAGGCGAAGGCCCTAGTCAAGGGCGAATCCGATGAT
CAAGGACGTAGGCTAGAGTTTTGAAAACGATTAGAGACCGGAGTAGTTCTAGCAGTAAACTATGCCGACGCCG
TGGTATCTAATTT-TAAC-AGGTATTGCGGAAGAGAAATC--AAGT-AAGGCTTT-
GGGGAGAGTACGCGCGCAAGCGATAAATT
>UA029A-7
CCAGGACCAAGGTCAGCAGCAGGCGCGAAAATTATCGAAGCCCGCG-TAGGGGCGATAGTGAGGAGACGTG-
TATTACGAAGTGTGTGTAAAGAACGCACTAATAACTGGAGGTCAAGTCTGGTGCCAGCATCCGCGGTAATTCC
AGCTCCAGGGGTGTCTATGATGATTGCTGCGATTAAAAAGTCCGTAGTCAAGCTGACTG-
ACTTGCCTGCAATG-
TGACTGATTAAGAGACGAGCAGGGCTAGGAAAGCAGAGAATTAGGAGCGACCGAGGGCTAGAGTATTGAATGG
CGAGAGGTGAAATTTGATGACCCATTCAGGAGTGACAAAGGCGAAGGCACTAGTCAAGGGCGAATCCGATGAT
CAAGGACGTAGGCTAGAGTTTCGAAAACGATTAGAGACCGGAGTAGTTCTAGCAGTAAACTATGCCGACGCCG
TGGTATGG------TATT-CTGTATTGCGGAAGAGAAATC--AAGT-AAGGCTTT-
GGGGAGAGTACGCGCGCAAGCGATAAATT
>DHAE_DOP13
CCAGGACCAAGGTCAGCAGCAGGCGCGAAAATTATCGAAGCCCGCG-TAGGGGCGATAGTGAGGAGACGTG-
TATTACGAAGTGTGTGTAAAGAACGCACTAATAACTGGAGGTCAAGTCTGGTGCCAGCATCCGCGGTAATTCC
AGCTCCAGGGGTGTCTATGATGATTGCTGCGATTAAAAAGTCCGTAGTCAAGCTGACTG-
ACTTGCCTGCAATG-
TGACTGATTAAGAGACGAGCAGGGCTAGGAAAGCAGAGAATTAGGAGCGACCGAGGGCTAGAGTATTGAATGG
CGAGAGGTGAAATTTGATGACCCATTCAGGAGTGACAAAGGCGAAGGCACTAGTCAAGGGCGAATCCGATGAT
CAAGGACGTAGGCTAGAGTTTCGAAAACGATTAGAGACCGGAGTAGTTCTAGCAGTAAACTATGCCGACGCCG
TGATATGG------TATT-CTGTATTGCGGAAGAGAAATC--AAGT-AAGGCTTT-
GGGGAGAGTACGCGCGCAAGCGATAAATT
>AJ438957_Dber37
CCAGGACCAAGGTCAGCAGCAGGCGCGAAAATTATCGAAGCCCGCA-TAGGGGCGATAGTGAGGAGACGTG-
TATTACGAAGTGTGTGTAAAGAACGCACTAATAACTGGAGGTCAAGTCTGGTGCCAGCATCCGCGGTAATTCC
AGCTCCAGGGGTGTCTATGATGATTGCTGCGATTAAAAAGTCCGTAGTCAAGCTGACTG-
ACTTGCCTGCAATG-
TGACTGATTAAGAGACGAGCAGGGCTAGGAAAGCAGAGAATTAGGAGCGACCGAGGGCTAGAGTATTGAATGG
CGAGAGGTGAAATTTGATGACCCATTCAGGAGTGACAAAGGCGAAGGCACTAGTCAAGGGCGAATCCGATGAT
CAAGGACGTAGGCTAGAGTTTCGAAAACGATTAGAGACCGGAGTAGTTCTAGCAGTAAACTATGCCGACGCCG
TGGTATGG------TATT-CTGTATTGCGGAAGAGAAATC--AAGT-AAGGCTTT-
GGGGAGAGTACGCGCGCAAGCGATAAATT
>HQ681059_Dsp42JEI-2011
CCAGGACCAAGGTCAGCAGCAGGCGCGAAAATTATCGAAGCCCGCG-TAGGGGCGATAGTGAGGAGACGTG-
TATTACGAAGTGTGTGTAAAGAACGCACTAATAACTGGAGGTCAAGTCTGGTGCCAGCATCCGCGGTAATTCC
AGCTCCAGGGGTGTCTATGATGATTGCTGCGATTAAAAAGTCCGTAGTCAAGCTGACTG-
ACTTGCCTGCAATG-
TGAGTGATTAAGAGACGAGCAGGGGTAGGAAAGCAGAGAATTAGGAGCGACCGAGGGCTAGAGTATTGAATGG
CGAGAGGTGAAATTTGATGACCCATTCAGGAGTGACAAAGGCGAAGGCACTAGTCAAGGGCGAATCCGATGAT
CAAGGACGTAGGCTAGAGTTTCGAAAACGATTAGAGACCGGAGTAGTTCTAGCAGTAAACTATGCCGACGCCG
TGGTGTGG------TATT-CTGTATTGCGGAAGAGAAATC--AAGT-AAGGCTTT-
GGGGAGAGTACGCGCGCAAGCGATAAATT
>DHAE_DML3
CCAGGACCAAGGTCAGCAGCAGGCGCGAAAATTATCGAAGCCCGCG-TAGGGGCGATAGTGAGGAGACGTG-
TATTACGAAGTGTGTGTAAAGAACGCACTAATAACTGGAGGTCAAGTCTGGTGCCAGCATCCGCGGTAATTCC
AGCTCCAGGGGTGTCTATGATGATTGCTGCGATTAAAAAGTCCGTA-TCAAGCTGACTG-
ACTTGCCTGCAATG-
TGACTGATTAAGAGACGAGCAGGGCTAGGAAAGCAGAGAATTAGGAGCGACCGAGGGCTAGAGTATTGAATGG
CGAGAGGTGAAATTTGATGACCCATTCAGGAGTGACAAAGGCGAAGGCACTAGTCAAGGGCGAATCCGATGAT
CAAGGACGTAGGCTAGAGTTTCGAAAACGATTAGAGACCGGAGTAGTTCTAGCAGTAAACTATGCCGACGCCG
TGGTATGG------TATT-CTGTATTGCGGAAGAGAAATC--AAGT-AAGGCTTT-
GGGGAGAGTACGCGCGCAAGCGATAAATT
>JQ673481_Dber

CCAGGACCAAGGTCAGCAGCAGGCGCGAAAATTATCGAAGCCCGCA-TAGGGGCGATAGTGAGGAGACGTG-
TATTACGAAGTGTGTGTAAAGAACGCACTAATAACTGGAGGTCAAGTCTGGTGCCAGCATCCGCGGTAATTCC
AGCTCCAGGGGTGTCTATGATGATTGCTGCGATTAAAAAGTCCGTAGTCAAGCTGACTG-
ACTTGCCTGCAATG-
TGACTGATTAAGAGACGAGCAGGGCTAGGAAAGCAGAGAATTAGGAGCGACCGAGGGCTAGAGTATTGAATGG
CGAGAGGTGAAATTTGATGACCCATTCAGGAGTGACAAAGGCGAAGGCACTAGTCAAGGGCGAATCCGATGAT
CAAGGACGTAGGCTAGAGTTTCGAAAACGATTAGAGACCGGAGTAGTTCTAGCAGTAAACTATGCCGACGCCG
TGGTATGGG-----TTTT-CTGTATTGCGGAAGAGAAATC--AAGT-AAGGCTTT-
GGGGAGAGTACGCGCGCAAGCGATAAATT
>HQ683745_Dsp32
CCCAGGCCCAGGTTCACCACCGGCGCGAAAATTATTGAAACCCCCC-TAGGGGCGATAGGGAGGAGACGGG-
TATTTCGAAGTACGTGTAAAGAACGTCCTAATAACTGGAGGTCAAGTCTGGTGCCAGCATCCGCGGTAATTCC
AGCTCCAGGGGTGTCTATGATGATTGCTGCGATTAAAAAGTCCGTAGTCAAACTGACTG-
ACTGACCTGCAATG-
TGATTGATTAAAGAACGAGCAGGGTTAGGAAAGCAGAGAATTAGGAGCGACCGAGGGCTAGAGTATTGAATGG
CGAGAGGTGAAATTTGATGACCCATTCAGGAGTGACAAAGGCGAAGGCACTAGTCAAGGGCGAATCCGATGAT
CAAGGACGTAGGCTAGAGTTTCGAAAACGATTAGAGACCGGAGTAGTTCTAGCAGTAAACTATGCCGACGCCG
TGATATTGTTTTTTGTGG-CAGTATTGCGGAAGAGAAATC--AAGT-AAGGCTTT-
GGGGAGAGTACGCGCGCAAGCGATAAATT
>KT956057_DspEVL-9
CCAGGACCAAGGTCAGCAGCAGGCGCGAAAATTATCGAAGCCCGCC-TAGGGGCGATAGTGAGGAGACGTG-
TATAACGAAGTACGTGTAAAGAACGTACTAATAACTGGAGGTCAAGTCTGGTGCCAGCATCCGCGGTAATTCC
AGCTCCAGGGGTGTCTATGATGATTGCTGCGATTAAAAAGTCCGTAGTCAAGCTGACTG-
ACTGACCTGCAATG-
TGATTGATCAAAGAACGAGCAGGGTTAGGAAAGCAGAGAATTAGGAGCGACCGAGGGCTAGAGTATTGAATGG
CGAGAGGTGAAATTTGATGACCCATTCAGGAGTGACAAAGGCGAAGGCACTAGTCAAGGGCGAATCCGATGAT
CACGGACGTAGGCTAGAGTTTCGAAAACGATTAGAGACCGGAGTAGTTCTAGCAGTAAACTATGCCGACGCCG
TGATATTGTTTTTTGTGG-CAGTATTGCGGATGAGAATCA--AGTT-
AAGGCTTTGGGGGAGAGTACGCGCGT-AGCGATAAATT
>HQ681060_Dsp43JEI-2011
CCAGGACCAAGGTCAGCAGCAGGCGCGAAAATTATCGAAGCCCGCC-TAGGGGCGATAGTGAGGAGACGTG-
TATAACGAAGTACGTGTAAAGAACGTGCTAATAACTGGAGGTCAAGTCTGGTGCCAGCATCCGCGGTAATTCC
AGCTCCAGGGGTGTCTATGATGATTGCTGCGATTAAAAAGTCCGTAGTCAATCTGACTG-
ACTGACCTGCAATG-
TGATTGATTAAAGAACGAGCAGGGTTAGGAAAGCAGAGAATTAGGAGCGACCGAGGGCTAGAGTATTGAATGG
CGAGAGGTGAAATTTGATGACCCATTCAGGAGTGACAAAGGCGAAGGCACTAGTCAAGGGCGAATCCGATGAT
CAAGGACGTAGGCTAGAGTTTCGAAAACGATTAAAAGCCGGAGGAATTTTAACCATTAAATAATCCGGCCCCG
GGAAAAAGTTTTTTGTGG-TTGTATTGCGGAAGAGAAATC--AAGT-AAGGCTTT-
GGGGAGAGTACGCGCGCAAGCGATAAATT
>KF537632_Ddiporeiae
CCAGGACCAAGGTCAGCAGCAGGCGCGAAAATTATCGAAGCCCACCATTGGGGCGATAGTGAGGAGACGTG-
TATAACGAAATACGGGTAAAGAACGTATGTATAACTGGAGGTCAAGTCTGGTGCCAGCATCCGCGGTAATTCC
AGCTCCAGGGGTGTCTATGATGATTGCTGCGATTAAAAAGTCCGTAGTCAAGCTGACTG-
ACTAACCTGTAATG-
TGGTTGATTAAAAGACGAGAAGGGTTAGGAAAGCAGAGGATTAGGAGCGACCGAGGGCTAGAGTATTGAATGG
CGAGAGGTGAAATTTGATGACCCATTCAGGAGTGACAAAGGCGAAGGCACTAGTCAAGGGCGAATCCGATGAT
CAAGGACGTAGGCTAGAGGTTCGAAAACGATTAGAGACCGGAGTAGTTCTAGCAGTAAACTATGCCGACGCCG
TGGTATGTTTTTT-----AATGTATTGCGGAAGAGAAATC--AAGT-AAGGCTTT-
GGGGAGAGTACGCGCGCAAGCGATAAATT
>AJ966726_DspHYAL
CCAGGACCAAGGTCAGCAGCAGGCGCGAAAATTATCGAAGCCCGCA-TTGGGGCGATAGTGAGGAGACGTG-
AAT-
TTTAGGTGCGGGTAAAAAACGCACTAGTAACTGGAGGTCAAGTCTGGTGCCAGCATCCGCGGTAATTCCAGCT
CCAGGGGTGTCTATGATGATTGCTGCGATTAAAAAGTCCGTAGTCAAGCCGCCAG-
ACCAGTCTGGAATGTTTCTTGATTAAGAGATGAATAGGGCTGGGAAAGCGGAGAATTAGGAGCGACCGAGGGC
TAGAGTATTGGGTGGCGAGAGGTGAAATTTGATGACCCATCCAGGAGTGACAAAGGCGAAGGCACTAGTCAAG
GGCGAATCCGATGATCAAGGACGTAGGCTAGAGTTTCGAAAACGATTAGAGACCGGAGTAGTTCTAGCAGTAA

ACTATGCCGACGCCGTGATATGAG-----GATA-TTGTATTGCGGAAGAGAAATC--AAGT-AAGGCTTT-
GGGGAGAGTACGCGCGCAAGCGATAAATT
>AJ438960_Dcavimanum4
CCAGGACCAAGGTCAGCAGCAGGCGCGAAAATTATCGAAGCCCGCC-TAGGGGCGATAGTGAGGAGACGTG-
AAT-
TTTAGGTGCGTGTAAAGAACGCACTAGCAACTGGAGGTCAAGTCTGGTGCCAGCATCCGCGGTAATTCCAGCT
CCAGGGGTGTCTATGATGATTGCTGCGATTAAAAAGTCCGTAGTCAAGCCGCCAG-
ACCAGTCTGGAATGTTTCTTGATCAAGAGACGAGCAGGGCTGGGAAAGCGGAGAATTAGGAGCGACCGAGGGC
TAGAGTATTGGGTGGCGAGAGGTGAAATTTGATGACCCATCCAGGAGTGACAAAGGCGAAGGCACTAGTCAAG
GGCGAATCCGATGATCAAGGACGTAGGCTAGAGTTTCGAAAACGATTAGAGACCGGAGTAGTTCTAGCAGTAA
ACTATGCCGACGCCGTGATATGAT-----TTTG-TTGTATTGCGGAAGAGAAATC--AAGT-AAGGCTTT-
GGGGAGAGTACGCGCGCAAGCGATAAATT
>AJ438961_DdeshayesumT10
CCAGGACCAAGGTCAGCAGCAGGCGCGAAAATTATCGAAGCCCGCC-TAGGGGCGATAGTGAGGAGACGTG-
AAT-
TGTAGGTGCGGGTAAAAAACGCACTAGCAACTGGAGGTCAAGTCTGGTGCCAGCATCCGCGGTAATTCCAGCT
CCAGGGGTGTCTATGATGATTGCTGCGATTAAAAAGTCCGTAGTCAAGCCGCCAG-ACCAGTCTGGAATG-
TTTCTGATCAAGGGACGAGCAGGGCTGGGAAAGCGGAGAATTAGGAGCGACCGAGGGCTAGAGTATTGGGTGG
CGAGAGGTGAAATTTGATGACCCATCCAGGAGTGACAAAGGCGAAGGCACTAGTCAAGGGCGAATCCGATGAT
CAAGGACGTAGGCTAGAGGTTCGAAAACGATTAGAGACCGGAGTAGTTCTAGCAGTAAACTATGCCGACGCCG
CGATATGAG-----GTTT-TTGTATTGCGGAAGAGAAATC--AAGT-AAGGCTTT-
GGGGAGAGTACGCGCGCAAGCGATAAATT
>KX364285_Unikaryon legeri
CCAGGACCAAGGTCAGCAGCAGGTGCGAAACTTATCGAAGCCCGCA-TTGGGGCGATAGTGAGGAGACGTG--
ATAACTATGGACGTGTAAAAACCATTCTAATAACTGGAGGCCAAGTCTGGTGCCAGCAGTCGCGGTAATTCCA
GCTCCAGTAGTGTCTATGATGATTGCTGCGATTAAAAAGTCCGTAGTCTATCTGACTG-
ACTTGCCTGTAATG-
TGATTGATTAAAAGACGAGTAGGGCTAGGAAAGCAGAGAATTAGGAGCGACCAAGGGCTAGAGTATTGAATGG
CGAGGGGTGAAATCTGATGACCCATTCAGGAGTGACAGAGGCGAAAGCGCTAGTCAGGGGCGAATCCGATGAT
CAAGGACGTAGGCTAGAGATTCGAAAACGATTAGAGACCGGAGTAGTTCTAGCAGTAAACTATGCCGACGCCG
TGGTATTTT-----TGTA-TAGTGCTGCGGAAGAGAAATC--AAGT-AAGGCTTT-
GGGGAGAGTACGCGCGCAAGCGATAAATT
>FJ756211_Dgammarellum
CCAGGACCAAGGTCAGCAGCAGGCGCGAAAATTATCGAAGCCCACT-TTGGGGCGATAGTGAGGAGACGTA-
AAT-
ATGATGTACGGGTAAAGAACGTACTATAAACTGGAGGTCAAGTCTGGTGCCAGCATCCGCGGTAATTCCAGCT
CCAGGGGTGTCTATGATGATTGCTGCGATTAAAAAGTCCGTAGTCTAGCCGCACG-ACCAGTCTGGAATG-
TTTCTGATCAAAAGAAGAGCAGGGCTGGGAAGGCGGAGAATTAGGAGCGACCAGGGGCTAGAGTATTGAATGG
CGAGAGGTGAAATTTGATGACCCATTCAGGAGTGACAAAGGCGAAGGCACTAGTCAGGGGCGAATCCGATGAT
CAAGGACGTAGGCTAGAGTATCGAAAACGATTAGAGACCGGAGTAGTTCTAGCAGTAAACTATGCCGACTTTG
TGGCAGAGGT---------ATCTGTTGCAAACGAGAAATCTTAAGT-AAGGCTTT-
GGGGAGAGTACGCGCGCAAGCGATAAATT
>AF044391_Glugea anomala
CCAGGTCCAAGGACAGCAGCAGGCGCGAAAATTACCGCAGCCTGCGTTCAGGGCGGTAGTAAGGAGACGTG--
AAAACAATGTGCGGGCAAAAAACGCACTAGATACAGGAGGACAAGACTGGTGCCAGCACCCGCGGTAATACCA
GCTCCTGGAGTGTCTATGATGATTGCTGCAGTTAAAGAGTTCGTAGTCGAAGTGGTTATAACGGTGTAACAGG
-
CCTTCTCTCAAGGAGGGTTATGCGCCGTGATTCCATGGAATAAGGAGCGTTTAGGGGCCAGGTTATTAAGCGA
CGAGGGGTGAAATCTGGTGACTCGCTTAGGAGCAACAGAGGCGAAAGCGCTGGCCAGGAGCGAATCCGATGAT
AAAGGACGTAGGCTAGAGGATCGAAGACGATTAGAGACCGTTGTAGTTCTAGCAGTAAACGATGCCGATACCG
TGGTGCGGAT---------AC-CGACG-GGAAGAGAAATC--GAGT-AGGGCCCT-
GGGGAGAGTACACGCGCAAGAGGAAATTT
>GQ203287_Glugea hertwigi
CCAGGTCCAAGGACAGCAGCAGGCGCGAAAATTACCGCAGCCTGCGTTCAGGGTGGTAGTAAGGAGACGTG--
AAAACAATGTGCGGGCAAAGACGCACTAGATACAGGAGGACAAGACTGGTGCCAGCACCCGCGGTAATACCA
GCTCCTGGAGTGTCTATGATGATTGCTGCAGTTAAAGAGTTCGTAGTCGAAGTGGTTATAACGGTGTAACAGG
-
CCTTCTCTCAAGGAGGGTTATGCGCCGTGATTCCATGGAATAAGGAGCGTTTAGGGGCCAGGTTATTAAGCGA

CGAGGGGTGAAATCTGGTGACTCGCTTAGGAGCAACAGAGGCGAAAGCGCTGGCCAGGAGCGAATCCGATGAT
AAAGGACGTAGGCTAGAGGATCGAAGACGATTAGAGACCGTTGTAGTTCTAGCAGTAAACGATGCCGATACCG
TGGTGCGGAT---------ACGCGACGCGGAAGAGAAATC--GAGT-AGGGCCCT-
GGGGAGAGTACACGCGCAAGCGAGAAATT
>Cucu_M2_UA009
CCAGGTCCAAGGACGGCAGCAGGCGCGAAAATTACCGAAGCTCGAA-
TAGAGGCGGTAGTAATGAGACGTATTAATATAAAACAAGGGTAAAAAACTTGTTAGTAACTGGAGGTCAAGTC
TGGTGCCAGCATCCGCGGTAATACCAGCTCCAGGGGTGTCTATGATGATTGCTGCGATTAAAAGGTCCGTAGT
CGAATTTATAT-AATTGTTTGTAATA-TGCTAGATAAAATAACAGAAAGAACAATTACTTTAAATGA-
AAGGAATAGTAAGGGGCTGATTAATTGAGCAACGAGAGGTGAAATTTGATGACTTGCTTAGGAGAAACAGAGG
CGAAAGCGTCAGTCAAGTATAAATCCTATGATCAAGGACGTAGGCTAGAGTATCGAACACGATTAGATACCGT
AGTAGTTCTAGCAGTAAACTATGCCTACACTATCGAATAA-----------
AAGTTTGGTAGAAGAGAAATCTTAAGT-AGGGCTTT-GGGGAGAGTACACGCGCAAGCGATAAATT

Alignment 2: SSU,ITS_partial LSU rDNA

>M32_Pr
AGC-GTAACGTGGAGCGGTGAAAGGCTCAGTAACGGGCGAGTTATTTGTTC-TCCTGGGA-------
CGGACAACACCGG-GAAACTGGTGGGAAAACGTCTAAGTTGCGG------T-
TTTTTAATCGTGGCGTAAACC------------
ATTTGGTGCAGGAGAGTAAGCTGCCATCCTATCAGTTAGTAAGTAGGGTAAGGGCCTACTTAGACGAAGACGG
GTACGGGGAATGAGGGTTTGATTCCG--
GAGAGGGAGCCTGAGAGACGGCTACCAGGACCAAGGTCAGCAGCAGGCGCGAAAATTATCGAAGCCCGCG-
TAGGGGCGATAGTGAGGAGACGTGTAT--TACGAAGTGTGTGTAAAGAACGCACTAATAACT---
GGAGGTCAAGTCTGGTGCCAGCATCCGCGGTAATTCCAGCTCCAGGGGTGTCTATGATGATTGCTGCGATTAA
AAAGTCCGTAGTCAAGCTGACTG-ACTTGCCTGCAATGTGAC-TG------
ATTAAGAGACGAGCAGGGCTAGGAAAGCAGAGAATTAGGAGCGACCGAGGGCTAGAGTATTGAATGGCGAGAG
GTGAAATTTGATGACCCAT-
TCAGGAGTGACAAAGGCGAAGGCACTAGTCAAGGGCGAATCCGATGATCAAGGACGTAGGCTAGAGTTTCGAA
AACGATT--AGAGACCGGAGTAGTTCTAGCAGTAAACTATGCCGACGCCGTGGTATG--GTATT------
CTGTATTGCGGAAGAGAAATC--AAGT-
AAGGCTTTGGGGAGAGTACGCGCGCAAGCGATAAATTTAAAGGAAATTGACGGAGGAACACCACAAGGAGTGG
AGTGTGCGGCTTAATTTGACTCAACGCGGGACAGCTTACCAGGCCCGATAATCATACGAGCGTAGTACGCGAT
AGGTTAGAGAGTGGTGCATGGCTGCTATCGACAGTTGGGGTGACC-TTAGGGTTAATTCCG-
GCAAGTAGTGAGACCTCTGCAGTTA----------TGGACAGGTATTTTT--AAGATACAGGAAGG-
AAGAGACAAGAGCA-GGTCAGTGATGCCCTTAGATGGCCTGGGCT----GCACGCGCACTACAGTGG-
TCATTATAAGTAGAAG-------TT-AGATTTTAAAGA-----------TGATCGAGAGGGA-
CTGGGCTTTG-TAAGAGGCCCAAGAACGAGGAATT-
GCTAGTAATCGTAGGCTCATTAAGATACGATGAATATGTCCCTGTACCTT---
GTACACACCGCCCGTCGTTATCGAAGATGGAATTGTGTGC-GAACGAGC--
AACAAGCGAGTGAGCGCATAGTTCTAGATGTGATAAAAG-
TCGTAACAAGGCAACTGTAGGAGAACCTGTAGTTGGATCATACAGATATAATAA----
AAAGGTAGGTTTGTTTTTAATTT-----CCCTG--------CGCAAGGG---ATCGTTTGGTTCTA--
TGTGCGATGAAGGTCGGAGCAGTATCTGATAATGTAGAATGATTGTAATAATTGTTCTATGTGACTGAATTGA
ACA-GTGATGTTCGGGTACTCCTTTGAATTAAGCATATGAGTAAAGGAAGGAAAAGAAACTAAC-
TAGGATTCTCTTATTAGTGGCGAATGAACAGAGAATAGCCC--AAGTGTAATCAATAATAAATAATT------
ATTGAGATGTCTAGTAT---ATTGAC-----
GTGAAAACAATGGAAAGTGTTGCCGTAGAGAGTTATAGCCTCGTAGCGT---
CTAAATTTAAAAAGGGAGTAGTTGTGCTCGGTAATGCACAATG--
AATAGGTGGTAGTGTCCATCTAAGGCTAAATATGACATAGAGACCGATAGTGAATAA-----
GTAGAGTGATCGAATATGGAATAGA
>DWP9
AGC-GTAACGTGGAGCGGTGAAAGGCTCAGTAACGGGCGAGTTATTTGTTC-TCCTGGGA-------
CGGACAACACCGG-GAAACTGGTGGGAAAACGTCTAAGTTGCGG------T-
TTTTTAATCGTGGCGTAAACC------------
ATTTGGTGCAGGAGAGTAAGCTGCCATCCTATCAGTTAGTAAGTAGGGTAAGGGCCTACTTAGACGAAGACGG

GTACGGGGAATGAGGGTTTGATTCCG--
GAGAGGGAGCCTGAGAGACGGCTACCAGGACCAAGGTCAGCAGCAGGCGCGAAAATTATCGAAGCCCGCG-
TAGGGGCGATAGTGAGGAGACGTGTAT--TACGAAGTGTGTGTAAAGAACGCACTAATAACT---
GGAGGTCAAGTCTGGTGCCAGCATCCGCGGTAATTCCAGCTCCAGGGGTGTCTATGATGATTGCTGCGATTAA
AAAGTCCGTAGTCAAGCTGACTG-ACTTGCCTGCAATGTGAC-TG------
ATTAAGAGACGAGCAGGGCTAGGAAAGCAGAGAATTAGGAGCGACCGAGGGCTAGAGTATTGAATGGCGAGAG
GTGAAATTTGATGACCCAT-
TCAGGAGTGACAAAGGCGAAGGCACTAGTCAAGGGCGAATCCGATGATCAAGGACGTAGGCTAGAGTTTCGAA
AACGATT--AGAGACCGGAGTAGTTCTAGCAGTAAACTATGCCGACGCCGTGGTATG--GTATT------
CTGTATTGCGGAAGAGAAATC--AAGT-
AAGGCTTTGGGGAGAGTACGCGCGCAAGCGATAAATTTAAAGGAAATTGACGGAGGAACACCACAAGGAGTGG
AGTGTGCGGCTTAATTTGACTCAACGCGGGACAGCTTACCAGGCCCGATAATCATACGAGCGTAGTACGCGAT
AGGTTAGAGAGTGGTGCATGGCTGCTATCGACAGTTGGGGTGACC-TTAGGGTTAATTCCG-
GCAAGTAGTGAGACCTCTGCAGTTA----------TGGACAGGTATTTTT--AAGATACAGGAAGG-
AAGAGACAAGAGCA-GGTCAGTGATGCCCTTAGATGGCCTGGGCT----GCACGCGCACTACAGTGG-
TCATTATAAGTAGAAG-------TT-AGATTTTAAAAA-----------TGATCGAGAGGGA-
CTGGGCTTTG-TAAGAGGCCCAAGAACGAGGAATT-
GCTAGTAATCGTAGGCTCATTAAGATACGATGAATATGTCCCTGTACCTT---
GTACACACCGCCCGTCGTTATCGAAGATGGAATTGTGTGC-GAACGAGC--
AACAAGCGAGTGAGCGCATAGTTCTAGATGTGATAAAAG-
TCGTAACAAGGCAACTGTAGGAGAACCTGTAGTTGGATCATACAGATATAATAA----
AAAGGTATGTTTGTTTTTAATTT-----CCCTG--------CGCAAGGG---ATCGTTTGGTTCTA--
TGTGCGATGAAGGTCGGAGCAGTATCTGATAATGTAGAATGATTGTAATAATTGTTCTATGTGACTGAATTGA
ACA-GTGATGTTCGGGTACTCCTTTGAATTAAGCATATGAGTAAAGGAAGGAAAAGAAACTAAC-
TAGGATTCTCTTATTAGTGGCGAATGAACAGAGAATAGCCC--AAGTGTAATCAATAATAAATAATT------
ATTGAGATGTCTAGTAT---ATTGAC-----
GTGAAAACAATGGAAAGTGTTGCCGTAGAGAGTTATAGCCTCGTAGCGT---
CTAAATTTAAAAAGGGAGTAGTTGTGCTCGGTAATGCACAATG--
AATAGGTGGTAGTGTCCATCTAAGGCTAAATATGACATAGAGACCGATAGTGAATAA-----
GTAGAGTGATCGAATATGGAATAGA
>DBP6
AGC-GTAACGTGGAGCGGTGAAAGGCTCAGTAACGGGCGAGTTATTTGTTC-TCCTGGGA-------
CGGACAACACCGG-GAAACTGGTGGGAAAACGTCTAAGTTGCGG------T-
TTTTTAATCGTGGCGTAAACC------------
ATTTGGTGCAGGAGAGTAAGCTGCCATCCTATCAGTTAGTAAGTAGGGTAAGGGCCTACTTAGACGAAGACGG
GTACGGGGAATGAGGGTTTGATTCCG--
GAGAGGGAGCCTGAGAGACGGCTACCAGGACCAAGGTCAGCAGCAGGCGCGAAAATTATCGAAGCCCGCG-
TAGGGGCGATAGTGAGGAGACGTGTAT--TACGAAGTGTGTGTAAAGAACGCACTAATAACT---
GGAGGTCAAGTCTGGTGCCAGCATCCGCGGTAATTCCAGCTCCAGGGGTGTCTATGATGATTGCTGCGATTAA
AAAGTCCGTAGTCAAGCTGACTG-ACTTGCCTGCAATGTGAC-TG------
ATTAAGAGACGAGCAGGGCTAGGAAAGCAGAGAATTAGGAGCGACCGAGGGCTAGAGTATTGAATGGCGAGAG
GTGAAATTTGATGACCCAT-
TCAGGAGTGACAAAGGCGAAGGCACTAGTCAAGGGCGAATCCGATGATCAAGGACGTAGGCTAGAGTTTCGAA
AACGATT--AGAGACCGGAGTAGTTCTAGCAGTAAACTATGCCGACGCCGTGGTATG--GTATT------
CTGTATTGCGGAAGAGAAATC--AAGT-
AAGGCTTTGGGGAGAGTACGCGCGCAAGCGATAAATTTAAAGGAAATTGACGGAGGAACACCACAAGGAGTGG
AGTGTGCGGCTTAATTTGACTCAACGCGGGACAGCTTACCAGGCCCGATAATCATACGAGCGTAGTACGCGAT
AGGTTAGAGAGTGGTGCATGGCTGCTATCGACAGTTGGGGTGACC-TTAGGGTTAATTCCG-
GCAAGTAGTGAGACCTCTGCAGTTA----------TGGACAGGTATTTTT--AAGATACAGGAAGG-
AAGAGACAAGAGCA-GGTCAGTGATGCCCTTAGATGGCCTGGGCT----GCACGCGCACTACAGTGG-
TCATTATAAGTAGAAG-------TT-AGATTTTAAAGA-----------TGATCGAGAGGGA-
CTGGGCTTTG-TAAGAGGCCCAAGAACGAGGAATT-
GCTAGTAATCGTAGGCTCATTAAGATACGATGAATATGTCCCTGTACCTT---
GTACACACCGCCCGTCGTTATCGAAGATGGAATTGTGTGC-GAACGAGC--
AACAAGCGAGTGAGCGCATAGTTCTAGATGTGATAAAAG-
TCGTAACAAGGCAACTGTAGGAGAACCTGTAGTTGGATCATACAGATATAATAA----
AAAGGTATGTTTGTTTTTAATTT-----CCCTG--------CGCAAGGG---ATCGTTTGGTTCTA--
TGTGCGATGAAGGTCGGAGCAGTATCTGATAATGTAGAATGATTGTAATAATTGTTCTATGTGACTGAATTGA

ACA-GTGATGTTCGGGTACTCCTTTGAATTAAGCATATGAGTAAAGGAAGGAAAAGAAACTAAC-
TAGGATTCTCTTATTAGTGGCGAATGAACAGAGAATAGCCC--AAGTGTAATCAATAATAAATAATT------
ATTGAGATGTCTAGTAT---ATTGAC-----
GTGAAAACAATGGAAAGTGTTGCCGTAGAGAGTTATAGCCTCGTAGCGT---
CTAAATTTAATAAGGGAGTAGTTGTGCTCGGTAATGCACAATG--
AATAGGTGGTAGTGTCCATCTAAGGCTAAATATGACATAGAGACCGATAGTGAATAA-----
GTAGAGTGATCGAATATGGAATAGA
>DUDC3
AGC-GTAACGTGGAGCGGTGAAAGGCTCAGTAACGGGCGAGTTATTTGTTC-TCCTGGGA-------
CGGACAACACCGG-GAAACTGGTGGGAAAACGTCTAAGTTGCGG------T-
TTTTTAATCGTGGCGTAAACC------------
ATGTGGTGCAGGAGAGTAAGCTGCCATCCTATCAGTTAGTAAGTAGGGTAAGGGCCTACTTAGACGAAGACGG
GTACGGGGAATGAGGGTTTGATTCCG--
GAGAGGGAGCCTGAGAGACGGCTACCAGGACCAAGGTCAGCAGCAGGCGCGAAAATTATCGAAGCCCGCG-
TAGGGGCGATAGTGAGGAGACGTGTAT--TACGAAGTGTGTGTAAAGAACGCACTAATAACT---
GGAGGTCAAGTCTGGTGCCAGCATCCGCGGTAATTCCAGCTCCAGGGGTGTCTATGATGATTGCTGCGATTAA
AAAGTCCGTAGTCAAGCTGACTG-ACTTGCCTGCAATGTGAC-TG------
ATTAAGAGACGAGCAGGGCTAGGAAAGCAGAGAATTAGGAGCGACCGAGGGCTAGAGTATTGAATGGCGAGAG
GTGAAATTTGATGACCCAT-
TCAGGAGTGACAAAGGCGAAGGCACTAGTCAAGGGCGAATCCGATGATCAAGGACGTAGGCTAGAGTTTCGAA
AACGATT--AGAGACCGGAGTAGTTCTAGCAGTAAACTATGCCGACGCCGTGGTATG--GTATT------
CTGTATTGCGGAAGAGAAATC--AAGT-
AAGGCTTTGGGGAGAGTACGCGCGCAAGCGATAAATTTAAAGGAAATTGACGGAGGAACACCACAAGGAGTGG
AGTGTGCGGCTTAATTTGACTCAACGCGGGACAGCTTACCAGGCCCGATAATCATACGAGCGTAGTACGCGAT
AGGTTAGAGAGTGGTGCATGGCTGCTATCGACAGTTGGGGTGACC-TTAGGGTTAATTCCG-
GCAAGTAGTGAGACCTCTGCAGTTA----------TGGACAGGTATTTTT--AAGATACAGGAAGG-
AAGAGACAAGAGCA-GGTCAGTGATGCCCTTAGATGGCCTGGGCT----GCACGCGCACTACAGTGG-
TCATTATAAGTAGAAG-------TT-AGATTTTAAAGA-----------TGATCGAGAGGGA-
CTGGGCTTTG-TAAGAGGCCCAAGAACGAGGAATT-
GCTAGTAATCGTAGGCTCATTAAGATACGATGAATATGTCCCTGTACCTT---
GTACACACCGCCCGTCGTTATCGAAGATGGAATTGTGTGC-GAACGAGC--
AACAAGCGAGTGAGCGCATAGTTCTAGATGTGATAAAAG-
TCGTAACAAGGCAACTGTAGGAGAACCTGTAGTTGGATCATACAGATATAATAA----
AAAGGTATGTTTGTTTTTAATTT-----CCCTG--------CGCAAGGG---ATCGTTTGGTTCTA--
TGTGCGATGAAGGTCGGAGCAGTATCTGATAATGTAGAATGATTGTAATAATTGTTCTATGTGACTGAATTGA
ACA-GTGATGTTCGGGTACTCCTTTGAATTAAGCATATGAGTAAAGGAAGGAAAAGAAACTAAC-
TAGGATTCTCTTATTAGTGGCGAATGAACAGAGAATAGCCC--AAGTGTAATCAATAATAAATAATT------
ATTGAGATGTCTAGTAT---ATTGAC-----
GTGAAAACAATGGAAAGTGTTGCCGTAGAGAGTTATAGCCTCGTAGCGT---
CTAAATTTAATAAGGGAGTAGTTGTGCTCGGTAATGCACAATG--
AATAGGTGGTAGTGTCCATCTAAGGCTAAATATGACATAGAGACCGATAGTGAATAA-----
GTAGAGTGATCGAATATGGAATAGA
>DUDC7
AGC-GTAACGTGGAGCGGTGAAAGGCTCAGTAACGGGCGAGTTATTTGTTC-TCCTGGGA-------
CGGACAACACCGG-GAAACTGGTGGGAAAACGTCTAAGTTGCGG------T-
TTTTTAATCGTGGCGTAAACC------------
ATGTGGTGCAGGAGAGTAAGCTGCCATCCTATCAGTTAGTAAGTAGGGTAAGGGCCTACTTAGACGAAGACGG
GTACGGGGAATGAGGGTTTGATTCCG--
GAGAGGGAGCCTGAGAGACGGCTACCAGGACCAAGGTCAGCAGCAGGCGCGAAAATTATCGAAGCCCGCG-
TAGGGGCGATAGTGAGGAGACGTGTAT--TACGAAGTGTGTGTAAAGAACGCACTAATAACT---
GGAGGTCAAGTCTGGTGCCAGCATCCGCGGTAATTCCAGCTCCAGGGGTGTCTATGATGATTGCTGCGATTAA
AAAGTCCGTAGTCAAGCTGACTG-ACTTGCCTGCAATGTGAC-TG------
ATTAAGAGACGAGCAGGGCTAGGAAAGCAGAGAATTAGGAGCGACCGAGGGCTAGAGTATTGAATGGCGAGAG
GTGAAATTTGATGACCCAT-
TCAGGAGTGACAAAGGCGAAGGCACTAGTCAAGGGCGAATCCGATGATCAAGGACGTAGGCTAGAGTTTCGAA
AACGATT--AGAGACCGGAGTAGTTCTAGCAGTAAACTATGCCGACGCCGTGGTATG--GTATT------
CTGTATTGCGGAAGAGAAATC--AAGT-
AAGGCTTTGGGGAGAGTACGCGCGCAAGCGATAAATTTAAAGGAAATTGACGGAGGAACACCACAAGGAGTGG

AGTGTGCGGCTTAATTTGACTCAACGCGGGACAGCTTACCAGGCCCGATAATCATACGAGCGTAGTACGCGAT
AGGTTAGAGAGTGGTGCATGGCTGCTATCGACAGTTGGGGTGACC-TTAGGGTTAATTCCG-
GCAAGTAGTGAGACCTCTGCAGTTA----------TGGACAGGTATTTTT--AAGATACAGGAAGG-
AAGAGACAAGAGCA-GGTCAGTGATGCCCTTAGATGGCCTGGGCT----GCACGCGCACTACAGTGG-
TCATTATAAGTAGAAG-------TT-AGATTTTAAAGA-----------TGATCGAGAGGGA-
CTGGGCTTTG-TAAGAGGCCCAAGAACGAGGAATT-
GCTAGTAATCGTAGGCTCATTAAGATACGATGAATATGTCCCTGTACCTT---
GTACACACCGCCCGTCGTTATCGAAGATGGAATTGTGTGC-GAACGAGC--
AACAAGCGAGTGAGCGCATAGTTCTAGATGTGATAAAAG-
TCGTAACAAGGCAACTGTAGGAGAACCTGTAGTTGGATCATACAGATATAATAA----
AAAGGTATGTTTGTTTTTAATTT-----CCCTG--------CGCAAGGG---ATCGTTTGGTTCTA--
TGTGCGATGAAGGTCGGAGCAGTATCTGATAATGTAGAATGATTGTAATAATTGTTCTATGTGACTGAATTGA
ACA-GTGATGTTCGGGTACTCCTTTGAATTAAGCATATGAGTAAAGGAAGGAAAAGAAACTAAC-
TAGGATTCTCTTATTAGTGGCGAATGAACAGAGAATAGCCC--AAGTGTAATCAATAATAAATAATT------
ATTGAGATGTCTAGTAT---ATTGAC-----
GTGAAAACAATGGAAAGTGTTGCCGTAGAGAGTTATAGCCTCGTAGCGT---
CTAAATTTAAAAAGGGAGTAGTTGTGCTCGGTAATGCACAATG--
AATAGGTGGTAGTGTCCATCTAAGGCTAAATATGACATAGAGACCGATAGTGAATAA-----
GTAGAGTGATCGAATATGGAATAGA
>D08-Ct3
AGC-GTAACGTGGAGCGGTGAAAGGCTCAGTAACGGGCGAGTTATTTGTTC-TCCTGGGA-------
CGGACAACACCGG-GAAACTGGTGGGAAAACGTCTAAGTTGCGG------T-
TTTTTAATCGTGGCGTAAACC------------
ATTTGGTGCAGGAGAGTAAGCTGCCATCCTATCAGTTAGTAAGTAGGGTAAGGGCCTACTTAGACGAAGACGG
GTACGGGGAATGAGGGTTTGATTCCG--
GAGAGGGAGCCTGAGAGACGGCTACCAGGACCAAGGTCAGCAGCAGGCGCGAAAATTATCGAAGCCCGCG-
TAGGGGCGATAGTGAGGAGACGTGTAT--TACGAAGTGTGTGTAAAGAACGCACTAATAACT---
GGAGGTCAAGTCTGGTGCCAGCATCCGCGGTAATTCCAGCTCCAGGGGTGTCTATGATGATTGCTGCGATTAA
AAAGTCCGTAGTCAAGCTGACTG-ACTTGCCTGCAATGTGAC-TG------
ATTAAGAGACGAGCAGGGCTAGGAAAGCAGAGAATTAGGAGCGACCGAGGGCTAGAGTATTGAATGGCGAGAG
GTGAAATTTGATGACCCAT-
TCAGGAGTGACAAAGGCGAAGGCACTAGTCAAGGGCGAATCCGATGATCAAGGACGTAGGCTAGAGTTTCGAA
AACGATT--AGAGACCGGAGTAGTTCTAGCAGTAAACTATGCCGACGCCGTGGTATG--GTATT------
CTGTATTGCGGAAGAGAAATC--AAGT-
AAGGCTTTGGGGAGAGTACGCGCGCAAGCGATAAATTTAAAGGAAATTGACGGAGGAACACCACAAGGAGTGG
AGTGTGCGGCTTAATTTGACTCAACGCGGGACAGCTTACCAGGCCCGATAATCATACGAGCGTAGTACGCGAT
AGGTTAGAGAGTGGTGCATGGCTGCTATCGACAGTTGGGGTGACC-TTAGGGTTAATTCCG-
GCAAGTAGTGAGACCTCTGCAGTTA----------TGGACAGGTATTTTT--AAGATACAGGAAGG-
AAGAGACAAGAGCA-GGTCAGTGATGCCCTTAGATGGCCTGGGCT----GCACGCGCACTACAGTGG-
TCATTATAAGTAGAAG-------TT-AGATTTTAAAGA-----------TGATCGAGAGGGA-
CTGGGCTTTG-TAAGAGGCCCAAGAACGAGGAATT-
GCTAGTAATCGTAGGCTCATTAAGATACGATGAATATGTCCCTGTACCTT---
GTACACACCGCCCGTCGTTATCGAAGATGGAATTGTGTGC-GAACGAGC--
AACAAGCGAGTGAGCGCATAGTTCTAGATGTGATAAAAG-
TCGTAACAAGGCAACTGTAGGAGAACCTGTAGTTGGATCATACAGATATAATAA----
AAAGGTATGTTTGTTTTTAATTT-----CCCTG--------CGCAAGGG---ATCGTTTGGTTCTA--
TGTGCGATGAAGGTCGGAGCAGTATCTGATAATGTAGAATGATTGTAATAATTGTTCTATGTGACTGAATTGA
ACA-GTGATGTTCGGGTACTCCTTTGAATTAAGCATATGAGTAAAGGAAGGAAAAGAAACTAAC-
TAGGATTCTCTTATTAGTGGCGAATGAACAGAGAATAGCCC--AAGTGTAATCAATAATAAATAATT------
ATTGAGATGTCTAGTAT---ATTGAC-----
GTGAAAACAATGGAAAGTGTTGTCGTAGAGAGTTATAGCCTCGTAGCGT---
CTAAATTTAAAAAGGGAGTAGTTGTGCTCGGTAATGCACAATG--
AATAGGTGGTAGTGTCCATCTAAGGCTAAATATGACATAGAGACCGATAGTGAATAA-----
GTAGAGTGATCGAATATGGAATAGA
>D08-Ct17
AGC-GTGGAGCGGAGCAGTGAAAGGCTCAGTAACGGGCGAGTTATTTGTTC-TCCTGGGA-------
CGGACAACACCGG-GAAACTGGTGGGAAAACGTCTAAGTTGCGG------T-
TTTTTAATCGTGGCGTAAACC------------

ATTTGGTGCAGGAGAGTAAGCTGCCATCCTATCAGTTAGTAAGTAGGGTAAGGGCCTACTTAGACGAAGACGG
GTACGGGGAATGAGGGTTTGATTCCG--
GAGAGGGAGCCTGAGAGACGGCTACCAGGACCAAGGTCAGCAGCAGGCGCGAAAATTATCGAAGCCCGCG-
TAGGGGCGATAGTGAGGAGACGTGTAT--TACGAAGTGTGTGTAAAGAACGCACTAATAACT---
GGAGGTCAAGTCTGGTGCCAGCATCCGCGGTAATTCCAGCTCCAGGGGTGTCTATGATGATTGCTGCGATTAA
AAAGTCCGTAGTCAAGCTGACTG-ACTTGCCTGCAATGTGAC-TG------
ATTAAGAGACGAGCAGGGCTAGGAAAGCAGAGAATTAGGAGCGACCGAGGGCTAGAGTATTGAATGGCGAGAG
GTGAAATTTGATGACCCAT-
TCAGGAGTGACAAAGGCGAAGGCACTAGTCAAGGGCGAATCCGATGATCAAGGACGTAGGCTAGAGTTTCGAA
AACGATT--AGAGACCGGAGTAGTTCTAGCAGTAAACTATGCCGACGCCGTGGTATG--GTATT------
CTGTATTGCGGAAGAGAAATC--AAGT-
AAGGCTTTGGGGAGAGTACGCGCGCAAGCGATAAATTTAAAGGAAATTGACGGAGGAACACCACAAGGAGTGG
AGTGTGCGGCTTAATTTGACTCAACGCGGGACAGCTTACCAGGCCCGATAATCATACGAGCGTAGTACGCGAT
AGGTTAGAGAGTGGTGCATGGCTGCTATCGACAGTTGGGGTGACC-TTAGGGTTAATTCCG-
GCAAGTAGTGAGACCTCTGCAGTTA----------TGGACAGGTATTTTT--AAGATACAGGAAGG-
AAGAGACAAGAGCA-GGTCAGTGATGCCCTTAGATGGCCTGGGCT----GCACGCGCACTACAGTGG-
TCATTATAAGTAGAAG-------TT-AGATTTTAAAGA-----------TGATCGAGAGGGA-
CTGGGCTTTG-TAAGAGGCCCAAGAACGAGGAATT-
GCTAGTAATCGTAGGCTCATTAAGATACGATGAATATGTCCCTGTACCTT---
GTACACACCGCCCGTCGTTATCGAAGATGGAATTGTGTGC-GAACGAGC--
AACAAGCGAGTGAGCGCATAGTTCTAGATGTGATAAAAG-
TCGTAACAAGGCAACTGTAGGAGAACCTGTAGTTGGATCATACAGATATAATAA----
AAAGGTATGTTTGTTTTTAATTT-----CCCTG--------CGCAAGGG---ATCGTTTGGTTCTA--
TGTGCGATGAAGGTCGGAGCAGTATCTGATAATGTAGAATGATTGTAATAATTGTTCTATGTGACTGAATTGA
ACA-GTGATGTTCGGGTACTCCTTTGAATTAAGCATATGAGTAAAGGAAGGAAAAGAAACTAAC-
TAGGATTCTCTTATTAGTGGCGAATGAACAGAGAATAGCCC--AAGTGTAATCAATAATAAATAATT------
ATTGAGATGTCTAGTAT---ATTGAC-----
GTGAAAACAATGGAAAGTGTTGCCGTAGAGAGTTATAGCCTCGTAGCGT---
CTAAATTTAAAAAGGGAGTAGTTGTGCTCGGTAATGCACAATG--
AATAGGTGGTAGTGTCCATCTAAGGCTAAATATGACATAGAGACCGATAGTGAATAA-----
GTAGAGTGATCGAATATGGAATAGA
>ZWC43
AGC-GTAACGTGGAGCGGTGAAAGGCTCAGTAACGGGCGAGTTATTTGTTC-TCCTGGGA-------
CGGACAACACCGG-GAAACTGGTGGGAAAACGTCTAAGTTGCGG------T-
TTTTTAATCGTGGCGTAAACC------------
ATTTGGTGCAGGAGAGTAAGCTGCCATCCTATCAGTTAGTAAGTAGGGTAAGGGCCTACTTAGACGAAGACGG
GTACGGGGAATGAGGGTTTGATTCCG--
GAGAGGGAGCCTGAGAGACGGCTACCAGGACCAAGGTCAGCAGCAGGCGCGAAAATTATCGAAGCCCGCG-
TAGGGGCGATAGTGAGGAGACGTGTAT--TACGAAGTGTGTGTAAAGAACGCACTAATAACT---
GGAGGTCAAGTCTGGTGCCAGCATCCGCGGTAATTCCAGCTCCAGGGGTGTCTATGATGATTGCTGCGATTAA
AAAGTCCGTAGTCAAGCTGACTG-ACTTGCCTGCAATGTGAC-TG------
ATTAAGAGACGAGCAGGGCTAGGAAAGCAGAGAATTAGGAGCGACCGAGGGCTAGAGTATTGAATGGCGAGAG
GTGAAATTTGATGACCCAT-
TCAGGAGTGACAAAGGCGAAGGCACTAGTCAAGGGCGAATCCGATGATCAAGGACGTAGGCTAGAGTTTCGAA
AACGATT--AGAGACCGGAGTAGTTCTAGCAGTAAACTATGCCGACGCCGTGGTATG--GTATT------
CTGTATTGCGGAAGAGAAATC--AAGT-
AAGGCTTTGGGGAGAGTACGCGCGCAAGCGATAAATTTAAAGGAAATTGACGGAGGAACACCACAAGGAGTGG
AGTGTGCGGCTTAATTTGACTCAACGCGGGACAGCTTACCAGGCCCGATAATCATACGAGCGTAGTACGCGAT
AGGTTAGAGAGTGGTGCATGGCTGCTATCGACAGTTGGGGTGACC-TTAGGGTTAATTCCG-
GCAAGTAGTGAGACCTCTGCAGTTA----------TGGACAGGTATTTTT--AAGATACAGGAAGG-
AAGAGACAAGAGCA-GGTCAGTGATGCCCTTAGATGGCCTGGGCT----GCACGCGCACTACAGTGG-
TCATTATAAGTAGAAG-------TT-AGATTTTAAAGA-----------TGATCGAGAGGGA-
CTGGGCTTTG-TAAGAGGCCCAAGAACGAGGAATT-
GCTAGTAATCGTAGGCTCATTAAGATACGATGAATATGTCCCTGTACCTT---
GTACACACCGCCCGTCGTTATCGAAGATGGAATTGTGTGC-GAACGAGC--
AACAAGCGAGTGAGCGCATAGTTCTAGATGTGATAAAAG-
TCGTAACAAGGCAACTGTAGGAGAACCTGTAGTTGGATCATACAGATATAATAA----
AAAGGTATGTTTGTTTTTAATTT-----CCCTG--------CGCAAGGG---ATCGTTTGGTTCTA--

TGTGCGATGAAGGTCGGAGCAGTATCTGATAATGTAGAATGATTGTAATAATTGTTCTATGTGACTGAATTGA
ACA-GTGATGTTCGGGTACTCCTTTGAATTAAGCATATGAGTAAAGGAAGGAAAAGAAACTAAC-
TAGGATTCTCTTATTAGTGGCGAATGAACAGAGAATAGCCC--AAGTGTAATCAATAATAAATAATT------
ATTGAGATGTCTAGTAT---ATTGAC-----
GTGAAAACAATGGAAAGTGTTGCCGTAGAGAGTTATAGCCTCGTAGCGT---
CTAAATTTAAAAAGGGAGTAGTTGTGCTCGGTAATGCACAATG--
AATAGGTGGTAGTGTCCATCTAAGGCTAAATATGACATAGAGACCGATAGTGAATAA-----
GTAGAGTGATCGAATATGGAATAGA
>DHAE_DOP2
AGC-GTAACGTGGAGCGGTGAAAGGCTCAGTAACGGGCGAGTTATTTGTTC-TCCTGGGA-------
CGGACAACACCGG-GAAACTGGTGGGAAAACGTCTAAGTTGCGG------T-
TTTTTAATCGTGGCGTAAACC------------
ATGTGGTGCAGGAGAGTAAGCTGCCATCCTATCAGTTAGTAAGTAGGGTAAGGGCCTACTTAGACGAAGACGG
GTACGGGGAATGAGGGTTTGATTCCG--
GAGAGGGAGCCTGAGAGACGGCTACCAGGACCAAGGTCAGCAGCAGGCGCGAAAATTATCGAAGCCCGCG-
TAGGGGCGATAGTGAGGAGACGTGTAT--TACGAAGTGTGTGTAAAGAACGCACTAATAACT---
GGAGGTCAAGTCTGGTGCCAGCATCCGCGGTAATTCCAGCTCCAGGGGTGTCTATGATGATTGCTGCGATTAA
AAAGTCCGTAGTCAAGCTGACTG-ACTTGCCTGCAATGTGAC-TG------
ATTAAGAGACGAGCAGGGCTAGGAAAGCAGAGAATTAGGAGCGACCGAGGGCTAGAGTATTGAATGGCGAGAG
GTGAAATTTGATGACCCAT-
TCAGGAGTGACAAAGGCGAAGGCACTAGTCAAGGGCGAATCCGATGATCAAGGACGTAGGCTAGAGTTTCGAA
AACGATT--AGAGACCGGAGTAGTTCTAGCAGTAAACTATGCCGACGCCGTGGTATG--GTATT------
CTGTATTGCGGAAGAGAAATC--AAGT-
AAGGCTTTGGGGAGAGTACGCGCGCAAGCGATAAATTTAAAGGAAATTGACGGAGGAACACCACAAGGAGTGG
AGTGTGCGGCTTAATTTGACTCAACGCGGGACAGCTTACCAGGCCCGATAATCATACGAGCGTAGTACGCGAT
AGGTTAGAGAGTGGTGCATGGCTGCTATCGACAGTTGGGGTGACC-TTAGGGTTAATTCCG-
GCAAGTAGTGAGACCTCTGCAGTTA----------TGGACAGGTATTTTT--AAGATACAGGAAGG-
AAGAGACAAGAGCA-GGTCAGTGATGCCCTTAGATGGCCTGGGCT----GCACGCGCACTACAGTGG-
TCATTATAAGTAGAAG-------TT-AGATTTTAAAAA-----------TGATCGAGAGGGA-
CTGGGCTTTG-TAAGAGGCCCAAGAACGAGGAATT-
GCTAGTAATCGTAGGCTCATTAAGATACGATGAATATGTCCCTGTACCTT---
GTACACACCGCCCGTCGTTATCGAAGATGGAATTGTGTGC-GAACGAGC--
AACAAGCGAGTGAGCGCATAGTTCTAGATGTGATAAAAG-
TCGTAACAAGGCAACTGTAGGAGAACCTGTAGTTGGATCATACAGATATAATAA----
AAAGGTATGTTTGTTTTTAATTT-----CCCTG--------CGCAAGGG---ATCGTTTGGTTCTA--
TGTGCGATGAAGGTCGGAGCAGTATCTGATAATGTAGAATGATTGTAATAATTGTTCTATGTGACTGAATTGA
ACA-GTGATGTTCGGGTACTCCTTTGAATTAAGCATATGAGTAAAGGAAGGAAAAGAAACTAAC-
TAGGATTCTCTTATTAGTGGCGAATGAACAGAGAATAGCCC--AAGTGTAATCAATAATAAATAATT------
ATTGAGATGTCTAGTAT---ATTGAC-----
GTGAAAACAATGGAAAGTGTTGCCGTAGAGAGTTATAGCCTCGTAGCGT---
CTAAATTTAAAAAGGGAGTAGTTGTGCTCGGTAATGCACAATG--
AATAGGTGGTAGTGTCCATCTAAGGCTAAATATGACATAGAGACCGATAGTGAATAA-----
GTAGAGTGATCGAATATGGAATAGA
>DHAE_DOP13
AGC-GTAACGTGGAGCGGTGAAAGGCTCAGTAACGGGCGAGTTATTTGTTC-TCCTGGGA-------
CGGACAACACCGG-GAAACTGGTGGGAAAACGTCTAAGTTGCGG------T-
TTTTTAATCGTGGCGTAAACC------------
ATGTGGTGCAGGAGAGTAAGCTGCCATCCTATCAGTTAGTAAGTAGGGTAAGGGCCTACTTAGACGAAGACGG
GTACGGGGAATGAGGGTTTGATTCCG--
GAGAGGGAGCCTGAGAGACGGCTACCAGGACCAAGGTCAGCAGCAGGCGCGAAAATTATCGAAGCCCGCG-
TAGGGGCGATAGTGAGGAGACGTGTAT--TACGAAGTGTGTGTAAAGAACGCACTAATAACT---
GGAGGTCAAGTCTGGTGCCAGCATCCGCGGTAATTCCAGCTCCAGGGGTGTCTATGATGATTGCTGCGATTAA
AAAGTCCGTAGTCAAGCTGACTG-ACTTGCCTGCAATGTGAC-TG------
ATTAAGAGACGAGCAGGGCTAGGAAAGCAGAGAATTAGGAGCGACCGAGGGCTAGAGTATTGAATGGCGAGAG
GTGAAATTTGATGACCCAT-
TCAGGAGTGACAAAGGCGAAGGCACTAGTCAAGGGCGAATCCGATGATCAAGGACGTAGGCTAGAGTTTCGAA
AACGATT--AGAGACCGGAGTAGTTCTAGCAGTAAACTATGCCGACGCCGTGATATG--GTATT------
CTGTATTGCGGAAGAGAAATC--AAGT-

AAGGCTTTGGGGAGAGTACGCGCGCAAGCGATAAATTTAAAGGAAATTGACGGAGGAACACCACAAGGAGTGG
AGTGTGCGGCTTAATTTGACTCAACGCGGGACAGCTTACCATGCCCGATAATCATACGAGCGTAGTACGCGAT
AGGTTAGAGAGTGGTGCATGGCTGCTATCGACAGTTGGGGTGACC-TTAGGGTTAATTCCG-
GCAAGTAGTGAGACCTCTGCAGTTA----------TGGACAGGTATTTTT--AAGATACAGGAAGG-
AAGAGACAAGAGCA-GGTCAGTGATGCCCTTAGATGGCCTGGGCT----GCACGCGCACTACAGTGG-
TCATTATAAGTAGAAG-------TT-AGATTTTAAAGA-----------TGATCGAGAGGGA-
CTGGGCTTTG-TAAGAGGCCCAAGAACGAGGAATT-
GCTAGTAATCGTAGGCTCATTAAGATACGATGAATATGTCCCTGTACCTT---
GTACACACCGCCCGTCGTTATCGAAGATGGAATTGTGTGC-GAACGAGC--
AACAAGCGAGTGAGCGCATAGTTCTAGATGTGATAAAAG-
TCGTAACAAGGCAACTGTAGGAGAACCTGTAGTTGGATCATACAGATATAATAA----
AAAGGTATGTTTGTTTTTAATTT-----CCCTG--------CGCAAGGG---ATCGTTTGGTTCTA--
TGTGCGATGAAGGTCGGAGCAGTATCTGATAATGTAGAATGATTGTAATAATTGTTCTATGTGACTGAATTGA
ACA-GTGATGTTCGGGTACTCCTTTGAATTAAGCATATGAGTAAAGGAAGGAAAAGAAACTAAC-
TAGGATTCTCTTATTAGTGGCGAATGAACAGAGAATAGCCC--AAGTGTAATCAATAATAAATAATT------
ATTGAGATGTCTAGTAT---ATTGAC-----
GTGAAAACAATGGAAAGTGTTGCCGTAGAGAGTTATAGCCTCGTAGCGT---
CTAAATTTAAAAAGGGAGTAGTTGTGCTCGGTAATGCACAATG--
AATAGGTGGTAGTGTCCATCTAAGGCTAAATATGACATAGAGACCGATAGTGAATAA-----
GTAGAGTGATCGAATATGGAATAGA
>DHAE_DOR9
AGC-GTAACGTGGAGCGGTGAAAGGCTCAGTAACGGGCGAGTTATTTGTTC-TCCTGGGA-------
CGGACAACACCGG-GAAACTGGTGGGAAAACGTCTAAGTTGCGG------T-
TTTTTAATCGTGGCGTAAACC------------
ATGTGGTGCAGGAGAGTAAGCTGCCATCCTATCAGTTAGTAAGTAGGGTAAGGGCCTACTTAGACGAAGACGG
GTACGGGGAATGAGGGTTTGATTCCG--
GAGAGGGAGCCTGAGAGACGGCTACCAGGACCAAGGTCAGCAGCAGGCGCGAAAATTATCGAAGCCCGCG-
TAGGGGCGATAGTGAGGAGACGTGTAT--TACGAAGTGTGTGTAAAGAACGCACTAATAACT---
GGAGGTCAAGTCTGGTGCCAGCATCCGCGGTAATTCCAGCTCCAGGGGTGTCTATGATGATTGCTGCGATTAA
AAAGTCCGTAGTCAAGCTGACTG-ACTTGCCTGCAATGTGAC-TG------
ATTAAGAGACGAGCAGGGCTAGGAAAGCAGAGAATTAGGAGCGACCGAGGGCTAGAGTATTGAATGGCGAGAG
GTGAAATTTGATGACCCAT-
TCAGGAGTGACAAAGGCGAAGGCACTAGTCAAGGGCGAATCCGATGATCAAGGACGTAGGCTAGAGTTTCGAA
AACGATT--AGAGACCGGAGTAGTTCTAGCAGTAAACTATGCCGACGCCGTGGTATG--GTATT------
CTGTATTGCGGAAGAGAAATC--AAGT-
AAGGCTTTGGGGAGAGTACGCGCGCAAGCGATAAATTTAAAGGAAATTGACGGAGGAACACCACAAGGAGTGG
AGTGTGCGGCTTAATTTGACTCAACGCGGGACAGCTTACCATGCCCGATAATCATACGAGCGTAGTACGCGAT
AGGTTAGAGAGTGGTGCATGGCTGCTATCGACAGTTGGGGTGACC-TTAGGGTTAATTCCG-
GCAAGTAGTGAGACCTCTGCAGTTA----------TGGACAGGTATTTTT--AAGATACAGGAAGG-
AAGAGACAAGAGCA-GGTCAGTGATGCCCTTAGATGGCCTGGGCT----GCACGCGCACTACAGTGG-
TCATTATAAGTAGAAG-------TT-AGATTTTAAAAA-----------TGATCGAGAGGGA-
CTGGGCTTTG-TAAGAGGCCCAAGAACGAGGAATT-
GCTAGTAATCGTAGGCTCATTAAGATACGATGAATATGTCCCTGTACCTT---
GTACACACCGCCCGTCGTTATTGAAGATGGAATTGTGTGC-GAACGAGC--
AACAAGCGAGTGAGCGCATAGTTCTAGATGTGATAAAAG-
TCGTAACAAGGCAACTGTAGGAGAACCTGTAGTTGGATCATACAGATATAATAA----
AAAGGTATGTTTGTTTTTAATTT-----CCCTG--------CGCAAGGG---ATCGTTTGGTTCTA--
TGTGCGATGAAGGTCGGAGCAGTATCTGATAATGTAGAATGATTGTAATAATTGTTCTATGTGACTGAATTGA
ACA-GTGATGTTCGGGTACTCCTTTGAATTAAGCATATGAGTAAAGGAAGGAAAAGAAACTAAC-
TAGGATTCTCTTATTAGTGGCGAATGAACAGAGAATAGCCC--AAGTGTAATCAATAATAAATAATT------
ATTGAGATGTCTAGTAT---ATTGAC-----
GTGAAAACAATGGAAAGTGTTGCCGTAGAGAGTTATAGCCTCGTAGCGT---
CTAAATTTAAAAAGGGAGTAGTTGTGCTCGGTAATGCACAATG--
AATAGGTGGTAGTGTCCATCTAAGGCTAAATATGACATAGAGACCGATAGTGAATAA-----
GTAGAGTGATCGAATATGGAATAGA
>ZWPr2
AGC-GTAACGTGGAGCGGTGAAAGGCTCAGTAACGGGCGAGTTATTTGTTC-TCCTGGGA-------
CGGACAACACCGG-GAAACTGGTGGGAAAACGTCTAAGTTGCGG------T-

TTTTTAATCGTGGCGTAAACC------------
ATTTGGTGCAGGAGAGTAAGCTGCCATCCTATCAGTTAGTAAGTAGGGTAAGGGCCTACTTAGACGAAGACGG
GTACGGGGAATGAGGGTTTGATTCCG--
GAGAGGGAGCCTGAGAGACGGCTACCAGGACCAAGGTCAGCAGCAGGCGCGAAAATTATCGAAGCCCGCG-
TAGGGGCGATAGTGAGGAGACGTGTAT--TACGAAGTGTGTGTAAAGAACGCACTAATAACT---
GGAGGTCAAGTCTGGTGCCAGCATCCGCGGTAATTCCAGCTCCAGGGGTGTCTATGATGATTGCTGCGATTAA
AAAGTCCGTAGTCAAGCTGACTG-ACTTGCCTGCAATGTGAC-TG------
ATTAAGAGACGAGCAGGGCTAGGAAAGCAGAGAATTAGGAGCGACCGAGGGCTAGAGTATTGAATGGCGAGAG
GTGAAATTTGATGACCCAT-
TCAGGAGTGACAAAGGCGAAGGCACTAGTCAAGGGCGAATCCGATGATCAAGGACGTAGGCTAGAGTTTCGAA
AACGATT--AGAGACCGGAGTAGTTCTAGCAGTAAACTATGCCGACGCCGTGGTATG--GTATT------
CTGTATTGCGGAAGAGAAATC--AAGT-
AAGGCTTTGGGGAGAGTACGCGCGCAAGCGATAAATTTAAAGGAAATTGACGGAGGAACACCACAAGGAGTGG
AGTGTGCGGCTTAATTTGACTCAACGCGGGACAGCTTACCAGGCCCGATAATCATACGAGCGTAGTACGCGAT
AGGTTAGAGAGTGGTGCATGGCTGCTATCGACAGTTGGGGTGACC-TTAGGGTTAATTCCG-
GCAAGTAGTGAGACCTCTGCAGTTA----------TGGACAGGTATTTTT--AAGATACAGGAAGG-
AAGAGACAAGAGCA-GGTCAGTGATGCCCTTAGATGGCCTGGGCT----GCACGCGCACTACAGTGG-
TCATTATAAGTAGAAG-------TT-AGATTTTAAAGA-----------TGATCGAGAGGGA-
CTGGGCTTTG-TAAGAGGCCCAAGAACGAGGAATT-
GCTAGTAATCGTAGGCTCATTAAGATACGATGAATATGTCCCTGTACCTT---
GTACACACCGCCCGTCGTTATCGAAGATGGAATTGTGTGC-GAACGAGC--
AACAAGCGAGTGAGCGCATAGTTCTAGATGTGATAAAAG-
TCGTAACAAGGCAACTGTAGGAGAACCTGTAGTTGGATCATACAGATATAATAA----
AAAGGTAGGTTTGTTTTTAATTT-----CCCTG--------CGCAAGGG---ATCGTTTGGTTCTA--
TGTGCGATGAAGGTCGGAGCAGTATCTGATAATGTAGAATGATTGTAATAATTGTTCTATGTGACTGAATTGA
ACA-GTGATGTTCGGGTACTCCTTTGAATTAAGCATATGAGTAAAGGAAGGAAAAGAAACTAAC-
TAGGATTCTCTTATTAGTGGCGAATGAACAGAGAATAGCCC--AAGTGTAATCAATAATAAATAATT------
ATTGAGATGTCTAGTAT---ATTGAC-----
GTGAAAACAATGGAAAGTGTTGCCGTAGAGAGTTATAGCCTCGTAGCGT---
CTAAATTTAAAAAGGGAGTAGTTGTGCTCGGTAATGCACAATG--
AATAGGTGGTAGTGTCCATCTAAGGCTAAATATGTCATAGAGACCGATAGTGAATAA-----
GTAGAGTGATCGAATATGGAATAGA
>DHAE_DML3
AGC-GTAACGTGGAGCGGTGAAAGGCTCAGTAACGGGCGAGTTATTTGTTC-TCCTGGGA-------
CGGACAACACCGG-GAAACTGGTGGGAAAACGTCTAAGTTGCGG------T-
TTTTTAATCGTGGCGTAAACC------------
ATGTGGTGCAGGAGAGTAAGCTGCCATCCTATCAGTTAGTAAGTAGGGTAAGGGCCTACTTAGACGAAGACGG
GTACGGGGAATGAGGGTTTGATTCCG--
GAGAGGGAGCCTGAGAGACGGCTACCAGGACCAAGGTCAGCAGCAGGCGCGAAAATTATCGAAGCCCGCG-
TAGGGGCGATAGTGAGGAGACGTGTAT--TACGAAGTGTGTGTAAAGAACGCACTAATAACT---
GGAGGTCAAGTCTGGTGCCAGCATCCGCGGTAATTCCAGCTCCAGGGGTGTCTATGATGATTGCTGCGATTAA
AAAGTCCGTA-TCAAGCTGACTG-ACTTGCCTGCAATGTGAC-TG------
ATTAAGAGACGAGCAGGGCTAGGAAAGCAGAGAATTAGGAGCGACCGAGGGCTAGAGTATTGAATGGCGAGAG
GTGAAATTTGATGACCCAT-
TCAGGAGTGACAAAGGCGAAGGCACTAGTCAAGGGCGAATCCGATGATCAAGGACGTAGGCTAGAGTTTCGAA
AACGATT--AGAGACCGGAGTAGTTCTAGCAGTAAACTATGCCGACGCCGTGGTATG--GTATT------
CTGTATTGCGGAAGAGAAATC--AAGT-
AAGGCTTTGGGGAGAGTACGCGCGCAAGCGATAAATTTAAAGGAAATTGACGGAGGAACACCACAAGGAGTGG
AGTGTGCGGCTTAATTTGACTCAACGCGGGACAGCTTACCAGGCCCGATAATCATACGAGCGTAGTACGCGAT
AGGTTAGAGAGTGGTGCATGGCTGCTATCGACAGTTGGGGTGACC-TTAGGGTTAATTCCG-
GCAAGTACTGAGACCTCTGCAGTTA----------TGGACAGGTATTTTT--AAGATACAGGAAGG-
AAGAGACAAGAGCA-GGTCAGTGATGCCCTTAGATGGCCTGGGCT----GCACGCGCACTACAGTGG-
TCATTATAAGTAGAAG-------TT-AGATTTTAAAGA-----------TGATCGAGAGGGA-
CTGGGCTTTG-TAAGAGGCCCAAGAACGAGGAATT-
GCTAGTAATCGTAGGCTCATTAAGATACGATGAATATGTCCCTGTACCTT---
GTACACACCGCCCGTCGTTATCGAAGATGGAATTGTGTGC-GAACGAGC--
AACAAGCGAGTGAGCGCATAGTTCTAGATGTGATAAAAG-
TCGTAACAAGGCAACTGTAGGAGAACCTGTAGTTGGATCATACAGATATAATAA----

AAAGGTATGTTTGTTTTTAATTT-----CCCTG--------CGCAAGGG---ATCGTTTGGTTCTA--
TGTGCGATGAAGGTCGGAGCAGTATCTGATAATGTAGAATGATTGTAATAATTGTTCTATGTGACTGAATTGA
ACA-GTGATGTTCGGGTACTCCTTTGAATTAAGCATATGAGTAAAGGAAGGAAAAGAAACTAAC-
TAGGATTCTCTTATTAGTGGCGAATGAACAGAGAATAGCCC--AAGTGTAATCAATAATAAATAATT------
ATTGAGATGTCTAGTAT---ATTGAC-----
GTGAAAACAATGGAAAGTGTTGCCGTAGAGAGTTATAGCCTCGTAGCGT---
CTAAATTTAAAAAGGGAGTAGTTGTGCTCGGTAATGCACAATG--
AATAGGTGGTAGTGTCCATCTAAGGCTAAATATGACATAGAGACCGATAGTGAATAA-----
GTAGAGTGATCGAATATGGAATAGA
>DVIL_TBQ33-9
AGC-GTAACGTGGAGCGGTGAAAGGCTCAGTAACGGGCGAGTTATTTGTTC-TCCTGGGA-------
CGGACAACACCGG-GAAACTGGTGGGAAAACGTCTAAGTTGCGG------T-
TTTTTAATCGTGGCGTAAACC------------
ATGTGGTGCAGGAGAGTAAGCTGCCATCCTATCAGTTAGTAAGTAGGGTAAGGGCCTACTTAGACGAAGACGG
GTACGGGGAATGAGGGTTTGATTCCG--
GAGAGGGAGCCTGAGAGACGGCTACCAGGACCAAGGTCAGCAGCAGGCGCGAAAATTATCGAAGCCCGCG-
TAGGGGCGATAGTGAGGAGACGTGTAT--TACGAAGTGTGTGTAAAGAACGCACTAATAACT---
GGAGGTCAAGTCTGGTGCCAGCATCCGCGGTAATTCCAGCTCCAGGGGTGTCTATGATGATTGCTGCGATTAA
AAAGTCCGTAGTCAAGCTGACTG-ACTTGCCTGCAATGTGAC-TG------
ATTAAGAGACGAGCAGGGCTAGGAAAGCAGAGAATTAGGAGCGACCGAGGGCTAGAGTATTGAATGGCGAGAG
GTGAAATTTGATGACCCAT-
TCAGGAGTGACAAAGGCGAAGGCACTAGTCAAGGGCGAATCCGATGATCAAGGACGTAGGCTAGAGTTTCGAA
AACGATT--AGAGACCGGAGTAGTTCTAGCAGTAAACTATGCCGACGCCGTGGTATG--GTATT------
CTGTATTGCGGAAGAGAAATC--AAGT-
AAGGCTTTGGGGAGAGTACGCGCGCAAGCGATAAATTTAAAGGAAATTGACGGAGGAACACCACAAGGAGTGG
AGTGTGCGGCTTAATTTGACTCAACGCGGGACAGCTTACCAGGCCCGATAATCATACGAGCGTAGTACGCGAT
AGGTTAGAGAGTGGTGCATGGCTGCTATCGACAGTTGGGGTGACC-TTAGGGTTAATTCCG-
GCAAGTAGTGAGACCTCTGCAGTTA----------TGGACAGGTATTTTT--AAGATACAGGAAGG-
AAGAGACAAGAGCA-GGTCAGTGATGCCCTTAGATGGCCTGGGCT----GCACGCGCACTACAGTGG-
TCATTATAAGTAGAAG-------TT-AGATTTTAAAGA-----------TGATCGAGAGGGA-
CTGGGCTTTG-TAAGAGGCCCAAGAACGAGGAATT-
GCTAGTAATCGTAGGGTCATTAAAATACGATGAATATGTCCCTGTACCTT---
GTACACACCGCCCGTCGTTATCGAAGATGGAATTGTGTGC-GAACGAGC--
AACAAGCGAGTGAGCGCATAGTTCTAGATGTGATAAAAG-
TCGTAACAAGGCAACTGTAGGAGAACCTGTAGTTGGATCATACAGATATAATAA----
AAAGGTATGTTTGTTTTTAATTT-----CCCTG--------CGCAAGGG---ATCGTTTGGTTCTA--
TGTGCGATGAAGGTCGGAGCAGTATCTGATAATGTAGAATGATTGTAATAATTGTTCTATGTGACTGAATTGA
ACA-GTGATGTTCGGGTACTCCTTTGAATTAAGCATATGAGTAAAGGAAGGAAAAGAAACTAAC-
TAGGATTCTCTTATTAGTGGCGAATGAACAGAGAATAGCCC--AAGTGTAATCAATAATAAATAATT------
ATTGAGATGTCTAGTAT---ATTGAC-----
GTGAAAACAATGGAAAGTGTTGCCGTAGAGAGTTATAGCCTCGTAGCGT---
CTAAATTTAAAAAGGGAGTAGTTGTGCTCGGTAATGCACAATG--
AATAGGTGGTAGTGTCCATCTAAGGCTAAATATGACATAGAGACCGATAGTGAATAA-----
GTAGAGTGATCGAATATGGAATAGA
>DVIL-UA29A-19
AGC-GTAACGTGGAGCGGTGAAAGGCTCAGTAACGGGCGAGTTATTTGTTC-TCCTGGGA-------
CGGACAACACCGG-GAAACTGGTGGGAAAACGTCTAAGTTGCGG------T-
TTTTTAATCGTGGCGTAAACC------------
ATGTGGTGCAGGAGAGTAAGCTGCCATCCTATCAGTTAGTAAGTAGGGTAAGGGCCTACTTAGACGAAGACGG
GTACGGGGAATGAGGGTTTGATTCCG--
GAGAGGGAGCCTGAGAGACGGCTACCAGGACCAAGGTCAGCAGCAGGCGCGAAAATTATCGAAGCCCGCG-
TAGGGGCGATAGTGAGGAGACGTGTAT--TACGAAGTGTGTGTAAAGAACGCACTAATAACT---
GGAGGTCAAGTCTGGTGCCAGCATCCGCGGTAATTCCAGCTCCAGGGGTGTCTATGATGATTGCTGCGATTAA
AAAGTCCGTAGTCAAGCTGACTG-ACTTGCCTGCAATGTGAC-TG------
ATTAAGAGACGAGCAGGGCTAGGAAAGCAGAGAATTAGGAGCGACCGAGGGCTAGAGTATTGAATGGCGAGAG
GTGAAATTTGATGACCCAT-
TCAGGAGTGACAAAGGCGAAGGCACTAGTCAAGGGCGAATCCGATGATCAAGGACGTAGGCTAGAGTTTCGAA
AACGATT--AGAGACCGGAGTAGTTCTAGCAGTAAACTATGCCGACGCCGTGGTATG--GTATT------

```
CTGTATTGCGGAAGAGAAATC--AAGT-
AAGGCTTTGGGGAGAGTACGCGCGCAAGCGATAAATTTAAAGGAAATTGACGGAGGAACACCACAAGGAGTGG
AGTGTGCGGCTTAATTTGACTCAACGCGGGACAGCTTACCAGGCCCGATAATCATACGAGCGTAGTACGCGAT
AGGTTAGAGAGTGGTGCATGGCTGCTATCGACAGTTGGGGTGACC-TTAGGGTTAATTCCG-
GCAAGTAGTGAGACCTCTGCAGTTA----------TGGACAGGTATTTTT--AAGATACAGGAAGG-
AAGAGACAAGAGCA-GGTCAGTGATGCCCTTAGATGGCCTGGGCT----GCACGCGCACTACAGTGG-
TCATTATAAGTAGAAG-------TT-AGATTTTAAAGA-----------TGATCGAGAGGGA-
CTGGGCTTTG-TAAGAGGCCCAAGAACGAGGAATT-
GCTAGTAATCGTAGGCTCATTAAGATACGATGAATATGTCCCTGTACCTT---
GTACACACCGCCCGTCGTTATCGAAGATGGAATTGTGTGC-GAACGAGC--
AACAAGCGAGTGAGCGCATAGTTCTAGATGTGATAAAAG-
TCGTAACAAGGCAACTGTAGGAGAACCTGTAGTTGGATCATACAGATATAATAA----
AAAGGTAGGTTTGTTTTTAATTT-----CCCTG--------CGCAAGGG---ATCGTTTGGTTCTA--
TGTGCGATGAAGGTCGGAGCAGTATCTGATAATGTAGAATGATTGTAATAATTGTTCTATGTGACTGAATTGA
ACA-GTGATGTTCGGGTACTCCTTTGAATTAAGCATATGAGTAAAGGAAGGAAAAGAAACTAAC-
TAGGATTCTCTTATTAGTGGCGAATGAACAGAGAATAGCCC--AAGTGTAATCAATAATAAATAATT------
ATTGAGATGTCTAGTAT---ATTGAC-----
GTGAAAACAATGGAAAGTGTTGCCGTAGAGAGTTATAGCCTCGTAGCGT---
CTAAATTTAAAAAGGGAGTAGTTGTGCTCGGTAATGCACAATG--
AATAGGTGGTAGTGTCCATCTAAGGCTAAATATGACATAGAGACCGATAGTGAATAA-----
GTAGAGTGATCGAATATGGAATAGA
>DVIL-UA29A-62
AGC-TTTTAGTGGAGCGGTATACGGCTCAGTAACGGGCGATTTATTTGTTC-TCCTGGGA-------
CGGACAACATCGG-GAAACTGATGGGAAAACGTCTAAGTTGCAG------T-
TAATTGATTGTGACGTAAACC------------
TTCGTGTGCAGGAGAGTAAGATGCCATCCTATCAGTTAGTAAGTAGGGTAAGGGCCTACTTAGACGAAGACGG
GTACGGGGAATGAGGGTTTGATTCCG--
GAGAGGGAGCCTGAGAGATGGCTACCAGGACCAAGGTCAGCAGCAGGCGCGAAAATTATCGAAGCCCGCC-
TAGGGGCGATAGTGAGGAGACGTGTAT--AACGAAGTACGTGTAAAGAACGTGCTAATAACT---
GGAGGTCAAGTCTGGTGCCAGCATCCGCGGTAATTCCAGCTCCAGGGGTGTCTATGATGATTGCTGCGATTAA
AAAGTCCGTAGTCAATCTGACTG-ACTGACCTGCAATGTGAT-TG------
ATTAAAGAACGAGCAGGGTTAGGAAAGCAGAGAATTAGGAGCGACCGAGGGCTAGAGTATTGAATGGCGAGAG
GTGAAATTTGATGACCCAT-
TCAGGAGTGACAAAGGCGAAGGCACTAGTCAAGGGCGAATCCGATGATCAAGGACGTAGGCTAGAGTTTCGAA
AACGATT--AGAGACCGGAGTAGTTCTAGCAGTAAACTATGCCGACGCCGTGGTATC--AACAA------
TGGTATTGCGGAAGAGAAATC--AAGT-
AAGGCTTTGGGGAGAGTACGCGCGCAAGCGATAAATTTAAAGGAAATTGACGGAGGAACACCACAAGGAGTGG
AGTGTGCGGCTTAATTTGACTCAACGCGGGACAGCTTACCAGGCCCGATAATCGAGCGAGCGTAGTACGCGAT
AGGTTAAAGAGTGGTGCATGGCTGCTATCGACAGTTGGGGTGACC-TTAGGGTTAATTCCG-
GCAAGTAGTGAGACCCCTGCAGATAG---------TGGACAGGTATTTTT--AAAATACAGGAAGG-
AAGGGACAAGAGCA-GGTCAGTGATGCCCTTAGATGGCCTGGGCT----GCACGCGCACTACAGTGG-
TCATTATAAGTAGAAG-------TT-AGA-TATAAAGA-----------TGATCGAGAGGGA-
CTGAGCTTTG-TAAGAGGCTCACGAACGAGGAATT-
GCTAGTAATCGTAGGCTCATTAAGATACGATGAATATGTCCCTGTACCTT---
GTACACACCGCCCGTCGTTATCGAAGATGGAATTGTGTGC-GAACGAGC--
AACAAGCGAGTGAGCGCATAGTTCTAGATGTGATAAAAG-
TCGTAACAAGGCAACTGTAGGAGAACCTGTAGTTGGATCATACAGATTTTCTTA----ATAGAAGTTTG---
AGAAGTATT-----CCTTA--------CGCAAGGG---ATCGTTTGGTTCTA--
TGTACGATGAAGGTCGGAGCTGTATTCGATAAAATGAGATGATTGTATAAATTGTCTTATATTACTGAATAGA
GTA-GCGATGCTCGAATACTCCTTTGAATTAAGCATATGAGTAAAGGAAGGAAAAGGAACTAAC-
AAGGATTCTCTTATTAGTGGCGAATGAACAGAGAAGAGCCC-TAAGTGTAATCAAATTCTCTGAAT-------
-TTGAATTGTCAGGCAT--AGAGGAT-----
GCGAATATATTGGAAAATATAGCCATAGAGAGTTAAAGCCTCGTAGCA----
TCTTAAATTAGGGATGAGTAGTTGTGCTCGGTAATGCACAATG-
AAATAGGTGGTAGTGTCCATCTAAGGCTAAATATGACATAGAAACCGATAGTGAAGAA-----
GTAAAGTGATCGAAAATGGAATAGA
>DEB2
```

AGC-TTATAGTGGAGCGGTATACGGCTCAGTAACGGGCGATTTATTTGTTC-TCCTGGGA-------
CGGACAACATCGG-GAAACTGATGGGAAAACGTCTAAGTTGCAG------T-
TAATTGACTGTGACGTAAACC------------
TTTGTGTGCAGGAGAGTAAGATGCCATCCTATCAGTTAGTAAGTAGGGTAAGGGCCTACTTAGACGAAGACGG
GTACGGGGAATGAGGGTTTGATTCCG--
GAGAGGGAGCCTGAGAGATGGCTACCAGGACCAAGGTCAGCAGCAGGCGCGAAAATTATCGAAGCCCGCC-
TAGGGGCGATAGTGAGGAGACGTGTAT--AACGAAGTACGTGTAAAGAACGTGCTAATAACT---
GGAGGTCAAGTCTGGTGCCAGCATCCGCGGTAATTCCAGCTCCAGGGGTGTCTATGATGATTGCTGCGATTAA
AAAGTCCGTAGTCAATCTGACTG-ACTGACCTGCAATGTGAT-TG------
ATTAAAGAACGAGCAGGGTTAGGAAAGCAAAGAATTAGGAGCGACCGAGGGCTAGAGTATTGAATGGCGAGAG
GTGAAATTTGATGACCCAT-
TCAGGAGTGACAAAGGCGAAGGCACTAGTCAAGGGCGAATCCGATGATCAAGGACGTAGGCTAAAGTTTCGAA
AACGATT--AGAGACCGGAGTAGTTCTAGCAGTAAACTATGCCGACGCCGTGGTATC--AACAA------
TGGTATTGCGGAAGAGAAATC--AAGT-
AAGGCTTTGGGGAGAGTACGCGCGCAAGCGATAAATTTAAAGGAAATTGACGGAGGAACACCACAAGGAGTGG
AGTGTGCGGCTTAATTTGACTCAACGCGGGACAGCTTACCAGGCCCGATAATCGAGCGAGCGTAGTACGCGAT
AGGTTAAAGAGTGGTGCATGGCTGCTATCGACAGTTGGGGTGACC-TTAGGGTTAATTCCG-
GCAAGTAGTGAGACCCCTGCAGATAG---------TGGACAGGTATTTTT--AAAATACAGGAAGG-
AAGGGACAAGAGCA-GGTCAGTGATGCCCTTAGATGGCCTGGGCT----GCACGCGCACTACAGTGG-
TCATTATAAGTAGAAG-------TT-AGA-TATAAAGA-----------TGATCGAGAGGGA-
CTGAGCTTTG-TAAGAGGCTCACGAACGAGGAATT-
GCTAGTAATCGTAGGCTCATTAAGATACGATGAATATGTCCCTGTACCTT---
GTACACACCGCCCGTCGTTATCGAAGATGGAATTGTGTGC-GAACGAGC--
AACAAGCGAGTGAGCGCATAGTTCTAGATGTGATAAAAG-
TCGTAACAAGGCAACTGTAGGAGAACCTGTAGTTGGATCATACAGATTTTCTTA----ATAGAAGTTTG---
AGAAGTATT-----CCTTA--------CGCAAGGG---ATCGTTTGGTTCTA--
TGTACGATGAAGGTCGGAGCTGTATTCGATAAAATGAGATGATTGTATAAATTGTCTTATATTACTGAATAGA
GTA-GCGATGCTCGAATACTCCTTTGAATTAAGCATATGAGTAAAGGAAGGAAAAGGAACTAAC-
AAGGATTCTCTTATTAGTGGCGAATGAACAGAGAAGAGCCC-TAAGTGTAATCAAATTCTCTGAAT-------
-TTGAATTGTCAGGCAT--GGAGGAT-----
GCGAATATATTGGAAAATATAGCCATAGAGAGTTAAAGCCTCGTAGCA----
TCTTAAATTAGGGATGAGTAGTTGTGCTCGGTAATGCACAATG-
AAATAGGTGGTAGTGTCCATCTAAGGCTAAATATGACATAGAAACCGATAGTGAAGAA-----
GTAAAGTGATCGAAAATGGAATAGA
>GbOv2
AGC-GATAAGTGGAGCGGTGAAAGGCTCAGTAACGGGCGATTTATTTGATC-TCCTGGGA-------
CGGACAACATCGG-GAAACTGATGGGAAAACGTCTAAGTTGCAG------T-
TGTTTTATTGTGACGTAAACC------------
TTTGTGTGCAGGAGAGTAAGATGCCATCCTATCAGTTAGTAAGTAGGGTAAGGGCCTACTTAGACGAAGACGG
GTACGGGGAATGAGGGTTTGATTCCG--
GAGAGGGAGCCTGAGAGATGGCTACCAGGACCAAGGTCAGCAGCAGGCGCGAAAATTATCGAAGCCCGCC-
TAGGGGCGATAGTGAGGAGACGTGTAT---ACGAAGTACGTGTAAAGACCGTACTAATAACT---
GGAGGTCAAGTCTGGTGCCAGCATCCGCGGTAATTCCAGCTCCAGGGGTGTCTATGATGATTGCTGCGATTAA
AAAGTCCGTAGTCGAGCTGACTG-ACTGACCTGCAATGTGAT-TG------
ATTAAAGAACGAGCAGGGTTAGGAAAGCAGAGGATTAGGAGCGACCGAGGGCTAGAGTATTGAATGGCGAGAG
GTGAAATTTGATGACCCAT-
TCAGGAGTGACAAAGGCGAAGGCACTAGTCAAGGGCGAATCCGATGATCAAGGACGTAGGCTAGAGTTTCGAA
AACGATT--AGAGACCGGAGTAGTTCTAGCAGTAAACTATGCCGACACCGTGGTATT--AATTT------
TAGTATTGCGGAAGAGAAATC--AAGT-
AAGGCTTTGGGGAGAGTACGCGCGCAAGCGATAAATTTAAAGGAAATTGACGGAGGAACACCACAAGGAGTGG
AGTGTGCGGCTTAATTTGACTCAACGCGGGACAGCTTACCAGGCCCGATAATCGAGCGAGCGTAGTACGCGAT
AGGTTAAAAAGTGGTGCATGGCTGCTATCGACAGTTGGGGTGACC-TTAGGGTTAATTCCG-
GCAAGTAGTGAGACCCCTGCAGATAG---------TGGACAGGTATTTTT--AAGATACAGGAAGG-
AAGGGACAAGAGCA-GGTCAGTGATGCCCTTAGATGGCCTGGGCT----GCACGCGCACTACAGTGG-
TCATTATAAGTAGAAG-------TT-AGA-TATAAAGA-----------TGATCGAGAGGGA-
CTGAGCTTTG-TAAGAGGCTCACGAACGAGGAATT-
GCTAGTAATCGTAGGCTCATTAAGATACGATGAATATGTCCCTGTACCTT---
GTACACACCGCCCGTCGTTATCGAAGATGGAATTGTGTGC-GAACGAGC--

ATTAAGCGAGTGAGCGCATAGTTCTAGATGTGATAAAAG-
TCGTAACAAGGCAACTGTAGGAGAACCTGTAGTTGGATCATACAGATTGTTTAT----TTAAAAAGTTTTA-
ATTCTAATT-----CCTTA--------CGCAAGGG---ATCGTTTGGTTCTA--
TGTACGATGAAGGTCGAAACTGTATTCGATAAAATAGGATGAACGTAAAAAATGTCCTATATTACTGAATAGA
GTA-GCGATGCTCGAATACTCCTTTGAATTAAGCATATGAGTAAAGGAAGGAGAAGAAACTAAC-
AAGGATTCTCTTATTAGTGGCGAATGAACAGAGAACAGCCC-TAAGTGTAATCAAATTCATGTAAT-------
-TTGAGATGTCAGGCATT-GAATAGT-----
GTGAATATACTGGAAAGTATAGCCCAAGAGAGTTATAGCCTCGTAGCA----
CTATATGATAGGGATGAGTAGTTGTGCTCGGTAATGCACAATG-
AAATAGGTGGTAGTGTCCATCTAAGGCTAAATATGACATAGAAACCGATAGTGGAAAA-----
GTAAAGTGATCGAAAATGGAATAGA
>M60_Gbal
AGC-GATATGTGGAGCGGTGAAAGGCTCAGTAACGGGCGATTTATTTGATC-TCCTGGGA-------
CGGACAACATCGG-GAAACTGATGGGAAAACGTCTAAGTTGCAG------T-
TAAATGATTGTGACGTAAACC------------
ATAGTGTGCAGGAGAGTAAGATGCCATCCTATCAGTTAGTAAGTAGGGTAAGGGCCTACTTAGACGAAGACGG
GTACGGGGAATGAGGGTTTGATTCCG--
GAGAGGGAGCCTGAGAGATGGCTACCAGGACCAAGGTCAGCAGCAGGCGCGAAAATTATCGAAGCCCGCC-
TAGGGGCGATAGTGAGGAGACGTGTAT---ACGAAGTACGTGTAAAGACCGTACTAATAACT---
GGAGGTCAAGTCTGGTGCCAGCATCCGCGGTAATTCCAGCTCCAGGGGTGTCTATGATGATTGCTGCGATTAA
AAAGTCCGTAGTCGAGCTGACTG-ACTGACCTGCAATGTGAT-TG------
ATTAAAGAACGAGCAGGGTTAGGAAAGCAGAGGATTAGGAGCGACCGAGGGCTAGAGTATTGAATGGCGAGAG
GTGAAATTTGATGACCCAT-
TCAGGAGTGACAAAGGCGAAGGCACTAGTCAAGGGCGAATCCGATGATCAAGGACGTAGGCTAGAGTTTCGAA
AACGATT--AGAGACCGGAGTAGTTCTAGCAGTAAACTATGCCGACGCCGTGGTATA--GGCTA------
TAGTATTGCGGAAGAGAAATC--AAGT-
AAGGCTTTGGGGAGAGTACGCGCGCAAGCGATAAATTTAAAGGAAATTGACGGAGGAACACCACAAGGAGTGG
AGTGTGCGGCTTAATTTGACTCAACGCGGGACAGCTTACCAGGCCCGATAATCGAGCGAGCGTAGTACGCGAT
AGGTTAAAAAGTGGTGCATGGCTGCTATCGACAGTTGGGGTGACC-TTAGGGTTAATTCCG-
GCAAGTAGTGAGACCCCTGCAGATAG---------TGGACAGGTATTTTT--AAGATACAGGAAGG-
AAGGGACAAGAGCA-GGTCAGTGATGCCCTTAGATGGCCTGGGCT----GCACGCGCACTACAGTGG-
TCATTATAAGTAGAAG-------TT-AGA-TATAAAGA-----------TGATCGAGAGGGA-
CTGAGCTTTG-TAAGAGGCTCACGAACGAGGAATT-
GCTAGTAATCGTAGGCTCATTAAGATACGATGAATATGTCCCTGTACCTT---
GTACACACCGCCCGTCGTTATCGAAGATGGAATTGTGTGC-GAACGAGC--
ATTAAGCGAGTGAGCGCATAGTTCTAGATGTGATAAAAG-
TCGTAACAAGGCAACTGTAGGAGAACCTGTAGTTGGATCATACAGATTGTTTAT----TAATAGTTTTTA-
AAGTATATT-----CCTTA--------CGCAAGGG---ATCGTTTGGTTCTA--
TGTACGATGAAGGTCGAAACTGTATTCGATAAAATAGGATGAATGTAAAAATTGTCCTATGTTACTGAATAGA
GTA-GCGATGCTCGGGTACTCCTTTGAATTAAGCATATGAGTAAAGGAAGGAGAAGAAACTAAC-
AAGGATTCTCTTATTAGTGGCGAATGAACAGAGAAGAGCCC-TAAGTGTAATCAAATTCTTGCAAT-------
-TTGAGATGTCAGGCATT-AGGTAGT-----
GTGAATATACTGGAAAGTATAGCCGTAGAGAGTTATAGCCTCGTAGCA----
CTATATGATAGGGATGAGTAGTTGTGCTCGGTAATGCACAATG-
AAATAGGTGGTAGTGTCCATCTAAGGCTAAATATGACATAGAAACCGATAGTGGATAA-----
GTAAAGTGATCGAAAATGGAATAGA
>GbOv3
AGC-GATAAGTGGAGCGGTGAAAGGCTCAGTAACGGGCGATTTATTTGATC-TCCTGGGA-------
CGGACAACATCGG-GAAACTGATGGGAAAACGTCTAAGTTGCAG------T-
AAATTTATTGTGACGTAAACC------------
TTTGTGTGCAGGAGAGTAAGATGCCATCCTATCAGTTAGTAAGTAGGGTAAGGGCCTACTTAGACGAAGACGG
GTACGGGGAATGAGGGTTTGATTCCG--
GAGAGGGAGCCTGAGAGATGGCTACCAGGACCAAGGTCAGCAGCAGGCGCGAAAATTATCGAAGCCCGCC-
TAGGGGCGATAGTGAGGAGACGTGTAT---ACGAAGTACGTGTAAAGACCGTACTAATAACT---
GGAGGTCAAGTCTGGTGCCAGCATCCGCGGTAATTCCAGCTCCAGGGGTGTCTATGATGATTGCTGCGATTAA
AAAGTCCGTAGTCGAGCTGACTG-ACTGACCTGCAATGTGAT-TG------
ATTAAAGAACGAGCAGGGTTAGGAAAGCAGAGGATTAGGAGCGACCGAGGGCTAGAGTATTGAATGGCGAGAG
GTGAAATTTGATGACCCAT-

TCAGGAGTGACAAAGGCGAAGGCACTAGTCAAGGGCGAATCCGATGATCAAGGACGTAGGCTAGAGTTTCGAA
AACGATT--AGAGACCGGAGTAGTTCTAGCAGTAAACTATGCCGACACCGTGGTATT--AATTT------
TAGTATTGCGGAAGAGAAATC--AAGT-
AAGGCTTTGGGGAGAGTACGCGCGCAAGCGATAAATTTAAAGGAAATTGACGGAGGAACACCACAAGGAGTGG
AGTGTGCGGCTTAATTTGACTCAACGCGGGACAGCTTACCAGGCCCGATAATCGAGCGAGCGTAGTACGCGAT
AGGTTAAAAAGTGGTGCATGGCTGCTATCGACAGTTGGGGTGACC-TTAGGGTTAATTCCG-
GCAAGTAGTGAGACCCCTGCAGATAG---------TGGACAGGTATTTTT--AAGATACAGGAAGG-
AAGGGACAAGAGCA-GGTCAGTGATGCCCTTAGATGGCCTGGGCT----GCACGCGCACTACAGTGG-
TCATTATAAGTAGAAG-------TT-AGA-TATAAAGA-----------TGATCGAGAGGGA-
CTGAGCTTTG-TAAGAGGCTCACGAACGAGGAATT-
GCTAGTAATCGTAGGCTCATTAAGATACGATGAATATGTCCCTGTACCTT---
GTACACACCGCCCGTCGTTATCGAAGATGGAATTGTGTGC-GAACGAGC--
ATTAAGCGAGTGAGCGCATAGTTCTAGATGTGATAAAAG-
TCGTAACAAGGCAACTGTAGGAGAACCTGTAGTTGGATCATACAGATTGTTTAT----TTGAAAAGTTTTA-
ATTCTAATT-----CCTTA--------CGCAAGGG---ATCGTTTGGTTCTA--
TGTACGATGAAGGTCGAAACTGTATTCGATAAAATAGGATGAACGTAAAAAATGTCCTATATTACTGAATAGA
GTA-GCGATGCTCGAATACTCCTTTGAATTAAGCATATGAGTAAAGGAAGGAGAAGAAACTAAC-
AAGGATTCTCTTATTAGTGGCGAATGAACAGAGAACAGCCC-TAAGTGTAATCAAATTCATGTAAT-------
-TTGAGATGTCAGGCATT-GAATAGT-----
GTGAATATACTGGAAAGTATAGCCCAAGAGAGTTATAGCCTCGTAGCA----
CTATATGATAGGGATGAGTAGTTGTGCTCGGTAATGCACAATG-
AAATAGGTGGTAGTGTCCATCTAAGGCTAAATATGACATAGAAACCGATAGTGGAAAA-----
GTAAAGTGATCGAAAATGGAATAGA
>GFOS_GfOv
AGC-GATATGTGGAGCGGTGAAAGGCTCAGTAACGGGCGATTTATTTGATC-TCCTGGGA-------
CGGACAACATCGG-GAAACTGATGGGAAAACGTCTAAGTTGCAG------T-
TTTCTGATTGCGACGTAAACC------------
ATAGTGTGCAGGAGAGTAAGATGCCATCCTATCAGTTAGTAAGTAGGGTAAGGGCCTACTTAGACGAAGACGG
GTACGGGGAATGAGGGTTTGATTCCG--
GAGAGGGAGCCTGAGAGATGGCTACCAGGACCAAGGTCAGCAGCAGGCGCGAAAATTATCGAAGCCCGCC-
TAGGGGCGATAGTGAGGAGACGTGTAT---ACGAAGTACGTGTAAAGACCGTACTAATAACT---
GGAGGTCAAGTCTGGTGCCAGCATCCGCGGTAATTCCAGCTCCAGGGGTGTCTATGATGATTGCTGCGATTAA
AAAGTCCGTAGTCGAGCTGACTG-ACTGACCTGCAATGTGAT-TG------
ATTAAAGAACGAGCAGGGTTAGGAAAGCAGAGAATTAGGAGCGACCGAGGGCTAGAGTATTGAATGGCGAGAG
GTGAAATTTGATGACCCAT-
TCAGGAGTGACAAAGGCGAAGGCACTAGTCAAGGGCGAATCCGATGATCAAGGACGTAGGCTAGAGTTTCGAA
AACGATT--AGAGACCGGAGTAGTTCTAGCAGTAAACTATGCCGACGCCGTGGTAT----TTTT------
TAGTATTGCGGAAGAGAAATC--AAGT-
AAGGCTTTGGGGAGAGTACGCGCGCAAGCGATAAATTTAAAGGAAATTGACGGAGGAACACCACAAGGAGTGG
AGTGTGCGGCTTAATTTGACTCAACGCGGGACAGCTTACCAGGCCCGATAATCGAGCGAGCGTAGTACGCGAT
AGGTTAAAAAGTGGTGCATGGCTGCTATCGACAGTTGGGGTGACC-TTAGGGTTAATTCCG-
GCAAGTAGTGAGACCCCTGCAGATAG---------TGGACAGGTATTTTT--AAGATACAGGAAGG-
AAGGGACAAGAGCA-GGTCAGTGATGCCCTTAGATGGCCTGGGCT----GCACGCGCACTACAGTGG-
TCATTATAAGTAGAAG-------TT-AGA-TATAAAGA-----------TGATCGAGAGGGA-
CTGAGCTTTG-TAAGAGGCTCACGAACGAGGAATT-
GCTAGTAATCGTAGGCTCATTAAGATACGATGAATATGTCCCTGTACCTT---
GTACACACCGCCCGTCGTTATCGAAGATGGAATTGTGTGC-GAACGAGC--
ATTAAGCGAGTGAGCGCATAGTTCTAGATGTGATAAAAG-
TCGTAACAAGGCAACTGTAGGAGAACCTGTAGTTGGATCATACAGATTGTTTAT----CATAGTTTTTTT---
AGTATATT-----CCTTA--------CGCAAGGG---ATCGTTTGGTTCTA--
TGTACGATGAAGGTCGAAACTGTATTCGATAAAATAGGATGAAAGTAAAAAATTGTCCTATATTACTGAATAGA
GTA-GCGATGCTCGAGTACTCCTTTGAATTAAGCATATGAGTAAAGGAAGGAGAAGAAACTAAC-
AAGGATTCTCTTATTAGTGGCGAATGAACAGAGAAGAGCCC-TAAGTGTAATCAAATTCTTGTAAT-------
-TTGAGATGTCAGGCATT-AGATAGT-----
GTGAATATACTGGAAAGTATAGTCGTAGAGAGTTATAGCCTCGTAGCA----
CTATATGATAGGGATGAGTAGTTGTGCTCGGTAATGCACAATG-
AAATAGGTGGTAGTGTCCATCTAAGGCTAAATATGACATAGAAACCGATAGTGAATAA-----
GTAAAGTGATCGAAAATGGAATAGA

```
>GRR24
AGC-GATAAGTGGAGCGGTGAAAGGCTCAGTAACGGGCGATTTATTTGATC-TCCTGGGA-------
CGGACAACATCGG-GAAACTGATGGAAAACGTCTAAGTTGCAGTTT---T-
TTTTTGATTGCGACGTAAACC------------
TTTTGGTGCAGGAGAGTAAGATGCCATCCTATCAGTTAGTAAGTAGGGTAAGGGCCTACTTAGACGAAGACGG
GTACGGGGAATGAGGGTTTGATTCCG--
GAGAGGGAGCCTGAGAGATGGCTACCAGGACCAAGGTCAGCAGCAGGCGCGAAAATTATCGAAGCCCGCC-
TAGGGGCGATAGTGAGGAGACGTGTAT---ACGAAGTACGTGTAAAGACCGTACTAATAACT---
GGAGGTCAAGTCTGGTGCCAGCATCCGCGGTAATTCCAGCTCCAGGGGTGTCTATGATGATTGCTGCGATTAA
AAAGTCCGTAGTCGAGCTGACTG-ACTGACCTGCAATGTGGT-TG------
ATTAAAAGACGAGCAGGGTTAGGAAAGCAGAGAATTAGGAGCGACCGAGGGCTAGAGTATTGAATGGCGAGAG
GTGAAATTTGATGACCCAT-
TCAGGAGTGACAAAGGCGAAGGCACTAGTCAAGGGCGAATCCGATGATCAAGGACGTAGGCTAGAGTTTCGAA
AACGATT--AGAGACCGGAGTAGTTCTAGCAGTAAACTATGCCGACACCGTGGTATTAATTTTT------
TAGTATTGCGGAAGAGAAATC--AAGT-
AAGGCTTTGGGGAGAGTACGCGCGCAAGCGATAAATTTAAAGGAAATTGACGGAGGAACACCACAAGGAGTGG
AGTGTGCGGCTTAATTTGACTCAACGCGGGACAGCTTACCAGGCCCGATAATCGAGCGAGCGTAGTACGCGAT
AGATTAAAAAGTGGTGCATGGCTGCTATCGACAGTTGGGGTGACC-TTAGGGTTAATTCCG-
GCAAGTAGTGAGACCCCTGCAGATAG---------TGGACAGGTATTTTT--AAGATACAGGAAGG-
AAGGGACAAGAGCA-GGTCAGTGATGCCCTTAGATGGCCTGGGCT----GCACGCGCACTACAGTGG-
TCATTATAAGTAGAAA-------TT-AGA-TATAAAGA-----------TGATCGAGAGGGA-
CTGAGCTTTG-TAAGAGGCTCACGAACGAGGAATT-
GCTAGTAATCGTAGGCTCATTAAGATACGATGAATATGTCCCTGTACCTT---
GTACACACCGCCCGTCGTTATCGAAGATGGAATTGTGTGC-GAACGAGC--
ATTAAGCGAGTGAGCGCATAGTTCTAGATGTGATAAAAG-
TCGTAACAAGGCAACTGTAGGAGAACCTGTAGTTGGATCATACAGATTGTTTAA----TAGAAAGTTTTT--
AATTTAATT-----CCTTA--------CGCAAGGG---ATCGTTTGGTTCTA--
TGTACGATGAAGGTCGACACTGTATTCGATAAAATAAGATGAAAGTAAAAATTGTCTTATATTACTGAATAGA
GTA-GCGATGCTCGAATACTCCTTTGAATTAAGCATATGAGTAAAGGAAGGAGAAGAAACTAAC-
AAGGATTCTCTTATTAGTGGCGAATGAACAGAGAATAGCCC-TAAGTGTAATCAAATTCTAGCAAT-------
-TTGAGATGTCAGGCATT-GAATAGT-----
GTGAATATACTGGAAAGTATAGCCAAAGAGAGTTATAGCCTCGTAACA----
CTATATGATAGGGATGAGTAGTTGTGCTCGGTAATGCACAATG-
AAATAGGTGGTAGTGTCCATCTAAGGCTAAATATGACATAGAGACCGATAGTGAATAA-----
GTAAAGTGATCGAAAATGGAATAGA
>M41_Gvar
AGC-GATATGTGGAGCGGTGAAAGGCTCAGTAACGGGCGATTTATTTGATC-TCCTGGGA-------
CGGACAACATCGG-GAAACTGATGGAAAACGTCTAAGTTGCAG------T-
TTTTTGACTGTGACGTAAACC------------
GTAGTGTGCAGGAGAGTAAGATGCCATCCTATCAGTTAGTAAGTAGGGTAAGGGCCTACTTAGACGAAGACGG
GTACGGGGAATGAGGGTTTGATTCCG--
GAGAGGGAGCCTGAGAGATGGCTACCAGGACCAAGGTCAGCAGCAGGCGCGAAAATTATCGAAGCCCGCC-
TAGGGGCGATAGTGAGGAGACGTGTTA---ACGAAGTACGTGTAAAGACCGTACTAATAACT---
GGAGGTCAAGTCTGGTGCCAGCATCCGCGGTAATTCCAGCTCCAGGGGTGTCTATGATGATTGCTGCGATTAA
AAAGTCCGTAGTCGAGCTGACTG-ACTGACCTGCAATGTGAT-TG------
ATTAAAGAACGAGCAGGGTTAGGAAAGCAGAGGATTAGGAGCGACCGAGGGCTAGAGTATTGAATGGCGAGAG
GTGAAATTTGATGACCCAT-
TCAGGAGTGACAAAGGCGAAGGCACTAGTCAAGGGCGAATCCGATGATCAAGGACGTAGGCTAGAGTTTCGAA
AACGATT--AGAGACCGGAGTAGTTCTAGCAGTAAACTATGCCGACGCCGTGGTATT-AGGTAT------
TAGTATTGCGGAAGAGAAATC--AAGT-
AAGGCTTTGGGGAGAGTACGCGCGCAAGCGATAAATTTAAAGGAAATTGACGGAGGAACACCACAAGGAGTGG
AGTGTGCGGCTTAATTTGACTCAACGCGGGACAGCTTACCAGGCCCGAT-
ATCGAGCGAGCGTAGTACGCGATAGGTTAAGAAGTGGTGCATGGCTGCTATCGACAGTTGGGGTGACC-
TTAGGGTTAATTCCG-GCAAGTAGTGAGACCCCTGCAGATAG---------TGGACAGGTATTTTC--
AAGATACAGGAAGG-AAGGGACAAGAGCA-GGTCAGTGATGCCCTTAGATGGCCTGGGCT----
GCACGCGCACTACAGTGG-TCATTATAAGTAGAAG-------TT-AGA-TATAAAGA-----------
TGATCGAGAGGGA-CTGAGCTTTG-TAAGAGGCTCACGAACGAGGAATT-
GCTAGTAATCGTAGGCTCATTAAGATACGATGAATATGTCCCTGTACCTT---
```

GTACACACCGCCCGTCGTTATCGAAGATGGAATTGTGTGC-GAACAAGC--
ATTAAGCGAGTGAGCGCATAGTTCTAGATGTGATAAAAG-
TCGTAACAAGGCAACTGTAGGAGAACCTGTAGTTGGATCATACAGATTGTTTTT----
TAACAGTTTTATTTATAAATATT-----CCTTA--------CGCAAGGG---ATCGTTTGGTTCTA--
TGTACGATGAAGGTCGAAACTGTATTCGATAAAATAGGATGAAAGTAAAAATTGTCCTATGTTACTGAATAGA
GTA-GCGATGCTCGAGTACTCCTTTGAATTAAGCATATGAGTAAAGGAAGGAGAAGAAACTAAC-
AAGGATTCTCTTATTAGTGGCGAATGAACAGAGAAGAGCCC-TAAGTGTAATCAAATTCTTGCAAT-------
-TTGAGATGTCAGGCATT-AGATAGT-----
GTGAATATACTGGAAAGTATTGCCGTAGAGAGTTATAGCCTCGTAGCA----
CTATATGATAGGGATGAGTAGTTGTGCTCGGTAATGCACAATG-
AAATAGGTGGTAGTGTCCATCTAAGGCTAAATATGACATAGAAACCGATAGTGGATAA-----
GTAAAGTGATCGAAAATGGAATAGA
>GVAR_M40
AGC-GATATGTGGAGCGGTGAAAGGCTCAGTAACGGGCGATTTATTTGATC-TCCTGGGA-------
CGGACAACATCGG-GAAACTGATGGGAAAACGTCTAAGTTGCAG------T-
TTTTTGACTGTGACGTAAACC------------
GTAGTGTGCAGGAGAGTAAGATGCCATCCTATCAGTTAGTAAGTAGGGTAAGGGCCTACTTAGACGAAGACGG
GTACGGGGAATGAGGGTTTGATTCCG--
GAGAGGGAGCCTGAGAGATGGCTACCAGGACCAAGGTCAGCAGCAGGCGCGAAAATTATCGAAGCCCGCC-
TAGGGGCGATAGTGAGGAGACGTGTTA---ACGAAGTACGTGTAAAGACCGTACTAATAACT---
GGAGGTCAAGTCTGGTGCCAGCATCCGCGGTAATTCCAGCTCCAGGGGTGTCTATGATGATTGCTGCGATTAA
AAAGTCCGTAGTCGAGCTGACTG-ACTGACCTGCAATGTGAT-TG------
ATTAAAGAACGAGCAGGGTTAGGAAAGCAGAGGATTAGGAGCGACCGAGGGCTAGAGTATTGAATGGCGAGAG
GTGAAATTTGATGACCCAT-
TCAGGAGTGACAAAGGCGAAGGCACTAGTCAAGGGCGAATCCGATGATCAAGGACGTAGGCTAGAGTTTCGAA
AACGATT--AGAGACCGGAGTAGTTCTAGCAGTAAACTATGCCGACGCCGTGGTATT-AGGTAT------
TAGTATTGCGGAAGAGAAATC--
AAGTAAAGGCTTTGGGGAGAGTACGCGCGCAAGCGATAAATTTAAAGGAAATTGACGGAGGAACACCACAAGG
AGTGGAGTGTGCGGCTTAATTTGACTCAACGCGGGACAGCTTACCAGGCCCGATAATCGAGCGAGCGTAGTAC
GCGATAGGTTAAGAAGTGGTGCATGGCTGCTATCGACAGTTGGGGTGACC-TTAGGGTTAATTCCG-
GCAAGTAGTGAGACCCCTGCAGATAG---------TGGACAGGTATTTTC--AAGATACAGGAAGG-
AAGGGACAAGAGCA-GGTCAGTGATGCCCTTAGATGGCCTGGGCT----GCACGCGCACTACAGTGG-
TCATTATAAGTAGAAG-------TT-AGA-TATAAAGA-----------TGATCGAGAGGGA-
CTGAGCTTTG-TAAGAGGCTCACGAACGAGGAATT-
GCTAGTAATCGTAGGCTCATTAAGATACGATGAATATGTCCCTGTACCTT---
GTACACACCGCCCGTCGTTATCGAAGATGGAATTGTGTGC-GAACAAGC--
ATTAAGCGAGTGAGCGCATAGTTCTAGATGTGATAAAAG-
TCGTAACAAGGCAACTGTAGGAGAACCTGTAGTTGGATCATACAGATTGTTTTT----
TAACAGTTTTATTTATAAATATT-----CCTTA--------CGCAAGGG---ATCGTTTGGTTCTA--
TGTACGATGAAGGTCGAAACTGTATTCGATAAAATAGGATGAAAGTAAAAATTGTCCTATGTTACTGAATAGA
GTA-GCGATGCTCGAGTACTCCTTTGAATTAAGCATATGAGTAAAGGAAGGAGAAGAAACTAAC-
AAGGATTCTCTTATTAGTGGCGAATGAACAGAGAAGAGCCC-TAAGTGTAATCAAATTCTTGCAAT-------
-TTGAGATGTCAGGCATT-AGATAGT-----
GTGAATATACTGGAAAGTATTGCCGTAGAGAGTTATAGCCTCGTAGCA----
CTATATGATAGGGATGAGTAGTTGTGCTCGGTAATGCACAATG-
AAATAGGTGGTAGTGTCCATCTAAGGCTAAATATGACATAGAAACCGATAGTGGATAA-----
GTAAAGTGATCGAAAATGGAATAGA
>EISC_PLZWC12
CGT-TTAACGTGGAGCGGTGAAAGGCTCAGTAACGGGCGATTTATTTGATC-TCCTGGGA-------
CGGACAACATCGG-GAAACTGATGGGAAAACGTCTAAGTTGCAG------T-
TGTATGATTGTGACGTAAACC-------------
TTTGGTGCAGGAGAGTAAGATGCCATCCTATCAGTTAGTAAGTAGGGTAAGGGCCTACTTAGACGAAGACGGG
TACGGGGAATGAGGGTTTGATTCCG--
GAGAGGGAGCCTGAGAGACGGCTACCAGGACCAAGGTCAGCAGCAGGCGCGAAAATTATCGAAGCCCGCC-
GAGGGGCGATAGTGAGGAGACGTGTAT--AACGAAGTACGTGTAAAGAACGTGCTAACAACT---
GGAGGTCAAGTCTGGTGCCAGCATCCGCGGTAATTCCAGCTCCAGGGGTGTCTATGATGATTGCTGCGATTAA
AAAGTCCGTAGTCAATCTGTCTG-ACTTGCCTGCAATGTGAT-TG------
ATTAAAGAACGAGCAGGGCTAGGAAAGCAGAGAATTAGGAGCGACCGAGGGCTAGAGTATTGAATGGCGAGAG

GTGAAATTTGATGACCCAT-
TCAGGAGTGACAAAGGCGAAGGCACTAGTCAAGGGCGAATCCGATGATCAAGGACGTAGGCTAGAGTTTCGAA
AACGATT--AGAGACCGGAGTAGTTCTAGCAGTAAACTATGCCGACGCCGTGGTATG--TATTT------
ATGTATTGCGGAAGAGAAATC--AAGT-
AAGGCTTTGGGGAGAGTACGCGCGCAAGCGATAAATTTAAAGGAAATTGACGGAGGAACACCACAAGGAGTGG
AGTGTGCGGCTTAATTTGACTCAACGCGGGACAGCTTACCAGGCCCGATAATCGAGCGAGCGTAGTACGCGAT
AGGTTAAAGAGTGGTGCATGGCTGCTATCGACAGTTGGGGTGACC-TTAGGGTTAATTCCG-
GCAAGTAGTGAGACCCCTGCAGATAG---------TGGACAGGTATTTTA--AAAATACAGGAAGG-
AAGGGACAAGAGCA-GGTCAGTGATGCCCTTAGATGGCCTGGGCT----GCACGCGCACTACAGTGG-
TCATTATAAGACGAAA-------GT-AGA-TATAAAGA-----------TGATCGAGAGGGA-
CTGAGCTTTG-TAAGAGGCTCACGAACGAGGAATT-
GCTAGTAATCGTAGGCTCATTAAGATACGATGAATATGTCCCTGTACCTT---
GTACACACCGCCCGTCGTTATCGAAGATGGAATTGTGTGC-GAACGAGC--
AATAAGCGAGTGAGCGCATAGTTCTAGATGTGATAAAAG-
TCGTAACAAGGCAACTGTAGGAGAACCTGTAGTTGGATCATACAGATATTGTCA----GCAAGAGTTT----
TATAGTATT-----CCTTA--------CGCAAGGG---ATCGTTTGGTTCTA--
TGTTCGAAGAAGGTCGGAGCAGTATTCGATAATGTAAGATGATTGTAAAAGTTGTCTTATGTGACTGAATTGA
GTACAAAATGCTCGGGTACTCCTTTGAATTAAGCATATGAGTAAAGGAAGGAAAAGAAACTAAC-
AAGGATTCTCTTATTAGTGGCGAATGAACAGAGAAGAGCCC-TAAGTGTAATCAAATTCTTGTAAT-------
-TTGAGATGTCTAGTATT-ATACAGT-----
GTGAATATGCTGGAAAGCATTGCCGTAGAGAGTTATAGCCTCGTAGCA----CTA-
GTTATAGGGATGAGTAGTTGTGCTCGGTAATGCACAATG-
AAATAGGTGGTAGTGTCCATCTAAGGCTAAATATGACATAGAAACCGATAGTGAATAA-----
GTAAAGTGATCGAAAATGGAATAGA
>EISC_PLZWC18
CGT-TTAACGTGGAGCGGTGAAAGGCTCAGTAACGGGCGATTTATTTGATC-TCCTGGGA-------
CGGACAACATCGG-GAAACTGATGGGAAAACGTCTAAGTTGCAG------T-
TGTATGATTGTGACGTAAACC-------------
TTTGGTGCAGGAGAGTAAGATGCCATCCTATCAGTTAGTAAGTAGGGTAAGGGCCTACTTAGACGAAGACGGG
TACGGGGAATGAGGGTTTGATTCCG--
GAGAGGGAGCCTGAGAGACGGCTACCAGGACCAAGGTCAGCAGCAGGCGCGAAAATTATCGAAGCCCGCC-
GAGGGGCGATAGTGAGGAGACGTGTAT--AACGAAGTACGTGTAAAGAACGTGCTAACAACT---
GGAGGTCAAGTCTGGTGCCAGCATCCGCGGTAATTCCAGCTCCAGGGGTGTCTATGATGATTGCTGCGATTAA
AAAGTCCGTAGTCAATCTGTCTG-ACTTGCCTGCAATGTGAT-TG------
ATTAAAGAACGAGCAGGGCTAGGAAAGCAGAGAATTAGGAGCGACCGAGGGCTAGAGTATTGAATGGCGAGAG
GTGAAATTTGATGACCCAT-
TCAGGAGTGACAAAGGCGAAGGCACTAGTCAAGGGCGAATCCGATGATCAAGGACGTAGGCTAGAGTTTCGAA
AACGATT--AGAGACCGGAGTAGTTCTAGCAGTAAACTATGCCGACGCCGTGGTATG--TATTT------
ATGTATTGCGGAAGAGAAATC--AAGT-
AAGGCTTTGGGGAGAGTACGCGCGCAAGCGATAAATTTAAAGGAAATTGACGGAGGAACACCACAAGGAGTGG
AGTGTGCGGCTTAATTTGACTCAACGCGGGACAGCTTACCAGGCCCGATAATCGAGCGAGCGTAGTACGCGAT
AGGTTAAAGAGTGGTGCATGGCTGCTATCGACAGTTGGGGTGACC-TTAGGGTTAATTCCG-
GCAAGTAGTGAGACCCCTGCAGATAG---------TGGACAGGTATTTTA--AAAATACAGGAAGG-
AAGGGACAAGAGCA-GGTCAGTGATGCCCTTAGATGGCCTGGGCT----GCACGCGCACTACAGTGG-
TCATTATAAGACGAAA-------GT-AGA-TATAAAGA-----------TGATCGAGAGGGA-
CTGAGCTTTG-TAAGAGGCTCACGAACGAGGAATT-
GCTAGTAATCGTAGGCTCATTAAGATACGATGAATATGTCCCTGTACCTT---
GTACACACCGCCCGTCGTTATCGAAGATGGAATTGTGTGC-GAACGAGC--
AATAAGCGAGTGAGCGCATAGTTCTAGATGTGATAAAAG-
TCGTAACAAGGCAACTGTAGGAGAACCTGTAGTTGGATCATACAGATATTGTCA----GCAAGAGTTT----
TATAGTATT-----CCTTA--------CGCAAGGG---ATCGTTTGGTTCTA--
TGTTCGAAGAAGGTCGGAGCAGTATTCGATAATGTAAGATGATTGTAAAAGTTGTCTTATGTGACTGAATTGA
GTACAAAATGCTCGGGTACTCCTTTGAATTAAGCATATGAGTAAAGGAAGGAAAAGAAACTAAC-
AAGGATTCTCTTATTAGTGGCGAATGAACAGAGAAGAGCCC-TAAGTGTAATCAAATTCTTGTAAT-------
-TTGAGATGTCTAGTATT-ATATAGT-----
GTGAATATGCTGGAAAGCATTGCCGTAGAGAGTTATAGCCTCGTAGCA----CTA-
GTTATAGGGATGAGTAGTTGTGCTCGGTAATGCACAATG-

AAATAGGTGGTAGTGTCCATCTAAGGCTAAATATGACATAGAAACCGATAGTGAATAA-----
GTAAAGTGATCGAAAATGGAATAGA
>EISC_PLZWC19
CGT-TTAACGTGGAGCGGTGAAAGGCTCAGTAACGGGCGATTTATTTGATC-TCCTGGGA-------
CGGACAACATCGG-GAAACTGATGGGAAAACGTCTAAGTTGCAG------T-
TGTATGATTGTGACGTAAACC-------------
TTTGGTGCAGGAGAGTAAGATGCCATCCTATCAGTTAGTAAGTAGGGTAAGGGCCTACTTAGACGAAGACGGG
TACGGGGAATGAGGGTTTGATTCCG--
GAGAGGGAGCCTGAGAGACGGCTACCAGGACCAAGGTCAGCAGCAGGCGCGAAAATTATCGAAGCCCGCC-
GAGGGGCGATAGTGAGGAGACGTGTAT--AACGAAGTACGTGTAAAGAACGTGCTAACAACT---
GGAGGTCAAGTCTGGTGCCAGCATCCGCGGTAATTCCAGCTCCAGGGGTGTCTATGATGATTGCTGCGATTAA
AAAGTCCGTAGTCAATCTGTCTG-ACTTGCCTGCAATGTGAT-TG------
ATTAAAGAACGAGCAGGGCTAGGAAAGCAGAGAATTAGGAGCGACCGAGGGCTAGAGTATTGAATGGCGAGAG
GTGAAATTTGATGACCCAT-
TCAGGAGTGACAAAGGCGAAGGCACTAGTCAAGGGCGAATCCGATGATCAAGGACGTAGGCTAGAGTTTCGAA
AACGATT--AGAGACCGGAGTAGTTCTAGCAGTAAACTATGCCGACGCCGTGGTATG--TATTT------
ATGTATTGCGGAAGAGAAATC--AAGT-
AAGGCTTTGGGGAGAGTACGCGCGCAAGCGATAAATTTAAAGGAAATTGACGGAGGAACACCACAAGGAGTGG
AGTGTGCGGCTTAATTTGACTCAACGCGGGACAGCTTACCAGGCCCGATAATCGAGCGAGCGTAGTACGCGAT
AGGTTAAAGAGTGGTGCATGGCTGCTATCGACAGTTGGGGTGACC-TTAGGGTTAATTCCG-
GCAAGTAGTGAGACCCCTGCAGATAG---------TGGACAGGTATTTTA--AAAATACAGGAAGG-
AAGGGACAAGAGCA-GGTCAGTGATGCCCTTAGATGGCCTGGGCT----GCACGCGCACTACAGTGG-
TCATTATAAGACGAAA-------GT-AGA-TATAAAGA-----------TGATCGAGAGGGA-
CTGAGCTTTG-TAAGAGGCTCACGAACGAGGAATT-
GCTAGTAATCGTAGGCTCATTAAGATACGATGAATATGTCCCTGTACCTT---
GTACACACCGCCCGTCGTTATCGAAGATGGAATTGTGTGC-GAACGAGC--
AATAAGCGAGTGAGCGCATAGTTCTAGATGTGATAAAAG-
TCGTAACAAGGCAACTGTAGGAGAACCTGTAGTTGGATCATACAGATATTGTCA----GCAAGAGTTT----
TATAGTATT-----CCTTA--------CGCAAGGG---ATCGTTTGGTTCTA--
TGTTCGAAGAAGGTCGGAGCAGTATTCGATAATGTAAGATGATTGTAAAAGTTGTCTTATGTGACTGAATTGA
GTACAAAATGCTCGGGTACTCCTTTGAATTAAGCATATGAGTAAAGGAAGGAAAAGAAACTAAC-
AAGGATTCTCTTATTAGTGGCGAATGAACAGAGAAGAGCCC-TAAGTGTAATCAAATTCTTGTAAT-------
-TTGAGATGTCAGGTATT-ATATAGT-----
GTGAATATGCTGGAAAGCATTGCCGTAGAGAGTTATAGCCTCGTAGCA----CTA-
GTTATAGGGATGAGTAGTTGTGCTCGGTAATGCACAATG-
AAATAGGTGGTAGTGTCCATCTAAGGCTAAATATGACATAGAAACCGATAGTGAATAA-----
GTAAAGTGATCGAAAATGGAATAGA
>M56_Gvar
AGC-TATATGTGGAGCGGTGAAAGGCTCAGTAACGGGCGATTTATTTAATC-TCCTGGGA-------
CGGACAACATCGG-GAAACTGATGGGAAAACGTCTAAGTTGCATTAA---T----
GTTAGTGTGACGTAAACG------------
ATATCGTGCAGGAGAGTAAGATGCCATCCTATCAGTTAGTAAGTAGGGTAAGGGCCTACTTAGACGAAGACGG
GTACGGGGAATGAGGGTTTGATTCCG--
GAGAGGGAGCCTGAGAGATGGCTACCAGGACCAAGGTCAGCAGCAGGCGCGAAAATTATCGAAGCCCGCC-
TAGGGGCGATAGTGAGGAGACGTGTAT--AACGAAGTACGTGTAAAGAACGTACTAATAACT---
GGAGGTCAAGTCTGGTGCCAGCATCCGCGGTAATTCCAGCTCCAGGGGTGTCTATGATGATTGCTGCGATTAA
AAAGTCCGTAGTCAAGCTGACTG-ACTGACCTGCAATGTGAT-TG------
ATTAAGGAACGAGCAGGGTTAGGAAAGCAGAGAATTAGGAGCGACCGAGGGCTAGAGTATTGAATGGCGAGAG
GTGAAATTTGATGACCCAT-
TCAGGAGTGACAAAGGCGAAGGCACTAGTCAAGGGCGAATCCGATGATCAAGGACGTAGGCTAGAGTTTCGAA
AACGATT--AGAGACCGGAGTAGTTCTAGCAGTAAACTATGCCGACGCCGTGATATA--GTTT------
GTGGTATTGCGGAAGAGAAATC--AAGT-
AAGGCTTTGGGGAGAGTACGCGCGCAAGCGATAAATTTAAAGGAAATTGACGGAGGAACACCACAAGGAGTGG
AGTGTGCGGCTTAATTTGACTCAACGCGGGACAGCTTACCAGGCCCGATAATCGAGCGAGCGTTGTACGCGAT
AGATTAAAGAGTGGTGCATGGCTGCTATCGACAGTTGGGGTGACC-TTAGGGTTAATTCCG-
GCAAGTAGTGAGACCCCTGCAGATAG---------TGGACAGGTATTTTT--AAAATACAGGAAGG-
AAGGGACAAGAGCA-GGTCAGTGATGCCCTTAGATGGCCTGGGCT----GCACGCGCACTACAGTGG-
TCATTATAAGTAGAAG-------TT-AGA-AGTAAAGA-----------TGATCGAGAGGGA-

CTGAGCTTTG-TAAGAGGCTCACGAACGAGGAATT-
GCTAGTAATCGTAGGCTCATTAAGATACGATGAATATGTCCCTGTACCTT---
GTACACACCGCCCGTCGTTATCGAAGATGGAATTGTGTGC-GAACGAGC--
AACAAGCGAGTGAGCGCATAGTTCTAGATGTGATAAAAG-
TCGTAACAAGGCAACTGTAGGAGAACCTGTAGTTGGATCATACAGATTTAATT-----TAAGTAGTTTT---
TAATAAATT-----CCTTA--------CGCAAGGG---ATCGTTTGGCTCTA--
TGTGCGATGAAGGTCGGAACAGTATTCGATAATATAAGATGATTGTATAAATTGTCTTATGTTTCTGAATAGA
GTA-GTGATGCTCGATTACTCCTTTGAATTAAGCATATGAGTAAAGGAAGGAGAAGAAACTAAC-
AAGGATTCTCTTATTAGTGGCGAATGAACAGAGAAGAGCCC-TAAGTGTAATCAAATTCTAAGAAT-------
-TTGAGATGTCAGGTATT-ATGTGGT-----
GTGAATGTACTGGAAAGTACTGCCGAAGAGAGTTATAGCCTCGTAACA----
CCATTTTGTAGGTATGAGTAGTTGTGCTCGGTAATGCACAATG-
AAATAGGTGGTAGTGTCCATCTAAGGCTAAATATGACATAGAGACCGATAGTGAATAA-----
GTAAAGTGATCGAAAATGGAATAGA
>GVAR_MB7
AGC-TATATGTGGAGCGGTGAAAGGCTCAGTAACGGGCGATTTATTTAATC-TCCTGGGG-------
CGGACAACATCGG-GAAACTGATGGGAAAACGTCTAAGTTGCATTAA---T----
TTTAGTGTGACGTAAACG-----------
ATTATCGTGCAGGAGAGTAAGATGCCATCCTATCAGTTAGTAAGTAGGGTAAGGGCCTACTTAGACGAAGACG
GGTACGGGGAATGAGGGTTTGATTCCG--
GAGAGGGAGCCTGAGAGATGGCTACCAGGACCAAGGTCAGCAGCAGGCGCGAAAATTATCGAAGCCCGCC-
TAGGGGCGATAGTGAGGAGACGTGTAT--AACGAAGTACGTGTAAAGAACGTACTAATAACT---
GGAGGTCAAGTCTGGTGCCAGCATCCGCGGTAATTCCAGCTCCAGGGGTGTCTATGATGATTGCTGCGATTAA
AAAGTCCGTAGTCAAGCTGCCTG-ACTGACCTGCAATGTGAT-TG------
ATTAAGGAACGAGCAGGGTTAGGAAAGCAGAGAATTAGGAGCGACCGAGGGCTAGAGTATTGAATGGCGAGAG
GTGAAATTTGATGACCCAT-
TCAGGAGTGACAAAGGCGAAGGCACTAGTCAAGGGCGAATCCGATGATCAAGGACGTAGGCTAGAGTTTCGAA
AACGATT--AGAGACCGGAGTAGTTCTAGCAGTAAACTATGCCGACGCCGTGGTATG--
GTTTTTTGTGGCTGTATTGCGGAAGAGAAATC--AAGT-
AAGGCTTTGGGGAGAGTACGCGCGCAAGCGATAAATTTAAAGGAAATTGACGGAGGAACACCACAAGGAGTGG
AGTGTGCGGCTTAATTTGACTCAACGCGGGACAGCTTACCAGGCCCGATAATCGAGCGAGCGTTGTACGCGAT
AGATTAAAGAGTGGTGCATGGCTGCTATCGACAGTTGGGGTGACC-TTAGGGTTAATTCCG-
GCAAGTAGTGAGACCCCTGCAGATAG---------TGGACAGGTATTTTT--AAAATACAGGAAGG-
AAGGGACAAGAGCA-GGTCAGTGATGCCCTTAGATGGCCTGGGCT----GCACGCGCACTACAGTGG-
TCATTATAAGTAGAAG-------TT-AGA-AGTAAAGA-----------TGATCGAGAGGGA-
CTGAGCTTTG-TAAGAGGCTCACGAACGAGGAATT-
GCTAGTAATCGTAGGCTCATTAAGATACGATGAATATGTCCCTGTACCTT---
GTACACACCGCCCGTCGTTATCGAAGATGGAATTGTGTGC-GAACGAGC--
AACAAGCGAGTGAGCGCATAGTTCTAGATGTGATAAAAG-
TCGTAACAAGGCAACTGTAGGAGAACCTGCAGTTGGATCATACAGATTTATTT-----TAGGTAGTTTA---
TAAAAAATT-----CCTTA--------CGCAAGGG---ATCGTTTGGCTCTA--
TGTGCGATGAAGGTCGGAGCAGTATTCGATAATATAAGATGATTGTATAAATTGTCTTATGTTACTGAATAGA
GTA-GTGATGCTCGATTACTCCTTTGAATTAAGCATATGAGTAAAGGAAGGAAAAGAAACCAAC-
GAGGATTCTCTTATTAGTGGCGAATGAACAGAGAACAGCCC-TAAGTGTAATCAAATTCGTTGAAT-------
-TTGAGATGTCAGGTATC-ATGTGGT-----
GTGAATGTACTGGAAAGTACTGCCGAAGAGAGTTATAGCCTCGTAGCA----
CCATTTTTTAGGTATGAGTAGTTGTGCTCGGTAATGCACAATG-
AAATAGGTGGTAGTGTCCATCTAAGGCTAAATATGACATAGAGACCGATAGTAAATAA-----
GTAAAGTGATCGAAAATGGAATAGA
>PrOv1
AGC-TATATGTGGAGCGGTGAAAGGCTCAGTAACGGGCGATTTATTTAATC-TCCTGGGA-------
CGGACAACATCGG-GAAACTGATGGGAAAACGTCTAAGTTGCAG------T-
TTTGTTATTGCGACGTAAACG-----------
GTAATCGTGCAGGAGAGTAAGATGCCATCCTATCAGTTAGTAAGTAGGGTAAGGGCCTACTTAGACGAAGACG
GGTACGGGGAATGAGGGTTTGATTCCG--
GAGAGGGAGCCTGAGAGATGGCTACCAGGACCAAGGTCAGCAGCAGGCGCGAAAATTATCGAAGCCCGCC-
TAGGGGCGATAGTGAGGAGACGTGTAT---TCGAAGTACGTGTAAAGAACGTACTAATAACT---
GGAGGTCAAGTCTGGTGCCAGCATCCGCGGTAATTCCAGCTCCAGGGGTGTCTATGATGATTGCTGCGATTAA

AAAGTCCGTAGTCAAGCTGACTG-ACTGACCTGCAATGTGAT-TG------
ATTAAAGAACGAGCAGGGTTAGGAAAGCAGAGAATTAGGAGCGACCGAGGGCTAGAGTATTGAATGGCGAGAG
GTGAAATTTGATGACCCAT-
TCAGGAGTGACAAAGGCGAAGGCACTAGTCAAGGGCGAATCCGATGATCAAGGACGTAGGCTAGAGTTTCGAA
AACGATT--AGAGACCGGAGTAGTTCTAGCAGTAAACTATGCCGACGCCGTGATATT--
GTTTTTTGTGGCGGTATTGCGGAAGAGAAATC--AAGT-
AAAGCTTTGGGGAGAGTACGCGCGCAAGCGATAAATTTAAAGGAAATTGACGGAGG-
ACACCACAAGGAGTGGAGTGTGCGGCTTAATTTGACTCAACGCGGGACAGCTTACCA-
GCCCGATAATCGAGCGAGCGTAGTACGCGATAGATTAAAGAGTGGTGCATGGCTGCTATCGACAGTTGGGGTG
ACC-TTAGGGTTAATTCCG-GCAAGTAGTGAGACCCCTGCAGATAG---------TGGACAGGTATTTTT-
CAAAATACAGGAAGG-AAGGGACAAGAGCA-GGTCAGTGATGCCCTTAGATGGCCTGGGCT----
GCACGCGCACTACAGTGG-TCATTATAAGTAGAAG-------TT-AGA-TATAAAGA-----------
TGATCGAGAGGGA-CTGAGCTTTG-TAAGAGGCTCACGAACGAGGAATT-
GCTAGTAATCGTAGGCTCATTAAGATACGATGAATATGTCCCTGTACCTT---
GTACACACCGCCCGTCGTTATCGAAGATGGAATTGTGTGC-GAACGAGC--
AACAAGCGAGTGAGCGCATAGTTCTAGATGTGATAAAAG-
TCGTAACAAGGCAACTGTAGGAGAACCTGTAGTTGGATCATACAGATTTATTTT----TAAGTAGTTTT---
TTATAATTT-----CCTTA--------CGCAAGGG---ATCGTTTGGTTCTA--
TGTACGATGAAGGTCGAAGCAGTATTCGATAATGTAAGATGGTTGTATAAATTGTCTTATGTTACTGAATAGA
GTA-GTGATGCTCGAATACTCCTTTGAATTAAGCATATGAGTAAAGGAAGGAGAAGAAACTAAC-
AAGGATTCTCTTATTAGTGGCGAATGAACAGAGAATAGCCC-TAAGTGTAATCAAATTCATTGAAT-------
-TTGAGATGTCAGGTATC-ATGTAGT-----
GTGAATATACTGGAAAGTATTGCCAGAGAGAGTTATAGCCTCGTAGCA----
CTATTTTGTAGGGATGAGTAGTTGTGCTCGGTAATGCACAATG-
AAATAGGTGGTAGTGTCCATCTAAGGCTAAATATGACATAGAGACCGATAGTGGATAA-----
GTAAAGTGATCGAAAATGGAATAGA
>DBP2
AGC-TATATGTGGAGCGGTGAAAGGCTCAGTAACGGGCGATTTATTTAATC-TCCTGGGA-------
CGGACAACATCGG-GAAACTGATGGGAAAACGTCTAAGTTGCAG------T-
TTTGTTATTGCGACGTAAACG-----------
GTAATCGTGCAGGAGAGTAAGATGCCATCCTATCAGTTAGTAAGTAGGGTAAGGGCCTACTTAGACGAAGACG
GGTACGGGGAATGAGGGTTTGATTCCG--
GAGAGGGAGCCTGAGAGATGGCTACCAGGACCAAGGTCAGCAGCAGGCGCGAAAATTATCGAAGCCCGCC-
TAGGGGCGATAGTGAGGAGACGTGTAT---TCGAAGTACGTGTAAAGAACGTACTAATAACT---
GGAGGTCAAGTCTGGTGCCAGCATCCGCGGTAATTCCAGCTCCAGGGGTGTCTATGATGATTGCTGCGATTAA
AAAGTCCGTAGTCAAGCTGACTG-ACTGACCTGCAATGTGAT-TG------
ATTAAAGAACGAGCAGGGTTAGGAAAGCAGAGAATTAGGAGCGACCGAGGGCTAGAGTATTGAATGGCGAGAG
GTGAAATTTGATGACCCAT-
TCAGGAGTGACAAAGGCGAAGGCACTAGTCAAGGGCGAATCCGATGATCAAGGACGTAGGCTAGAGTTTCGAA
AACGATT--AGAGACCGGAGTAGTTCTAGCAGTAAACTATGCCGACGCCGTGATATT--
GTTTTTTGTGGCGGTATTGCGGAAGAGAAATC--AAGT-
AAGGCTTTGGGGAGAGTACGCGCGCAAGCGATAAATTTAAAGGAAATTGACGGAGGAACACCACAAGGAGTGG
AGTGTGCGGCTTAATTTGACTCAACGCGGGACAGCTTACCAGGCCCGATAATCGAGCGAGCGTAGTACGCGAT
AGATTAAAGAGTGGTGCATGGCTGCTATCGACAGTTGGGGTGACC-TTAGGGTTAATTCCG-
GCAAGTAGTGAGACCCCTGCAGATAG---------TGGACAGGTATTTTT-CAAAATACAGGAAGG-
AAGGGACAAGAGCA-GGTCAGTGATGCCCTTAGATGGCCTGGGCT----GCACGCGCACTACAGTGG-
TCATTATAAGTAGAAG-------TT-AGA-TATAAAGA-----------TGATCGAGAGGGA-
CTGAGCTTTG-TAAGAGGCTCACGAACGAGGAATT-
GCTAGTAATCGTAGGCTCATTAAGATACGATGAATATGTCCCTGTACCTT---
GTACACACCGCCCGTCGTTATCGAAGATGGAATTGTGTGC-GAACGAGC--
AACAAGCGAGTGAGCGCATAGTTCTAGATGTGATAAAAG-
TCGTAACAAGGCAACTGTAGGAGAACCTGTAGTTGGATCATACAGATTTATTTC----TAAGTAGTTTT---
TTATAATTT-----CCTTA--------CGCAAGGG---ATCGTTTGGTTCTA--
TGTACGATGAAGGTCGAAGCAGTATTCGATAATGTAAGATGGTTGTATAAATTGTCTTATGTTACTGAATAGA
GTA-GTGATGCTCGAATACTCCTTTGAATTAAGCATATGAGTAAAGGAAGGAGAAGAAACTAAC-
AAGGATTCTCTTATTAGTGGCGAATGAACAGAGAATAGCCC-TAAGTGTAATCAAATTCATTGAAT-------
-TTGAGATGTCAGGTATC-ATGTAGT-----
GTGAATATACTGGAAAGTATTGCCAGAGAGAGTTATAGCCTCGTAGCA----

CTATTTTGTAGGGATGAGTAGTTGTGCTCGGTAATGCACAATG-AAATAGGTGGTAGTGTCCATCTAA-
GCTAAATATGACATAGAGACCGATAGTGGATAA-----GTAAAGTGATCGAAAATGGAATAGA
>ZWPr1
AGC-TATATGTGGAGCGGTGAAAGGCTCAGTAACGGGCGATTTATTTAATC-TCCTGGGA-------
CGGACAACATCGG-GAAACTGATGGGAAAACGTCTAAGTTGCAG------T-
TTTGTTATTGTGACGTAAACG-----------
GTAATCGTGCAGGAGAGTAAGATGCCATCCTATCAGTTAGTAAGTAGGGTAAGGGCCTACTTAGACGAAGACG
GGTACGGGGAATGAGGGTTTGATTCCG--
GAGAGGGAGCCTGAGAGATGGCTACCAGGACCAAGGTCAGCAGCAGGCGCGAAAATTATCGAAGCCCGCC-
TAGGGGCGATAGTGAGGAGACGTGTAT---TCGAAGTACGTGTAAAGAACGTACTAATAACT---
GGAGGTCAAGTCTGGTGCCAGCATCCGCGGTAATTCCAGCTCCAGGGGTGTCTATGATGATTGCTGCGATTAA
AAAGTCCGTAGTCAAGCTGACTG-ACTGACCTGCAATGTGAT-TG------
ATTAAAGAACGAGCAGGGTTAGGAAAGCAGAGAATTAGGAGCGACCGAGGGCTAGAGTATTGAATGGCGAGAG
GTGAAATTTGATGACCCAT-
TCAGGAGTGACAAAGGCGAAGGCACTAGTCAAGGGCGAATCCGATGATCAAGGACGTAGGCTAGAGTTTCGAA
AACGATT--AGAGACCGGAGTAGTTCTAGCAGTAAACTATGCCGACGCCGTGATATT--
GTTTTTTGTGGCGGTATTGCGGAAGAGAAATC--AAGT-
AAGGCTTTGGGGAGAGTACGCGCGCAAGCGATAAATTTAAAGGAAATTGACGGAGGAACACCACAAGGAGTGG
AGTGTGCGGCTTAATTTGACTCAACGCGGGACAGCTTACCAGGCCCGATAATCGAGCGAGCGTAGTACGCGAT
AGATTAAAGAGTGGTGCATGGCTGCTATCGACAGTTGGGGTGACC-TTAGGGTTAATTCCG-
GCAAGTAGTGAGACCCCTGCAGATAG---------TGGACAGGTATTTTT-CAAAATACAGGAAGG-
AAGGGACAAGAGCA-GGTCAGTGATGCCCTTAGATGGCCTGGGCT----GCACGCGCACTACAGTGG-
TCATTATAAGTAGAAG-------TT-AGA-TATAAAGA-----------TGATCGAGAGGGA-
CTGAGCTTTG-TAAGAGGCTCACGAACGAGGAATT-
GCTAGTAATCGTAGGCTCATTAAGATACGATGAATATGTCCCTGTACCTT---
GTACACACCGCCCGTCGTTATCGAAGATGGAATTGTGTGC-GAACGAGC--
AACAAGCGAGTGAGCGCATAGTTCTAGATGTGATAAAAG-
TCGTAACAAGGCAACTGTAGGAGAACCTGTAGTTGGATCATACAGATTTATTTT----TAAGTAGTTTT---
TTATAATTT-----CCTTA--------CGCAAGGG---ATCGTTTGGTTCTA--
TGTACGATGAAGGTCGAAGCAGTATTCGATAATGTAAGATGGTTGTATAAATTGTCTTATGTTACTGAATAGA
GTA-GTGATGCTCGAATACTCCTTTGAATTAAGCATATGAGTAAAGGAAGGAGAAGAAACTAAC-
AAGGATTCTCTTATTAGTGGCGAATGAACAGAGAATAGCCC-TAAGTGTAATCAAATTCATTGAAT-------
-TTGAGATGTCAGGTATC-ATGTAGT-----
GTGAATATACTGGAAAGTATTGCCAGAGAGAGTTATAGCCTCGTAGCA----
CTATTTTGTAGGGATGAGTAGTTGTGCTCGGTAATGCACAATG-
AAATAGGTGGTAGTGTCCATCTAAGGCTAAATATGACATAGAGACCGATAGTGGATAA-----
GTAAAGTGATCGAAAATGGAATAGA
>ZWH16
AGC-TATATGTGGAGCGGTGAAAGGCTCAGTAACGGGCGATTTATTTAATC-TCCTGGGA-------
CGGACAACATCGG-GAAACTGATGGGAAAACGTCTAAGTTGCAG------T-
TTTGTTATTGCGACGTAAACG-----------
GTAATCGTGCAGGAGAGTAAGATGCCATCCTATCAGTTAGTAAGTAGGGTAAGGGCCTACTTAGACGAAGACG
GGTACGGGGAATGAGGGTTTGATTCCG--
GAGAGGGAGCCTGAGAGATGGCTACCAGGACCAAGGTCAGCAGCAGGCGCGAAAATTATCGAAGCCCGCC-
TAGGGGCGATAGTGAGGAGACGTGTAT---TCGAAGTACGTGTAAAGAACGTACTAATAACT---
GGAGGTCAAGTCTGGTGCCAGCATCCGCGGTAATTCCAGCTCCAGGGGTGTCTATGATGATTGCTGCGATTAA
AAAGTCCGTAGTCAAGCTGACTG-ACTGACCTGCAATGTGAT-TG------
ATTAAAGAACGAGCAGGGTTAGGAAAGCAGAGAATTAGGAGCGACCGAGGGCTAGAGTATTGAATGGCGAGAG
GTGAAATTTGATGACCCAT-
TCAGGAGTGACAAAGGCGAAGGCACTAGTCAAGGGCGAATCCGATGATCAAGGACGTAGGCTAGAGTTTCGAA
AACGATT--AGAGACCGGAGTAGTTCTAGCAGTAAACTATGCCGACGCCGTGATATT--
GTTTTTTGTGGCGGTATTGCGGAAGAGAAATC--AAGT-
AAGGCTTTGGGGAGAGTACGCGCGCAAGCGATAAATTTAAAGGAAATTGACGGAGGAACACCACAAGGAGTGG
AGTGTGCGGCTTAATTTGACTCAACGCGGGACAGCTTACCAGGCCCGATAATCGAGCGAGCGTAGTACGCGAT
AGATTAAAGAGTGGTGCATGGCTGCTATCGACAGTTGGGGTGACC-TTAGGGTTAATTCCG-
GCAAGTAGTGAGACCCCTGCAGATAG---------TGGACAGGTATTTTT-CAAAATACAGGAAGG-
AAGGGACAAGAGCA-GGTCAGTGATGCCCTTAGATGGCCTGGGCT----GCACGCGCACTACAGTGG-
TCATTATAAGTAGAAG-------TT-AGA-TATAAAGA-----------TGATCGAGAGGGA-

CTGAGCTTTG-TAAGAGGCTCACGAACGAGGAATT-
GCTAGTAATCGTAGGCTCATTAAGATACGATGAATATGTCCCTGTACCTT---
GTACACACCGCCCGTCGTTATCGAAGATGGAATTGTGTGC-GAACGAGC--
AACAAGCGAGTGAGCGCATAGTTCTAGATGTGATAAAAG-
TCGTAACAAGGCAACTGTAGGAGAACCTGTAGTTGGATCATACAGATTTATTTT----TAAGTAGTTTT---
TTATAATTT-----CCTTA--------CGCAAGGG---ATCGTTTGGTTCTA--
TGTACGATGAAGGTCGGAGCAGTATTCGATAATGTAAGATGGTTGTATAAATTGTCTTATGTTACTGAATAGA
GTA-GTGATGCTCGAATACTCCTTTGAATTAAGCATATGAGTAAAGGAAGGAGAAGAAACTAAC-
AAGGATTCTCTTATTAGTGGCGAATGAACAGAGAATAGCCC-TAAGTGTAATCAAATTCATTGAAT-------
-TTGAGATGTCAGGTATC-ATGTAGT-----
GTGAATATACTGGAAAGTATTGCCAGAGAGAGTTATAGCCTCGTAGCA----
CTATTTTGTAGGGATGAGTAGTTGTGCTCGGTAATGCACAATG-
AAATAGGTGGTAGTGTCCATCTAAGGCTAAATATGACATAGAGACCGATAGTGGATAA-----
GTAAAGTGATCGAAAATGGAATAGA
>MB6
AGC-TATATGTGGAGCGGTGAAAGGCTCAGTAACGGGCGATTTATTTAATC-TCCTGGGG-------
CGGACAACATCGG-GAAACTGATGGGAAAACGTCTAAGTTGCATTAA---T----
TTTAGTGAGACGTAAACG-----------
ATTATCGTGCAGGAGAGTAAGATGCCATCCTATCAGTTAGTAAGTAGGGTAAGGGCCTACTTAGACGAAGACG
GGTACGGGGAATGAGGGTTTGATTCCG--
GAGAGGGAGCCTGAGAGATGGCTACCAGGACCAAGGTCAGCAGCAGGCGCGAAAATTATCGAAGCCCGCC-
TAGGGGCGATAGTGAGGAGACGTGTAT--AACGAAGTACGTGTAAAGAACGTACTAATAACT---
GGAGGTCAAGTCTGGTGCCAGCATCCGCGGTAATTCCAGCTCCAGGGGTGTCTATGATGATTGCTGCGATTAA
AAAGTCCGTAGTCAAGCTGCCTG-ACTGACCTGCAATGTGAT-TG------
ATTAAGGAACGAGCAGGGTTAGGAAAGCAGAGAATTAGGAGCGACCGAGGGCTAGAGTATTGAATGGCGAGAG
GTGAAATTTGATGACCCAT-
TCAGGAGTGACAAAGGCGAAGGCACTAGTCAAGGGCGAATCCGATGATCAAGGACGTAGGCTAGAGTTTTGAA
AACGATT--AGAGACCGGAGTAGTTCTAGCAGTAAACTATGCCGACGCCGTGGTATG--
GTTTTTTGTGGCTGTATTGCGGAAGAGAAATC--AAGT-
AAGGCTTTGGGGAGAGTACGCGCGCAAGCGATAAATTTAAAGGAAATTGACGGAGGAACACCACAAGGAGTGG
AGTGTGCGGCTTAATTTGACTCAACGCGGGACAGCTTACCAGGCCCGATAATCGAGCGAGCGTTGTACGCGAT
AGATTAAAGAGTGGTGCATGGCTGCTATCGACAGTTGGGGTGACC-TTAGGGTTAATTCCG-
GCAAGTAGTGAGACCCCTGCAGATAG---------TGGACAGGTATTTTT--AAAATACAGGAAGG-
AAGGGACAAGAGCA-GGTCAGTGATGCCCTTAGATGGCCTGGGCT----GCACGCGCACTACAGTGG-
TCATTATAAGTAGAAG-------TT-AGA-AGTAAAGA-----------TGATCGAGAGGGA-
CTGAGCTTTG-TAAGAGGCTCACGAACGAGGAATT-
GCTAGTAATCGTAGGCTCATTAAGATACGATGAATATGTCCCTGTACCTT---
GTACACACCGCCCGTCGTTATCGAAGATGGAATTGTGTGC-GAACGAGC--
AACAAGCGAGTGAGCGCATAGTTCTAGATGTGATAAAAG-
TCGTAACAAGGCAACTGTAGGAGAACCTGCAGTTGGATCATACAGATTTATTT-----TAGGTAGTTTA---
TAAAAAATT-----CCTTA--------CGCAAGGG---ATCGTTTGGCTCTA--
TGTGCGATGAAGGTCGGAGCAGTATTCGATAATATAAGATGATTGTATAAATTGTCTTATGTTACTGAATAGA
GTA-GTGATGCTCGATTACTCCTTTGAATTAAGCATATGAGTAAAGGAAGGAAAAGAAACCAAC-
GAGGATTCTCTTATTAGTGGCGAATGAACAGAGAACAGCCC-TAAGTGTAATCAAATTCGTTGAAT-------
-TTGAGATGTCAGGTATC-ATGTGGT-----
GTGAATGTACTGGAAAGTACTGCCGAAGAGAGTTATAGCCTCGTAGCA----
CCATTTTTTAGGTATGAGTAGTTGTGCTCGGTAATGCACAATG-
AAATAGGTGGTAGTGTCCATCTAAGGCTAAATATGACATAGAGACCGATAGTAAATAA-----
GTAAAGTGATCGAAAATGGAATAGA
>MB5
AGC-TATATGTGGAGCGGTGAAAGGCTCAGTAACGGGCGATTTATTTAATC-TCCTGGGG-------
CGGACAACATCGG-GAAACTGATGGGAAAACGTCTAAGTTGCATTAA---T----
TTTAGTGTGACGTAAACG-----------
ATTATCGTGCAGGAGAGTAAGATGCCATCCTATCAGTTAGTAAGTAGGGTAAGGGCCTACTTAGACGAAGACG
GGTACGGGGAATGAGGGTTTGATTCCG--
GAGAGGGAGCCTGAGAGATGGCTACCAGGACCAAGGTCAGCAGCAGGCGCGAAAATTATCGAAGCCCGCC-
TAGGGGCGATAGTGAGGAGACGTGTAT--AACGAAGTACGTGTAAAGAACGTACTAATAACT---
GGAGGTCAAGTCTGGTGCCAGCATCCGCGGTAATTCCAGCTCCAGGGGTGTCTATGATGATTGCTGCGATTAA

AAAGTCCGTAGTCAAGCTGCCTG-ACTGACCTGCAATGTGAT-TG------
ATTAAGGAACGAGCAGGGTTAGGAAAGCAGAGAATTAGGAGCGACCGAGGGCTAGAGTATTGAATGGCGAGAG
GTGAAATTTGATGACCCAT-
TCAGGAGTGACAAAGGCGAAGGCACTAGTCAAGGGCGAATCCGATGATCAAGGACGTAGGCTAGAGTTTCGAA
AACGATT--AGAGACCGGAGTAGTTCTAGCAGTAAAATATGCCGACGCCGTGGTATG--
GTTTTTTGTGGCTGTATTGCGGAAGAGAAATC--AAGT-
AAGGCTTTGGGGAGAGTACGCGCGCAAGCGATAAATTTAAAGGAAATTGACGGAGGAACACCACAAGGAGTGG
AGTGTGCGGCTTAATTTGACTCAACGCGGGACAGCTTACCAGGCCCGATAATCGAGCGAGCGTTGTACGCGAT
AGATTAAAGAGTGGTGCATGGCTGCTATCGACAGTTGGGGTGACC-TTAGGGTTAATTCCG-
GCAAGTAGTGAGACCCCTGCAGATAG---------TGGACAGGTATTTTT--AAAATACAGGAAGG-
AAGGGACAAGAGCA-GGTCAGTGATGCCCTTAGATGGCCTGGGCT----GCACGCGCACTACAGTGG-
TCATTATAAGTAGAAG-------TT-AGA-AGTAAAGA-----------TGATCGAGAGGGA-
CTGAGCTTTG-TAAGAGGCTCACGAACGAGGAATT-
GCTAGTAATCGTAGGCTCATTAAGATACGATGAATATGTCCCTGTACCTT---
GTACACACCGCCCGTCGTTATCGAAGATGGAATTGTGTGC-GAACGAGC--
AACAAGCGAGTGAGCGCATAGTTCTAGATGTGATAAAAG-
TCGTAACAAGGCAACTGTAGGAGAACCTGCAGTTGGATCATACAGATTTATTT-----TAGGTAGTTTA---
TAAAAAATT-----CCTTA--------CGCAAGGG---ATCGTTTGGCTCTA--
TGTGCGATGAAGGTCGGAGCAGTATTCGATAATATAAGATGATTGTATAAATTGTCTTATGTTACTGAATAGA
GTA-GTGATGCTCGATTACTCCTTTGAATTAAGCATATGAGTAAAGGAAGGAAAAGAAACCAAC-
GAGGATTCTCTTATTAGTGGCGAATGAACAGAGAACAGCCC-TAAGTGTAATCAAATTCGTTGAAT-------
-TTGAGATGTCAGGTATC-ATGTGGT-----
GTGAATGTACTGGAAAGTACTGCCGAAGAGAGTTATAGCCTCGTAGCA----
CCATTTTTTAGGTATGAGTAGTTGTGCTCGGTAATGCACAATG-
AAATAGGTGGTAGTGTCCATCTAAGGCTAAATATGACATAGAGACCGATAGTAAATAA-----
GTAAAGTGATCGAAAATGGAATAGA
>Gvar_M48
AGC-TATATGTGGAGCGGTGAAAGGCTCAGTAACGGGCGATTTATTTAATC-TCCTGGGA-------
CGGACAACATCGG-GAAACTGATGGGAAAACGTCTAAGTTGCATTAA---T----
GTTAGTGTGACGTAAACG------------
ATATCGTGCAGGAGAGTAAGATGCCATCCTATCAGTTAGTAAGTAGGGTAAGGGCCTACTTAGACGAAGACGG
GTACGGGGAATGAGGGTTTGATTCCG--
GAGAGGGAGCCTGAGAGATGGCTACCAGGACCAAGGTCAGCAGCAGGCGCGAAAATTATCGAAGCCCGCC-
TAGGGGCGATAGTGAGGAGACGTGTAT--AACGAAGTACGTGTAAAGAACGTACTAATAACT---
GGAGGTCAAGTCTGGTGCCAGCATCCGCGGTAATTCCAGCTCCAGGGGTGTCTATGATGATTGCTGCGATTAA
AAAGTCCGTAGTCAAGCTGACTG-ACTGACCTGCAATGTGAT-TG------
ATTAAGGAACGAGCAGGGTTAGGAAAGCAGAGAATTAGGAGCGACCGAGGGCTAGAGTATTGAATGGCGAGAG
GTGAAATTTGATGACCCAT-
TCAGGAGTGACAAAGGCGAAGGCACTAGTCAAGGGCGAATCCGATGATCAAGGACGTAGGCTAGAGTTTCGAA
AACGATT--AGAGACCGGAGTAGTTCTAGCAGTAAACTATGCCGACGCCGTGATATA--
GTTTTTTGTGGCTGTATTGCGGAAGAGAAATC--AAGT-
AAGGCTTTGGGGAGAGTACGCGCGCAAGCGATAAATTTAAAGGAAATTGACGGAGGAACACCACAAGGAGTGG
AGTGTGCGGCTTAATTTGACTCAACGCGGGACAGCTTACCAGGCCCGATAATCGAGCGAGCGTTGTACGCGAT
AGATTAAAGAGTGGTGCATGGCTGCTATCGACAGTTGGGGTGACCTTTAGGGTTAATTCCG-
GCAAGTAGTGAGACCCCTGCAGATAG---------TGGACAGGTATTTTT--AAAATACAGGAAGG-
AAGGGACAAGAGCA-GGTCAGTGATGCCCTTAGATGGCCTGGGCT----GCACGCGCACTACAGTGG-
TCATTATAAGTAGAAG-------TT-AGA-AGTAAAGA-----------TGATCGAGAGGGA-
CTGAGCTTTG-TAAGAGGCTCACGAACGAGGAATT-
GCTAGTAATCGTAGGCTCATTAAGATACGATGAATATGTCCCTGTACCTT---
GTACACACCGCCCGTCGTTATCGAAGATGGAATTGTGTGC-GAACGAGC--
AACAAGCGAGTGAGCGCATAGTTCTAGATGTGATAAAAG-
TCGTAACAAGGCAACTGTAGGAGAACCTGTAGTTGGATCATACAGATTTAATT-----TAAGTAGTTTT---
TAATAAATT-----CCTTA--------CGCAAGGG---ATCGTTTGGCTCTA--
TGTGCGATGAAGGTCGGAGCAGTATTCGATAATATAAGATGATTGTATAAATTGTCTTATGTTACTGAATAGA
GTA-GTGATGCTCGATTACTCCTTTGAATTAAGCATATGAGTAAAGGA-GGAGAAGAAACTAAC-
AAGGATTCTCTTATTAGTGGCGAATGAACAGAGAACAGCCC-TAAGTGTAATCAAATTCTATGAAT-------
-TTGAGATGTCAGGTATC-ATGTGGT-----
GTGAATGTACTGGAAAGTACTGCCGAAGAGAGTTATAGCCTCGTAACA----

CCATTTTGTAGGTATGAGTAGTTGTGCTCGGTAATGCACAATG-
AAATAGGTGGTAGTGTCCATCTAAGGCTAAATATGACATAGAGACCGATAGTGAATAA-----
GTAAAGTGATCGAAAATGGAATAGA
>M33_Pr
AGC-TATATGTGGAGCGGTGAAAGGCTCAGTAACGGGCGATTTATTTAATC-TCCTGGGA-------
CGGACAACATCGG-GAAACTGATGGGAAAACGTCTAAGTTGCATTAA---
TATTAGTTAGTGTGACGTAAACG-----------
GTAATCGTGCAGGAGAGTAAGATGCCATCCTATCAGTTAGTAAGTAGGGTAAGGGCCTACTTAGACGAAGACG
GGTACGGGGAATGAGGGTTTGATTCCG--
GAGAGGGAGCCTGAGAGATGGCTACCAGGACCAAGGTCAGCAGCAGGCGCGAAAATTATCGAAGCCCGCC-
TAGGGGCGATAGTGAGGAGACGTGTAT--AACGAAGTACGTGTAAAGAACGTACTAATAACT---
GGAGGTCAAGTCTGGTGCCAGCATCCGCGGTAATTCCAGCTCCAGGGGTGTCTATGATGATTGCTGCGATTAA
AAAGTCCGTAGTCAAACTGACTG-ACTGACCTGCAATGTGAT-TG------
ATTAAAGAACGAGCAGGGTTAGGAAAGCAGAGAATTAGGAGCGACCGAGGGCTAGAGTATTGAATGGCGAGAG
GTGAAATTTGATGACCCAT-
TCAGGAGTGACAAAGGCGAAGGCACTAGTCAAGGGCGAATCCGATGATCAAGGACGTAGGCTAGAGTTTCGAA
AACGATT--AGAGACCGGAGTAGTTCTAGCAGTAAACTATGCCGACGCCGTGATATT--
GTTTTTTGTGGCAGTATTGCGGAAGAGAAATC--AAGT-
AAAGCTTTGGGGAGAGTACGCGCGCAAGCGATAAATTTAAAGGAAATTGACGGAGGAACACCACAAGGAGTGG
AGTGTGCGGCTTAATTTGACTCAACGCGGGACAGCTTACCAGGCCCGATAATCGAGCGAGCGTAGTACGCGAT
AGATTAAAGAGTGGTGCATGGCTGCTATCGACAGTTGGGGTGACC-TTAGGGTTAATTCCG-
GCAAGTAGTGAGACCCCTGCAGATAG---------TGGACAGGTATTTTT--AAAATACAGGAAGG-
AAGGGACAAGAGCA-GGTCAGTGATGCCCTTAGATGGCCTGGGCT----GCACGCGCACTACAGTGG-
TCATTATAAGTAGAAA-------TT-AGA-AATAAAAA-----------TGATCGAGAGGGA-
CTGAGCTTTG-TAAGAGGCTCACGAACGAGGAATT-
GCTAGTAATCGTAGGCTCATTAAGATACGATGAATATGTCCCTGTACCTT---
GTACACACCGCCCGTCGTTATCGAAGATGGAATTGTGTGC-GAACGAGC--
AACAAGCGAGTGAGCGCATAGTTCTAGATGTGATAAAAG-
TCGTAACAAGGCAACTGTAGGAGAACCTGTAGTTGGATCATACAGATTTATAT-----
TAAGTAGTTTTTTATATTAAATT-----CCTTA--------CGCAAGGG---ATCGTTTGGTTCTA--
TGTACGATGAAGGTCGGAGCAGTATTCGATAATATAAAATGATTGTTTAAATTGTTTTATGTTACTGAATAGA
GTA-GTGATGCTCGATTACTCCTTTGAATTAAGCATATGAGTAAAGGAAGGAAAAGAAACTAAC-
AAGGATTCTCTTATTAGTGGCGAATGAACAGAGAAGAGCCC-TAAGTGTAATCAAATTCTATGAAT-------
-TTGAGATGTCAGGTATA-AAGTGGT-----
GTGAATGTATTGGAAAATACAGCCTAAGAGAGTTATAGCCTCGTAACA----
CCATTTTGTAGGAATGAGTAGTTGTGCTCGGTAATGCACAATG-
AAATAGGTGGTAGTGTCCATCTAAGGCTAAATATGACATAGAGACCGATAGTGAATAA-----
GTAAAGTGATCGAAAATGGAATAGA
>M22_Dh_PrOv2
AGC-TATATGTGGAGCGGTGAAAGGCTCAGTAACGGGCGATTTATTTAATC-TCCTGGGA-------
CGGACAACATCGG-GAAACTGATGGGAAAACGTCTAAGTTGCATTAA---
TATTAGTTAGTGTGACGTAAACG-----------
GTAATCGTGCAGGAGAGTAAGATGCCATCCTATCAGTTAGTAAGTAGGGTAAGGGCCTACTTAGACGAAGACG
GGTACGGGGAATGAGGGTTTGATTCCG--
GAGAGGGAGCCTGAGAGATGGCTACCAGGACCAAGGTCAGCAGCAGGCGCGAAAATTATCGAAGCCCGCC-
TAGGGGCGATAGTGAGGAGACGTGTAT--AACGAAGTACGTGTAAAGAACGTACTAATAACT---
GGAGGTCAAGTCTGGTGCCAGCATCCGCGGTAATTCCAGCTCCAGGGGTGTCTATGATGATTGCTGCGATTAA
AAAGTCCGTAGTCAAACTGACTG-ACTGACCTGCAATGTGAT-TG------
ATTAAAGAACGAGCAGGGTTAGGAAAGCAGAGAATTAGGAGCGACCGAGGGCTAGAGTATTGAATGGCGAGAG
GTGAAATTTGATGACCCAT-
TCAGGAGTGACAAAGGCGAAGGCACTAGTCAAGGGCGAATCCGATGATCAAGGACGTAGGCTAGAGTTTCGAA
AACGATT--AGAGACCGGAGTAGTTCTAGCAGTAAACTATGCCGACGCCGTGATATT--
GTTTTTTGTGGCAGTATTGCGGAAGAGAAATC--AAGT-
AAGGCTTTGGGGAGAGTACGCGCGCAAGCGATAAATTTAAAGGAAATTGACGGAGGAACACCACAAGGAGTGG
AGTGTGCGGCTTAATTTGACTCAACGCGGGACAGCTTACCAGGCCCGATAATCGAGCGAGCGTAGTACGCGAT
AGATTAAAGAGTGGTGCATGGCTGCTATCGACAGTTGGGGTGACC-TTAGGGTTAATTCCG-
GCAAGTAGTGAGACCCCTGCAGATAG---------TGGACAGGTATTTTT--AAAATACAGGAAGG-
AAGGGACAAGAGCA-GGTCAGTGATGCCCTTAGATGGCCTGGGCT----GCACGCGCACTACAGTGG-

TCATTATAAGTAGAAA-------TT-AGA-AATAAAAA-----------TGATCGAGAGGGA-
CTGAGCTTTG-TAAGAGGCTCACGAACGAGGAATT-
GCTAGTAATCGTAGGCTCATTAAGATACGATGAATATGTCCCTGTACCTT---
GTACACACCGCCCGTCGTTATCGAAGATGGAATTGTGTGC-GAACGAGC--
AACAAGCGAGTGAGCGCATAGTTCTAGATGTGATAAAAG-
TCGTAACAAGGCAACTGTAGGAGAACCTGTAGTTGGATCATACAGATTTATAT-----
TAAGTAGTTTTTTATAATAAATT-----CCTTA--------CGCAAGGG---ATCGTTTGGTTCTA--
TGTACGATGAAGGTCGGAGCAGTATTCGATAATATAAAATGATTGTTTAAATTGTTTTATGTTACTGAATAGA
GTA-GTGATGCTCGATTACTCCTTTGAATTAAGCATATGAGTAAAGGAAGGAAAAGAAACTAAC-
AAGGATTCTCTTATTAGTGGCGAATGAACAGAGAAGAGCCC-TAAGTGTAATCAAATTCTATGAAT-------
-TTGAGATGTCAGGTATA-AATTGGT-----
GTGAATGTATTGGAAAATACAGCCTAAGAGAGTTATAGCCTCGTAACA----
CCATTTTGTAGGAATGAGTAGTTGTGCTCGGTAATGCACAATG-
AAATAGGTGGTAGTGTCCATCTAAGGCTAAATATGACATAGAGACCGATAGTGAATAA-----
GTAAAGTGATCGAAAATGGAATAGA
>M34_Gaequi
AGC-TATATGTGGAGCGGTGAAAGGCTCAGTAACGGGCGATTTATTTAATC-TCCTGGGA-------
CGGACAACATCGG-GAAACTGATGGGAAAACGTCTAAGTTGCATTAA---
TATTAGTTAGTGTGACGTAAACG-----------
GTAATCGTGCAGGAGAGTAAGATGCCATCCTATCAGTTAGTAAGTAGGGTAAGGGCCTACTTAGACGAAGACG
GGTACGGGGAATGAGGGTTTGATTCCG--
GAGAGGGAGCCTGAGAGATGGCTACCAGGACCAAGGTCAGCAGCAGGCGCGAAAATTATCGAAGCCCGCC-
TAGGGGCGATAGTGAGGAGACGTGTAT--AACGAAGTACGTGTAAAGAACGTACTAATAACT---
GGAGGTCAAGTCTGGTGCCAGCATCCGCGGTAATTCCAGCTCCAGGGGTGTCTATGATGATTGCTGCGATTAA
AAAGTCCGTAGTCAAACTGACTG-ACTGACCTGCAATGTGAT-TG------
ATTAAAGAACGAGCAGGGTTAGGAAAGCAGAGAATTAGGAGCGACCGAGGGCTAGAGTATTGAATGGCGAGAG
GTGAAATTTGATGACCCAT-
TCAGGAGTGACAAAGGCGAAGGCACTAGTCAAGGGCGAATCCGATGATCAAGGACGTAGGCTAGAGTTTCGAA
AACGATT--AGAGACCGGAGTAGTTCTAGCAGTAAACTATGCCGACGCCGTGATATT--
GTTTTTTGTGGCGGTATTGCGGAAGAGAAATC--AAGT-
AAGGCTTTGGGGAGAGTACGCGCGCAAGCGATAAATTTAAAGGAAATTGACGGAGGAACACCACAAGGAGTGG
AGTGTGCGGCTTAATTTGACTCAACGCGGGACAGCTTACCAGGCCCGATAATCGAGCGAGCGTAGTACGCGAT
AGATTAAAGAGTGGTGCATGGCTGCTATCGACAGTTGGGGTGACC-TTAGGGTTAATTCCG-
GCAAGTAGTGAGACCCCTGCAGATAG---------TGGACAGGTATTTTT--AAAATACAGGAAGG-
AAGGGACAAGAGCA-GGTCAGTGATGCCCTTAGATGGCCTGGGCT----GCACGCGCACTACAGTGG-
TCATTATAAGTAGAAA-------TT-AGA-AGTAAAAA-----------TGATCGAGAGGGA-
CTGAGCTTTG-TAAGAGGCTCACGAACGAGGAATT-
GCTAGTAATCGTAGGCTCATTAAGATACGATGAATATGTCCCTGTACCTT---
GTACACACCGCCCGTCGTTATCGAAGATGGAATTGTGTGC-GAACGAGC--
AACAAGCGAGTGAGCGCATAGTTCTAGATGTGATAAAAG-
TCGTAACAAGGCAACTGTAGGAGAACCTGTAGTTGGATCATACAGATTTATAT-----
TAAGTAGTTTTTTATAATAAATT-----CCTTA--------CGCAAGGG---ATCGTTTGGTTCTA--
TGTACGATGAAGGTCGGAGCAGTATTCGATAATATAAAATGATTGTTTAAATTGTTTTATGTTACTGAATAGA
GTA-GTGATGCTCGATTACTCCTTTGAATTAAGCATATGAGTAAAGGAAGGAAAAGAAACTAAC-
AAGGATTCTCTTATTAGTGGCGAATGAACAGAGAAGAGCCC-TAAGTGTAATCAAATTCTATGAAT-------
-TTGAGATGTCAGGTATC-ATGTGGT-----
GTGAATGTATTGGAAAATACAGCCTAAGAGAGTTATAGCCTCGTAACA----
CCATTTTGTAGGAATGAGTAGTTGTGCTCGGTAATGCACAATG-
AAATAGGTGGTAGTGTCCATCTAAGGCTAAATATGACATAGAGACCGATAGTGAATAA-----
GTAAAGTGATCGAAAATGGAATAGA
>DVIL_ALP23-22
AGC-TATATGTGGAGCGGTGAAAGGCTCAGTAACGGGCGATTTATTTAATC-TCCTGGGA-------
CGGACAACATCGG-GAAACTGATGGGAAAACGTTTAGTTGCATTAA---
TATTAGTTAGTGTGACGTAAACG-----------
GTAATCGTGCAGGAGAGTAAGATGCCATCCTATCAGTTAGTAAGTAGGGTAAGGGCCTACTTAGACGAAGACG
GGTACGGGGAATGAGGGTTTGATTCCG--
GAGAGGGAGCCTGAGAGATGGCTACCAGGACCAAGGTCAGCAGCAGGCGCGAAAATTATCGAAGCCCGCC-
TAGGGGCGATAGTGAGGAGACGTGTAT--AACGAAGTACGTGTAAAGAACGTACTAATAACT---

GGAGGTCAAGTCTGGTGCCAGCATCCGCGGTAATTCCAGCTCCAGGGGTGTCTATGATGATTGCTGCGATTAA
AAAGTCCGTAGTCAAACTGACTG-ACTGACCTGCAATGTGAT-TG------
ATTAAAGAACGAGCAGGGTTAGGAAAGCAGAGAATTAGGAGCGACCGAGGGCTAGAGTATTGAATGGCGAGAG
GTGAAATTTGATGACCCAT-
TCAGGAGTGACAAAGGCGAAGGCACTAGTCAAGGGCGAATCCGATGATCAAGGACGTAGGCTAGAGTTTCGAA
AACGATT--AGAGACCGGAGTAGTTCTAGCAGTAAACTATGCCGACGCCGTGATATT--
GTTTTTTGTGGCAGTATTGCGGAAGAGAAATC--AAGT-
AAGGCTTTGGGGAGAGTACGCGCGCAAGCGATAAATTTAAAGGAAATTGACGGAGGAACACCACAAGGAGTGG
AGTGTGCGGCTTAATTTGACTCAACGCGGGACAGCTTACCAGGCCCGATAATCGAGCGAGCGTAGTACGCGAT
AGATTAAAGAGTGGTGCATGGCTGCTATCGACAGTTGGGGTGACC-TTAGGGTTAATTCCG-
GCAAGTAGTGAGACCCCTGCAGATAG---------TGGACAGGTATTTTT--AAAATACAGGAAGG-
AAGGGACAAGAGCA-GGTCAGTGATGCCCTTAGATGGCCTGGGCT----GCACGCGCACTACAGTGG-
TCATTATAAGTAGAAA-------TT-AGA-AGTAAAAA-----------TGATCGAGAGGGA-
CTGAGCTTTG-TAAGAGGCTCACGAACGAGGAATT-
GCTAGTAATCGTAGGCTCATTAAGATACGATGAATATGTCCCTGTACCTT---
GTACACACCGCCCGTCGTTATCGAAGATGGAATTGTGTGC-GAACGAGC--
AACAAGCGAGTGAGCGCATAGTTCTAGATGTGATAAAAG-
TCGTAACAAGGCAACTGTAGGAGAACCTGTAGTTGGATCATACAGATTTATAT-----
TAAGTAGTTTTTTATAATAAATT-----CCTTA--------CGCAAGGG---ATCGTTTGGTTCTA--
TGTACGATGAAGGTCGGAGCAGTATTCGATAATATAAAATGATTGTTTAAATTGTTTTATGTTACTGAATAGA
GTA-GTGATGCTCGATTACTCCTTTGAATTAAGCATATGAGTAAAGGAAGGAAAAGAAACTAAC-
AAGGATTCTCTTATTAGTGGCGAATGAACAGAGAAGAGCCC-TAAGTGTAATCAAATTCTATGAAT-------
-TTGAGATGTCAGGTATA-ATGTGGT-----
GTGAATGTATTGGAAAATACAGCCTAAGAGAGTTATAGCCTCGTAACA----
CCATTTTGTAGGAATGAGTAGTTGTGCTCGGTAATGCACAATG-
AAATAGGTGGTAGTGTCCATCTAAGGCTAAATATGACATAGAGACCGATAGTGAATAA-----
GTAAAGTGATCGAAAATGGAATAGA
>DVIL_ALP25-17
AGC-TATATGTGGAGCGGTGAAAGGCTCAGTAACGGGCGATTTATTTAATC-TCCTGGGA-------
CGGACAACATCGG-GAAACTGATGGGAAAACGTCTAAGTTGCATTAA---
TATTAGTTAGTGTGACGTAAACG-----------
GTAATCGTGCAGGAGAGTAAGATGCCATCCTATCAGTTAGTAAGTAGGGTAAGGGCCTACTTAGACGAAGACG
GGTACGGGGAATGAGGGTTTGATTCCG--
GAGAGGGAGCCTGAGAGATGGCTACCAGGACCAAGGTCAGCAGCAGGCGCGAAAATTATCGAAGCCCGCC-
TAGGGGCGATAGTGAGGAGACGTGTAT--AACGAAGTACGTGTAAAGAACGTACTAATAACT---
GGAGGTCAAGTCTGGTGCCAGCATCCGCGGTAATTCCAGCTCCAGGGGTGTCTATGATGATTGCTGCGATTAA
AAAGTCCGTAGTCAAACTGACTG-ACTGACCTGCAATGTGAT-TG------
ATTAAAGAACGAGCAGGGTTAGGAAAGCAGAGAATTAGGAGCGACCGAGGGCTAGAGTATTGAATGGCGAGAG
GTGAAATTTGATGACCCAT-
TCAGGAGTGACAAAGGCGAAGGCACTAGTCAAGGGCGAATCCGATGATCAAGGACGTAGGCTAGAGTTTCGAA
AACGATT--AGAGACCGGAGTAGTTCTAGCAGTAAACTATGCCGACGCCGTGATATT--
GTTTTTTGTGGCAGTATTGCGGAAGAGAAATC--AAGT-
AAGGCTTTGGGGAGAGTACGCGCGCAAGCGATAAATTTAAAGGAAATTGACGGAGGAACACCACAAGGAGTGG
AGTGTGCGGCTTAATTTGACTCAACGCGGGACAGCTTACCAGGCCCGATAATCGAGCGAGCGTAGTACGCGAT
AGATTAAAGAGTGGTGCATGGCTGCTATCGACAGTTGGGGTGACC-TTAGGGTTAATTCCG-
GCAAGTAGTGAGACCCCTGCAGATAG---------TGGACAGGTATTTTT--AAAATACAGGAAGG-
AAGGGACAAGAGCA-GGTCAGTGATGCCCTTAGATGGCCTGGGCT----GCACGCGCACTACAGTGG-
TCATTATAAGTAGAAA-------TT-AGA-AATAAAGA-----------TGATCGAGAGGGA-
CTGAGCTTTG-TAAGAGGCTCACGAACGAGGAATT-
GCTAGTAATCGTAGGCTCATTAAGATACGATGAATATGTCCCTGTACCTT---
GTACACACCGCCCGTCGTTATCGAAGATGGAATTGTGTGC-GAACGAGC--
AACAAGCGAGTGAGCGCATAGTTCTAGATGTGATAAAAG-
TCGTAACAAGGCAACTGTAGGAGAACCTGTAGTTGGATCATACAGATTTATAT-----
TAAGTAGTTTTTTATATTAAATT-----CCTTA--------CGCAAGGG---ATCGTTTGGTTCTA--
TGTACGATGAAGGTCGGAGCAGTATTCGATAATATAAAATGATTGTTTAAATTGTTTTATGTTACTGAATAGA
GTA-GTGATGCTCGATTACTCCTTTGAATTAAGCATATGAGTAAAGGAAGGAAAAGAAACTAAC-
AAGGATTCTCTTATTAGTGGCGAATGAACAGAGAAGAGCCC-TAAGTGTAATCAAATTCTATGAAT-------
-TTGAGATGTCAGGTATA-ATGTGGT-----

GTGAATGTATTGGAAAATACAGCCTAAGAGAGTTATAGCCTCGTAACA----
CCATTTTGTAGGAATGAGTAGTTGTGCTCGGTAATGCACAATG-
AAATAGGTGGTAGTGTCCATCTAAGGCTAAATATGACATAGAGACCGATAGTGAATAA-----
GTAAAGTGATCGAAAATGGAATAGA
>DVIL_ALP25-19
AGC-TATATGTGGAGCGGTGAAAGGCTCAGTAACGGGCGATTTATTTAATC-TCCTGGGA-------
CGGACAACATCGG-GAAACTGATGGGAAAACGTCTAAGTTGCATTAA---
TATTAGTTAGTGTGACGTAAACG-----------
GTAATCGTGCAGGAGAGTAAGATGCCATCCTATCAGTTAGTAAGTAGGGTAAGGGCCTACTTAGACGAAGACG
GGTACGGGGAATGAGGGTTTGATTCCG--
GAGAGGGAGCCTGAGAGATGGCTACCAGGACCAAGGTCAGCAGCAGGCGCGAAAATTATCGAAGCCCGCC-
TAGGGGCGATAGTGAGGAGACGTGTAT--AACGAAGTACGTGTAAAGAACGTACTAATAACT---
GGAGGTCAAGTCTGGTGCCAGCATCCGCGGTAATTCCAGCTCCAGGGGTGTCTATGATGATTGCTGCGATTAA
AAAGTCCGTAGTCAAACTGACTG-ACTGACCTGCAATGTGAT-TG------
ATTAAAGAACGAGCAGGGTTAGGAAAGCAGAGAATTAGGAGCGACCGAGGGCTAGAGTATTGAATGGCGAGAG
GTGAAATTTGATGACCCAT-
TCAGGAGTGACAAAGGCGAAGGCACTAGTCAAGGGCGAATCCGATGATCAAGGACGTAGGCTAGAGTTTCGAA
AACGATT--AGAGACCGGAGTAGTTCTAGCAGTAAACTATGCCGACGCCGTGATATT--
GTTTTTTGTGGCAGTATTGCGGAAGAGAAATC--AAGT-
AAGGCTTTGGGGAGAGTACGCGCGCAAGCGATAAATTTAAAGGAAATTGACGGAGGAACACCACAAGGAGTGG
AGTGTGCGGCTTAATTTGACTCAACGCGGGACAGCTTACCAGGCCCGATAATCGAGCGAGCGTAGTACGCGAT
AGATTAAAGAGTGGTGCATGGCTGCTATCGACAGTTGGGGTGACC-TTAGGGTTAATTCCG-
GCAAGTAGTGAGACCCCTGCAGATAG---------TGGACAGGTATTTTT--AAAATACAGGAAGG-
AAGGGACAAGAGCA-GGTCAGTGATGCCCTTAGATGGCCTGGGCT----GCACGCGCACTACAGTGG-
TCATTATAAGTAGAAA-------TT-AGA-AGTAAAAA-----------TGATCGAGAGGGA-
CTGAGCTTTG-TAAGAGGCTCACGAACGAGGAATT-
GCTAGTAATCGTAGGCTCATTAAGATACGATGAATATGTCCCTGTACCTT---
GTACACACCGCCCGTCGTTATCGAAGATGGAATTGTGTGC-GAACGAGC--
AACAAGCGAGTGAGCGCATAGTTCTAGATGTGATAAAAG-
TCGTAACAAGGCAACTGTAGGAGAACCTGTAGTTGGATCATACAGATTTATAT-----
TAAGTAGTTTTTTATATTAAATT-----CCTTA--------CGCAAGGG---ATCGTTTGGTTCTA--
TGTACGATGAAGGTCGGAGCAGTATTCGATAATATAAAATGATTGTTTAAATTGTTTTATGTTACTGAATAGA
GTA-GTGATGCTCGATTACTCCTTTGAATTAAGCATATGAGTAAAGGAAGGAGAAGAAACTAAC-
AAGGATTCTCTTATTAGTGGCGAATGAACAGAGAAGAGCCC-TAAGTGTAATCAAATTCTATGAAT-------
-TTGAGATGTCAGGTATA-AATTGGT-----
GTGAATGTATTGGAAAATACAGCCTAAGAGAGTTATAGCCTCGTAACA----
CCATTTTGTAGGAATGAGTAGTTGTGCTCGGTAATGCACAATG-
AAATAGGTGGTAGTGTCCATCTAAGGCTAAATATGACATAGAGACCGATAGTGAATAA-----
GTAAAGTGATCGAAAATGGAATAGA
>DVIL_ALP30-3
AGC-TATATGTGGAGCGGTGAAAGGCTCAGTAACGGGCGATTTATTTAATC-TCCTGGGA-------
CGGACAACATCGG-GAAACTGATGGGAAAACGTCTAAGTTGCATTAA---
TATTAGTTAGTGTGACGTAAACG-----------
GTAATCGTGCAGGAGAGTAAGATGCCATCCTATCAGTTAGTAAGTAGGGTAAGGGCCTACTTAGACGAAGACG
GGTACGGGGAATGAGGGTTTGATTCCG--
GAGAGGGAGCCTGAGAGATGGCTACCAGGACCAAGGTCAGCAGCAGGCGCGAAAATTATCGAAGCCCGCC-
TAGGGGCGATAGTGAGGAGACGTGTAT--AACGAAGTACGTGTAAAGAACGTACTAATAACT---
GGAGGTCAAGTCTGGTGCCAGCATCCGCGGTAATTCCAGCTCCAGGGGTGTCTATGATGATTGCTGCGATTAA
AAAGTCCGTAGTCAAACTGACTG-ACTGACCTGCAATGTGAT-TG------
ATTAAAGAACGAGCAGGGTTAGGAAAGCAGAGAATTAGGAGCGACCGAGGGCTAGAGTATTGAATGGCGAGAG
GTGAAATTTGATGACCCAT-
TCAGGAGTGACAAAGGCGAAGGCACTAGTCAAGGGCGAATCCGATGATCAAGGACGTAGGCTAGAGTTTCGAA
AACGATT--AGAGACCGGAGTAGTTCTAGCAGTAAACTATGCCGACGCCGTGATATT--
GTTTTTTGTGGCAGTATTGCGGAAGAGAAATC--AAGT-
AAGGCTTTGGGGAGAGTACGCGCGCAAGCGATAAATTTAAAGGAAATTGACGGAGGAACACCACAAGGAGTGG
AGTGTGCGGCTTAATTTGACTCAACGCGGGACAGCTTACCAGGCCCGATAATCGAGCGAGCGTAGTACGCGAT
AGATTAAAGAGTGGTGCATGGCTGCTATCGACAGTTGGGGTGACC-TTAGGGTTAATTCCG-
GCAAGTAGTGAGACCCCTGCAGATAG---------TGGACAGGTATTTTT--AAAATACAGGAAGG-

AAGGGACAAGAGCA-GGTCAGTGATGCCCTTAGATGGCCTGGGCT----GCACGCGCACTACAGTGG-
TCATTATAAGTAGAAA-------TT-AGA-AATAAAAA-----------TGATCGAGAGGGA-
CTGAGCTTTG-TAAGAGGCTCACGAACGAGGAATT-
GCTAGTAATCGTAGGCTCATTAAGATACGATGAATATGTCCCTGTACCTT---
GTACACACCGCCCGTCGTTATCGAAGATGGAATTGTGTGC-GAACGAGC--
AACAAGCGAGTGAGCGCATAGTTCTAGATGTGATAAAAG-
TCGTAACAAGGCAACTGTAGGAGAACCTGTAGTTGGATCATACAGATTTATAT-----
TAAGTAGTTTTTTATATTAAATT-----CCTTA--------CGCAAGGG---ATCGTTTGGTTCTA--
TGTACGATGAAGGTCGGAGCAGTATTCGATAATATAAAATGATTGTTTAAATTGTTTTATGTTACTGAATAGA
GTA-GTGATGCTCGATTACTCCTTTGAATTAAGCATATGAGTAAAGGAAGGAGAAGAAACTAAC-
AAGGATTCTCTTATTAGTGGCGAATGAACAGAGAAGAGCCC-TAAGTGTAATCAAATTCTATGAAT-------
-TTGAGATGTCAGGTATA-ATGTGGT-----
GTGAATGTATTGGAAAATACAGCCTAAGAGAGTTATAGCCTCGTAACA----
CCATTTTGTAGGAATGAGTAGTTGTGCTCGGTAATGCACAATG-
AAATAGGTGGTAGTGTCCATCTAAGGCTAAATATGACATAGAGACCGATAGTGAATAA-----
GTAAAGTGATCGAAAATGGAATAGA
>DVIL_ALP30-10
AGC-TATATGTGGAGCGGTGAAAGGCTCAGTAACGGGCGATTTATTTAATC-TCCTGGGA-------
CGGACAACATCGG-GAAACTGATGGGAAAACGTCTAAGTTGCATTAA---
TATTAGTTAGTGTGACGTAAACG-----------
GTAATCGTGCAGGAGAGTAAGATGCCATCCTATCAGTTAGTAAGTAGGGTAAGGGCCTACTTAGACGAAGACG
GGTACGGGGAATGAGGGTTTGATTCCG--
GAGAGGGAGCCTGAGAGATGGCTACCAGGACCAAGGTCAGCAGCAGGCGCGAAAATTATCGAAGCCCGCC-
TAGGGGCGATAGTGAGGAGACGTGTAT--AACGAAGTACGTGTAAAGAACGTACTAATAACT---
GGAGGTCAAGTCTGGTGCCAGCATCCGCGGTAATTCCAGCTCCAGGGGTGTCTATGATGATTGCTGCGATTAA
AAAGTCCGTAGTCAAACTGACTG-ACTGACCTGCAATGTGAT-TG------
ATTAAAGAACGAGCAGGGTTAGGAAAGCAGAGAATTAGGAGCGACCGAGGGCTAGAGTATTGAATGGCGAGAG
GTGAAATTTGATGACCCAT-
TCAGGAGTGACAAAGGCGAAGGCACTAGTCAAGGGCGAATCCGATGATCAAGGACGTAGGCTAGAGTTTCGAA
AACGATT--AGAGACCGGAGTAGTTCTAGCAGTAAACTATGCCGACGCCGTGATATT--
GTTTTTTGTGGCAGTATTGCGGAAGAGAAATC--AAGT-
AAGGCTTTGGGGAGAGTACGCGCGCAAGCGATAAATTTAAAGGAAATTGACGGAGGAACACCACAAGGAGTGG
AGTGTGCGGCTTAATTTGACTCAACGCGGGACAGCTTACCAGGCCCGATAATCGAGCGAGCGTAGTACGCGAT
AGATTAAAGAGTGGTGCATGGCTGCTATCGACAGTTGGGGTGACC-TTAGGGTTAATTCCG-
GCAAGTAGTGAGACCCCTGCAGATAG---------TGGACAGGTATTTTT--AAAATACAGGAAGG-
AAGGGACAAGAGCA-GGTCAGTGATGCCCTTAGATGGCCTGGGCT----GCACGCGCACTACAGTGG-
TCATTATAAGTAGAAA-------TT-AGA-AATAAAAA-----------TGATCGAGAGGGA-
CTGAGCTTTG-TAAGAGGCTCACGAACGAGGAATT-
GCTAGTAATCGTAGGCTCATTAAGATACGATGAATATGTCCCTGTACCTT---
GTACACACCGCCCGTCGTTATCGAAGATGGAATTGTGTGC-GAACGAGC--
AACAAGCGAGTGAGCGCATAGTTCTAGATGTGATAAAAG-
TCGTAACAAGGCAACTGTAGGAGAACCTGTAGTTGGATCATACAGATTTATAT-----
TAAGTAGTTTTTTATATTAAATT-----CCTTA--------CGCAAGGG---ATCGTTTGGTTCTA--
TGTACGATGAAGGTCGGAGCAGTATTCGATAATATAAAATGATTGTTTAAATTGTTTTATGTTACTGAATAGA
GTA-GTGATGCTCGATTACTCCTTTGAATTAAGCATATGAGTAAAGGAAGGAAAAGAAACTAAC-
AAGGATTCTCTTATTAGTGGCGAATGAACAGAGAAGAGCCC-TAAGTGTAATCAAATTCTATGAAT-------
-TTGAGATGTCAGGTATA-ATGTGGT-----
GTGAATGTATTGGAAAATACAGCCTAAGAGAGTTATAGCCTCGTAACA----
CCATTTTGTAGGAATGAGTAGTTGTGCTCGGTAATGCACAATG-
AAATAGGTGGTAGTGTCCATCTAAGGCTAAATATGACATAGAGACCGATAGTGAATAA-----
GTAAAGTGATCGAAAATGGAATAGA
>DVIL_ALP31-37
AGC-TATATGTGGAGCGGTGAAAGGCTCAGTAACGGGCGATTTATTTAATC-TCCTGGGA-------
CGGACAACATCGG-GAAACTGATGGGAAAACGTCTAAGTTGCATTAA---
TATTAGTTAGTGTGACGTAAACG-----------
GTAATCGTGCAGGAGAGTAAGATGCCATCCTATCAGTTAGTAAGTAGGGTAAGGGCCTACTTAGACGAAGACG
GGTACGGGGAATGAGGGTTTGATTCCG--
GAGAGGGAGCCTGAGAGATGGCTACCAGGACCAAGGTCAGCAGCAGGCGCGAAAATTATCGAAGCCCGCC-

TAGGGGCGATAGTGAGGAGACGTGTAT--AACGAAGTACGTGTAAAGAACGTACTAATAACT---
GGAGGTCAAGTCTGGTGCCAGCATCCGCGGTAATTCCAGCTCCAGGGGTGTCTATGATGATTGCTGCGATTAA
AAAGTCCGTAGTCAAACTGACTG-ACTGACCTGCAATGTGAT-TG------
ATTAAAGAACGAGCAGGGTTAGGAAAGCAGAGAATTAGGAGCGACCGAGGGCTAGAGTATTGAATGGCGAGAG
GTGAAATTTGATGACCCAT-
TCAGGAGTGACAAAGGCGAAGGCACTAGTCAAGGGCGAATCCGATGATCAAGGACGTAGGCTAGAGTTTCGAA
AACGATT--AGAGACCGGAGTAGTTCTAGCAGTAAACTATGCCGACGCCGTGATATT--
GTTTTTTGTGGCAGTATTGCGGAAGAGAAATC--AAGT-
AAGGCTTTGGGGAGAGTACGCGCGCAAGCGATAAATTTAAAGGAAATTGACGGAGGAACACCACAAGGAGTGG
AGTGTGCGGCTTAATTTGACTCAACGCGGGACAGCTTACCAGGCCCGATAATCGAGCGAGCGTAGTACGCGAT
AGATTAAAGAGTGGTGCATGGCTGCTATCGACAGTTGGGGTGACC-TTAGGGTTAATTCCG-
GCAAGTAGTGAGACCCCTGCAGATAG---------TGGACAGGTATTTTT--AAAATACAGGAAGG-
AAGGGACAAGAGCA-GGTCAGTGATGCCCTTAGATGGCCTGGGCT----GCACGCGCACTACAGTGG-
TCATTATAAGTAGAAA-------TT-AGA-AATAAAAA-----------TGATCGAGAGGGA-
CTGAGCTTTG-TAAGAGGCTCACGAACGAGGAATT-
GCTAGTAATCGTAGGCTCATTAAGATACGATGAATATGTCCCTGTACCTT---
GTACACACCGCCCGTCGTTATCGAAGATGGAATTGTGTGC-GAACGAGC--
AACAAGCGAGTGAGCGCATAGTTCTAGATGTGATAAAAG-
TCGTAACAAGGCAACTGTAGGAGAACCTGTAGTTGGATCATACAGATTTATAT-----
TAAGTAGTTTTTTATATTAAATT-----CCTTA--------CGCAAGGG---ATCGTTTGGTTCTA--
TGTACGATGAAGGTCGGAGCAGTATTCGATAATATAAAATGATTGTTTAAATTGTTTTATGTTACTGAATAGA
GTA-GTGATGCTCGATTACTCCTTTGAATTAAGCATATGAGTAAAGGAAGGAAAAGAAACTAAC-
AAGGATTCTCTTATTAGTGGCGAATGAACAGAGAAGAGCCC-TAAGTGTAATCAAATTCTATGAAT-------
-TTGAGATGTCAGGTATA-AAGTGGT-----
GTGAATGTATTGGAAAATACAGCCTAAGAGAGTTATAGCCTCGTAACA----
CCATTTTGTAGGAATGAGTAGTTGTGCTCGGTAATGCACAATG-
AAATAGGTGGTAGTGTCCATCTAAGGCTAAATATGACATAGAGACCGATAGTGAATAA-----
GTAAAGTGATCGAAAATGGAATAGA
>DVIL_FREXP-C5
AGC-TATATGTGGAGCGGTGAAAGGCTCAGTAACGGGCGATTTATTTAATC-TCCTGGGA-------
CGGACAACATCGG-GAAACTGATGGGAAAACGTCTAAGTTGCATTAA---
TATTAGTTAGTGTGACGTAAACG-----------
GTAATCGTGCAGGAGAGTAAGATGCCATCCTATCAGTTAGTAAGTAGGGTAAGGGCCTACTTAGACGAAGACG
GGTACGGGGAATGAGGGTTTGATTCCG--
GAGAGGGAGCCTGAGAGATGGCTACCAGGACCAAGGTCAGCAGCAGGCGCGAAAATTATCGAAGCCCGCC-
TAGGGGCGATAGTGAGGAGACGTGTAT--AACGAAGTACGTGTAAAGAACGTACTAATAACT---
GGAGGTCAAGTCTGGTGCCAGCATCCGCGGTAATTCCAGCTCCAGGGGTGTCTATGATGATTGCTGCGATTAA
AAAGTCCGTAGTCAAACTGACTG-ACTGACCTGCAATGTGAT-TG------
ATTAAAGAACGAGCAGGGTTAGGAAAGCAGAGAATTAGGAGCGACCGAGGGCTAGAGTATTGAATGGCGAGAG
GTGAAATTTGATGACCCAT-
TCAGGAGTGACAAAGGCGAAGGCACTAGTCAAGGGCGAATCCGATGATCAAGGACGTAGGCTAGAGTTTCGAA
AACGATT--AGAGACCGGAGTAGTTCTAGCAGTAAACTATGCCGACGCCGTGATATT--
GTTTTTTGTGGCAGTATTGCGGAAGAGAAATC--AAGT-
AAGGCTTTGGGGAGAGTACGCGCGCAAGCGATAAATTTAAAGGAAATTGACGGAGGAACACCACAAGGAGTGG
AGTGTGCGGCTTAATTTGACTCAACGCGGGACAGCTTACCAGGCCCGATAATCGAGCGAGCGTAGTACGCGAT
AGATTAAAGAGTGGTGCATGGCTGCTATCGACAGTTGGGGTGACC-TTAGGGTTAATTCCG-
GCAAGTAGTGAGACCCCTGCAGATAG---------TGGACAGGTATTTTT--AAAATACAGGAAGG-
AAGGGACAAGAGCA-GGTCAGTGATGCCCTTAGATGGCCTGGGCT----GCACGCGCACTACAGTGG-
TCATTATAAGTAGAAA-------TT-AGA-AGTAAAAA-----------TGATCGAGAGGGA-
CTGAGCTTTG-TAAGAGGCTCACGAACGAGGAATT-
GCTAGTAATCGTAGGCTCATTAAGATACGATGAATATGTCCCTGTACCTT---
GTACACACCGCCCGTCGTTATCGAAGATGGAATTGTGTGC-GAACGAGC--
AACAAGCGAGTGAGCGCATAGTTCTAGATGTGATAAAAG-
TCGTAACAAGGCAACTGTAGGAGAACCTGTAGTTGGATCATACAGATTTATTT-----
tAAGTAGTTTTTTATATTAAATT-----CCTTA--------CGCAAGGG---ATCGTTTGGTTCTA--
TGTACGATGAAGGTCGGAGCAGTATTCGATAATATAAAATGATTGTTTAAATTGTTTTATGTTACTGAATAGA
GTA-GTGATGCTCGATTACTCCTTTGAATTAAGCATATGAGTAAAGGAAGGAAAAGAAACTAAC-
AAGGATTCTCTTATTAGTGGCGAATGAACAGAGAAGAGCCC-TAAGTGTAATCAAATTCTATGAAT-------

-TTGAGATGTCAGGTATA-AAGTGGT-----
GTGAATGTATTGGAAAATACAGCCTAAGAGAGTTATAGCCTCGTAACA----
CCATTTTGTAGGAATGAGTAGTTGTGCTCGGTAATGCACAATG-
AAATAGGTGGTAGTGTCCATCTAAGGCTAAATATGACATAGAGACCGATAGTGAATAA-----
GTAAAGTGATCGAAAATGGAATAGA
>GROE_ALPM41-1
AGC-TATATGTGGAGCGGTGAAAGGCTCAGTAACGGGCGATTTATTTAATC-TCCTGGGA-------
CGGACAACATCGG-GAAACTGATGGGAAAACGTCTAAGTTGCATTAA---T----
GTTAGTGTGACGTAAACG-----------
GTAATCGTGCAGGAGAGTAAGATGCCATCCTATCAGTTAGTAAGTAGGGTAAGGGCCTACTTAGACGAAGACG
GGTACGGGGAATGAGGGTTTGATTCCG--
GAGAGGGAGCCTGAGAGATGGCTACCAGGACCAAGGTCAGCAGCAGGCGCGAAAATTATCGAAGCCCGCC-
TAGGGGCGATAGTGAGGAGACGTGTAT--AACGAAGTACGTGTAAAGAACGTACTAATAACT---
GGAGGTCAAGTCTGGTGCCAGCATCCGCGGTAATTCCAGCTCCAGGGGTGTCTATGATGATTGCTGCGATTAA
AAAGTCCGTAGTCAAACTGACTG-ACTGACCTGCAATGTGAT-TG------
ATTAAAGAACGAGCAGGGTTAGGAAAGCAGAGAATTAGGAGCGACCGAGGGCTAGAGTATTGAATGGCGAGAG
GTGAAATTTGATGACCCAT-
TCAGGAGTGACAAAGGCGAAGGCACTAGTCAAGGGCGAATCCGATGATCAAGGACGTAGGCTAGAGTTTCGAA
AACGATT--AGAGACCGGAGTAGTTCTAGCAGTAAACTATGCCGACGCCGTGATATT--
GTTTTTTGTGGCGGTATTGCGGAAGAGAAATC--AAGT-
AAAGCTTTGGGGAGAGTACGCGCGCAAGCGATAAATTTAAAGGAAATTGACGGAGGAACACCACAAGGAGTGG
AGTGTGCGGCTTAATTTGACTCAACGCGGGACAGCTTACCAGGCCCGATAATCGAGCGAGCGTAGTACGCGAT
AGATTAAAGAGTGGTGCATGGCTGCTATCGACAGTTGGGGTGACC-TTAGGGTTAATTCCG-
GCAAGTAGTGAGACCCCTGCAGATAG---------TGGACAGGTATTTTT--AAAATACAGGAAGG-
AAGGGACAAGAGCA-GGTCAGTGATGCCCTTAGATGGCCTGGGCT----GCACGCGCACTACAGTGG-
TCATTATAAGTAGAAA-------TT-AGA-AGTAAAAA-----------TGATCGAGAGGGA-
CTGAGCTTTG-TAAGAGGCTCACGAACGAGGAATT-
GCTAGTAATCGTAGGCTCATTAAGATACGATGAATATGTCCCTGTACCTT---
GTACACACCGCCCGTCGTTATCGAAGATGGAATTGTGTGC-GAACGAGC--
AACAAGCGAGTGAGCGCATAGTTCTAGATGTGATAAAAG-
TCGTAACAAGGCAACTGTAGGAGAACCTGTAGTTGGATCATACAGATTTATAT-----
TAAGTAGTTTTTTATATTAAATT-----CCTTA--------CGCAAGGG---ATCGTTTGGTTCTA--
TGTACGATGAAGGTCGGAGCAGTATTCGATAATATAAGATGATTGTTTAAATTGTTTTATGTTACTGAATAGA
GTA-GTGATGCTCGATTACTCCTTTGAATTAAGCATATGAGTAAAGGAAGGAAAAGAAACTAAC-
AAGGATTCTCTTATTAGTGGCGAATGAACAGAGAAGAGCCC-TAAGTGTAATCAAATTCTATGAAT-------
-TTGAGATGTCAGGTATA-ATGTGGT-----
GTGAATGTATTGGAAAATACAGCCTAAGAGAGTTATAGCCTCGTAACA----
CCATTTTGTAGGAATGAGTAGTTGTGCTCGGTAATGCACAATG-
AAATAGGTGGTAGTGTCCATCTAAGGCTAAATATGACATAGAGACCGATAGTGAATAA-----
GTAAAGTGATCGAAAATGGAATAGA
>GROE_ALPM41-2
AGC-TATATGTGGAGCGGTGAAAGGCTCAGTAACGGGCGATTTATTTAATC-TCCTGGGA-------
CGGACAACATCGG-GAAACTGATGGGAAAACGTCTAAGTTGCATTAA---T----
GTTAGTGTGACGTAAACG-----------
GTAATCGTGCAGGAGAGTAAGATGCCATCCTATCAGTTAGTAAGTAGGGTAAGGGCCTACTTAGACGAAGACG
GGTACGGGGAATGAGGGTTTGATTCCG--
GAGAGGGAGCCTGAGAGATGGCTACCAGGACCAAGGTCAGCAGCAGGCGCGAAAATTATCGAAGCCCGCC-
TAGGGGCGATAGTGAGGAGACGTGTAT--AACGAAGTACGTGTAAAGAACGTACTAATAACT---
GGAGGTCAAGTCTGGTGCCAGCATCCGCGGTAATTCCAGCTCCAGGGGTGTCTATGATGATTGCTGCGATTAA
AAAGTCCGTAGTCAAACTGACTG-ACTGACCTGCAATGTGAT-TG------
ATTAAAGAACGAGCAGGGTTAGGAAAGCAGAGAATTAGGAGCGACCGAGGGCTAGAGTATTGAATGGCGAGAG
GTGAAATTTGATGACCCAT-
TCAGGAGTGACAAAGGCGAAGGCACTAGTCAAGGGCGAATCCGATGATCAAGGACGTAGGCTAGAGTTTCGAA
AACGATT--AGAGACCGGAGTAGTTCTAGCAGTAAACTATGCCGACGCCGTGATATT--
GTTTTTTGTGGCGGTATTGCGGAAGAGAAATC--AAGT-
AAGGCTTTGGGGAGAGTACGCGCGCAAGCGATAAATTTAAAGGAAATTGACGGAGGAACACCACAAGGAGTGG
AGTGTGCGGCTTAATTTGACTCAACGCGGGACAGCTTACCAGGCCCGATAATCGAGCGAGCGTAGTACGCGAT
AGATTAAAGAGTGGTGCATGGCTGCTATCGACAGTTGGGGTGACC-TTAGGGTTAATTCCG-

GCAAGTAGTGAGACCCCTGCAGATAG---------TGGACAGGTATTTTT--AAAATACAGGAAGG-
AAGGGACAAGAGCA-GGTCAGTGATGCCCTTAGATGGCCTGGGCT----GCACGCGCACTACAGTGG-
TCATTATAAGTAGAAA-------TT-AGA-AGTAAAAA-----------TGATCGAGAGGGA-
CTGAGCTTTG-TAAGAGGCTCACGAACGAGGAATT-
GCTAGTAATCGTAGGCTCATTAAGATACGATGAATATGTCCCTGTACCTT---
GTACACACCGCCCGTCGTTATCGAAGATGGAATTGTGTGC-GAACGAGC--
AACAAGCGAGTGAGCGCATAGTTCTAGATGTGATAAAAG-
TCGTAACAAGGCAACTGTAGGAGAACCTGTAGTTGGATCATACAGATTTATAT-----
TAAGTAGTTTTTTATATTAAATT-----CCTTA--------CGCAAGGG---ATCGTTTGGTTCTA--
TGTACGATGAAGGTCGGAGCAGTATTCGATAATATAAGATGATTGTTTAAATTGTTTTATGTTACTGAATAGA
GTA-GTGATGCTCGATTACTCCTTTGAATTAAGCATATGAGTAAAGGAAGGAAAAGAAACTAAC-
AAGGATTCTCTTATTAGTGGCGAATGAACAGAGAAGAGCCC-TAAGTGTAATCAAATTCTATGAAT-------
-TTGAGATGTCAGGTATA-ATGTGGT-----
GTGAATGTATTGGAAAATACAGCCTAAGAGAGTTATAGCCTCGTAACA----
CCATTTTGTAGGAATGAGTAGTTGTGCTCGGTAATGCACAATG-
AAATAGGTGGTAGTGTCCATCTAAGGCTAAATATGACATAGAGACCGATAGTGAATAA-----
GTAAAGTGATCGAAAATGGAATAGA
>KX364285_Unikaryon legeri
AGCAGTTATGTGGAGCGGTAAACGGCTCAGTAATAGGCGATTTATTTGTTC-TTTTGGGA-------
TGGACAACATCGG-GAAACTGATGGGAAAACATCTAAGATGCATTTT---
TTTAATGAGATGTGTCGTAAACC-----------
TTCTGGTGCAAGAGAGTAAGACGCCTTCCTATCAGTTAGTAAGTAGGGTAAGGGCCTACTTAGACGAAGACGG
GTACGGGGAATGAGGGTTTGATTCCG--
GAGAGGGAGCCTGAGAGATGGCTACCAGGACCAAGGTCAGCAGCAGGTGCGAAACTTATCGAAGCCCGCA-
TTGGGGCGATAGTGAGGAGACGTGATA---ACTATGGACGTGTAAAAACCATTCTAATAACT---
GGAGGCCAAGTCTGGTGCCAGCAGTCGCGGTAATTCCAGCTCCAGTAGTGTCTATGATGATTGCTGCGATTAA
AAAGTCCGTAGTCTATCTGACTG-ACTTGCCTGTAATGTGAT-TG------
ATTAAAAGACGAGTAGGGCTAGGAAAGCAGAGAATTAGGAGCGACCAAGGGCTAGAGTATTGAATGGCGAGGG
GTGAAATCTGATGACCCAT-
TCAGGAGTGACAGAGGCGAAAGCGCTAGTCAGGGGCGAATCCGATGATCAAGGACGTAGGCTAGAGATTCGAA
AACGATT--AGAGACCGGAGTAGTTCTAGCAGTAAACTATGCCGACGCCGTGGTATT--TTTGT-----
ATAGTGCTGCGGAAGAGAAATC--AAGT-
AAGGCTTTGGGGAGAGTACGCGCGCAAGCGATAAATTTAAAGGAAATTGACGGAGGAACACCCACAAGGAGTGG
AGTGTGCGGCTTAATTTGACTCAACGCGGGACAGCTTACCAAGCCCGATAACCGTAAGAGCGTTGTACGCGAT
AGGTTAAAGAGTGGTGCATGGCTGCCATCAACAGCTGAGGTGACT-TTAGGGTTAATTCCG-
GCAAGTAGTGAGACCCCTATAATTGAA--------TAGACAGGTGTCTTT--AAGACACAGGAAGG-
AAGGGACTAGAGCA-GGTCAGTGATGCCCTTAGATGGCTTGGGCT----GCACGCGCACTACAGTGG-
TCATTATAAAAAGAAA---GACAGT-AGA-TATAAAGG-----------TGATCGAGAGGGA-
TTGAGCTTTG-TAAAAGGCTCATGAACGAGGAATT-
GCTAGTAGTCGTAGGCTCATTAAGATACGACGAATATGTCCCTGTACCTT---
GTACACACCGCCCGTCGTTATCAAAGATGAAATTTAGTGC-GAACGAGC--
ATTAAGCGAGTAAGCGCTAAGTTTTAGATCTGATAAAGG-
TCGTAACAAGGCTACTGTAGGAGAACCTGTAGTAGGATCATACAGATAACAAGTTTATAAGTATTGTTTTGTA
TATTTATT-----CCTTA--------CGCAAGGG---ATCGTTTGGTTCTA--
TGTGCGATGAAGGGCGAAACAGTTTTCGATATTGTAAGATATTAGTA--
AATTGTCTTACGTCCCCGAATTTGATA-
GAGATATCAGATTACTCCTTTGAATTAAGCATATGAGTAAAGGAAGGAAAAGAAACTAAC-
TAGGATTCTCTTATTAGTGGCGAATGAACAGAGAAGAGCCC--AAGTGTAATCAATACCAATATATG-----
TATTGAATTGTCATATATA-ATATAATT-
TAATTGAATCTGTTGGAATACAGAGCTTTAAAGAGTTATAGCCTCGTAGATTAAATTAATTTGTATCAA-
GAGTAGTTGTGCTCGGTAATGCACAATGAAAATAGGTGGTAGTGTCCATCTAAGGCTAAATATTACATAGAGA
CCGATAGTGGATAA-----GTAATGTGAACGAATATGGAATAGA
>KF537632_Ddip
AGC-GTAACGTGGAGCGGTGAAAGGCTCAGTAACGGGCGATTTATTTGATC-TCCTGGGA-------
TGGACAACACCGG-GAAACTGGTGGGAAAACATCTAAGTTGCGG------
TTCTATTGATCGTGATGTAAACCT----------
ATGTGGGTGCAGGAGAGTAAGATGCCATCCTATCAGTTAGTAAGTAGGGTAAGGGCCTACTTAGACGAAGACG
GGTACGGGGAATGAGGGTTTGATTCCG--

```
GAGAGGGAGCCTGAGAGATGGCTACCAGGACCAAGGTCAGCAGCAGGCGCGAAAATTATCGAAGCCCACCATT
GGGGCGATAGTGAGGAGACGTGTAT--AACGAAATACGGGTAAAGAACGTATGTATAACT---
GGAGGTCAAGTCTGGTGCCAGCATCCGCGGTAATTCCAGCTCCAGGGGTGTCTATGATGATTGCTGCGATTAA
AAAGTCCGTAGTCAAGCTGACTG-ACTAACCTGTAATGTGGT-TG------
ATTAAAAGACGAGAAGGGTTAGGAAAGCAGAGGATTAGGAGCGACCGAGGGCTAGAGTATTGAATGGCGAGAG
GTGAAATTTGATGACCCAT-
TCAGGAGTGACAAAGGCGAAGGCACTAGTCAAGGGCGAATCCGATGATCAAGGACGTAGGCTAGAGGTTCGAA
AACGATT--AGAGACCGGAGTAGTTCTAGCAGTAAACTATGCCGACGCCGTGGTATG--TTTTTT----
AATGTATTGCGGAAGAGAAATC--AAGT-
AAGGCTTTGGGGAGAGTACGCGCGCAAGCGATAAATTTAAAGGAAATTGACGGAGGAACACCACAAGGAGTGG
AGTGTGTGGCTTAATTTGACTCAACGCGGGACAGCTTACCAGGCCCGATAATCGTACGAGCGTAGTACGCGAT
AGGTTAAAGAGTGGTGCATGGCTGCTATCGACAGTTGGGGTGACC-TTAGGGTTAATTCCG-
GCAAGTAGTGAGACCCCTGCTATTAAA-------TAGGACAGGTATTTTT--AAAATACAGGAAGG-
AAGGGACAAGAGCA-GGTCGGTGATGCCCTTAGATGGCCTGGGCT----GCACGCGCACTACAGTGG-
TCATTATAATGAGTAA-------AT-AGA-AGTAAAAA-----------TGATCGAGAGGGA-
CTGGGCTTTG-TAAGAGGCCCACGAACGAGGAATT-
GCTAGTAATCGTAGGCTCATTAAGATACGATGAATATGTCCCTGTACCTT---
GTACACACCGCCCGTCGTTATCGAAGATGGAATTGTGTGC-GAACGAGC--
ATTAAGCGAGTGAGCGCATAGTTCTAGATGTGATAAAAG-
TCGTAACAAGGCAACTGTAGGAGAACCTGTAGTTGGATCATACAGATTTTATTA-AATTGACAAGTTTTTT-
TATAATTTT-----CCTTA--------CGCAAGGG---ATCGTTTGGTTCTA--
TGTACGATGAAGGTCGGAGCAGTATCTGATAATATTAAATGATTGTA-
AAATTGTTTAATGTTACTGAATTAAGGTTAAAAACCTTGAATACTCCTTTGAATTAAGCATATGAGTAAAGGA
AGGAAAAGAAACTAAC-AAGGATTCTCTTATTAGTGGCGAATGAACAGAGAAGAGCCC--
CAGTGTAATCACTTTAAATTTATAATTTTTAGTGAGTTGTCTAGCAG--ATAGGAC-----
GTGAAAATAATGGAAAGTATTGCCGCAGAGAGTGATAGCCTCGTAACGTC--
TAATGAATTGGGATTGAGTAGTTGTGCTCGGTAATGCACAATG-
AATTTGGTGGTAGTGTCCATCAAAGGCTAAATATGACATAGAGACCGATAGAGAAAAA-----
GTAGAGTGATCGAAAATGAAG-AGA
>AJ438955_Dmue
AGC-TATATGTGGAGCGGTGAAAGGCTCAGTAACGGGCGATTTATTTAATC-TCCTGGGA-------
CGGACAACATCGG-GAAACTGATGGGAAAACGTCTAAGTTGCATTAA---
TATTAGTTAGTGTGACGTAAACG-----------
GTAATCGTGCAGGAGAGTAAGATGCCATCCTATCAGTTAGTAAGTAGGGTAAGGGCCTACTTAGACGAAGACG
GGTACGGGGAATGAGGGTTTGATTCCG--
GAGAGGGAGCCTGAGAGATGGCTACCAGGACCAAGGTCAGCAGCAGGCGCGAAAATTATCGAAGCCCGCC-
TAGGGGCGATAGTGAGGAGACGTGTAT--AACGAAGTACGTGTAAAGAACGTACTAATAACT---
GGAGGTCAAGTCTGGTGCCAGCATCCGCGGTAATTCCAGCTCCAGGGGTGTCTATGATGATTGCTGCGATTAA
AAAGTCCGTAGTCAAACTGACTG-ACTGACCTGCAATGTGAT-TG------
ATTAAAGAACGAGCAGGGTTAGGAAAGCAGAGAATTAGGAGCGACCGAGGGCTAGAGTATTGAATGGCGAGAG
GTGAAATTTGATGACCCAT-
TCAGGAGTGACAAAGGCGAAGGCACTAGTCAAGGGCGAATCCGATGATCAAGGACGTAGGCTAGAGTTTCGAA
AACGATT--AGAGACCGGAGTAGTTCTAGCAGTAAACTATGCCGACGCCGTGATATT--
GTTTTTTGTGGCAGTATTGCGGAAGAGAAATC--AAGT-
AAGGCTTTGGGGAGAGTACGCGCGCAAGCGATAAATTTAAAGGAAATTGACGGAGGAACACCACAAGGAGTGG
AGTGTGCGGCTTAATTTGACTCAACGCGGGACAGCTTACCAGGCCCGATAATCGAGCGAGCGTAGTACGCGAT
AGATTAAAGAGTGGTGCATGGCTGCTATCGACAGTTGGGGTGACC-TTAGGGTTAATTCCG-
GCAAGTAGTGAGACCCCTGCAGATAG---------TGGACAGGTATTTTT--AAAATACAGGAAGG-
AAGGGACAAGAGCA-GGTCAGTGATGCCCTTAGATGGCCTGGGCT----GCACGCGCACTACAGTGG-
TCATTATAAGTAGAAA-------TT-AGA-AGTAAAAA-----------TGATCGAGAGGGA-
CTGAGCTTTG-TAAGAGGCTCACGAACGAGGAATT-
GCTAGTAATCGTAGGCTCATTAAGATACGATGAATATGTCCCTGTACCTT---
GTACACACCGCCCGTCGTTATCGAAGATGGAATTGTGTGC-GAACGAGC--
AACAAGCGAGTGAGCGCATAGTTCTAGATGTGATAAAAG-
TCGTAACAAGGCAACTGTAGGAGAACCTGTAGTTGGATCATACAGATTTATAT-----
TAAGTAGTTTTTTATAATAAATT-----CCTTA--------CGCAAGGG---ATCGTTTGGTTCTA--
TGTACGATGAAGGTCGGAGCAGTATTCGATAATATAAGATGATTGTTTAAATTGTTTTATGTTACTGAATAAA
GTA-
```

GTGATGCTCGATTACTCCTTTGAATTAAGCATATGAGTAAAGGAGG???????????????????????????
???
???
???
???
>AJ438956_Dmue
AGC-TATATGTGGAGCGGTGAAAGGCTCAGTAACGGGCGATTTATTTAATC-TCCTGGGG-------
CGGACAACATCGG-GAAACTGATGGGAAAACGTCTAAGTTGCATTAA---T----
TTTAGTGTGACGTAAACG-----------
ATTATCGTGCAGGAGAGTAAGATGCCATCCTATCAGTTAGTAAGTAGGGTAAGGGCCTACTTAGACGAAGACG
GGTACGGGGAATGAGGGTTTGATTCCG--
GAGAGGGAGCCTGAGAGATGGCTACCAGGACCAAGGTCAGCAGCAGGCGCGAAAATTATCGAAGCCCGCC-
TAGGGGCGATAGTGAGGAGACGTGTAT--AACGAAGTACGTGTAAAGAACGTACTAATAACT---
GGAGGTCAAGTCTGGTGCCAGCATCCGCGGTAATTCCAGCTCCAGGGGTGTCTATGATGATTGCTGCGATTAA
AAAGTCCGTAGTCAAGCTGCCTG-ACTGACCTGCAATGTGAT-TG------
ATTAAGGAACGAGCAGGGTTAGGAAAGCAGAGAATTAGGAGCGACCGAGGGCTAGAGTATTGAATGGCGAGAG
GTGAAATTTGATGACCCAT-
TCAGGAGTGACAAAGGCGAAGGCACTAGTCAAGGGCGAATCCGATGATCAAGGACGTAGGCTAGAGTTTCGAA
AACGATT--AGAGACCGGAGTAGTTCTAGCAGTAAACTATGCCGACGCCGTGGTATG--
GTTTTTTGTGGCTGTATTGCGGAAGAGAAATC--AAGT-
AAGGCTTTGGGGAGAGTACGCGCGCAAGCGATAAATTTAAAGGAAATTGACGGAGGAACACCACAAGGAGTGG
AGTGTGCGGCTTAATTTGACTCAACGCGGGACAGCTTACCAGGCCCGATAATCGAGCGAGCGTTGTACGCGAT
AGATTAAAGAGTGGTGCATGGCTGCTATCGACAGTTGGGGTGACC-TTAGGGTTAATTCCG-
GCAAGTAGTGAGACCCCTGCAGATAG---------TGGACAGGTATTTTT--AAAATACAGGAAGG-
AAGGGACAAGAGCA-GGTCAGTGATGCCCTTAGATGGCCTGGGCT----GCACGCGCACTACAGTGG-
TCATTATAAGTAGAAG-------TT-AGA-AGTAAAGA-----------TGATCGAGAGGGA-
CTGAGCTTTG-TAAGAGGCTCACGAACGAGGAATT-
GCTAGTAATCGTAGGCTCATTAAGATACGATGAATATGTCCCTGTACCTT---
GTACACACCGCCCGTCGTTATCGAAGATGGAATTGTGTGC-GAACGAGC--
AACAAGCGAGTGAGCGCATAGTTCTAGATGTGATAAAAG-
TCGTAACAAGGCAACTGTAGGAGAACCTGTAGTTGGATCATACAGATTTATTT-----CAAGTAGTTTG---
TAATAAATT-----CCTTA--------CGCAAGGG---ATCGTTTGGCTCTA--
TGTGCGATGAAGGTCGGAGCAGTATTCGATAATATAAGATGATTGTATAAATTGTCTTATGTTACTGAATAGA
GTA-
GTGATGCTCGATTACTCCTTTGAATTAAGCATA??
???
???
???
???
>AJ438961_Ddes
AGC-GTAACGTGGAGCGGTGAAAGGCTCAGTAACGGGCGATTTATTTGATC-TCCTGGGA-------
CGGACAACACCGG-GAAACTGGTGGGAATACGTCTAAGCTGCGGGTCA-
CGGCATGTGATTGCGGCGGAAACA--------------
GCAGTGCAGGAGAGTAAGGTGCCATCCTATCAGTTAGTAAGTAGGGTAAGGGCCTACTTAGACGAAGACGGGT
ACGGGGAATGAGGGTTTGATTCCG--
GAGAGGGAGCCTGAGAGACGGCTACCAGGACCAAGGTCAGCAGCAGGCGCGAAAATTATCGAAGCCCGCC-
TAGGGGCGATAGTGAGGAGACGTGAAT---TGTAGGTGCGGGTAAAAAACGCACTAGCAACT---
GGAGGTCAAGTCTGGTGCCAGCATCCGCGGTAATTCCAGCTCCAGGGGTGTCTATGATGATTGCTGCGATTAA
AAAGTCCGTAGTCAAGCCGCCAG-ACCAGTCTGGAATGTTTC-TG------
ATCAAGGGACGAGCAGGGCTGGGAAAGCGGAGAATTAGGAGCGACCGAGGGCTAGAGTATTGGGTGGCGAGAG
GTGAAATTTGATGACCCAT-
CCAGGAGTGACAAAGGCGAAGGCACTAGTCAAGGGCGAATCCGATGATCAAGGACGTAGGCTAGAGGTTCGAA
AACGATT--AGAGACCGGAGTAGTTCTAGCAGTAAACTATGCCGACGCCGCGATATG-AGGTTT------
TTGTATTGCGGAAGAGAAATC--AAGT-
AAGGCTTTGGGGAGAGTACGCGCGCAAGCGATAAATTTAAAGGAAATTGACGGAGGAACACCACAAGGAGTGG
AGTGTGCGGCTTAATTTGACTCAACGCGGGACAGCTTACCAGGCCCGATAACTAAGCGAGCGTAGTACGCGAT
AGGTTAGAGAGTGGTGCATGGCTGCTATCGACAGTTGGGGTGACC-TTAGGGTTAATTCCG-
GCAAGTAGTGAGACCCCTGCAGGTA----------TGGACAGGCATTGTG--AAGATGCAGGAAGG-

AGGGGACAAGAGCA-GGTCAGTGATGCCCTTAGATGGCCTGGGCT----GCACGCGCACTACAGTGG-
TCATTATAGGAAGAGA-------GT-AGA-GACAAAGA-----------TGATCGAGAGGGA-
CTGGGCTTTG-TAAGAGGCCCACGAACGAGGAATT-
GCTAGTAATCGCAGGCTCATTAAGATGCGATGAATATGTCCCTGTACCTT---
GTACACACCGCCCGTCGTTATCGAAGATGGAATTGTATGC-GAACGAGC--
AGCAAGCGAGTGAGCGTATAGTTCTAGATGTGATAAAAG-
TCGTAACAAGGCAACTGTAGGAGAACCTGTAGTTGGATCAGACAGCTTTATTTA----
TGTTTAGTTTTATAGTTAGTAAT-----CCCTG--------CGCAAGGG---ATCGTTAGGCTCTA--
TGTACGATGAAGGCCGGGGCAGCAACCGATATGGCGCAATGTATGCAGATATCGTTGTGCGTGGCCGAATTGA
ACA-
GAGATGTTCGGATACTCCTTTGAATTAAGCA??
???
???
???
???
>AJ438960_Dcav
AGC--TATTGTGGAGCGGTGAAAGGCTCAGTAACGGGCGATTTATTTAGTC-TTCTGGGA-------
CGGACAACACCGG-GAAACTGGTGGGAAAACGTCTAAGCTGCGAATCA-
CGCTATGTGGTTGTGGCAGAAACT-------------
GTTAGTGCAAAAAAGTAAGATGCCATCCTATCAGTTAGTAAGTAGGGTAAGGGCCTACTTAGACGAAGACGGG
TACGGGGAATGAGGGTTTGATTCCG--
GAGAGGGAGCCTGAGAGACGGCTACCAGGACCAAGGTCAGCAGCAGGCGCGAAAATTATCGAAGCCCGCC-
TAGGGGCGATAGTGAGGAGACGTGAAT---TTTAGGTGCGTGTAAAGAACGCACTAGCAACT---
GGAGGTCAAGTCTGGTGCCAGCATCCGCGGTAATTCCAGCTCCAGGGGTGTCTATGATGATTGCTGCGATTAA
AAAGTCCGTAGTCAAGCCGCCAG-ACCAGTCTGGAATGTTTCTTG------
ATCAAGAGACGAGCAGGGCTGGGAAAGCGGAGAATTAGGAGCGACCGAGGGCTAGAGTATTGGGTGGCGAGAG
GTGAAATTTGATGACCCAT-
CCAGGAGTGACAAAGGCGAAGGCACTAGTCAAGGGCGAATCCGATGATCAAGGACGTAGGCTAGAGTTTCGAA
AACGATT--AGAGACCGGAGTAGTTCTAGCAGTAAACTATGCCGACGCCGTGATATG--ATTTT-----
GTTGTATTGCGGAAGAGAAATC--AAGT-
AAGGCTTTGGGGAGAGTACGCGCGCAAGCGATAAATTTAAAGGAAATTGACGGAGGAACACCACAAGGAGTGG
AGTGTGCGGCTTAATTTGACTCAACGCGGGACAGCTTACCAGGCCCGATAATCGTACGAGCGTAGTACGCGAT
AGATTAGAGAGTGGTGCATGGCTGCTATCGACAGTTGGGGTGACC-TTAGGGTTAATTCCG-
GCAAGTAGTGAGACCCCCGCAGTAT----------TGGACAGGCGTCGTG--AAGATGCAGGAAGG-
AGGGGACAAGAGCA-GGTCAGTGATGCCCTTAGATGGCCTGGGCT----GCACGCGCACTACAGTGG-
TCATTATAAGGAGAAG-------TT-AGA-AATAAAGA-----------TGATCGAGAGGGA-
CTGGGCTTTG-TAAGAGGCCCACGAACGAGGAATT-
GCTAGTAATCGCAGGCTCATTAGGATGCGATGAATATGTCCCTGTACCTT---
GTACACACCGCCCGTCGTTATCGAAGATGGAATTGTATGC-GAACGAGC--
AGCAAGCGAGTGAGCGTATAGTTCTAGATGTGATAAAAG-
TCGTAACAAGGCAACTGTAGGAGAACCTGTAGTTGGATCACACAGATTTTATTTGAATTGTTTGTTTTT----
------AT-----CTCTG--------CGCAAGGG---ATCGTTAGGTTCTA--
TGTACGACGAAGGCCGGAGTAGTATCCGATATGGTGCTACAAGCGCAGTTATTGTTGCACGTTGCCTAATTGA
ACA-
GTGATGTTCGAATACTCCTTCTAATTAAGCA??
???
???
???
???
>AJ438959_Dcav
AGC--TATTGTGGAGCGGTGAAAGGCTCAGTAACGGGCGATTTATTTAGTC-TTCTGGGA-------
CGGACAACACCGG-GAAACTGGTGGGAAAACGTTTAAGCTGCGAATCA-
TGCGATGTGGTTGTGGCAGAAACT-------------
GTTAGTGCAGAAGAGTAAGATGCCATCCTATCAGTTAGTAAGTAGGGTAAGGGCCTACTTAGACGAAGACGGG
TACGGGGAATGAGGGTTTGATTCCG--
GAGAGGGAGCCTGAGAGACGGCTACCAGGACCAAGGTCAGCAGCAGGCGCGAAAATTATCGAAGCCCGCC-
TAGGGGCGATAGTGAGGAGACGTGAAT---TTTAGGTGCGTGTAAAGAACGCACTAGCAACT---
GGAGGTCAAGTCTGGTGCCAGCATCCGCGGTAATTCCAGCTCCAGGGGTGTCTATGATGATTGCTGCGATTAA

AAAGTCCGTAGTCAAGCCGCCAG-ACCAGTCTGGAATGTTTCTTG------
ATCAAGAGACGAGCAGGGCTGGGAAAGCGGAGAATTAGGAGCGACCGAGGGCTAGAGTATTGGGTGGCGAGAG
GTGAAATTTGATGACCCAT-
CCAGGAGTGACAAAGGCGAAGGCACTAGTCAAGGGCGAATCCGATGATCAAGGACGTAGGCTAGAGTTTCGAA
AACGATT--AGAGACCGGAGTAGTTCTAGCAGTAAACTATGCCGACGCCGTGATATG--ATTTT-----
GTTGTATTGCGGAAGAGAAATC--AAGT-
AAGGCTTTGGGGAGAGTACGCGCGCAAGCGATAAATTTAAAGGAAATTGACGGAGGAACACCACAAGGAGTGG
AGTGTGCGGCTTAATTTGACTCAACGCGGGACAGCTTACCAGGCCCGATAATCGTACGAGCGTAGTACGCGAT
AGATTAGAGAGTGGTGCATGGCTGCTATCGACAGTTGGGGTGACC-TTAGGGTTAATTCCG-
GCAAGTAGTGAGACCCCCGCAGTAT----------TGGACAGGCGTCGTG--AAGATGCAGGAAGG-
AGGGGACAAGAGCA-GGTCAGTGATGCCCTTAGATGGCCTGGGCT----GCACGCGCACTACAGTGG-
TCATTATAAGAAGGAA-------TT-AAA-AGTAAAGA-----------TGATCGAGAGGGA-
CTGGGCTTTG-TAAGAGGCCCACGAACGAGGAATT-
GCTAGTAATCGCAGGCTCATTAGGATGCGATGAATATGTCCCTGTACCTT---
GTACACACCGCCCGTCGTTATCGAAGATGGAATTGTATGC-GAACGAGC--
AGCAAGCGAGTGAGCGTATAGTTCTAGATGTGATAAAAG-
TCGTAACAAGGCAACTGTAGGAGAACCTGTAGTTGGATCACACAGATTTTATTTGAATTGTTTGTTTTT----
------AT-----CTCTG--------CGCAAGGG---ATCGTTAGGTTCTA--
TGTACGATGAAGGTCGGAGTGGTATCCGATATGGTGCTACAAGCGCAGTTATTGTTGCACGTTGCCTAATTGA
ACA-
ATGATGTTCGAATACTCCTTTGAATTAAGCA???
???
???
???
???
>AJ438957_Dber
AGC-GTAACGTGGAGCGGTGAAAGGCTCAGTAACGGGCGAGTTATTTGTTC-TCCTGGGA-------
CGGACAACACCGG-GAAACTGGTGGGAAAACGTCTAAGTTGCGG------T-
TTTTTAATCGTGGCGTAAACC------------
ATTTGGTGCAGGAGAGTAAGCTGCCATCCTATCAGTTAGTAAGTAGGGTAAGGGCCTACTTAGACGAAGACGG
GTACGGGGAATGAGGGTTTGATTCCG--
GAGAGGGAGCCTGAGAGACGGCTACCAGGACCAAGGTCAGCAGCAGGCGCGAAAATTATCGAAGCCCGCA-
TAGGGGCGATAGTGAGGAGACGTGTAT--TACGAAGTGTGTGTAAAGAACGCACTAATAACT---
GGAGGTCAAGTCTGGTGCCAGCATCCGCGGTAATTCCAGCTCCAGGGGTGTCTATGATGATTGCTGCGATTAA
AAAGTCCGTAGTCAAGCTGACTG-ACTTGCCTGCAATGTGAC-TG------
ATTAAGAGACGAGCAGGGCTAGGAAAGCAGAGAATTAGGAGCGACCGAGGGCTAGAGTATTGAATGGCGAGAG
GTGAAATTTGATGACCCAT-
TCAGGAGTGACAAAGGCGAAGGCACTAGTCAAGGGCGAATCCGATGATCAAGGACGTAGGCTAGAGTTTCGAA
AACGATT--AGAGACCGGAGTAGTTCTAGCAGTAAACTATGCCGACGCCGTGGTATG--GTATT------
CTGTATTGCGGAAGAGAAATC--AAGT-
AAGGCTTTGGGGAGAGTACGCGCGCAAGCGATAAATTTAAAGGAAATTGACGGAGGAACACCACAAGGAGTGG
AGTGTGCGGCTTAATTTGACTCAACGCGGGACAGCTTACCAGGCCCGATAATCATACGAGCGTAGTACGCGAT
AGGTTAAAGAGTGGTGCATGGCTGCTATCGACAGTTGGGGTGACC-TTAGGGTTAATTCCG-
GCAAGTAGTGAGACCTCTGCAGTTA----------TGGACAGGTATTTTT--AAGATACAGGAAGG-
AAGAGACAAGAGCA-GGTCAGTGATGCCCTTAGATGGCCTGGGCT----GCACGCGCACTACAGTGG-
TCATTATAAGATGAAG-------TT-AGATTTTAAAGA-----------TGATCGAGAGGGA-
CTGGGCTTTG-TAAGAGGCCCAAGAACGAGGAATT-
GCTAGTAATCGTAGGCTCATTAAGATACGATGAATATGTCCCTGTACCTT---
GTACACACCGCCCGTCGTTATCGAAGATGGAATTGTGTGC-GAACGAGC--
AACAAGCGAGTGAGCGCATAGTTCTAGATGTGATAAAAG-
TCGTAACAAGGCAACTGTAGGAGAACCTGTAGTTGGATCATACAGATATAATAA----AAA-
GTAAGTTTGATTTTAATTT-----CCCTG--------CGCAAGGG---ATCGTTTGGTTCTA--
TGTGCGATGAAGGTCGGAGCAGTATCTGATAATGTAGAATGATTGTAATAATTGTTCTATGTGACTGAATTGA
ACA-GTGATGTTCGGGTACTCCTT-
GAATAA???
???
???

???
????????????????????????????????????
>AF397404_Ddue
AGC-TTTTAGTGGAGCGGTATACGGCTCAGTAACGGGCGATTTATTTGTTC-TCCTGGGA-------
CGGACAACATCGG-GAAACTGATGGGAAAACGTCTAAGTTGCAG------T-
TAATTGACTGTGACGTAAACC------------
TTCGTGTGCAGGAGAGTAAGATGCCATCCTATCAGTTAGTAAGTAGGGTAAGGGCCTACTTAGACGAAGACGG
GTACGGGGAATGAGGGTTTGATTCCG--
GAGAGGGAGCCTGAGAGATGGCTACCAGGACCAAGGTCAGCAGCAGGCGCGAAAATTATCGAAGCCCGCC-
TAGGGGCGATAGTGAGGAGACGTGTAT--AACGAAGTACGTGTAAAGAACGTGCTAATAACT---
GGAGGTCAAGTCTGGTGCCAGCATCCGCGGTAATTCCAGCTCCAGGGGTGTCTATGATGATTGCTGCGATTAA
AAAGTCCGTAGTCAATCTGACTG-ACTGACCTGCAATGTGAT-TG------
ATTAAAGAACGAGCAGGGTTAGGAAAGCAGAGAATTAGGAGCGACCGAGGGCTAGAGTATTGAATGGCGAGAG
GTGAAATTTGATGACCCAT-
TCAGGAGTGACAAAGGCGAAGGCACTAGTCAAGGGCGAATCCGATGATCAAGGACGTAGGCTAGAGTTTCGAA
AACGATT--AGAGACCGGAGTAGTTCTAGCAGTAAACTATGCCGACGCCGTGGTATC--AACAA------
TGGTATTGCGGAAGAGAAATC--AAGT-
AAGGCTTTGGGGAGAGTACGCGCGCAAGCGATAAATTTAAAGGAAATTGACGGAGGAACACCACAAGGAGTGG
AGTGTGCGGCTTAATTTGACTCAACGCGGGACAGCTTACCAGGCCCGATAATCGAGCGAGCGTAGTACGCGAT
AGGTTAAAGAGTGGTGCATGGCTGCTATCGACAGTTGGGGTGACC-TTAGGGTTAATTCCG-
GCAAGTAGTGAGACCCCTGCAGATAG---------TGGACAGGTATTTTT--AAAATACAGGAAGG-
AAGGGACAAGAGCA-GGTCAGTGATGCCCTTAGATGGCCTGGGCT----GCACGCGCACTACAGTGG-
TCATTATAAGTAGAAG-------TT-AGA-TATAAAGA-----------TGATCGAGAGGGA-
CTGAGCTTTG-TAAGAGGCTCACGAACGAGGAATT-
GCTAGTAATCGTAGGCTCATTAAGATACGATGAATATGTCCCTGTACCTT---
GTACACACCGCCCGTCGTTATCGAAGATGGAATTGTGTGC-GAACGAGC--
AACAAGCGAGTGAGCGCATAGTTCTAGATGTGATAAAAG-
TCGTAACAAGGCAACTGTAGGAGAACCTGTAGTTGGATCATACAGATTTTCTTA----ATAGAAGTTTG---
AGAAGTATT-----CCTTA--------CGCAAGGG---ATCGTTTGGTTCTA--
TGTACGATGAAGGTCGGAGCTGTATTCGATAAAATGAGATGATTGTATAAATTGTCTTATATTACTGAATAGA
GTA-GCGATGCTCGAATACTCCTT-
GAA??
???
???
???
????????????????????????????????????
>AF044391_Glugea anomala
AGC--GTAAGTGGAGCGGCGCAAGGCTCAGTAACGGGCGAGT-ATTTGATCTTCCTAGAG-------
TGGATATCCTCTGCTAACCGGAGGGCAAAACACAAGATGAGCGATTG------
ACGAGGTCGTTCGTCTAACGA------------
ATAGTGTAGGAGAGTAAGAAGCCATCCCATCAGTTAGTAAGTAGGGTAAGGGCCTACTTAGACGAAGACGGGT
ACGGGGAATTATCGTTTGATTCCG--
GAGAGGGAGCCTGAGAGACGGCTACCAGGTCCAAGGACAGCAGCAGGCGCGAAAATTACCGCAGCCTGCGTTC
AGGGCGGTAGTAAGGAGACGTGAAA---ACAATGTGCGGGCAAAAAACGCACTAGATACA---
GGAGGACAAGACTGGTGCCAGCACCCGCGGTAATACCAGCTCCTGGAGTGTCTATGATGATTGCTGCAGTTAA
AGAGTTCGTAGTCGAAGTGGTTATAACGGTGTAACAGGCCTT-CT------
CTCAAGGAGGGTTATGCGCCGTGATTCCATGGAATAAGGAGCGTTTAGGGGCCAGGTTATTAAGCGACGAGGG
GTGAAATCTGGTGACTCGC-
TTAGGAGCAACAGAGGCGAAAGCGCTGGCCAGGAGCGAATCCGATGATAAAGGACGTAGGCTAGAGGATCGAA
GACGATT--AGAGACCGTTGTAGTTCTAGCAGTAAACGATGCCGATACCGTGGTGCG----------GATAC-
CGACG-GGAAGAGAAATC--GAGT-AGGGCCCTGGGGAGAGTACACGCGCAAG-
AGGAAATTTAAAGGAAATTGACGGAAGAACACCACAAGGAGTGGAGTGTGCGGCTTAATTTGACTCAACGCGG
GACAGCTTACCAGGCCCGACGGCCGGACGAGTGTTGTACACGATAGGTCGAAGAGTGGTGCATGGCCGTTAAC
GACGAGTGAGGTGACT-TTTGGGTTAAATCCG-GGAGGTAGTGAGACCCCTACCGAAA----------
GGGACAGGTGCC----GAAAGCACAGGAAGG-AAGGGTCAAGAACA-
GGTCAGTGATGCCCTCAGATGGTCTGGGCT----GCACGCGCACTACAGTGG-TCATAGAAATGAAACG----
---AT-AGA-ATTAAAGA-----------TGATCGAGAGGGA-ATGAGCTTTG-
TAAGAGGCTCAGGAACGAGGAATT-

GCTAGTAATCGCGGACTCATTAAGACGCGATGAATACGTCCCTGTTCTTTGTAGTACGCACCGCCCGTCGTTA
TCGAAGATGGAGTCAGGCGCGGAACAAGC---
GAGAGCGAGTGAGTGCAGGATTCTAGATGTGATACAAGTTCGTAACAAGGTAGCTGTAGGAGAACCTGTGACT
GGTACAGAACGATGTTTAAT----AAGAAAG-------AGATAATAA-----CCCTG------
TACGCAAGGGTACATCTCTAGGCTCGC--ATGACGAGAAGGG-CGCAGTTGTAGGCGA-
AATGTGTCAGGGCAGCAGCAATGACGACACGTGCCTGAACAAGCGA----
AAGCTCGGATACCCCCTTGAATTAAGCATATGAGTAAAGGGAGGAAAAGAAACTAAC-
AAGGATTCCTGCAGTAGCGGCGAGCGAAGAAGGACCAGCCC-CGTGTGTAATCGTTCTT---------
TCGAGAACGAGATGTCATGGACG----------
CGATGTAACGATCTGGAAAGATCGGCCTGAGAGGGTGATAGCCCCGTACAGCG--------
TGAATGGATGAGTAGTAGTGTTTGGTATTACACTATG--
AACAGGTGGCAGCGCCCATCTAAGGCTAAATACTATGC-
GAGACCGATAGCGAATAAAATAAGTAGAGTGATCGAAGGATGAATAGG
>GQ203287_Glugea hertwigi
AGC--GTAAGTGGAGCGGCGCAAGGCTCAGTAACGGGCGAGT-ATTTGATC-TCCTAGAG-------
TGGATATCCTCTG-TAACCGGAGGGCAAAACACAAGATGAGCGATTG------
ACAAGGTCGTTCGTTTAACGA-------------
ATAGTGTAGGAGAGTAAGAAGCCATCCCATCAGTTAGTAAGTAGGGTAAGGGCCTACTTAGACGAAGACGGGT
ACGGGGAATTATCGTTTGATTCCGGAGAGAGGGAGCCTGAGAGACGGCTACCAGGTCCAAGGACAGCAGCAGG
CGCGAAAATTACCGCAGCCTGCGTTCAGGGTGGTAGTAAGGAGACGTGAAA---
ACAATGTGCGGGCAAAAGACGCACTAGATACA---
GGAGGACAAGACTGGTGCCAGCACCCGCGGTAATACCAGCTCCTGGAGTGTCTATGATGATTGCTGCAGTTAA
AGAGTTCGTAGTCGAAGTGGTTATAACGGTGTAACAGGCCTT-CT------
CTCAAGGAGGGTTATGCGCCGTGATTCCATGGAATAAGGAGCGTTTAGGGGCCAGGTTATTAAGCGACGAGGG
GTGAAATCTGGTGACTCGC-
TTAGGAGCAACAGAGGCGAAAGCGCTGGCCAGGAGCGAATCCGATGATAAAGGACGTAGGCTAGAGGATCGAA
GACGATT--AGAGACCGTTGTAGTTCTAGCAGTAAACGATGCCGATACCGTGGTGCG----------
GATACGCGACGCGGAAGAGAAATC--GAGT-
AGGGCCCTGGGGAGAGTACACGCGCAAGCGAGAAATTTAAAGGAAATTGACGGAAGAACACCACAAGGAGTGG
AGTGTGCGGCTTAATTTGACTCAACGCGGGACAGCTTACCAGGCCCGACGGCCGGACGAGTGTTGTACACGAT
AGGTCGAAGAGTGGTGCATGGCCGTTAACGACGAGTGAGGTGACT-TTTGGGTTAAATCCG-
GGAAGTAGTGAGACCCCTACCGAAA----------GGGACAGGTGCC----GAAAGCACAGGAAGG-
AAGGGTCAAGAACA-GGTCAGTGATGCCCTCAGATGGTCTGGGCT----GCACGCGCACTACAGTGG-
TCATAGAAATGAAACG-------AT-AGA-ATTAAAGA-----------TGATCGAGAGGGA-
ATGAGCTTTG-TAAGAGGCTCAGGAACGAGGAATT-
GCTAGTAATCGCGGACTCATTAAGACGCGATGAATACGTCCCTGTTCTTT---
GTACACACCGCCCGTCGTTATCGAAGATGGAGTCAGGCGC-GAACAAGC---
GAGAGCGAGTGAGTGCAGGATTCTAGATGTGATACAAG-
TCGTAACAAGGTAGCTGTAGGAGAACCTGTAGCTGGATCAGAACGATGTTTTAT----AAGAAAG-------
AGATGATAA-----CCCTG--------CGCAAGGG---ATCTCTAGGCTCGC--
ATGACGAGAAAGGGCGCAGTTGTAGGCGA-
AATGTGTCAGAGCAGCAGCAATGACGACACGTGCCTGAACAAGCGA----
AAGCTCGGATACCCCTTTGAATTAAGCATATGAGTAAAGGGAGGAAAAGAAACTAACAAAGGATTCCTGTAGT
AGCGGCGAGCGAAGAAGGACCAGCCC-CGTGTGTAATCGTTTTT---------
TCGAGAACGAGATGTCATGGACG----------
CGATGTAACGATCTGGAAAGATCGGCCTAAGAGGGTGATAGCCCCGTGCGGCG--------
TAAATTGATGAGTAGTAGTGTTTGGTATTGCACTATG--
AACAGGTGGCAGCGCCCATCTAAGGCTAAATACTATGC-GAGACCGATAGCGAATAA-----
GTAGAGTGATCGAAGGATGAATAGG
>GQ246188_Cucumispora dikerogammari
A----CAAGAGGAAGCTGCGGACTGCTCAGTAACAGACATATAATTTAATC-
TTTACAGAAACGAGCGGAATAAACTCAG-GAAACAGAGTGCAATACGTAAAAGACGAATTTT---------
TTATTATAAGAAATACGTTTTTAGCTTGAACAAAGCGGTAAAGAATAAGTTGTCAGCCTATCAGTTAGTAAGT
AGGGTAAGGGCCTATTTAGACGAAGACGGGTACGGGGAATTAGAGTTTGATTCCG--
GAGAGGGAGCCTGAGAAATAGCTACCAGGTCCAAGGACGGCAGCAGGCGCGAAAATTACCGAAGCTCGAA-
TAGAGGCGGTAGTAATGAGACGTATTAA-TATAAAACAAGGGTAAAAAACTTGTTAGTAACT---
GGAGGTCAAGTCTGGTGCCAGCATCCGCGGTAATACCAGCTCCAGGGGTGTCTATGATGATTGCTGCGATTAA
AAGGTCCGTAGTCGAATTTATAT-AATTGTTTGTAATATGCT-AG------

```
ATAAAATAACAGAAAGAACAATTACTTTAAATGAA-
AGGAATAGTAAGGGGCTGATTAATTGAGCAACGAGAGGTGAAATTTGATGACTTGC-
TTAGGAGAAACAGAGGCGAAAGCGTCAGTCAAGTATAAATCCTATGATCAAGGACGTAGGCTAGAGTATCGAA
CACGATT--AGATACCGTAGTAGTTCTAGCAGTAAACTATGCCTACACTATCGAATA------------
AAAGTTTGGTAGAAGAGAAATCTTAAGT-
AGGGCTTTGGGGAGAGTACACGCGCAAGCGATAAATTTAAAGGAAATTGACGGAAGAACACCACAAGGAGTGG
AGTGTGCGGCTTAATTTGACTCAACGCGGGACAGCTTACCATACCCGAGGACTATAAGAGTG-
AATACACGATAAGTCTAAAAGTGGTGCATGGCCGTTATCGACGAGTGAAGTGATT-TTATGGTTAAATCCG-
ACAAGTTGTGAGACCCTTA------------TTTAAATACAGGTATTGTT--AAAATACAGGAAGG-
AAAGGACAAGAACA-GGTCAGTGATGCCCTTAGATGGTATGGGCT----GCACGCGCACTACAATGG-
TTATAATAATAAA-----GATAATT-AAAGTATAAATA-----------TAATCAAGAGGAA-
TTGAGAACTG-AAAAGTTCCCATGAACGAGGAATT-
GCTAGTAATCGTAGGCTCAGTAAGATACGATGAATATGTCCCTGTTCTTT---
GTACACACCGCCCGTCGTTATCGAAGATGGAGTTTTACCC-GAACAAGC---
TTAAGCGAGTGAGTGTATGATTCTAGATCTGATACAAG-
TCGTAACAAGGCAGCCGTAGGAGAACCTGCTGCTGGATCAACTAAG--------
AAAAAACCAACAAAATACAAAAATATA--------TAAGAAATAATACAAGGG---
ATCATTTGGTTCTAGATTGACGAAGAAGGGCAATAAAGATATTGA-
AAACTATTAGAACATTAGTAACGACAATAGGTTCCCTAAT-----A-
ACTTTGTAAGGCAACTCCTCGGATTTAAGCATATGAGTAGAGGAAGGAAAAGAAACTAAC-
AAGGATTTCTCTAGTAGCGGCGAGTGAACAAGAAACAGCTC-AAAGTATAATTCTAATATAAA--
TATATATATAGAATATGTCAAAAAAATATACAATTTAATTTTAAACATTTTAAATAATGTACCAAAGAAAGTT
ATAGTCTTGTTAAATTAAATAAATATAAAAGAAGAGTAATTGTGTTTGGTAGTACATAATGAATATTGGTGGT
GTGAATCATCAAAAGCTAAATACAAC-TAGAAACCGATAGCAAATAA-----
GTAGATGACTGATATGTGAAATGTA
```

Alignment 3: molecular characteristic features

```
                ....|....| ....|....| ....|....| ....|....| ....|....|
....|....|
                     5          15         25         35         45
55
D1              AGCTTTTAGT GGAGCGGTAT ACGGCTCAGT AACGGGCGAT TTATTTGTTC
TCCTGGGACG
D2              AGCTTTTAGT GGAGCGGTAT ACGGCTCAGT AACGGGCGAT TTATTTGTTC
TCCTGGGACG
D3              AGCTTATAGT GGAGCGGTAT ACGGCTCAGT AACGGGCGAT TTATTTGTTC
TCCTGGGACG
R1              AGCGATATGT GGAGCGGTGA AAGGCTCAGT AACGGGCGAT TTATTTGATC
TCCTGGGACG
R2              AGCGATATGT GGAGCGGTGA AAGGCTCAGT AACGGGCGAT TTATTTGATC
TCCTGGGACG
R3              AGCGATATGT GGAGCGGTGA AAGGCTCAGT AACGGGCGAT TTATTTGATC
TCCTGGGACG
R4              AGCGATATGT GGAGCGGTGA AAGGCTCAGT AACGGGCGAT TTATTTGATC
TCCTGGGACG
R5              AGCGATAAGT GGAGCGGTGA AAGGCTCAGT AACGGGCGAT TTATTTGATC
TCCTGGGACG
R6              AGCGATAAGT GGAGCGGTGA AAGGCTCAGT AACGGGCGAT TTATTTGATC
TCCTGGGACG
R7              AGCGATAAGT GGAGCGGTGA AAGGCTCAGT AACGGGCGAT TTATTTGATC
TCCTGGGACG
N1              CGTTTAACGT GGAGCGGTGA AAGGCTCAGT AACGGGCGAT TTATTTGATC
TCCTGGGACG
```

N2 CGTTTAACGT GGAGCGGTGA AAGGCTCAGT AACGGGCGAT TTATTTGATC
TCCTGGGACG
N3 CGTTTAACGT GGAGCGGTGA AAGGCTCAGT AACGGGCGAT TTATTTGATC
TCCTGGGACG
M1 AGCTATATGT GGAGCGGTGA AAGGCTCAGT AACGGGCGAT TTATTTAATC
TCCTGGGACG
M2 AGCTATATGT GGAGCGGTGA AAGGCTCAGT AACGGGCGAT TTATTTAATC
TCCTGGGACG
M3 AGCTATATGT GGAGCGGTGA AAGGCTCAGT AACGGGCGAT TTATTTAATC
TCCTGGGACG
M4 AGCTATATGT GGAGCGGTGA AAGGCTCAGT AACGGGCGAT TTATTTAATC
TCCTGGGACG
M5 AGCTATATGT GGAGCGGTGA AAGGCTCAGT AACGGGCGAT TTATTTAATC
TCCTGGGACG
M6 AGCTATATGT GGAGCGGTGA AAGGCTCAGT AACGGGCGAT TTATTTAATC
TCCTGGGACG
M7 AGCTATATGT GGAGCGGTGA AAGGCTCAGT AACGGGCGAT TTATTTAATC
TCCTGGGACG
M8 AGCTATATGT GGAGCGGTGA AAGGCTCAGT AACGGGCGAT TTATTTAATC
TCCTGGGACG
M9 AGCTATATGT GGAGCGGTGA AAGGCTCAGT AACGGGCGAT TTATTTAATC
TCCTGGGACG
M10 AGCTATATGT GGAGCGGTGA AAGGCTCAGT AACGGGCGAT TTATTTAATC
TCCTGGGACG
M11 AGCTATATGT GGAGCGGTGA AAGGCTCAGT AACGGGCGAT TTATTTAATC
TCCTGGGACG
M12 AGCTATATGT GGAGCGGTGA AAGGCTCAGT AACGGGCGAT TTATTTAATC
TCCTGGGACG
M13 AGCTATATGT GGAGCGGTGA AAGGCTCAGT AACGGGCGAT TTATTTAATC
TCCTGGGACG
M14 AGCTATATGT GGAGCGGTGA AAGGCTCAGT AACGGGCGAT TTATTTAATC
TCCTGGGGCG
M15 AGCTATATGT GGAGCGGTGA AAGGCTCAGT AACGGGCGAT TTATTTAATC
TCCTGGGGCG
M16 AGCTATATGT GGAGCGGTGA AAGGCTCAGT AACGGGCGAT TTATTTAATC
TCCTGGGGCG
M17 AGCTATATGT GGAGCGGTGA AAGGCTCAGT AACGGGCGAT TTATTTAATC
TCCTGGGGCG
M18 AGCTATATGT GGAGCGGTGA AAGGCTCAGT AACGGGCGAT TTATTTAATC
TCCTGGGACG
M19 AGCTATATGT GGAGCGGTGA AAGGCTCAGT AACGGGCGAT TTATTTAATC
TCCTGGGACG
M20 AGCTATATGT GGAGCGGTGA AAGGCTCAGT AACGGGCGAT TTATTTAATC
TCCTGGGACG
M21 AGCTATATGT GGAGCGGTGA AAGGCTCAGT AACGGGCGAT TTATTTAATC
TCCTGGGACG
M22 AGCTATATGT GGAGCGGTGA AAGGCTCAGT AACGGGCGAT TTATTTAATC
TCCTGGGACG
M23 AGCTATATGT GGAGCGGTGA AAGGCTCAGT AACGGGCGAT TTATTTAATC
TCCTGGGACG
B1 AGCGTAACGT GGAGCGGTGA AAGGCTCAGT AACGGGCGAG TTATTTGTTC
TCCTGGGACG
B2 AGCGTGGAGC GGAGCAGTGA AAGGCTCAGT AACGGGCGAG TTATTTGTTC
TCCTGGGACG
B3 AGCGTAACGT GGAGCGGTGA AAGGCTCAGT AACGGGCGAG TTATTTGTTC
TCCTGGGACG
B4 AGCGTAACGT GGAGCGGTGA AAGGCTCAGT AACGGGCGAG TTATTTGTTC
TCCTGGGACG

```
B5             AGCGTAACGT GGAGCGGTGA AAGGCTCAGT AACGGGCGAG TTATTTGTTC
TCCTGGGACG
B6             AGCGTAACGT GGAGCGGTGA AAGGCTCAGT AACGGGCGAG TTATTTGTTC
TCCTGGGACG
B7             AGCGTAACGT GGAGCGGTGA AAGGCTCAGT AACGGGCGAG TTATTTGTTC
TCCTGGGACG
B8             AGCGTAACGT GGAGCGGTGA AAGGCTCAGT AACGGGCGAG TTATTTGTTC
TCCTGGGACG
B9             AGCGTAACGT GGAGCGGTGA AAGGCTCAGT AACGGGCGAG TTATTTGTTC
TCCTGGGACG
B10            AGCGTAACGT GGAGCGGTGA AAGGCTCAGT AACGGGCGAG TTATTTGTTC
TCCTGGGACG
B11            AGCGTAACGT GGAGCGGTGA AAGGCTCAGT AACGGGCGAG TTATTTGTTC
TCCTGGGACG
B12            AGCGTAACGT GGAGCGGTGA AAGGCTCAGT AACGGGCGAG TTATTTGTTC
TCCTGGGACG
B13            AGCGTAACGT GGAGCGGTGA AAGGCTCAGT AACGGGCGAG TTATTTGTTC
TCCTGGGACG
B14            AGCGTAACGT GGAGCGGTGA AAGGCTCAGT AACGGGCGAG TTATTTGTTC
TCCTGGGACG
B15            AGCGTAACGT GGAGCGGTGA AAGGCTCAGT AACGGGCGAG TTATTTGTTC
TCCTGGGACG
B16            AGCGTAACGT GGAGCGGTGA AAGGCTCAGT AACGGGCGAG TTATTTGTTC
TCCTGGGACG
KF537632_Ddi   AGCGTAACGT GGAGCGGTGA AAGGCTCAGT AACGGGCGAT TTATTTGATC
TCCTGGGATG
AJ438961_Dde   AGCGTAACGT GGAGCGGTGA AAGGCTCAGT AACGGGCGAT TTATTTGATC
TCCTGGGACG
AJ438960_Dca   AGC-TATTGT GGAGCGGTGA AAGGCTCAGT AACGGGCGAT TTATTTAGTC
TTCTGGGACG
AJ438959_Dca   AGC-TATTGT GGAGCGGTGA AAGGCTCAGT AACGGGCGAT TTATTTAGTC
TTCTGGGACG
AJ438958_Dga   AGCATATTGT GGAGCGGCGA AAGGCTCAGT AACGGGCGAT TTATTTATTC
TTCTGTGACG

               ....|....| ....|....| ....|....| ....|....| ....|....|
....|....|
                   65         75         85         95         105
115
D1             GACAACATCG GGAAACTGAT GGGAAAACGT CTAAGTTGCA G---T-TAAT
TGACTGTGAC
D2             GACAACATCG GGAAACTGAT GGGAAAACGT CTAAGTTGCA G---T-TAAT
TGATTGTGAC
D3             GACAACATCG GGAAACTGAT GGGAAAACGT CTAAGTTGCA G---T-TAAT
TGACTGTGAC
R1             GACAACATCG GGAAACTGAT GGGAAAACGT CTAAGTTGCA G---T-TTTC
TGATTGCGAC
R2             GACAACATCG GGAAACTGAT GGGAAAACGT CTAAGTTGCA G---T-TTTT
TGACTGTGAC
R3             GACAACATCG GGAAACTGAT GGGAAAACGT CTAAGTTGCA G---T-TTTT
TGACTGTGAC
R4             GACAACATCG GGAAACTGAT GGGAAAACGT CTAAGTTGCA G---T-TAAA
TGATTGTGAC
R5             GACAACATCG GGAAACTGAT GGGAAAACGT CTAAGTTGCA GTTTT-TTTT
TGATTGCGAC
R6             GACAACATCG GGAAACTGAT GGGAAAACGT CTAAGTTGCA G---T-TGTT
TTATTGTGAC
```

R7 GACAACATCG GGAAACTGAT GGGAAAACGT CTAAGTTGCA G---T-AAAT
TTATTGTGAC
N1 GACAACATCG GGAAACTGAT GGGAAAACGT CTAAGTTGCA G---T-TGTA
TGATTGTGAC
N2 GACAACATCG GGAAACTGAT GGGAAAACGT CTAAGTTGCA G---T-TGTA
TGATTGTGAC
N3 GACAACATCG GGAAACTGAT GGGAAAACGT CTAAGTTGCA G---T-TGTA
TGATTGTGAC
M1 GACAACATCG GGAAACTGAT GGGAAAACGT CTAAGTTGCA TTAATATTAG
TTAGTGTGAC
M2 GACAACATCG GGAAACTGAT GGGAAAACGT TTTAGTTGCA TTAATATTAG
TTAGTGTGAC
M3 GACAACATCG GGAAACTGAT GGGAAAACGT CTAAGTTGCA TTAATATTAG
TTAGTGTGAC
M4 GACAACATCG GGAAACTGAT GGGAAAACGT CTAAGTTGCA TTAATATTAG
TTAGTGTGAC
M5 GACAACATCG GGAAACTGAT GGGAAAACGT CTAAGTTGCA TTAATATTAG
TTAGTGTGAC
M6 GACAACATCG GGAAACTGAT GGGAAAACGT CTAAGTTGCA TTAATATTAG
TTAGTGTGAC
M7 GACAACATCG GGAAACTGAT GGGAAAACGT CTAAGTTGCA TTAATATTAG
TTAGTGTGAC
M8 GACAACATCG GGAAACTGAT GGGAAAACGT CTAAGTTGCA TTAATATTAG
TTAGTGTGAC
M9 GACAACATCG GGAAACTGAT GGGAAAACGT CTAAGTTGCA TTAATATTAG
TTAGTGTGAC
M10 GACAACATCG GGAAACTGAT GGGAAAACGT CTAAGTTGCA TTAATATTAG
TTAGTGTGAC
M11 GACAACATCG GGAAACTGAT GGGAAAACGT CTAAGTTGCA TTAAT----G
TTAGTGTGAC
M12 GACAACATCG GGAAACTGAT GGGAAAACGT CTAAGTTGCA TTAAT----G
TTAGTGTGAC
M13 GACAACATCG GGAAACTGAT GGGAAAACGT CTAAGTTGCA TTAATATTAG
TTAGTGTGAC
M14 GACAACATCG GGAAACTGAT GGGAAAACGT CTAAGTTGCA TTAAT----T
TTAGTGTGAC
M15 GACAACATCG GGAAACTGAT GGGAAAACGT CTAAGTTGCA TTAAT----T
TTAGTGTGAC
M16 GACAACATCG GGAAACTGAT GGGAAAACGT CTAAGTTGCA TTAAT----T
TTAGTGTGAC
M17 GACAACATCG GGAAACTGAT GGGAAAACGT CTAAGTTGCA TTAAT----T
TTAGTGAGAC
M18 GACAACATCG GGAAACTGAT GGGAAAACGT CTAAGTTGCA TTAAT----G
TTAGTGTGAC
M19 GACAACATCG GGAAACTGAT GGGAAAACGT CTAAGTTGCA TTAAT----G
TTAGTGTGAC
M20 GACAACATCG GGAAACTGAT GGGAAAACGT CTAAGTTGCA G---T-TTTG
TTATTGCGAC
M21 GACAACATCG GGAAACTGAT GGGAAAACGT CTAAGTTGCA G---T-TTTG
TTATTGCGAC
M22 GACAACATCG GGAAACTGAT GGGAAAACGT CTAAGTTGCA G---T-TTTG
TTATTGCGAC
M23 GACAACATCG GGAAACTGAT GGGAAAACGT CTAAGTTGCA G---T-TTTG
TTATTGTGAC
B1 GACAACACCG GGAAACTGGT GGGAAAACGT CTAAGTTGCG G---T-TTTT
TAATCGTGGC
B2 GACAACACCG GGAAACTGGT GGGAAAACGT CTAAGTTGCG G---T-TTTT
TAATCGTGGC

B3 GACAACACCG GGAAACTGGT GGGAAAACGT CTAAGTTGCG G---T-TTTT
TAATCGTGGC
B4 GACAACACCG GGAAACTGGT GGGAAAACGT CTAAGTTGCG G---T-TTTT
TAATCGTGGC
B5 GACAACACCG GGAAACTGGT GGGAAAACGT CTAAGTTGCG G---T-TTTT
TAATCGTGGC
B6 GACAACACCG GGAAACTGGT GGGAAAACGT CTAAGTTGCG G---T-TTTT
TAATCGTGGC
B7 GACAACACCG GGAAACTGGT GGGAAAACGT CTAAGTTGCG G---T-TTTT
TAATCGTGGC
B8 GACAACACCG GGAAACTGGT GGGAAAACGT CTAAGTTGCG G---T-TTTT
TAATCGTGGC
B9 GACAACACCG GGAAACTGGT GGGAAAACGT CTAAGTTGCG G---T-TTTT
TAATCGTGGC
B10 GACAACACCG GGAAACTGGT GGGAAAACGT CTAAGTTGCG G---T-TTTT
TAATCGTGGC
B11 GACAACACCG GGAAACTGGT GGGAAAACGT CTAAGTTGCG G---T-TTTT
TAATCGTGGC
B12 GACAACACCG GGAAACTGGT GGGAAAACGT CTAAGTTGCG G---T-TTTT
TAATCGTGGC
B13 GACAACACCG GGAAACTGGT GGGAAAACGT CTAAGTTGCG G---T-TTTT
TAATCGTGGC
B14 GACAACACCG GGAAACTGGT GGGAAAACGT CTAAGTTGCG G---T-TTTT
TAATCGTGGC
B15 GACAACACCG GGAAACTGGT GGGAAAACGT CTAAGTTGCG G---T-TTTT
TAATCGTGGC
B16 GACAACACCG GGAAACTGGT GGGAAAACGT CTAAGTTGCG G---T-TTTT
TAATCGTGGC
KF537632_Ddi GACAACACCG GGAAACTGGT GGGAAAACAT CTAAGTTGCG G---TTCTAT
TGATCGTGAT
AJ438961_Dde GACAACACCG GGAAACTGGT GGGAATACGT CTAAGCTGCG GGTCGGCATG
TGATTGCGGC
AJ438960_Dca GACAACACCG GGAAACTGGT GGGAAAACGT CTAAGCTGCG AATCGCTATG
TGGTTGTGGC
AJ438959_Dca GACAACACCG GGAAACTGGT GGGAAAACGT TTAAGCTGCG AATCGCGATG
TGGTTGTGGC
AJ438958_Dga GACAACACCG GGAAACTGGT GGGAAAACGT CTATACTGCA GGAGTAGAAT
TCTGTGGTAG

....|....| ....|....| ....|....| ....|....| ....|....|
....|....|
125 135 145 155 165
175
D1 GTAAACC--T TCGTGTGCAG GAGAGTAAGA TGCCATCCTA TCAGTTAGTA
AGTAGGGTAA
D2 GTAAACC--T TCGTGTGCAG GAGAGTAAGA TGCCATCCTA TCAGTTAGTA
AGTAGGGTAA
D3 GTAAACC--T TTGTGTGCAG GAGAGTAAGA TGCCATCCTA TCAGTTAGTA
AGTAGGGTAA
R1 GTAAACC--A TAGTGTGCAG GAGAGTAAGA TGCCATCCTA TCAGTTAGTA
AGTAGGGTAA
R2 GTAAACC--G TAGTGTGCAG GAGAGTAAGA TGCCATCCTA TCAGTTAGTA
AGTAGGGTAA
R3 GTAAACC--G TAGTGTGCAG GAGAGTAAGA TGCCATCCTA TCAGTTAGTA
AGTAGGGTAA
R4 GTAAACC--A TAGTGTGCAG GAGAGTAAGA TGCCATCCTA TCAGTTAGTA
AGTAGGGTAA

R5 GTAAACC--T TTTGGTGCAG GAGAGTAAGA TGCCATCCTA TCAGTTAGTA
AGTAGGGTAA
R6 GTAAACC--T TTGTGTGCAG GAGAGTAAGA TGCCATCCTA TCAGTTAGTA
AGTAGGGTAA
R7 GTAAACC--T TTGTGTGCAG GAGAGTAAGA TGCCATCCTA TCAGTTAGTA
AGTAGGGTAA
N1 GTAAACC--- TTTGGTGCAG GAGAGTAAGA TGCCATCCTA TCAGTTAGTA
AGTAGGGTAA
N2 GTAAACC--- TTTGGTGCAG GAGAGTAAGA TGCCATCCTA TCAGTTAGTA
AGTAGGGTAA
N3 GTAAACC--- TTTGGTGCAG GAGAGTAAGA TGCCATCCTA TCAGTTAGTA
AGTAGGGTAA
M1 GTAAACG-GT AATCGTGCAG GAGAGTAAGA TGCCATCCTA TCAGTTAGTA
AGTAGGGTAA
M2 GTAAACG-GT AATCGTGCAG GAGAGTAAGA TGCCATCCTA TCAGTTAGTA
AGTAGGGTAA
M3 GTAAACG-GT AATCGTGCAG GAGAGTAAGA TGCCATCCTA TCAGTTAGTA
AGTAGGGTAA
M4 GTAAACG-GT AATCGTGCAG GAGAGTAAGA TGCCATCCTA TCAGTTAGTA
AGTAGGGTAA
M5 GTAAACG-GT AATCGTGCAG GAGAGTAAGA TGCCATCCTA TCAGTTAGTA
AGTAGGGTAA
M6 GTAAACG-GT AATCGTGCAG GAGAGTAAGA TGCCATCCTA TCAGTTAGTA
AGTAGGGTAA
M7 GTAAACG-GT AATCGTGCAG GAGAGTAAGA TGCCATCCTA TCAGTTAGTA
AGTAGGGTAA
M8 GTAAACG-GT AATCGTGCAG GAGAGTAAGA TGCCATCCTA TCAGTTAGTA
AGTAGGGTAA
M9 GTAAACG-GT AATCGTGCAG GAGAGTAAGA TGCCATCCTA TCAGTTAGTA
AGTAGGGTAA
M10 GTAAACG-GT AATCGTGCAG GAGAGTAAGA TGCCATCCTA TCAGTTAGTA
AGTAGGGTAA
M11 GTAAACG-GT AATCGTGCAG GAGAGTAAGA TGCCATCCTA TCAGTTAGTA
AGTAGGGTAA
M12 GTAAACG-GT AATCGTGCAG GAGAGTAAGA TGCCATCCTA TCAGTTAGTA
AGTAGGGTAA
M13 GTAAACG-GT AATCGTGCAG GAGAGTAAGA TGCCATCCTA TCAGTTAGTA
AGTAGGGTAA
M14 GTAAACG-AT TATCGTGCAG GAGAGTAAGA TGCCATCCTA TCAGTTAGTA
AGTAGGGTAA
M15 GTAAACG-AT TATCGTGCAG GAGAGTAAGA TGCCATCCTA TCAGTTAGTA
AGTAGGGTAA
M16 GTAAACG-AT TATCGTGCAG GAGAGTAAGA TGCCATCCTA TCAGTTAGTA
AGTAGGGTAA
M17 GTAAACG-AT TATCGTGCAG GAGAGTAAGA TGCCATCCTA TCAGTTAGTA
AGTAGGGTAA
M18 GTAAACG--A TATCGTGCAG GAGAGTAAGA TGCCATCCTA TCAGTTAGTA
AGTAGGGTAA
M19 GTAAACG--A TATCGTGCAG GAGAGTAAGA TGCCATCCTA TCAGTTAGTA
AGTAGGGTAA
M20 GTAAACG-GT AATCGTGCAG GAGAGTAAGA TGCCATCCTA TCAGTTAGTA
AGTAGGGTAA
M21 GTAAACG-GT AATCGTGCAG GAGAGTAAGA TGCCATCCTA TCAGTTAGTA
AGTAGGGTAA
M22 GTAAACG-GT AATCGTGCAG GAGAGTAAGA TGCCATCCTA TCAGTTAGTA
AGTAGGGTAA
M23 GTAAACG-GT AATCGTGCAG GAGAGTAAGA TGCCATCCTA TCAGTTAGTA
AGTAGGGTAA

```
B1            GTAAACC--A TTTGGTGCAG GAGAGTAAGC TGCCATCCTA TCAGTTAGTA
AGTAGGGTAA
B2            GTAAACC--A TTTGGTGCAG GAGAGTAAGC TGCCATCCTA TCAGTTAGTA
AGTAGGGTAA
B3            GTAAACC--A TTTGGTGCAG GAGAGTAAGC TGCCATCCTA TCAGTTAGTA
AGTAGGGTAA
B4            GTAAACC--A TTTGGTGCAG GAGAGTAAGC TGCCATCCTA TCAGTTAGTA
AGTAGGGTAA
B5            GTAAACC--A TTTGGTGCAG GAGAGTAAGC TGCCATCCTA TCAGTTAGTA
AGTAGGGTAA
B6            GTAAACC--A TTTGGTGCAG GAGAGTAAGC TGCCATCCTA TCAGTTAGTA
AGTAGGGTAA
B7            GTAAACC--A TTTGGTGCAG GAGAGTAAGC TGCCATCCTA TCAGTTAGTA
AGTAGGGTAA
B8            GTAAACC--A TTTGGTGCAG GAGAGTAAGC TGCCATCCTA TCAGTTAGTA
AGTAGGGTAA
B9            GTAAACC--A TGTGGTGCAG GAGAGTAAGC TGCCATCCTA TCAGTTAGTA
AGTAGGGTAA
B10           GTAAACC--A TGTGGTGCAG GAGAGTAAGC TGCCATCCTA TCAGTTAGTA
AGTAGGGTAA
B11           GTAAACC--A TGTGGTGCAG GAGAGTAAGC TGCCATCCTA TCAGTTAGTA
AGTAGGGTAA
B12           GTAAACC--A TGTGGTGCAG GAGAGTAAGC TGCCATCCTA TCAGTTAGTA
AGTAGGGTAA
B13           GTAAACC--A TGTGGTGCAG GAGAGTAAGC TGCCATCCTA TCAGTTAGTA
AGTAGGGTAA
B14           GTAAACC--A TGTGGTGCAG GAGAGTAAGC TGCCATCCTA TCAGTTAGTA
AGTAGGGTAA
B15           GTAAACC--A TGTGGTGCAG GAGAGTAAGC TGCCATCCTA TCAGTTAGTA
AGTAGGGTAA
B16           GTAAACC--A TGTGGTGCAG GAGAGTAAGC TGCCATCCTA TCAGTTAGTA
AGTAGGGTAA
KF537632_Ddi  GTAAACCTAT GTGGGTGCAG GAGAGTAAGA TGCCATCCTA TCAGTTAGTA
AGTAGGGTAA
AJ438961_Dde  GGAAACA--- -GCAGTGCAG GAGAGTAAGG TGCCATCCTA TCAGTTAGTA
AGTAGGGTAA
AJ438960_Dca  AGAAACT--- GTTAGTGCAA AAAAGTAAGA TGCCATCCTA TCAGTTAGTA
AGTAGGGTAA
AJ438959_Dca  AGAAACT--- GTTAGTGCAG AAGAGTAAGA TGCCATCCTA TCAGTTAGTA
AGTAGGGTAA
AJ438958_Dga  AAAGATT--- -TATCTGTGG AAGAGTAAGA TGCCATCCTA TCAGTTAGTA
AGTAGGGTAA

              ....|....| ....|....| ....|....| ....|....| ....|....|
....|....|
                  185        195        205        215        225
235
D1            GGGCCTACTT AGACGAAGAC GGGTACGGGG AATGAGGGTT TGATTCCGGA
GAGGGAGCCT
D2            GGGCCTACTT AGACGAAGAC GGGTACGGGG AATGAGGGTT TGATTCCGGA
GAGGGAGCCT
D3            GGGCCTACTT AGACGAAGAC GGGTACGGGG AATGAGGGTT TGATTCCGGA
GAGGGAGCCT
R1            GGGCCTACTT AGACGAAGAC GGGTACGGGG AATGAGGGTT TGATTCCGGA
GAGGGAGCCT
R2            GGGCCTACTT AGACGAAGAC GGGTACGGGG AATGAGGGTT TGATTCCGGA
GAGGGAGCCT
```

R3 GGGCCTACTT AGACGAAGAC GGGTACGGGG AATGAGGGTT TGATTCCGGA GAGGGAGCCT
R4 GGGCCTACTT AGACGAAGAC GGGTACGGGG AATGAGGGTT TGATTCCGGA GAGGGAGCCT
R5 GGGCCTACTT AGACGAAGAC GGGTACGGGG AATGAGGGTT TGATTCCGGA GAGGGAGCCT
R6 GGGCCTACTT AGACGAAGAC GGGTACGGGG AATGAGGGTT TGATTCCGGA GAGGGAGCCT
R7 GGGCCTACTT AGACGAAGAC GGGTACGGGG AATGAGGGTT TGATTCCGGA GAGGGAGCCT
N1 GGGCCTACTT AGACGAAGAC GGGTACGGGG AATGAGGGTT TGATTCCGGA GAGGGAGCCT
N2 GGGCCTACTT AGACGAAGAC GGGTACGGGG AATGAGGGTT TGATTCCGGA GAGGGAGCCT
N3 GGGCCTACTT AGACGAAGAC GGGTACGGGG AATGAGGGTT TGATTCCGGA GAGGGAGCCT
M1 GGGCCTACTT AGACGAAGAC GGGTACGGGG AATGAGGGTT TGATTCCGGA GAGGGAGCCT
M2 GGGCCTACTT AGACGAAGAC GGGTACGGGG AATGAGGGTT TGATTCCGGA GAGGGAGCCT
M3 GGGCCTACTT AGACGAAGAC GGGTACGGGG AATGAGGGTT TGATTCCGGA GAGGGAGCCT
M4 GGGCCTACTT AGACGAAGAC GGGTACGGGG AATGAGGGTT TGATTCCGGA GAGGGAGCCT
M5 GGGCCTACTT AGACGAAGAC GGGTACGGGG AATGAGGGTT TGATTCCGGA GAGGGAGCCT
M6 GGGCCTACTT AGACGAAGAC GGGTACGGGG AATGAGGGTT TGATTCCGGA GAGGGAGCCT
M7 GGGCCTACTT AGACGAAGAC GGGTACGGGG AATGAGGGTT TGATTCCGGA GAGGGAGCCT
M8 GGGCCTACTT AGACGAAGAC GGGTACGGGG AATGAGGGTT TGATTCCGGA GAGGGAGCCT
M9 GGGCCTACTT AGACGAAGAC GGGTACGGGG AATGAGGGTT TGATTCCGGA GAGGGAGCCT
M10 GGGCCTACTT AGACGAAGAC GGGTACGGGG AATGAGGGTT TGATTCCGGA GAGGGAGCCT
M11 GGGCCTACTT AGACGAAGAC GGGTACGGGG AATGAGGGTT TGATTCCGGA GAGGGAGCCT
M12 GGGCCTACTT AGACGAAGAC GGGTACGGGG AATGAGGGTT TGATTCCGGA GAGGGAGCCT
M13 GGGCCTACTT AGACGAAGAC GGGTACGGGG AATGAGGGTT TGATTCCGGA GAGGGAGCCT
M14 GGGCCTACTT AGACGAAGAC GGGTACGGGG AATGAGGGTT TGATTCCGGA GAGGGAGCCT
M15 GGGCCTACTT AGACGAAGAC GGGTACGGGG AATGAGGGTT TGATTCCGGA GAGGGAGCCT
M16 GGGCCTACTT AGACGAAGAC GGGTACGGGG AATGAGGGTT TGATTCCGGA GAGGGAGCCT
M17 GGGCCTACTT AGACGAAGAC GGGTACGGGG AATGAGGGTT TGATTCCGGA GAGGGAGCCT
M18 GGGCCTACTT AGACGAAGAC GGGTACGGGG AATGAGGGTT TGATTCCGGA GAGGGAGCCT
M19 GGGCCTACTT AGACGAAGAC GGGTACGGGG AATGAGGGTT TGATTCCGGA GAGGGAGCCT
M20 GGGCCTACTT AGACGAAGAC GGGTACGGGG AATGAGGGTT TGATTCCGGA GAGGGAGCCT
M21 GGGCCTACTT AGACGAAGAC GGGTACGGGG AATGAGGGTT TGATTCCGGA GAGGGAGCCT

M22 GGGCCTACTT AGACGAAGAC GGGTACGGGG AATGAGGGTT TGATTCCGGA
GAGGGAGCCT
M23 GGGCCTACTT AGACGAAGAC GGGTACGGGG AATGAGGGTT TGATTCCGGA
GAGGGAGCCT
B1 GGGCCTACTT AGACGAAGAC GGGTACGGGG AATGAGGGTT TGATTCCGGA
GAGGGAGCCT
B2 GGGCCTACTT AGACGAAGAC GGGTACGGGG AATGAGGGTT TGATTCCGGA
GAGGGAGCCT
B3 GGGCCTACTT AGACGAAGAC GGGTACGGGG AATGAGGGTT TGATTCCGGA
GAGGGAGCCT
B4 GGGCCTACTT AGACGAAGAC GGGTACGGGG AATGAGGGTT TGATTCCGGA
GAGGGAGCCT
B5 GGGCCTACTT AGACGAAGAC GGGTACGGGG AATGAGGGTT TGATTCCGGA
GAGGGAGCCT
B6 GGGCCTACTT AGACGAAGAC GGGTACGGGG AATGAGGGTT TGATTCCGGA
GAGGGAGCCT
B7 GGGCCTACTT AGACGAAGAC GGGTACGGGG AATGAGGGTT TGATTCCGGA
GAGGGAGCCT
B8 GGGCCTACTT AGACGAAGAC GGGTACGGGG AATGAGGGTT TGATTCCGGA
GAGGGAGCCT
B9 GGGCCTACTT AGACGAAGAC GGGTACGGGG AATGAGGGTT TGATTCCGGA
GAGGGAGCCT
B10 GGGCCTACTT AGACGAAGAC GGGTACGGGG AATGAGGGTT TGATTCCGGA
GAGGGAGCCT
B11 GGGCCTACTT AGACGAAGAC GGGTACGGGG AATGAGGGTT TGATTCCGGA
GAGGGAGCCT
B12 GGGCCTACTT AGACGAAGAC GGGTACGGGG AATGAGGGTT TGATTCCGGA
GAGGGAGCCT
B13 GGGCCTACTT AGACGAAGAC GGGTACGGGG AATGAGGGTT TGATTCCGGA
GAGGGAGCCT
B14 GGGCCTACTT AGACGAAGAC GGGTACGGGG AATGAGGGTT TGATTCCGGA
GAGGGAGCCT
B15 GGGCCTACTT AGACGAAGAC GGGTACGGGG AATGAGGGTT TGATTCCGGA
GAGGGAGCCT
B16 GGGCCTACTT AGACGAAGAC GGGTACGGGG AATGAGGGTT TGATTCCGGA
GAGGGAGCCT
KF537632_Ddi GGGCCTACTT AGACGAAGAC GGGTACGGGG AATGAGGGTT TGATTCCGGA
GAGGGAGCCT
AJ438961_Dde GGGCCTACTT AGACGAAGAC GGGTACGGGG AATGAGGGTT TGATTCCGGA
GAGGGAGCCT
AJ438960_Dca GGGCCTACTT AGACGAAGAC GGGTACGGGG AATGAGGGTT TGATTCCGGA
GAGGGAGCCT
AJ438959_Dca GGGCCTACTT AGACGAAGAC GGGTACGGGG AATGAGGGTT TGATTCCGGA
GAGGGAGCCT
AJ438958_Dga GGGCCTACTT AGACGAAGAC GGGTACGGGG AATGAGGGTT TGATTCCGGA
GAGGGAGCCT

....|....| ....|....| ....|....| ....|....| ....|....|
....|....|
245 255 265 275 285
295
D1 GAGAGATGGC TACCAGGACC AAGGTCAGCA GCAGGCGCGA AAATTATCGA
AGCCCGCC-T
D2 GAGAGATGGC TACCAGGACC AAGGTCAGCA GCAGGCGCGA AAATTATCGA
AGCCCGCC-T
D3 GAGAGATGGC TACCAGGACC AAGGTCAGCA GCAGGCGCGA AAATTATCGA
AGCCCGCC-T

R1 GAGAGATGGC TACCAGGACC AAGGTCAGCA GCAGGCGCGA AAATTATCGA
AGCCCGCC-T
R2 GAGAGATGGC TACCAGGACC AAGGTCAGCA GCAGGCGCGA AAATTATCGA
AGCCCGCC-T
R3 GAGAGATGGC TACCAGGACC AAGGTCAGCA GCAGGCGCGA AAATTATCGA
AGCCCGCC-T
R4 GAGAGATGGC TACCAGGACC AAGGTCAGCA GCAGGCGCGA AAATTATCGA
AGCCCGCC-T
R5 GAGAGATGGC TACCAGGACC AAGGTCAGCA GCAGGCGCGA AAATTATCGA
AGCCCGCC-T
R6 GAGAGATGGC TACCAGGACC AAGGTCAGCA GCAGGCGCGA AAATTATCGA
AGCCCGCC-T
R7 GAGAGATGGC TACCAGGACC AAGGTCAGCA GCAGGCGCGA AAATTATCGA
AGCCCGCC-T
N1 GAGAGACGGC TACCAGGACC AAGGTCAGCA GCAGGCGCGA AAATTATCGA
AGCCCGCC-G
N2 GAGAGACGGC TACCAGGACC AAGGTCAGCA GCAGGCGCGA AAATTATCGA
AGCCCGCC-G
N3 GAGAGACGGC TACCAGGACC AAGGTCAGCA GCAGGCGCGA AAATTATCGA
AGCCCGCC-G
M1 GAGAGATGGC TACCAGGACC AAGGTCAGCA GCAGGCGCGA AAATTATCGA
AGCCCGCC-T
M2 GAGAGATGGC TACCAGGACC AAGGTCAGCA GCAGGCGCGA AAATTATCGA
AGCCCGCC-T
M3 GAGAGATGGC TACCAGGACC AAGGTCAGCA GCAGGCGCGA AAATTATCGA
AGCCCGCC-T
M4 GAGAGATGGC TACCAGGACC AAGGTCAGCA GCAGGCGCGA AAATTATCGA
AGCCCGCC-T
M5 GAGAGATGGC TACCAGGACC AAGGTCAGCA GCAGGCGCGA AAATTATCGA
AGCCCGCC-T
M6 GAGAGATGGC TACCAGGACC AAGGTCAGCA GCAGGCGCGA AAATTATCGA
AGCCCGCC-T
M7 GAGAGATGGC TACCAGGACC AAGGTCAGCA GCAGGCGCGA AAATTATCGA
AGCCCGCC-T
M8 GAGAGATGGC TACCAGGACC AAGGTCAGCA GCAGGCGCGA AAATTATCGA
AGCCCGCC-T
M9 GAGAGATGGC TACCAGGACC AAGGTCAGCA GCAGGCGCGA AAATTATCGA
AGCCCGCC-T
M10 GAGAGATGGC TACCAGGACC AAGGTCAGCA GCAGGCGCGA AAATTATCGA
AGCCCGCC-T
M11 GAGAGATGGC TACCAGGACC AAGGTCAGCA GCAGGCGCGA AAATTATCGA
AGCCCGCC-T
M12 GAGAGATGGC TACCAGGACC AAGGTCAGCA GCAGGCGCGA AAATTATCGA
AGCCCGCC-T
M13 GAGAGATGGC TACCAGGACC AAGGTCAGCA GCAGGCGCGA AAATTATCGA
AGCCCGCC-T
M14 GAGAGATGGC TACCAGGACC AAGGTCAGCA GCAGGCGCGA AAATTATCGA
AGCCCGCC-T
M15 GAGAGATGGC TACCAGGACC AAGGTCAGCA GCAGGCGCGA AAATTATCGA
AGCCCGCC-T
M16 GAGAGATGGC TACCAGGACC AAGGTCAGCA GCAGGCGCGA AAATTATCGA
AGCCCGCC-T
M17 GAGAGATGGC TACCAGGACC AAGGTCAGCA GCAGGCGCGA AAATTATCGA
AGCCCGCC-T
M18 GAGAGATGGC TACCAGGACC AAGGTCAGCA GCAGGCGCGA AAATTATCGA
AGCCCGCC-T
M19 GAGAGATGGC TACCAGGACC AAGGTCAGCA GCAGGCGCGA AAATTATCGA
AGCCCGCC-T

M20 GAGAGATGGC TACCAGGACC AAGGTCAGCA GCAGGCGCGA AAATTATCGA
AGCCCGCC-T
M21 GAGAGATGGC TACCAGGACC AAGGTCAGCA GCAGGCGCGA AAATTATCGA
AGCCCGCC-T
M22 GAGAGATGGC TACCAGGACC AAGGTCAGCA GCAGGCGCGA AAATTATCGA
AGCCCGCC-T
M23 GAGAGATGGC TACCAGGACC AAGGTCAGCA GCAGGCGCGA AAATTATCGA
AGCCCGCC-T
B1 GAGAGACGGC TACCAGGACC AAGGTCAGCA GCAGGCGCGA AAATTATCGA
AGCCCGCA-T
B2 GAGAGACGGC TACCAGGACC AAGGTCAGCA GCAGGCGCGA AAATTATCGA
AGCCCGCG-T
B3 GAGAGACGGC TACCAGGACC AAGGTCAGCA GCAGGCGCGA AAATTATCGA
AGCCCGCG-T
B4 GAGAGACGGC TACCAGGACC AAGGTCAGCA GCAGGCGCGA AAATTATCGA
AGCCCGCG-T
B5 GAGAGACGGC TACCAGGACC AAGGTCAGCA GCAGGCGCGA AAATTATCGA
AGCCCGCG-T
B6 GAGAGACGGC TACCAGGACC AAGGTCAGCA GCAGGCGCGA AAATTATCGA
AGCCCGCG-T
B7 GAGAGACGGC TACCAGGACC AAGGTCAGCA GCAGGCGCGA AAATTATCGA
AGCCCGCG-T
B8 GAGAGACGGC TACCAGGACC AAGGTCAGCA GCAGGCGCGA AAATTATCGA
AGCCCGCG-T
B9 GAGAGACGGC TACCAGGACC AAGGTCAGCA GCAGGCGCGA AAATTATCGA
AGCCCGCG-T
B10 GAGAGACGGC TACCAGGACC AAGGTCAGCA GCAGGCGCGA AAATTATCGA
AGCCCGCG-T
B11 GAGAGACGGC TACCAGGACC AAGGTCAGCA GCAGGCGCGA AAATTATCGA
AGCCCGCG-T
B12 GAGAGACGGC TACCAGGACC AAGGTCAGCA GCAGGCGCGA AAATTATCGA
AGCCCGCG-T
B13 GAGAGACGGC TACCAGGACC AAGGTCAGCA GCAGGCGCGA AAATTATCGA
AGCCCGCG-T
B14 GAGAGACGGC TACCAGGACC AAGGTCAGCA GCAGGCGCGA AAATTATCGA
AGCCCGCG-T
B15 GAGAGACGGC TACCAGGACC AAGGTCAGCA GCAGGCGCGA AAATTATCGA
AGCCCGCG-T
B16 GAGAGACGGC TACCAGGACC AAGGTCAGCA GCAGGCGCGA AAATTATCGA
AGCCCGCG-T
KF537632_Ddi GAGAGATGGC TACCAGGACC AAGGTCAGCA GCAGGCGCGA AAATTATCGA
AGCCCACCAT
AJ438961_Dde GAGAGACGGC TACCAGGACC AAGGTCAGCA GCAGGCGCGA AAATTATCGA
AGCCCGCC-T
AJ438960_Dca GAGAGACGGC TACCAGGACC AAGGTCAGCA GCAGGCGCGA AAATTATCGA
AGCCCGCC-T
AJ438959_Dca GAGAGACGGC TACCAGGACC AAGGTCAGCA GCAGGCGCGA AAATTATCGA
AGCCCGCC-T
AJ438958_Dga GAGAGATGGC TACCAGGACC AAGGTCAGCA GCAGGCGCGA AAATTATCGA
AGCCCACT-T

....|....| ....|....| ....|....| ....|....| ....|....|
....|....|
305 315 325 335 345
355
D1 AGGGGCGATA GTGAGGAGAC GTGTATAACG AAGTACGTGT AAAGAACGTG
CTAATAACTG

D2 AGGGGCGATA GTGAGGAGAC GTGTATAACG AAGTACGTGT AAAGAACGTG CTAATAACTG
D3 AGGGGCGATA GTGAGGAGAC GTGTATAACG AAGTACGTGT AAAGAACGTG CTAATAACTG
R1 AGGGGCGATA GTGAGGAGAC GTGTAT-ACG AAGTACGTGT AAAGACCGTA CTAATAACTG
R2 AGGGGCGATA GTGAGGAGAC GTGTTA-ACG AAGTACGTGT AAAGACCGTA CTAATAACTG
R3 AGGGGCGATA GTGAGGAGAC GTGTTA-ACG AAGTACGTGT AAAGACCGTA CTAATAACTG
R4 AGGGGCGATA GTGAGGAGAC GTGTAT-ACG AAGTACGTGT AAAGACCGTA CTAATAACTG
R5 AGGGGCGATA GTGAGGAGAC GTGTAT-ACG AAGTACGTGT AAAGACCGTA CTAATAACTG
R6 AGGGGCGATA GTGAGGAGAC GTGTAT-ACG AAGTACGTGT AAAGACCGTA CTAATAACTG
R7 AGGGGCGATA GTGAGGAGAC GTGTAT-ACG AAGTACGTGT AAAGACCGTA CTAATAACTG
N1 AGGGGCGATA GTGAGGAGAC GTGTATAACG AAGTACGTGT AAAGAACGTG CTAACAACTG
N2 AGGGGCGATA GTGAGGAGAC GTGTATAACG AAGTACGTGT AAAGAACGTG CTAACAACTG
N3 AGGGGCGATA GTGAGGAGAC GTGTATAACG AAGTACGTGT AAAGAACGTG CTAACAACTG
M1 AGGGGCGATA GTGAGGAGAC GTGTATAACG AAGTACGTGT AAAGAACGTA CTAATAACTG
M2 AGGGGCGATA GTGAGGAGAC GTGTATAACG AAGTACGTGT AAAGAACGTA CTAATAACTG
M3 AGGGGCGATA GTGAGGAGAC GTGTATAACG AAGTACGTGT AAAGAACGTA CTAATAACTG
M4 AGGGGCGATA GTGAGGAGAC GTGTATAACG AAGTACGTGT AAAGAACGTA CTAATAACTG
M5 AGGGGCGATA GTGAGGAGAC GTGTATAACG AAGTACGTGT AAAGAACGTA CTAATAACTG
M6 AGGGGCGATA GTGAGGAGAC GTGTATAACG AAGTACGTGT AAAGAACGTA CTAATAACTG
M7 AGGGGCGATA GTGAGGAGAC GTGTATAACG AAGTACGTGT AAAGAACGTA CTAATAACTG
M8 AGGGGCGATA GTGAGGAGAC GTGTATAACG AAGTACGTGT AAAGAACGTA CTAATAACTG
M9 AGGGGCGATA GTGAGGAGAC GTGTATAACG AAGTACGTGT AAAGAACGTA CTAATAACTG
M10 AGGGGCGATA GTGAGGAGAC GTGTATAACG AAGTACGTGT AAAGAACGTA CTAATAACTG
M11 AGGGGCGATA GTGAGGAGAC GTGTATAACG AAGTACGTGT AAAGAACGTA CTAATAACTG
M12 AGGGGCGATA GTGAGGAGAC GTGTATAACG AAGTACGTGT AAAGAACGTA CTAATAACTG
M13 AGGGGCGATA GTGAGGAGAC GTGTATAACG AAGTACGTGT AAAGAACGTA CTAATAACTG
M14 AGGGGCGATA GTGAGGAGAC GTGTATAACG AAGTACGTGT AAAGAACGTA CTAATAACTG
M15 AGGGGCGATA GTGAGGAGAC GTGTATAACG AAGTACGTGT AAAGAACGTA CTAATAACTG
M16 AGGGGCGATA GTGAGGAGAC GTGTATAACG AAGTACGTGT AAAGAACGTA CTAATAACTG
M17 AGGGGCGATA GTGAGGAGAC GTGTATAACG AAGTACGTGT AAAGAACGTA CTAATAACTG

```
M18            AGGGGCGATA GTGAGGAGAC GTGTATAACG AAGTACGTGT AAAGAACGTA
CTAATAACTG
M19            AGGGGCGATA GTGAGGAGAC GTGTATAACG AAGTACGTGT AAAGAACGTA
CTAATAACTG
M20            AGGGGCGATA GTGAGGAGAC GTGTAT-TCG AAGTACGTGT AAAGAACGTA
CTAATAACTG
M21            AGGGGCGATA GTGAGGAGAC GTGTAT-TCG AAGTACGTGT AAAGAACGTA
CTAATAACTG
M22            AGGGGCGATA GTGAGGAGAC GTGTAT-TCG AAGTACGTGT AAAGAACGTA
CTAATAACTG
M23            AGGGGCGATA GTGAGGAGAC GTGTAT-TCG AAGTACGTGT AAAGAACGTA
CTAATAACTG
B1             AGGGGCGATA GTGAGGAGAC GTGTATTACG AAGTGTGTGT AAAGAACGCA
CTAATAACTG
B2             AGGGGCGATA GTGAGGAGAC GTGTATTACG AAGTGTGTGT AAAGAACGCA
CTAATAACTG
B3             AGGGGCGATA GTGAGGAGAC GTGTATTACG AAGTGTGTGT AAAGAACGCA
CTAATAACTG
B4             AGGGGCGATA GTGAGGAGAC GTGTATTACG AAGTGTGTGT AAAGAACGCA
CTAATAACTG
B5             AGGGGCGATA GTGAGGAGAC GTGTATTACG AAGTGTGTGT AAAGAACGCA
CTAATAACTG
B6             AGGGGCGATA GTGAGGAGAC GTGTATTACG AAGTGTGTGT AAAGAACGCA
CTAATAACTG
B7             AGGGGCGATA GTGAGGAGAC GTGTATTACG AAGTGTGTGT AAAGAACGCA
CTAATAACTG
B8             AGGGGCGATA GTGAGGAGAC GTGTATTACG AAGTGTGTGT AAAGAACGCA
CTAATAACTG
B9             AGGGGCGATA GTGAGGAGAC GTGTATTACG AAGTGTGTGT AAAGAACGCA
CTAATAACTG
B10            AGGGGCGATA GTGAGGAGAC GTGTATTACG AAGTGTGTGT AAAGAACGCA
CTAATAACTG
B11            AGGGGCGATA GTGAGGAGAC GTGTATTACG AAGTGTGTGT AAAGAACGCA
CTAATAACTG
B12            AGGGGCGATA GTGAGGAGAC GTGTATTACG AAGTGTGTGT AAAGAACGCA
CTAATAACTG
B13            AGGGGCGATA GTGAGGAGAC GTGTATTACG AAGTGTGTGT AAAGAACGCA
CTAATAACTG
B14            AGGGGCGATA GTGAGGAGAC GTGTATTACG AAGTGTGTGT AAAGAACGCA
CTAATAACTG
B15            AGGGGCGATA GTGAGGAGAC GTGTATTACG AAGTGTGTGT AAAGAACGCA
CTAATAACTG
B16            AGGGGCGATA GTGAGGAGAC GTGTATTACG AAGTGTGTGT AAAGAACGCA
CTAATAACTG
KF537632_Ddi   TGGGGCGATA GTGAGGAGAC GTGTATAACG AAATACGGGT AAAGAACGTA
TGTATAACTG
AJ438961_Dde   AGGGGCGATA GTGAGGAGAC GTGAAT-TGT AGGTGCGGGT AAAAAACGCA
CTAGCAACTG
AJ438960_Dca   AGGGGCGATA GTGAGGAGAC GTGAAT-TTT AGGTGCGTGT AAAGAACGCA
CTAGCAACTG
AJ438959_Dca   AGGGGCGATA GTGAGGAGAC GTGAAT-TTT AGGTGCGTGT AAAGAACGCA
CTAGCAACTG
AJ438958_Dga   TGGGGCGATA GTGAGGAGAC GTAAAT-ATG ATGTACGGGT AAAGAACGTA
CTATAAACTG

               ....|....| ....|....| ....|....| ....|....| ....|....|
....|....|
```

365 375 385 395 405
415
D1 GAGGTCAAGT CTGGTGCCAG CATCCGCGGT AATTCCAGCT CCAGGGGTGT
CTATGATGAT
D2 GAGGTCAAGT CTGGTGCCAG CATCCGCGGT AATTCCAGCT CCAGGGGTGT
CTATGATGAT
D3 GAGGTCAAGT CTGGTGCCAG CATCCGCGGT AATTCCAGCT CCAGGGGTGT
CTATGATGAT
R1 GAGGTCAAGT CTGGTGCCAG CATCCGCGGT AATTCCAGCT CCAGGGGTGT
CTATGATGAT
R2 GAGGTCAAGT CTGGTGCCAG CATCCGCGGT AATTCCAGCT CCAGGGGTGT
CTATGATGAT
R3 GAGGTCAAGT CTGGTGCCAG CATCCGCGGT AATTCCAGCT CCAGGGGTGT
CTATGATGAT
R4 GAGGTCAAGT CTGGTGCCAG CATCCGCGGT AATTCCAGCT CCAGGGGTGT
CTATGATGAT
R5 GAGGTCAAGT CTGGTGCCAG CATCCGCGGT AATTCCAGCT CCAGGGGTGT
CTATGATGAT
R6 GAGGTCAAGT CTGGTGCCAG CATCCGCGGT AATTCCAGCT CCAGGGGTGT
CTATGATGAT
R7 GAGGTCAAGT CTGGTGCCAG CATCCGCGGT AATTCCAGCT CCAGGGGTGT
CTATGATGAT
N1 GAGGTCAAGT CTGGTGCCAG CATCCGCGGT AATTCCAGCT CCAGGGGTGT
CTATGATGAT
N2 GAGGTCAAGT CTGGTGCCAG CATCCGCGGT AATTCCAGCT CCAGGGGTGT
CTATGATGAT
N3 GAGGTCAAGT CTGGTGCCAG CATCCGCGGT AATTCCAGCT CCAGGGGTGT
CTATGATGAT
M1 GAGGTCAAGT CTGGTGCCAG CATCCGCGGT AATTCCAGCT CCAGGGGTGT
CTATGATGAT
M2 GAGGTCAAGT CTGGTGCCAG CATCCGCGGT AATTCCAGCT CCAGGGGTGT
CTATGATGAT
M3 GAGGTCAAGT CTGGTGCCAG CATCCGCGGT AATTCCAGCT CCAGGGGTGT
CTATGATGAT
M4 GAGGTCAAGT CTGGTGCCAG CATCCGCGGT AATTCCAGCT CCAGGGGTGT
CTATGATGAT
M5 GAGGTCAAGT CTGGTGCCAG CATCCGCGGT AATTCCAGCT CCAGGGGTGT
CTATGATGAT
M6 GAGGTCAAGT CTGGTGCCAG CATCCGCGGT AATTCCAGCT CCAGGGGTGT
CTATGATGAT
M7 GAGGTCAAGT CTGGTGCCAG CATCCGCGGT AATTCCAGCT CCAGGGGTGT
CTATGATGAT
M8 GAGGTCAAGT CTGGTGCCAG CATCCGCGGT AATTCCAGCT CCAGGGGTGT
CTATGATGAT
M9 GAGGTCAAGT CTGGTGCCAG CATCCGCGGT AATTCCAGCT CCAGGGGTGT
CTATGATGAT
M10 GAGGTCAAGT CTGGTGCCAG CATCCGCGGT AATTCCAGCT CCAGGGGTGT
CTATGATGAT
M11 GAGGTCAAGT CTGGTGCCAG CATCCGCGGT AATTCCAGCT CCAGGGGTGT
CTATGATGAT
M12 GAGGTCAAGT CTGGTGCCAG CATCCGCGGT AATTCCAGCT CCAGGGGTGT
CTATGATGAT
M13 GAGGTCAAGT CTGGTGCCAG CATCCGCGGT AATTCCAGCT CCAGGGGTGT
CTATGATGAT
M14 GAGGTCAAGT CTGGTGCCAG CATCCGCGGT AATTCCAGCT CCAGGGGTGT
CTATGATGAT
M15 GAGGTCAAGT CTGGTGCCAG CATCCGCGGT AATTCCAGCT CCAGGGGTGT
CTATGATGAT

M16 GAGGTCAAGT CTGGTGCCAG CATCCGCGGT AATTCCAGCT CCAGGGGTGT
CTATGATGAT
M17 GAGGTCAAGT CTGGTGCCAG CATCCGCGGT AATTCCAGCT CCAGGGGTGT
CTATGATGAT
M18 GAGGTCAAGT CTGGTGCCAG CATCCGCGGT AATTCCAGCT CCAGGGGTGT
CTATGATGAT
M19 GAGGTCAAGT CTGGTGCCAG CATCCGCGGT AATTCCAGCT CCAGGGGTGT
CTATGATGAT
M20 GAGGTCAAGT CTGGTGCCAG CATCCGCGGT AATTCCAGCT CCAGGGGTGT
CTATGATGAT
M21 GAGGTCAAGT CTGGTGCCAG CATCCGCGGT AATTCCAGCT CCAGGGGTGT
CTATGATGAT
M22 GAGGTCAAGT CTGGTGCCAG CATCCGCGGT AATTCCAGCT CCAGGGGTGT
CTATGATGAT
M23 GAGGTCAAGT CTGGTGCCAG CATCCGCGGT AATTCCAGCT CCAGGGGTGT
CTATGATGAT
B1 GAGGTCAAGT CTGGTGCCAG CATCCGCGGT AATTCCAGCT CCAGGGGTGT
CTATGATGAT
B2 GAGGTCAAGT CTGGTGCCAG CATCCGCGGT AATTCCAGCT CCAGGGGTGT
CTATGATGAT
B3 GAGGTCAAGT CTGGTGCCAG CATCCGCGGT AATTCCAGCT CCAGGGGTGT
CTATGATGAT
B4 GAGGTCAAGT CTGGTGCCAG CATCCGCGGT AATTCCAGCT CCAGGGGTGT
CTATGATGAT
B5 GAGGTCAAGT CTGGTGCCAG CATCCGCGGT AATTCCAGCT CCAGGGGTGT
CTATGATGAT
B6 GAGGTCAAGT CTGGTGCCAG CATCCGCGGT AATTCCAGCT CCAGGGGTGT
CTATGATGAT
B7 GAGGTCAAGT CTGGTGCCAG CATCCGCGGT AATTCCAGCT CCAGGGGTGT
CTATGATGAT
B8 GAGGTCAAGT CTGGTGCCAG CATCCGCGGT AATTCCAGCT CCAGGGGTGT
CTATGATGAT
B9 GAGGTCAAGT CTGGTGCCAG CATCCGCGGT AATTCCAGCT CCAGGGGTGT
CTATGATGAT
B10 GAGGTCAAGT CTGGTGCCAG CATCCGCGGT AATTCCAGCT CCAGGGGTGT
CTATGATGAT
B11 GAGGTCAAGT CTGGTGCCAG CATCCGCGGT AATTCCAGCT CCAGGGGTGT
CTATGATGAT
B12 GAGGTCAAGT CTGGTGCCAG CATCCGCGGT AATTCCAGCT CCAGGGGTGT
CTATGATGAT
B13 GAGGTCAAGT CTGGTGCCAG CATCCGCGGT AATTCCAGCT CCAGGGGTGT
CTATGATGAT
B14 GAGGTCAAGT CTGGTGCCAG CATCCGCGGT AATTCCAGCT CCAGGGGTGT
CTATGATGAT
B15 GAGGTCAAGT CTGGTGCCAG CATCCGCGGT AATTCCAGCT CCAGGGGTGT
CTATGATGAT
B16 GAGGTCAAGT CTGGTGCCAG CATCCGCGGT AATTCCAGCT CCAGGGGTGT
CTATGATGAT
KF537632_Ddi GAGGTCAAGT CTGGTGCCAG CATCCGCGGT AATTCCAGCT CCAGGGGTGT
CTATGATGAT
AJ438961_Dde GAGGTCAAGT CTGGTGCCAG CATCCGCGGT AATTCCAGCT CCAGGGGTGT
CTATGATGAT
AJ438960_Dca GAGGTCAAGT CTGGTGCCAG CATCCGCGGT AATTCCAGCT CCAGGGGTGT
CTATGATGAT
AJ438959_Dca GAGGTCAAGT CTGGTGCCAG CATCCGCGGT AATTCCAGCT CCAGGGGTGT
CTATGATGAT
AJ438958_Dga GAGGTCAAGT CTGGTGCCAG CATCCGCGGT AATTCCAGCT CCAGGGGTGT
CTATGATGAT

```
               ....|....| ....|....| ....|....| ....|....| ....|....|
....|....|
                    425        435        445        455        465
475
D1             TGCTGCGATT AAAAAGTCCG TAGTCAATCT GACTGACTGA CCTGCAATGT
GAT-TGATTA
D2             TGCTGCGATT AAAAAGTCCG TAGTCAATCT GACTGACTGA CCTGCAATGT
GAT-TGATTA
D3             TGCTGCGATT AAAAAGTCCG TAGTCAATCT GACTGACTGA CCTGCAATGT
GAT-TGATTA
R1             TGCTGCGATT AAAAAGTCCG TAGTCGAGCT GACTGACTGA CCTGCAATGT
GAT-TGATTA
R2             TGCTGCGATT AAAAAGTCCG TAGTCGAGCT GACTGACTGA CCTGCAATGT
GAT-TGATTA
R3             TGCTGCGATT AAAAAGTCCG TAGTCGAGCT GACTGACTGA CCTGCAATGT
GAT-TGATTA
R4             TGCTGCGATT AAAAAGTCCG TAGTCGAGCT GACTGACTGA CCTGCAATGT
GAT-TGATTA
R5             TGCTGCGATT AAAAAGTCCG TAGTCGAGCT GACTGACTGA CCTGCAATGT
GGT-TGATTA
R6             TGCTGCGATT AAAAAGTCCG TAGTCGAGCT GACTGACTGA CCTGCAATGT
GAT-TGATTA
R7             TGCTGCGATT AAAAAGTCCG TAGTCGAGCT GACTGACTGA CCTGCAATGT
GAT-TGATTA
N1             TGCTGCGATT AAAAAGTCCG TAGTCAATCT GTCTGACTTG CCTGCAATGT
GAT-TGATTA
N2             TGCTGCGATT AAAAAGTCCG TAGTCAATCT GTCTGACTTG CCTGCAATGT
GAT-TGATTA
N3             TGCTGCGATT AAAAAGTCCG TAGTCAATCT GTCTGACTTG CCTGCAATGT
GAT-TGATTA
M1             TGCTGCGATT AAAAAGTCCG TAGTCAAACT GACTGACTGA CCTGCAATGT
GAT-TGATTA
M2             TGCTGCGATT AAAAAGTCCG TAGTCAAACT GACTGACTGA CCTGCAATGT
GAT-TGATTA
M3             TGCTGCGATT AAAAAGTCCG TAGTCAAACT GACTGACTGA CCTGCAATGT
GAT-TGATTA
M4             TGCTGCGATT AAAAAGTCCG TAGTCAAACT GACTGACTGA CCTGCAATGT
GAT-TGATTA
M5             TGCTGCGATT AAAAAGTCCG TAGTCAAACT GACTGACTGA CCTGCAATGT
GAT-TGATTA
M6             TGCTGCGATT AAAAAGTCCG TAGTCAAACT GACTGACTGA CCTGCAATGT
GAT-TGATTA
M7             TGCTGCGATT AAAAAGTCCG TAGTCAAACT GACTGACTGA CCTGCAATGT
GAT-TGATTA
M8             TGCTGCGATT AAAAAGTCCG TAGTCAAACT GACTGACTGA CCTGCAATGT
GAT-TGATTA
M9             TGCTGCGATT AAAAAGTCCG TAGTCAAACT GACTGACTGA CCTGCAATGT
GAT-TGATTA
M10            TGCTGCGATT AAAAAGTCCG TAGTCAAACT GACTGACTGA CCTGCAATGT
GAT-TGATTA
M11            TGCTGCGATT AAAAAGTCCG TAGTCAAACT GACTGACTGA CCTGCAATGT
GAT-TGATTA
M12            TGCTGCGATT AAAAAGTCCG TAGTCAAACT GACTGACTGA CCTGCAATGT
GAT-TGATTA
M13            TGCTGCGATT AAAAAGTCCG TAGTCAAACT GACTGACTGA CCTGCAATGT
GAT-TGATTA
```

M14 TGCTGCGATT AAAAAGTCCG TAGTCAAGCT GCCTGACTGA CCTGCAATGT
GAT-TGATTA
M15 TGCTGCGATT AAAAAGTCCG TAGTCAAGCT GCCTGACTGA CCTGCAATGT
GAT-TGATTA
M16 TGCTGCGATT AAAAAGTCCG TAGTCAAGCT GCCTGACTGA CCTGCAATGT
GAT-TGATTA
M17 TGCTGCGATT AAAAAGTCCG TAGTCAAGCT GCCTGACTGA CCTGCAATGT
GAT-TGATTA
M18 TGCTGCGATT AAAAAGTCCG TAGTCAAGCT GACTGACTGA CCTGCAATGT
GAT-TGATTA
M19 TGCTGCGATT AAAAAGTCCG TAGTCAAGCT GACTGACTGA CCTGCAATGT
GAT-TGATTA
M20 TGCTGCGATT AAAAAGTCCG TAGTCAAGCT GACTGACTGA CCTGCAATGT
GAT-TGATTA
M21 TGCTGCGATT AAAAAGTCCG TAGTCAAGCT GACTGACTGA CCTGCAATGT
GAT-TGATTA
M22 TGCTGCGATT AAAAAGTCCG TAGTCAAGCT GACTGACTGA CCTGCAATGT
GAT-TGATTA
M23 TGCTGCGATT AAAAAGTCCG TAGTCAAGCT GACTGACTGA CCTGCAATGT
GAT-TGATTA
B1 TGCTGCGATT AAAAAGTCCG TAGTCAAGCT GACTGACTTG CCTGCAATGT
GAC-TGATTA
B2 TGCTGCGATT AAAAAGTCCG TAGTCAAGCT GACTGACTTG CCTGCAATGT
GAC-TGATTA
B3 TGCTGCGATT AAAAAGTCCG TAGTCAAGCT GACTGACTTG CCTGCAATGT
GAC-TGATTA
B4 TGCTGCGATT AAAAAGTCCG TAGTCAAGCT GACTGACTTG CCTGCAATGT
GAC-TGATTA
B5 TGCTGCGATT AAAAAGTCCG TAGTCAAGCT GACTGACTTG CCTGCAATGT
GAC-TGATTA
B6 TGCTGCGATT AAAAAGTCCG TAGTCAAGCT GACTGACTTG CCTGCAATGT
GAC-TGATTA
B7 TGCTGCGATT AAAAAGTCCG TAGTCAAGCT GACTGACTTG CCTGCAATGT
GAC-TGATTA
B8 TGCTGCGATT AAAAAGTCCG TAGTCAAGCT GACTGACTTG CCTGCAATGT
GAC-TGATTA
B9 TGCTGCGATT AAAAAGTCCG TA-TCAAGCT GACTGACTTG CCTGCAATGT
GAC-TGATTA
B10 TGCTGCGATT AAAAAGTCCG TAGTCAAGCT GACTGACTTG CCTGCAATGT
GAC-TGATTA
B11 TGCTGCGATT AAAAAGTCCG TAGTCAAGCT GACTGACTTG CCTGCAATGT
GAC-TGATTA
B12 TGCTGCGATT AAAAAGTCCG TAGTCAAGCT GACTGACTTG CCTGCAATGT
GAC-TGATTA
B13 TGCTGCGATT AAAAAGTCCG TAGTCAAGCT GACTGACTTG CCTGCAATGT
GAC-TGATTA
B14 TGCTGCGATT AAAAAGTCCG TAGTCAAGCT GACTGACTTG CCTGCAATGT
GAC-TGATTA
B15 TGCTGCGATT AAAAAGTCCG TAGTCAAGCT GACTGACTTG CCTGCAATGT
GAC-TGATTA
B16 TGCTGCGATT AAAAAGTCCG TAGTCAAGCT GACTGACTTG CCTGCAATGT
GAC-TGATTA
KF537632_Ddi TGCTGCGATT AAAAAGTCCG TAGTCAAGCT GACTGACTAA CCTGTAATGT
GGT-TGATTA
AJ438961_Dde TGCTGCGATT AAAAAGTCCG TAGTCAAGCC GCCAGACCAG TCTGGAATGT
TTC-TGATCA
AJ438960_Dca TGCTGCGATT AAAAAGTCCG TAGTCAAGCC GCCAGACCAG TCTGGAATGT
TTCTTGATCA

```
AJ438959_Dca    TGCTGCGATT AAAAAGTCCG TAGTCAAGCC GCCAGACCAG TCTGGAATGT
TTCTTGATCA
AJ438958_Dga    TGCTGCGATT AAAAAGTCCG TAGTCTAGCC GCACGACCAG TCTGGAATGT
TTC-TGATCA

                ....|....| ....|....| ....|....| ....|....| ....|....|
....|....|
                      485        495        505        515        525
535
D1              AAGAACGAGC AGGGTTAGGA AAGCAGAGAA TTAGGAGCGA CCGAGGGCTA
GAGTATTGAA
D2              AAGAACGAGC AGGGTTAGGA AAGCAGAGAA TTAGGAGCGA CCGAGGGCTA
GAGTATTGAA
D3              AAGAACGAGC AGGGTTAGGA AAGCAAAGAA TTAGGAGCGA CCGAGGGCTA
GAGTATTGAA
R1              AAGAACGAGC AGGGTTAGGA AAGCAGAGAA TTAGGAGCGA CCGAGGGCTA
GAGTATTGAA
R2              AAGAACGAGC AGGGTTAGGA AAGCAGAGGA TTAGGAGCGA CCGAGGGCTA
GAGTATTGAA
R3              AAGAACGAGC AGGGTTAGGA AAGCAGAGGA TTAGGAGCGA CCGAGGGCTA
GAGTATTGAA
R4              AAGAACGAGC AGGGTTAGGA AAGCAGAGGA TTAGGAGCGA CCGAGGGCTA
GAGTATTGAA
R5              AAAGACGAGC AGGGTTAGGA AAGCAGAGAA TTAGGAGCGA CCGAGGGCTA
GAGTATTGAA
R6              AAGAACGAGC AGGGTTAGGA AAGCAGAGGA TTAGGAGCGA CCGAGGGCTA
GAGTATTGAA
R7              AAGAACGAGC AGGGTTAGGA AAGCAGAGGA TTAGGAGCGA CCGAGGGCTA
GAGTATTGAA
N1              AAGAACGAGC AGGGCTAGGA AAGCAGAGAA TTAGGAGCGA CCGAGGGCTA
GAGTATTGAA
N2              AAGAACGAGC AGGGCTAGGA AAGCAGAGAA TTAGGAGCGA CCGAGGGCTA
GAGTATTGAA
N3              AAGAACGAGC AGGGCTAGGA AAGCAGAGAA TTAGGAGCGA CCGAGGGCTA
GAGTATTGAA
M1              AAGAACGAGC AGGGTTAGGA AAGCAGAGAA TTAGGAGCGA CCGAGGGCTA
GAGTATTGAA
M2              AAGAACGAGC AGGGTTAGGA AAGCAGAGAA TTAGGAGCGA CCGAGGGCTA
GAGTATTGAA
M3              AAGAACGAGC AGGGTTAGGA AAGCAGAGAA TTAGGAGCGA CCGAGGGCTA
GAGTATTGAA
M4              AAGAACGAGC AGGGTTAGGA AAGCAGAGAA TTAGGAGCGA CCGAGGGCTA
GAGTATTGAA
M5              AAGAACGAGC AGGGTTAGGA AAGCAGAGAA TTAGGAGCGA CCGAGGGCTA
GAGTATTGAA
M6              AAGAACGAGC AGGGTTAGGA AAGCAGAGAA TTAGGAGCGA CCGAGGGCTA
GAGTATTGAA
M7              AAGAACGAGC AGGGTTAGGA AAGCAGAGAA TTAGGAGCGA CCGAGGGCTA
GAGTATTGAA
M8              AAGAACGAGC AGGGTTAGGA AAGCAGAGAA TTAGGAGCGA CCGAGGGCTA
GAGTATTGAA
M9              AAGAACGAGC AGGGTTAGGA AAGCAGAGAA TTAGGAGCGA CCGAGGGCTA
GAGTATTGAA
M10             AAGAACGAGC AGGGTTAGGA AAGCAGAGAA TTAGGAGCGA CCGAGGGCTA
GAGTATTGAA
M11             AAGAACGAGC AGGGTTAGGA AAGCAGAGAA TTAGGAGCGA CCGAGGGCTA
GAGTATTGAA
```

M12 AAGAACGAGC AGGGTTAGGA AAGCAGAGAA TTAGGAGCGA CCGAGGGCTA
GAGTATTGAA
M13 AAGAACGAGC AGGGTTAGGA AAGCAGAGAA TTAGGAGCGA CCGAGGGCTA
GAGTATTGAA
M14 AGGAACGAGC AGGGTTAGGA AAGCAGAGAA TTAGGAGCGA CCGAGGGCTA
GAGTATTGAA
M15 AGGAACGAGC AGGGTTAGGA AAGCAGAGAA TTAGGAGCGA CCGAGGGCTA
GAGTATTGAA
M16 AGGAACGAGC AGGGTTAGGA AAGCAGAGAA TTAGGAGCGA CCGAGGGCTA
GAGTATTGAA
M17 AGGAACGAGC AGGGTTAGGA AAGCAGAGAA TTAGGAGCGA CCGAGGGCTA
GAGTATTGAA
M18 AGGAACGAGC AGGGTTAGGA AAGCAGAGAA TTAGGAGCGA CCGAGGGCTA
GAGTATTGAA
M19 AGGAACGAGC AGGGTTAGGA AAGCAGAGAA TTAGGAGCGA CCGAGGGCTA
GAGTATTGAA
M20 AAGAACGAGC AGGGTTAGGA AAGCAGAGAA TTAGGAGCGA CCGAGGGCTA
GAGTATTGAA
M21 AAGAACGAGC AGGGTTAGGA AAGCAGAGAA TTAGGAGCGA CCGAGGGCTA
GAGTATTGAA
M22 AAGAACGAGC AGGGTTAGGA AAGCAGAGAA TTAGGAGCGA CCGAGGGCTA
GAGTATTGAA
M23 AAGAACGAGC AGGGTTAGGA AAGCAGAGAA TTAGGAGCGA CCGAGGGCTA
GAGTATTGAA
B1 AGAGACGAGC AGGGCTAGGA AAGCAGAGAA TTAGGAGCGA CCGAGGGCTA
GAGTATTGAA
B2 AGAGACGAGC AGGGCTAGGA AAGCAGAGAA TTAGGAGCGA CCGAGGGCTA
GAGTATTGAA
B3 AGAGACGAGC AGGGCTAGGA AAGCAGAGAA TTAGGAGCGA CCGAGGGCTA
GAGTATTGAA
B4 AGAGACGAGC AGGGCTAGGA AAGCAGAGAA TTAGGAGCGA CCGAGGGCTA
GAGTATTGAA
B5 AGAGACGAGC AGGGCTAGGA AAGCAGAGAA TTAGGAGCGA CCGAGGGCTA
GAGTATTGAA
B6 AGAGACGAGC AGGGCTAGGA AAGCAGAGAA TTAGGAGCGA CCGAGGGCTA
GAGTATTGAA
B7 AGAGACGAGC AGGGCTAGGA AAGCAGAGAA TTAGGAGCGA CCGAGGGCTA
GAGTATTGAA
B8 AGAGACGAGC AGGGCTAGGA AAGCAGAGAA TTAGGAGCGA CCGAGGGCTA
GAGTATTGAA
B9 AGAGACGAGC AGGGCTAGGA AAGCAGAGAA TTAGGAGCGA CCGAGGGCTA
GAGTATTGAA
B10 AGAGACGAGC AGGGCTAGGA AAGCAGAGAA TTAGGAGCGA CCGAGGGCTA
GAGTATTGAA
B11 AGAGACGAGC AGGGCTAGGA AAGCAGAGAA TTAGGAGCGA CCGAGGGCTA
GAGTATTGAA
B12 AGAGACGAGC AGGGCTAGGA AAGCAGAGAA TTAGGAGCGA CCGAGGGCTA
GAGTATTGAA
B13 AGAGACGAGC AGGGCTAGGA AAGCAGAGAA TTAGGAGCGA CCGAGGGCTA
GAGTATTGAA
B14 AGAGACGAGC AGGGCTAGGA AAGCAGAGAA TTAGGAGCGA CCGAGGGCTA
GAGTATTGAA
B15 AGAGACGAGC AGGGCTAGGA AAGCAGAGAA TTAGGAGCGA CCGAGGGCTA
GAGTATTGAA
B16 AGAGACGAGC AGGGCTAGGA AAGCAGAGAA TTAGGAGCGA CCGAGGGCTA
GAGTATTGAA
KF537632_Ddi AAAGACGAGA AGGGTTAGGA AAGCAGAGGA TTAGGAGCGA CCGAGGGCTA
GAGTATTGAA

AJ438961_Dde   AGGGACGAGC AGGGCTGGGA AAGCGGAGAA TTAGGAGCGA CCGAGGGCTA
GAGTATTGGG
AJ438960_Dca   AGAGACGAGC AGGGCTGGGA AAGCGGAGAA TTAGGAGCGA CCGAGGGCTA
GAGTATTGGG
AJ438959_Dca   AGAGACGAGC AGGGCTGGGA AAGCGGAGAA TTAGGAGCGA CCGAGGGCTA
GAGTATTGGG
AJ438958_Dga   AAAGAAGAGC AGGGCTGGGA AGGCGGAGAA TTAGGAGCGA CCAGGGGCTA
GAGTATTGAA

....|....| ....|....| ....|....| ....|....| ....|....|
....|....|
545 555 565 575 585
595
D1 TGGCGAGAGG TGAAATTTGA TGACCCATTC AGGAGTGACA AAGGCGAAGG
CACTAGTCAA
D2 TGGCGAGAGG TGAAATTTGA TGACCCATTC AGGAGTGACA AAGGCGAAGG
CACTAGTCAA
D3 TGGCGAGAGG TGAAATTTGA TGACCCATTC AGGAGTGACA AAGGCGAAGG
CACTAGTCAA
R1 TGGCGAGAGG TGAAATTTGA TGACCCATTC AGGAGTGACA AAGGCGAAGG
CACTAGTCAA
R2 TGGCGAGAGG TGAAATTTGA TGACCCATTC AGGAGTGACA AAGGCGAAGG
CACTAGTCAA
R3 TGGCGAGAGG TGAAATTTGA TGACCCATTC AGGAGTGACA AAGGCGAAGG
CACTAGTCAA
R4 TGGCGAGAGG TGAAATTTGA TGACCCATTC AGGAGTGACA AAGGCGAAGG
CACTAGTCAA
R5 TGGCGAGAGG TGAAATTTGA TGACCCATTC AGGAGTGACA AAGGCGAAGG
CACTAGTCAA
R6 TGGCGAGAGG TGAAATTTGA TGACCCATTC AGGAGTGACA AAGGCGAAGG
CACTAGTCAA
R7 TGGCGAGAGG TGAAATTTGA TGACCCATTC AGGAGTGACA AAGGCGAAGG
CACTAGTCAA
N1 TGGCGAGAGG TGAAATTTGA TGACCCATTC AGGAGTGACA AAGGCGAAGG
CACTAGTCAA
N2 TGGCGAGAGG TGAAATTTGA TGACCCATTC AGGAGTGACA AAGGCGAAGG
CACTAGTCAA
N3 TGGCGAGAGG TGAAATTTGA TGACCCATTC AGGAGTGACA AAGGCGAAGG
CACTAGTCAA
M1 TGGCGAGAGG TGAAATTTGA TGACCCATTC AGGAGTGACA AAGGCGAAGG
CACTAGTCAA
M2 TGGCGAGAGG TGAAATTTGA TGACCCATTC AGGAGTGACA AAGGCGAAGG
CACTAGTCAA
M3 TGGCGAGAGG TGAAATTTGA TGACCCATTC AGGAGTGACA AAGGCGAAGG
CACTAGTCAA
M4 TGGCGAGAGG TGAAATTTGA TGACCCATTC AGGAGTGACA AAGGCGAAGG
CACTAGTCAA
M5 TGGCGAGAGG TGAAATTTGA TGACCCATTC AGGAGTGACA AAGGCGAAGG
CACTAGTCAA
M6 TGGCGAGAGG TGAAATTTGA TGACCCATTC AGGAGTGACA AAGGCGAAGG
CACTAGTCAA
M7 TGGCGAGAGG TGAAATTTGA TGACCCATTC AGGAGTGACA AAGGCGAAGG
CACTAGTCAA
M8 TGGCGAGAGG TGAAATTTGA TGACCCATTC AGGAGTGACA AAGGCGAAGG
CACTAGTCAA
M9 TGGCGAGAGG TGAAATTTGA TGACCCATTC AGGAGTGACA AAGGCGAAGG
CACTAGTCAA

M10 TGGCGAGAGG TGAAATTTGA TGACCCATTC AGGAGTGACA AAGGCGAAGG
CACTAGTCAA
M11 TGGCGAGAGG TGAAATTTGA TGACCCATTC AGGAGTGACA AAGGCGAAGG
CACTAGTCAA
M12 TGGCGAGAGG TGAAATTTGA TGACCCATTC AGGAGTGACA AAGGCGAAGG
CACTAGTCAA
M13 TGGCGAGAGG TGAAATTTGA TGACCCATTC AGGAGTGACA AAGGCGAAGG
CACTAGTCAA
M14 TGGCGAGAGG TGAAATTTGA TGACCCATTC AGGAGTGACA AAGGCGAAGG
CACTAGTCAA
M15 TGGCGAGAGG TGAAATTTGA TGACCCATTC AGGAGTGACA AAGGCGAAGG
CACTAGTCAA
M16 TGGCGAGAGG TGAAATTTGA TGACCCATTC AGGAGTGACA AAGGCGAAGG
CACTAGTCAA
M17 TGGCGAGAGG TGAAATTTGA TGACCCATTC AGGAGTGACA AAGGCGAAGG
CACTAGTCAA
M18 TGGCGAGAGG TGAAATTTGA TGACCCATTC AGGAGTGACA AAGGCGAAGG
CACTAGTCAA
M19 TGGCGAGAGG TGAAATTTGA TGACCCATTC AGGAGTGACA AAGGCGAAGG
CACTAGTCAA
M20 TGGCGAGAGG TGAAATTTGA TGACCCATTC AGGAGTGACA AAGGCGAAGG
CACTAGTCAA
M21 TGGCGAGAGG TGAAATTTGA TGACCCATTC AGGAGTGACA AAGGCGAAGG
CACTAGTCAA
M22 TGGCGAGAGG TGAAATTTGA TGACCCATTC AGGAGTGACA AAGGCGAAGG
CACTAGTCAA
M23 TGGCGAGAGG TGAAATTTGA TGACCCATTC AGGAGTGACA AAGGCGAAGG
CACTAGTCAA
B1 TGGCGAGAGG TGAAATTTGA TGACCCATTC AGGAGTGACA AAGGCGAAGG
CACTAGTCAA
B2 TGGCGAGAGG TGAAATTTGA TGACCCATTC AGGAGTGACA AAGGCGAAGG
CACTAGTCAA
B3 TGGCGAGAGG TGAAATTTGA TGACCCATTC AGGAGTGACA AAGGCGAAGG
CACTAGTCAA
B4 TGGCGAGAGG TGAAATTTGA TGACCCATTC AGGAGTGACA AAGGCGAAGG
CACTAGTCAA
B5 TGGCGAGAGG TGAAATTTGA TGACCCATTC AGGAGTGACA AAGGCGAAGG
CACTAGTCAA
B6 TGGCGAGAGG TGAAATTTGA TGACCCATTC AGGAGTGACA AAGGCGAAGG
CACTAGTCAA
B7 TGGCGAGAGG TGAAATTTGA TGACCCATTC AGGAGTGACA AAGGCGAAGG
CACTAGTCAA
B8 TGGCGAGAGG TGAAATTTGA TGACCCATTC AGGAGTGACA AAGGCGAAGG
CACTAGTCAA
B9 TGGCGAGAGG TGAAATTTGA TGACCCATTC AGGAGTGACA AAGGCGAAGG
CACTAGTCAA
B10 TGGCGAGAGG TGAAATTTGA TGACCCATTC AGGAGTGACA AAGGCGAAGG
CACTAGTCAA
B11 TGGCGAGAGG TGAAATTTGA TGACCCATTC AGGAGTGACA AAGGCGAAGG
CACTAGTCAA
B12 TGGCGAGAGG TGAAATTTGA TGACCCATTC AGGAGTGACA AAGGCGAAGG
CACTAGTCAA
B13 TGGCGAGAGG TGAAATTTGA TGACCCATTC AGGAGTGACA AAGGCGAAGG
CACTAGTCAA
B14 TGGCGAGAGG TGAAATTTGA TGACCCATTC AGGAGTGACA AAGGCGAAGG
CACTAGTCAA
B15 TGGCGAGAGG TGAAATTTGA TGACCCATTC AGGAGTGACA AAGGCGAAGG
CACTAGTCAA

```
B16             TGGCGAGAGG TGAAATTTGA TGACCCATTC AGGAGTGACA AAGGCGAAGG
CACTAGTCAA
KF537632_Ddi    TGGCGAGAGG TGAAATTTGA TGACCCATTC AGGAGTGACA AAGGCGAAGG
CACTAGTCAA
AJ438961_Dde    TGGCGAGAGG TGAAATTTGA TGACCCATCC AGGAGTGACA AAGGCGAAGG
CACTAGTCAA
AJ438960_Dca    TGGCGAGAGG TGAAATTTGA TGACCCATCC AGGAGTGACA AAGGCGAAGG
CACTAGTCAA
AJ438959_Dca    TGGCGAGAGG TGAAATTTGA TGACCCATCC AGGAGTGACA AAGGCGAAGG
CACTAGTCAA
AJ438958_Dga    TGGCGAGAGG TGAAATTTGA TGACCCATTC AGGAGTGACA AAGGCGAAGG
CACTAGTCAG

                ....|....| ....|....| ....|....| ....|....| ....|....|
....|....|
                   605        615        625        635        645
655
D1              GGGCGAATCC GATGATCAAG GACGTAGGCT AGAGTTTCGA AAACGATTAG
AGACCGGAGT
D2              GGGCGAATCC GATGATCAAG GACGTAGGCT AGAGTTTCGA AAACGATTAG
AGACCGGAGT
D3              GGGCGAATCC GATGATCAAG GACGTAGGCT AAAGTTTCGA AAACGATTAG
AGACCGGAGT
R1              GGGCGAATCC GATGATCAAG GACGTAGGCT AGAGTTTCGA AAACGATTAG
AGACCGGAGT
R2              GGGCGAATCC GATGATCAAG GACGTAGGCT AGAGTTTCGA AAACGATTAG
AGACCGGAGT
R3              GGGCGAATCC GATGATCAAG GACGTAGGCT AGAGTTTCGA AAACGATTAG
AGACCGGAGT
R4              GGGCGAATCC GATGATCAAG GACGTAGGCT AGAGTTTCGA AAACGATTAG
AGACCGGAGT
R5              GGGCGAATCC GATGATCAAG GACGTAGGCT AGAGTTTCGA AAACGATTAG
AGACCGGAGT
R6              GGGCGAATCC GATGATCAAG GACGTAGGCT AGAGTTTCGA AAACGATTAG
AGACCGGAGT
R7              GGGCGAATCC GATGATCAAG GACGTAGGCT AGAGTTTCGA AAACGATTAG
AGACCGGAGT
N1              GGGCGAATCC GATGATCAAG GACGTAGGCT AGAGTTTCGA AAACGATTAG
AGACCGGAGT
N2              GGGCGAATCC GATGATCAAG GACGTAGGCT AGAGTTTCGA AAACGATTAG
AGACCGGAGT
N3              GGGCGAATCC GATGATCAAG GACGTAGGCT AGAGTTTCGA AAACGATTAG
AGACCGGAGT
M1              GGGCGAATCC GATGATCAAG GACGTAGGCT AGAGTTTCGA AAACGATTAG
AGACCGGAGT
M2              GGGCGAATCC GATGATCAAG GACGTAGGCT AGAGTTTCGA AAACGATTAG
AGACCGGAGT
M3              GGGCGAATCC GATGATCAAG GACGTAGGCT AGAGTTTCGA AAACGATTAG
AGACCGGAGT
M4              GGGCGAATCC GATGATCAAG GACGTAGGCT AGAGTTTCGA AAACGATTAG
AGACCGGAGT
M5              GGGCGAATCC GATGATCAAG GACGTAGGCT AGAGTTTCGA AAACGATTAG
AGACCGGAGT
M6              GGGCGAATCC GATGATCAAG GACGTAGGCT AGAGTTTCGA AAACGATTAG
AGACCGGAGT
M7              GGGCGAATCC GATGATCAAG GACGTAGGCT AGAGTTTCGA AAACGATTAG
AGACCGGAGT
```

M8 GGGCGAATCC GATGATCAAG GACGTAGGCT AGAGTTTCGA AAACGATTAG
AGACCGGAGT
M9 GGGCGAATCC GATGATCAAG GACGTAGGCT AGAGTTTCGA AAACGATTAG
AGACCGGAGT
M10 GGGCGAATCC GATGATCAAG GACGTAGGCT AGAGTTTCGA AAACGATTAG
AGACCGGAGT
M11 GGGCGAATCC GATGATCAAG GACGTAGGCT AGAGTTTCGA AAACGATTAG
AGACCGGAGT
M12 GGGCGAATCC GATGATCAAG GACGTAGGCT AGAGTTTCGA AAACGATTAG
AGACCGGAGT
M13 GGGCGAATCC GATGATCAAG GACGTAGGCT AGAGTTTCGA AAACGATTAG
AGACCGGAGT
M14 GGGCGAATCC GATGATCAAG GACGTAGGCT AGAGTTTCGA AAACGATTAG
AGACCGGAGT
M15 GGGCGAATCC GATGATCAAG GACGTAGGCT AGAGTTTCGA AAACGATTAG
AGACCGGAGT
M16 GGGCGAATCC GATGATCAAG GACGTAGGCT AGAGTTTCGA AAACGATTAG
AGACCGGAGT
M17 GGGCGAATCC GATGATCAAG GACGTAGGCT AGAGTTTTGA AAACGATTAG
AGACCGGAGT
M18 GGGCGAATCC GATGATCAAG GACGTAGGCT AGAGTTTCGA AAACGATTAG
AGACCGGAGT
M19 GGGCGAATCC GATGATCAAG GACGTAGGCT AGAGTTTCGA AAACGATTAG
AGACCGGAGT
M20 GGGCGAATCC GATGATCAAG GACGTAGGCT AGAGTTTCGA AAACGATTAG
AGACCGGAGT
M21 GGGCGAATCC GATGATCAAG GACGTAGGCT AGAGTTTCGA AAACGATTAG
AGACCGGAGT
M22 GGGCGAATCC GATGATCAAG GACGTAGGCT AGAGTTTCGA AAACGATTAG
AGACCGGAGT
M23 GGGCGAATCC GATGATCAAG GACGTAGGCT AGAGTTTCGA AAACGATTAG
AGACCGGAGT
B1 GGGCGAATCC GATGATCAAG GACGTAGGCT AGAGTTTCGA AAACGATTAG
AGACCGGAGT
B2 GGGCGAATCC GATGATCAAG GACGTAGGCT AGAGTTTCGA AAACGATTAG
AGACCGGAGT
B3 GGGCGAATCC GATGATCAAG GACGTAGGCT AGAGTTTCGA AAACGATTAG
AGACCGGAGT
B4 GGGCGAATCC GATGATCAAG GACGTAGGCT AGAGTTTCGA AAACGATTAG
AGACCGGAGT
B5 GGGCGAATCC GATGATCAAG GACGTAGGCT AGAGTTTCGA AAACGATTAG
AGACCGGAGT
B6 GGGCGAATCC GATGATCAAG GACGTAGGCT AGAGTTTCGA AAACGATTAG
AGACCGGAGT
B7 GGGCGAATCC GATGATCAAG GACGTAGGCT AGAGTTTCGA AAACGATTAG
AGACCGGAGT
B8 GGGCGAATCC GATGATCAAG GACGTAGGCT AGAGTTTCGA AAACGATTAG
AGACCGGAGT
B9 GGGCGAATCC GATGATCAAG GACGTAGGCT AGAGTTTCGA AAACGATTAG
AGACCGGAGT
B10 GGGCGAATCC GATGATCAAG GACGTAGGCT AGAGTTTCGA AAACGATTAG
AGACCGGAGT
B11 GGGCGAATCC GATGATCAAG GACGTAGGCT AGAGTTTCGA AAACGATTAG
AGACCGGAGT
B12 GGGCGAATCC GATGATCAAG GACGTAGGCT AGAGTTTCGA AAACGATTAG
AGACCGGAGT
B13 GGGCGAATCC GATGATCAAG GACGTAGGCT AGAGTTTCGA AAACGATTAG
AGACCGGAGT

B14 GGGCGAATCC GATGATCAAG GACGTAGGCT AGAGTTTCGA AAACGATTAG
AGACCGGAGT
B15 GGGCGAATCC GATGATCAAG GACGTAGGCT AGAGTTTCGA AAACGATTAG
AGACCGGAGT
B16 GGGCGAATCC GATGATCAAG GACGTAGGCT AGAGTTTCGA AAACGATTAG
AGACCGGAGT
KF537632_Ddi GGGCGAATCC GATGATCAAG GACGTAGGCT AGAGGTTCGA AAACGATTAG
AGACCGGAGT
AJ438961_Dde GGGCGAATCC GATGATCAAG GACGTAGGCT AGAGGTTCGA AAACGATTAG
AGACCGGAGT
AJ438960_Dca GGGCGAATCC GATGATCAAG GACGTAGGCT AGAGTTTCGA AAACGATTAG
AGACCGGAGT
AJ438959_Dca GGGCGAATCC GATGATCAAG GACGTAGGCT AGAGTTTCGA AAACGATTAG
AGACCGGAGT
AJ438958_Dga GGGCGAATCC GATGATCAAG GACGTAGGCT AGAGTATCGA AAACGATTAG
AGACCGGAGT

....|....| ....|....| ....|....| ....|....| ....|....|
....|....|
665 675 685 695 705
715
D1 AGTTCTAGCA GTAAACTATG CCGACGCCGT GGTATC--AA CAA------T
GGTATTGCGG
D2 AGTTCTAGCA GTAAACTATG CCGACGCCGT GGTATC--AA CAA------T
GGTATTGCGG
D3 AGTTCTAGCA GTAAACTATG CCGACGCCGT GGTATC--AA CAA------T
GGTATTGCGG
R1 AGTTCTAGCA GTAAACTATG CCGACGCCGT GGTAT----T TTT------T
AGTATTGCGG
R2 AGTTCTAGCA GTAAACTATG CCGACGCCGT GGTATT-AGG TAT------T
AGTATTGCGG
R3 AGTTCTAGCA GTAAACTATG CCGACGCCGT GGTATT-AGG TAT------T
AGTATTGCGG
R4 AGTTCTAGCA GTAAACTATG CCGACGCCGT GGTATA--GG CTA------T
AGTATTGCGG
R5 AGTTCTAGCA GTAAACTATG CCGACACCGT GGTATTAATT TTT------T
AGTATTGCGG
R6 AGTTCTAGCA GTAAACTATG CCGACACCGT GGTATT--AA TTT------T
AGTATTGCGG
R7 AGTTCTAGCA GTAAACTATG CCGACACCGT GGTATT--AA TTT------T
AGTATTGCGG
N1 AGTTCTAGCA GTAAACTATG CCGACGCCGT GGTATG--TA TTT------A
TGTATTGCGG
N2 AGTTCTAGCA GTAAACTATG CCGACGCCGT GGTATG--TA TTT------A
TGTATTGCGG
N3 AGTTCTAGCA GTAAACTATG CCGACGCCGT GGTATG--TA TTT------A
TGTATTGCGG
M1 AGTTCTAGCA GTAAACTATG CCGACGCCGT GATATT--GT TTTTTGTGGC
AGTATTGCGG
M2 AGTTCTAGCA GTAAACTATG CCGACGCCGT GATATT--GT TTTTTGTGGC
AGTATTGCGG
M3 AGTTCTAGCA GTAAACTATG CCGACGCCGT GATATT--GT TTTTTGTGGC
AGTATTGCGG
M4 AGTTCTAGCA GTAAACTATG CCGACGCCGT GATATT--GT TTTTTGTGGC
AGTATTGCGG
M5 AGTTCTAGCA GTAAACTATG CCGACGCCGT GATATT--GT TTTTTGTGGC
AGTATTGCGG

M6 AGTTCTAGCA GTAAACTATG CCGACGCCGT GATATT--GT TTTTTGTGGC
AGTATTGCGG
M7 AGTTCTAGCA GTAAACTATG CCGACGCCGT GATATT--GT TTTTTGTGGC
AGTATTGCGG
M8 AGTTCTAGCA GTAAACTATG CCGACGCCGT GATATT--GT TTTTTGTGGC
AGTATTGCGG
M9 AGTTCTAGCA GTAAACTATG CCGACGCCGT GATATT--GT TTTTTGTGGC
AGTATTGCGG
M10 AGTTCTAGCA GTAAACTATG CCGACGCCGT GATATT--GT TTTTTGTGGC
AGTATTGCGG
M11 AGTTCTAGCA GTAAACTATG CCGACGCCGT GATATT--GT TTTTTGTGGC
GGTATTGCGG
M12 AGTTCTAGCA GTAAACTATG CCGACGCCGT GATATT--GT TTTTTGTGGC
GGTATTGCGG
M13 AGTTCTAGCA GTAAACTATG CCGACGCCGT GATATT--GT TTTTTGTGGC
GGTATTGCGG
M14 AGTTCTAGCA GTAAACTATG CCGACGCCGT GGTATG--GT TTTTTGTGGC
TGTATTGCGG
M15 AGTTCTAGCA GTAAACTATG CCGACGCCGT GGTATG--GT TTTTTGTGGC
TGTATTGCGG
M16 AGTTCTAGCA GTAAAATATG CCGACGCCGT GGTATG--GT TTTTTGTGGC
TGTATTGCGG
M17 AGTTCTAGCA GTAAACTATG CCGACGCCGT GGTATG--GT TTTTTGTGGC
TGTATTGCGG
M18 AGTTCTAGCA GTAAACTATG CCGACGCCGT GATATA--GT TTTTTGTGGC
TGTATTGCGG
M19 AGTTCTAGCA GTAAACTATG CCGACGCCGT GATATA--GT TT------GT
GGTATTGCGG
M20 AGTTCTAGCA GTAAACTATG CCGACGCCGT GATATT--GT TTTTTGTGGC
GGTATTGCGG
M21 AGTTCTAGCA GTAAACTATG CCGACGCCGT GATATT--GT TTTTTGTGGC
GGTATTGCGG
M22 AGTTCTAGCA GTAAACTATG CCGACGCCGT GATATT--GT TTTTTGTGGC
GGTATTGCGG
M23 AGTTCTAGCA GTAAACTATG CCGACGCCGT GATATT--GT TTTTTGTGGC
GGTATTGCGG
B1 AGTTCTAGCA GTAAACTATG CCGACGCCGT GGTATG--GT ATT------C
TGTATTGCGG
B2 AGTTCTAGCA GTAAACTATG CCGACGCCGT GGTATG--GT ATT------C
TGTATTGCGG
B3 AGTTCTAGCA GTAAACTATG CCGACGCCGT GGTATG--GT ATT------C
TGTATTGCGG
B4 AGTTCTAGCA GTAAACTATG CCGACGCCGT GGTATG--GT ATT------C
TGTATTGCGG
B5 AGTTCTAGCA GTAAACTATG CCGACGCCGT GGTATG--GT ATT------C
TGTATTGCGG
B6 AGTTCTAGCA GTAAACTATG CCGACGCCGT GGTATG--GT ATT------C
TGTATTGCGG
B7 AGTTCTAGCA GTAAACTATG CCGACGCCGT GGTATG--GT ATT------C
TGTATTGCGG
B8 AGTTCTAGCA GTAAACTATG CCGACGCCGT GGTATG--GT ATT------C
TGTATTGCGG
B9 AGTTCTAGCA GTAAACTATG CCGACGCCGT GGTATG--GT ATT------C
TGTATTGCGG
B10 AGTTCTAGCA GTAAACTATG CCGACGCCGT GGTATG--GT ATT------C
TGTATTGCGG
B11 AGTTCTAGCA GTAAACTATG CCGACGCCGT GGTATG--GT ATT------C
TGTATTGCGG

B12 AGTTCTAGCA GTAAACTATG CCGACGCCGT GGTATG--GT ATT------C
TGTATTGCGG
B13 AGTTCTAGCA GTAAACTATG CCGACGCCGT GGTATG--GT ATT------C
TGTATTGCGG
B14 AGTTCTAGCA GTAAACTATG CCGACGCCGT GGTATG--GT ATT------C
TGTATTGCGG
B15 AGTTCTAGCA GTAAACTATG CCGACGCCGT GATATG--GT ATT------C
TGTATTGCGG
B16 AGTTCTAGCA GTAAACTATG CCGACGCCGT GGTATG--GT ATT------C
TGTATTGCGG
KF537632_Ddi AGTTCTAGCA GTAAACTATG CCGACGCCGT GGTATG--TT TTTT----AA
TGTATTGCGG
AJ438961_Dde AGTTCTAGCA GTAAACTATG CCGACGCCGC GATATG-AGG TTT------T
TGTATTGCGG
AJ438960_Dca AGTTCTAGCA GTAAACTATG CCGACGCCGT GATATG--AT TTT-----GT
TGTATTGCGG
AJ438959_Dca AGTTCTAGCA GTAAACTATG CCGACGCCGT GATATG--AT TTT-----GT
TGTATTGCGG
AJ438958_Dga AGTTCTAGCA GTAAACTATG CCGACTTTGT GGCAGA--GG TAT------C
TGTTGCAAAC

....|....| ....|....| ....|....| ....|....| ....|....|
....|....|
725 735 745 755 765
775
D1 AAGAGAAATC AAGT-AAGGC TTTGGGGAGA GTACGCGCGC AAGCGATAAA
TTTAAAGGAA
D2 AAGAGAAATC AAGT-AAGGC TTTGGGGAGA GTACGCGCGC AAGCGATAAA
TTTAAAGGAA
D3 AAGAGAAATC AAGT-AAGGC TTTGGGGAGA GTACGCGCGC AAGCGATAAA
TTTAAAGGAA
R1 AAGAGAAATC AAGT-AAGGC TTTGGGGAGA GTACGCGCGC AAGCGATAAA
TTTAAAGGAA
R2 AAGAGAAATC AAGTAAAGGC TTTGGGGAGA GTACGCGCGC AAGCGATAAA
TTTAAAGGAA
R3 AAGAGAAATC AAGT-AAGGC TTTGGGGAGA GTACGCGCGC AAGCGATAAA
TTTAAAGGAA
R4 AAGAGAAATC AAGT-AAGGC TTTGGGGAGA GTACGCGCGC AAGCGATAAA
TTTAAAGGAA
R5 AAGAGAAATC AAGT-AAGGC TTTGGGGAGA GTACGCGCGC AAGCGATAAA
TTTAAAGGAA
R6 AAGAGAAATC AAGT-AAGGC TTTGGGGAGA GTACGCGCGC AAGCGATAAA
TTTAAAGGAA
R7 AAGAGAAATC AAGT-AAGGC TTTGGGGAGA GTACGCGCGC AAGCGATAAA
TTTAAAGGAA
N1 AAGAGAAATC AAGT-AAGGC TTTGGGGAGA GTACGCGCGC AAGCGATAAA
TTTAAAGGAA
N2 AAGAGAAATC AAGT-AAGGC TTTGGGGAGA GTACGCGCGC AAGCGATAAA
TTTAAAGGAA
N3 AAGAGAAATC AAGT-AAGGC TTTGGGGAGA GTACGCGCGC AAGCGATAAA
TTTAAAGGAA
M1 AAGAGAAATC AAGT-AAGGC TTTGGGGAGA GTACGCGCGC AAGCGATAAA
TTTAAAGGAA
M2 AAGAGAAATC AAGT-AAGGC TTTGGGGAGA GTACGCGCGC AAGCGATAAA
TTTAAAGGAA
M3 AAGAGAAATC AAGT-AAGGC TTTGGGGAGA GTACGCGCGC AAGCGATAAA
TTTAAAGGAA

M4 AAGAGAAATC AAGT-AAGGC TTTGGGGAGA GTACGCGCGC AAGCGATAAA
TTTAAAGGAA
M5 AAGAGAAATC AAGT-AAGGC TTTGGGGAGA GTACGCGCGC AAGCGATAAA
TTTAAAGGAA
M6 AAGAGAAATC AAGT-AAGGC TTTGGGGAGA GTACGCGCGC AAGCGATAAA
TTTAAAGGAA
M7 AAGAGAAATC AAGT-AAGGC TTTGGGGAGA GTACGCGCGC AAGCGATAAA
TTTAAAGGAA
M8 AAGAGAAATC AAGT-AAGGC TTTGGGGAGA GTACGCGCGC AAGCGATAAA
TTTAAAGGAA
M9 AAGAGAAATC AAGT-AAAGC TTTGGGGAGA GTACGCGCGC AAGCGATAAA
TTTAAAGGAA
M10 AAGAGAAATC AAGT-AAGGC TTTGGGGAGA GTACGCGCGC AAGCGATAAA
TTTAAAGGAA
M11 AAGAGAAATC AAGT-AAAGC TTTGGGGAGA GTACGCGCGC AAGCGATAAA
TTTAAAGGAA
M12 AAGAGAAATC AAGT-AAGGC TTTGGGGAGA GTACGCGCGC AAGCGATAAA
TTTAAAGGAA
M13 AAGAGAAATC AAGT-AAGGC TTTGGGGAGA GTACGCGCGC AAGCGATAAA
TTTAAAGGAA
M14 AAGAGAAATC AAGT-AAGGC TTTGGGGAGA GTACGCGCGC AAGCGATAAA
TTTAAAGGAA
M15 AAGAGAAATC AAGT-AAGGC TTTGGGGAGA GTACGCGCGC AAGCGATAAA
TTTAAAGGAA
M16 AAGAGAAATC AAGT-AAGGC TTTGGGGAGA GTACGCGCGC AAGCGATAAA
TTTAAAGGAA
M17 AAGAGAAATC AAGT-AAGGC TTTGGGGAGA GTACGCGCGC AAGCGATAAA
TTTAAAGGAA
M18 AAGAGAAATC AAGT-AAGGC TTTGGGGAGA GTACGCGCGC AAGCGATAAA
TTTAAAGGAA
M19 AAGAGAAATC AAGT-AAGGC TTTGGGGAGA GTACGCGCGC AAGCGATAAA
TTTAAAGGAA
M20 AAGAGAAATC AAGT-AAGGC TTTGGGGAGA GTACGCGCGC AAGCGATAAA
TTTAAAGGAA
M21 AAGAGAAATC AAGT-AAAGC TTTGGGGAGA GTACGCGCGC AAGCGATAAA
TTTAAAGGAA
M22 AAGAGAAATC AAGT-AAGGC TTTGGGGAGA GTACGCGCGC AAGCGATAAA
TTTAAAGGAA
M23 AAGAGAAATC AAGT-AAGGC TTTGGGGAGA GTACGCGCGC AAGCGATAAA
TTTAAAGGAA
B1 AAGAGAAATC AAGT-AAGGC TTTGGGGAGA GTACGCGCGC AAGCGATAAA
TTTAAAGGAA
B2 AAGAGAAATC AAGT-AAGGC TTTGGGGAGA GTACGCGCGC AAGCGATAAA
TTTAAAGGAA
B3 AAGAGAAATC AAGT-AAGGC TTTGGGGAGA GTACGCGCGC AAGCGATAAA
TTTAAAGGAA
B4 AAGAGAAATC AAGT-AAGGC TTTGGGGAGA GTACGCGCGC AAGCGATAAA
TTTAAAGGAA
B5 AAGAGAAATC AAGT-AAGGC TTTGGGGAGA GTACGCGCGC AAGCGATAAA
TTTAAAGGAA
B6 AAGAGAAATC AAGT-AAGGC TTTGGGGAGA GTACGCGCGC AAGCGATAAA
TTTAAAGGAA
B7 AAGAGAAATC AAGT-AAGGC TTTGGGGAGA GTACGCGCGC AAGCGATAAA
TTTAAAGGAA
B8 AAGAGAAATC AAGT-AAGGC TTTGGGGAGA GTACGCGCGC AAGCGATAAA
TTTAAAGGAA
B9 AAGAGAAATC AAGT-AAGGC TTTGGGGAGA GTACGCGCGC AAGCGATAAA
TTTAAAGGAA

B10 AAGAGAAATC AAGT-AAGGC TTTGGGGAGA GTACGCGCGC AAGCGATAAA
TTTAAAGGAA
B11 AAGAGAAATC AAGT-AAGGC TTTGGGGAGA GTACGCGCGC AAGCGATAAA
TTTAAAGGAA
B12 AAGAGAAATC AAGT-AAGGC TTTGGGGAGA GTACGCGCGC AAGCGATAAA
TTTAAAGGAA
B13 AAGAGAAATC AAGT-AAGGC TTTGGGGAGA GTACGCGCGC AAGCGATAAA
TTTAAAGGAA
B14 AAGAGAAATC AAGT-AAGGC TTTGGGGAGA GTACGCGCGC AAGCGATAAA
TTTAAAGGAA
B15 AAGAGAAATC AAGT-AAGGC TTTGGGGAGA GTACGCGCGC AAGCGATAAA
TTTAAAGGAA
B16 AAGAGAAATC AAGT-AAGGC TTTGGGGAGA GTACGCGCGC AAGCGATAAA
TTTAAAGGAA
KF537632_Ddi AAGAGAAATC AAGT-AAGGC TTTGGGGAGA GTACGCGCGC AAGCGATAAA
TTTAAAGGAA
AJ438961_Dde AAGAGAAATC AAGT-AAGGC TTTGGGGAGA GTACGCGCGC AAGCGATAAA
TTTAAAGGAA
AJ438960_Dca AAGAGAAATC AAGT-AAGGC TTTGGGGAGA GTACGCGCGC AAGCGATAAA
TTTAAAGGAA
AJ438959_Dca AAGAGAAATC AAGT-AAGGC TTTGGGGAGA GTACGCGCGC AAGCGATAAA
TTTAAAGGAA
AJ438958_Dga GAGAAATCTT AAGT-AAGGC TTTGGGGAGA GTACGCGCGC AAGCGATAAA
TTTAAAGGAA

....|....| ....|....| ....|....| ....|....| ....|....|
....|....|
785 795 805 815 825
835
D1 ATTGACGGAG GAACACCACA AGGAGTGGAG TGTGCGGCTT AATTTGACTC
AACGCGGGAC
D2 ATTGACGGAG GAACACCACA AGGAGTGGAG TGTGCGGCTT AATTTGACTC
AACGCGGGAC
D3 ATTGACGGAG GAACACCACA AGGAGTGGAG TGTGCGGCTT AATTTGACTC
AACGCGGGAC
R1 ATTGACGGAG GAACACCACA AGGAGTGGAG TGTGCGGCTT AATTTGACTC
AACGCGGGAC
R2 ATTGACGGAG GAACACCACA AGGAGTGGAG TGTGCGGCTT AATTTGACTC
AACGCGGGAC
R3 ATTGACGGAG GAACACCACA AGGAGTGGAG TGTGCGGCTT AATTTGACTC
AACGCGGGAC
R4 ATTGACGGAG GAACACCACA AGGAGTGGAG TGTGCGGCTT AATTTGACTC
AACGCGGGAC
R5 ATTGACGGAG GAACACCACA AGGAGTGGAG TGTGCGGCTT AATTTGACTC
AACGCGGGAC
R6 ATTGACGGAG GAACACCACA AGGAGTGGAG TGTGCGGCTT AATTTGACTC
AACGCGGGAC
R7 ATTGACGGAG GAACACCACA AGGAGTGGAG TGTGCGGCTT AATTTGACTC
AACGCGGGAC
N1 ATTGACGGAG GAACACCACA AGGAGTGGAG TGTGCGGCTT AATTTGACTC
AACGCGGGAC
N2 ATTGACGGAG GAACACCACA AGGAGTGGAG TGTGCGGCTT AATTTGACTC
AACGCGGGAC
N3 ATTGACGGAG GAACACCACA AGGAGTGGAG TGTGCGGCTT AATTTGACTC
AACGCGGGAC
M1 ATTGACGGAG GAACACCACA AGGAGTGGAG TGTGCGGCTT AATTTGACTC
AACGCGGGAC

M2 ATTGACGGAG GAACACCACA AGGAGTGGAG TGTGCGGCTT AATTTGACTC
AACGCGGGAC
M3 ATTGACGGAG GAACACCACA AGGAGTGGAG TGTGCGGCTT AATTTGACTC
AACGCGGGAC
M4 ATTGACGGAG GAACACCACA AGGAGTGGAG TGTGCGGCTT AATTTGACTC
AACGCGGGAC
M5 ATTGACGGAG GAACACCACA AGGAGTGGAG TGTGCGGCTT AATTTGACTC
AACGCGGGAC
M6 ATTGACGGAG GAACACCACA AGGAGTGGAG TGTGCGGCTT AATTTGACTC
AACGCGGGAC
M7 ATTGACGGAG GAACACCACA AGGAGTGGAG TGTGCGGCTT AATTTGACTC
AACGCGGGAC
M8 ATTGACGGAG GAACACCACA AGGAGTGGAG TGTGCGGCTT AATTTGACTC
AACGCGGGAC
M9 ATTGACGGAG GAACACCACA AGGAGTGGAG TGTGCGGCTT AATTTGACTC
AACGCGGGAC
M10 ATTGACGGAG GAACACCACA AGGAGTGGAG TGTGCGGCTT AATTTGACTC
AACGCGGGAC
M11 ATTGACGGAG GAACACCACA AGGAGTGGAG TGTGCGGCTT AATTTGACTC
AACGCGGGAC
M12 ATTGACGGAG GAACACCACA AGGAGTGGAG TGTGCGGCTT AATTTGACTC
AACGCGGGAC
M13 ATTGACGGAG GAACACCACA AGGAGTGGAG TGTGCGGCTT AATTTGACTC
AACGCGGGAC
M14 ATTGACGGAG GAACACCACA AGGAGTGGAG TGTGCGGCTT AATTTGACTC
AACGCGGGAC
M15 ATTGACGGAG GAACACCACA AGGAGTGGAG TGTGCGGCTT AATTTGACTC
AACGCGGGAC
M16 ATTGACGGAG GAACACCACA AGGAGTGGAG TGTGCGGCTT AATTTGACTC
AACGCGGGAC
M17 ATTGACGGAG GAACACCACA AGGAGTGGAG TGTGCGGCTT AATTTGACTC
AACGCGGGAC
M18 ATTGACGGAG GAACACCACA AGGAGTGGAG TGTGCGGCTT AATTTGACTC
AACGCGGGAC
M19 ATTGACGGAG GAACACCACA AGGAGTGGAG TGTGCGGCTT AATTTGACTC
AACGCGGGAC
M20 ATTGACGGAG GAACACCACA AGGAGTGGAG TGTGCGGCTT AATTTGACTC
AACGCGGGAC
M21 ATTGACGGAG G-ACACCACA AGGAGTGGAG TGTGCGGCTT AATTTGACTC
AACGCGGGAC
M22 ATTGACGGAG GAACACCACA AGGAGTGGAG TGTGCGGCTT AATTTGACTC
AACGCGGGAC
M23 ATTGACGGAG GAACACCACA AGGAGTGGAG TGTGCGGCTT AATTTGACTC
AACGCGGGAC
B1 ATTGACGGAG GAACACCACA AGGAGTGGAG TGTGCGGCTT AATTTGACTC
AACGCGGGAC
B2 ATTGACGGAG GAACACCACA AGGAGTGGAG TGTGCGGCTT AATTTGACTC
AACGCGGGAC
B3 ATTGACGGAG GAACACCACA AGGAGTGGAG TGTGCGGCTT AATTTGACTC
AACGCGGGAC
B4 ATTGACGGAG GAACACCACA AGGAGTGGAG TGTGCGGCTT AATTTGACTC
AACGCGGGAC
B5 ATTGACGGAG GAACACCACA AGGAGTGGAG TGTGCGGCTT AATTTGACTC
AACGCGGGAC
B6 ATTGACGGAG GAACACCACA AGGAGTGGAG TGTGCGGCTT AATTTGACTC
AACGCGGGAC
B7 ATTGACGGAG GAACACCACA AGGAGTGGAG TGTGCGGCTT AATTTGACTC
AACGCGGGAC

```
B8            ATTGACGGAG GAACACCACA AGGAGTGGAG TGTGCGGCTT AATTTGACTC
AACGCGGGAC
B9            ATTGACGGAG GAACACCACA AGGAGTGGAG TGTGCGGCTT AATTTGACTC
AACGCGGGAC
B10           ATTGACGGAG GAACACCACA AGGAGTGGAG TGTGCGGCTT AATTTGACTC
AACGCGGGAC
B11           ATTGACGGAG GAACACCACA AGGAGTGGAG TGTGCGGCTT AATTTGACTC
AACGCGGGAC
B12           ATTGACGGAG GAACACCACA AGGAGTGGAG TGTGCGGCTT AATTTGACTC
AACGCGGGAC
B13           ATTGACGGAG GAACACCACA AGGAGTGGAG TGTGCGGCTT AATTTGACTC
AACGCGGGAC
B14           ATTGACGGAG GAACACCACA AGGAGTGGAG TGTGCGGCTT AATTTGACTC
AACGCGGGAC
B15           ATTGACGGAG GAACACCACA AGGAGTGGAG TGTGCGGCTT AATTTGACTC
AACGCGGGAC
B16           ATTGACGGAG GAACACCACA AGGAGTGGAG TGTGCGGCTT AATTTGACTC
AACGCGGGAC
KF537632_Ddi  ATTGACGGAG GAACACCACA AGGAGTGGAG TGTGTGGCTT AATTTGACTC
AACGCGGGAC
AJ438961_Dde  ATTGACGGAG GAACACCACA AGGAGTGGAG TGTGCGGCTT AATTTGACTC
AACGCGGGAC
AJ438960_Dca  ATTGACGGAG GAACACCACA AGGAGTGGAG TGTGCGGCTT AATTTGACTC
AACGCGGGAC
AJ438959_Dca  ATTGACGGAG GAACACCACA AGGAGTGGAG TGTGCGGCTT AATTTGACTC
AACGCGGGAC
AJ438958_Dga  ATTGACGGAG GAACACCACA AGGAGTAGAG TGTGCGGCTT AATTTGACTC
AACGCGGGAC

              ....|....| ....|....| ....|....| ....|....| ....|....|
....|....|
                   845        855        865        875        885
895
D1            AGCTTACCAG GCCCGATAAT CGAGCGAGCG TAGTACGCGA TAGGTTAAAG
AGTGGTGCAT
D2            AGCTTACCAG GCCCGATAAT CGAGCGAGCG TAGTACGCGA TAGGTTAAAG
AGTGGTGCAT
D3            AGCTTACCAG GCCCGATAAT CGAGCGAGCG TAGTACGCGA TAGGTTAAAG
AGTGGTGCAT
R1            AGCTTACCAG GCCCGATAAT CGAGCGAGCG TAGTACGCGA TAGGTTAAAA
AGTGGTGCAT
R2            AGCTTACCAG GCCCGATAAT CGAGCGAGCG TAGTACGCGA TAGGTTAAGA
AGTGGTGCAT
R3            AGCTTACCAG GCCCGAT-AT CGAGCGAGCG TAGTACGCGA TAGGTTAAGA
AGTGGTGCAT
R4            AGCTTACCAG GCCCGATAAT CGAGCGAGCG TAGTACGCGA TAGGTTAAAA
AGTGGTGCAT
R5            AGCTTACCAG GCCCGATAAT CGAGCGAGCG TAGTACGCGA TAGATTAAAA
AGTGGTGCAT
R6            AGCTTACCAG GCCCGATAAT CGAGCGAGCG TAGTACGCGA TAGGTTAAAA
AGTGGTGCAT
R7            AGCTTACCAG GCCCGATAAT CGAGCGAGCG TAGTACGCGA TAGGTTAAAA
AGTGGTGCAT
N1            AGCTTACCAG GCCCGATAAT CGAGCGAGCG TAGTACGCGA TAGGTTAAAG
AGTGGTGCAT
N2            AGCTTACCAG GCCCGATAAT CGAGCGAGCG TAGTACGCGA TAGGTTAAAG
AGTGGTGCAT
```

N3 AGCTTACCAG GCCCGATAAT CGAGCGAGCG TAGTACGCGA TAGGTTAAAG AGTGGTGCAT
M1 AGCTTACCAG GCCCGATAAT CGAGCGAGCG TAGTACGCGA TAGATTAAAG AGTGGTGCAT
M2 AGCTTACCAG GCCCGATAAT CGAGCGAGCG TAGTACGCGA TAGATTAAAG AGTGGTGCAT
M3 AGCTTACCAG GCCCGATAAT CGAGCGAGCG TAGTACGCGA TAGATTAAAG AGTGGTGCAT
M4 AGCTTACCAG GCCCGATAAT CGAGCGAGCG TAGTACGCGA TAGATTAAAG AGTGGTGCAT
M5 AGCTTACCAG GCCCGATAAT CGAGCGAGCG TAGTACGCGA TAGATTAAAG AGTGGTGCAT
M6 AGCTTACCAG GCCCGATAAT CGAGCGAGCG TAGTACGCGA TAGATTAAAG AGTGGTGCAT
M7 AGCTTACCAG GCCCGATAAT CGAGCGAGCG TAGTACGCGA TAGATTAAAG AGTGGTGCAT
M8 AGCTTACCAG GCCCGATAAT CGAGCGAGCG TAGTACGCGA TAGATTAAAG AGTGGTGCAT
M9 AGCTTACCAG GCCCGATAAT CGAGCGAGCG TAGTACGCGA TAGATTAAAG AGTGGTGCAT
M10 AGCTTACCAG GCCCGATAAT CGAGCGAGCG TAGTACGCGA TAGATTAAAG AGTGGTGCAT
M11 AGCTTACCAG GCCCGATAAT CGAGCGAGCG TAGTACGCGA TAGATTAAAG AGTGGTGCAT
M12 AGCTTACCAG GCCCGATAAT CGAGCGAGCG TAGTACGCGA TAGATTAAAG AGTGGTGCAT
M13 AGCTTACCAG GCCCGATAAT CGAGCGAGCG TAGTACGCGA TAGATTAAAG AGTGGTGCAT
M14 AGCTTACCAG GCCCGATAAT CGAGCGAGCG TTGTACGCGA TAGATTAAAG AGTGGTGCAT
M15 AGCTTACCAG GCCCGATAAT CGAGCGAGCG TTGTACGCGA TAGATTAAAG AGTGGTGCAT
M16 AGCTTACCAG GCCCGATAAT CGAGCGAGCG TTGTACGCGA TAGATTAAAG AGTGGTGCAT
M17 AGCTTACCAG GCCCGATAAT CGAGCGAGCG TTGTACGCGA TAGATTAAAG AGTGGTGCAT
M18 AGCTTACCAG GCCCGATAAT CGAGCGAGCG TTGTACGCGA TAGATTAAAG AGTGGTGCAT
M19 AGCTTACCAG GCCCGATAAT CGAGCGAGCG TTGTACGCGA TAGATTAAAG AGTGGTGCAT
M20 AGCTTACCAG GCCCGATAAT CGAGCGAGCG TAGTACGCGA TAGATTAAAG AGTGGTGCAT
M21 AGCTTACCA- GCCCGATAAT CGAGCGAGCG TAGTACGCGA TAGATTAAAG AGTGGTGCAT
M22 AGCTTACCAG GCCCGATAAT CGAGCGAGCG TAGTACGCGA TAGATTAAAG AGTGGTGCAT
M23 AGCTTACCAG GCCCGATAAT CGAGCGAGCG TAGTACGCGA TAGATTAAAG AGTGGTGCAT
B1 AGCTTACCAG GCCCGATAAT CATACGAGCG TAGTACGCGA TAGGTTAAAG AGTGGTGCAT
B2 AGCTTACCAG GCCCGATAAT CATACGAGCG TAGTACGCGA TAGGTTAGAG AGTGGTGCAT
B3 AGCTTACCAG GCCCGATAAT CATACGAGCG TAGTACGCGA TAGGTTAGAG AGTGGTGCAT
B4 AGCTTACCAG GCCCGATAAT CATACGAGCG TAGTACGCGA TAGGTTAGAG AGTGGTGCAT
B5 AGCTTACCAG GCCCGATAAT CATACGAGCG TAGTACGCGA TAGGTTAGAG AGTGGTGCAT

B6 AGCTTACCAG GCCCGATAAT CATACGAGCG TAGTACGCGA TAGGTTAGAG
AGTGGTGCAT
B7 AGCTTACCAG GCCCGATAAT CATACGAGCG TAGTACGCGA TAGGTTAGAG
AGTGGTGCAT
B8 AGCTTACCAG GCCCGATAAT CATACGAGCG TAGTACGCGA TAGGTTAGAG
AGTGGTGCAT
B9 AGCTTACCAG GCCCGATAAT CATACGAGCG TAGTACGCGA TAGGTTAGAG
AGTGGTGCAT
B10 AGCTTACCAG GCCCGATAAT CATACGAGCG TAGTACGCGA TAGGTTAGAG
AGTGGTGCAT
B11 AGCTTACCAG GCCCGATAAT CATACGAGCG TAGTACGCGA TAGGTTAGAG
AGTGGTGCAT
B12 AGCTTACCAG GCCCGATAAT CATACGAGCG TAGTACGCGA TAGGTTAGAG
AGTGGTGCAT
B13 AGCTTACCAG GCCCGATAAT CATACGAGCG TAGTACGCGA TAGGTTAGAG
AGTGGTGCAT
B14 AGCTTACCAG GCCCGATAAT CATACGAGCG TAGTACGCGA TAGGTTAGAG
AGTGGTGCAT
B15 AGCTTACCAT GCCCGATAAT CATACGAGCG TAGTACGCGA TAGGTTAGAG
AGTGGTGCAT
B16 AGCTTACCAT GCCCGATAAT CATACGAGCG TAGTACGCGA TAGGTTAGAG
AGTGGTGCAT
KF537632_Ddi AGCTTACCAG GCCCGATAAT CGTACGAGCG TAGTACGCGA TAGGTTAAAG
AGTGGTGCAT
AJ438961_Dde AGCTTACCAG GCCCGATAAC TAAGCGAGCG TAGTACGCGA TAGGTTAGAG
AGTGGTGCAT
AJ438960_Dca AGCTTACCAG GCCCGATAAT CGTACGAGCG TAGTACGCGA TAGATTAGAG
AGTGGTGCAT
AJ438959_Dca AGCTTACCAG GCCCGATAAT CGTACGAGCG TAGTACGCGA TAGATTAGAG
AGTGGTGCAT
AJ438958_Dga AGCTTACCAG GCCCGATGAA CCGGCGAGCG TGATACGCGA TAGGTTTAGA
AGTGGTGCAT

....|....| ....|....| ....|....| ....|....| ....|....|
....|....|
905 915 925 935 945
955
D1 GGCTGCTATC GACAGTTGGG GTGACC-TTA GGGTTAATTC CG-GCAAGTA
GTGAGACCCC
D2 GGCTGCTATC GACAGTTGGG GTGACC-TTA GGGTTAATTC CG-GCAAGTA
GTGAGACCCC
D3 GGCTGCTATC GACAGTTGGG GTGACC-TTA GGGTTAATTC CG-GCAAGTA
GTGAGACCCC
R1 GGCTGCTATC GACAGTTGGG GTGACC-TTA GGGTTAATTC CG-GCAAGTA
GTGAGACCCC
R2 GGCTGCTATC GACAGTTGGG GTGACC-TTA GGGTTAATTC CG-GCAAGTA
GTGAGACCCC
R3 GGCTGCTATC GACAGTTGGG GTGACC-TTA GGGTTAATTC CG-GCAAGTA
GTGAGACCCC
R4 GGCTGCTATC GACAGTTGGG GTGACC-TTA GGGTTAATTC CG-GCAAGTA
GTGAGACCCC
R5 GGCTGCTATC GACAGTTGGG GTGACC-TTA GGGTTAATTC CG-GCAAGTA
GTGAGACCCC
R6 GGCTGCTATC GACAGTTGGG GTGACC-TTA GGGTTAATTC CG-GCAAGTA
GTGAGACCCC
R7 GGCTGCTATC GACAGTTGGG GTGACC-TTA GGGTTAATTC CG-GCAAGTA
GTGAGACCCC

N1 GGCTGCTATC GACAGTTGGG GTGACC-TTA GGGTTAATTC CG-GCAAGTA
GTGAGACCCC
N2 GGCTGCTATC GACAGTTGGG GTGACC-TTA GGGTTAATTC CG-GCAAGTA
GTGAGACCCC
N3 GGCTGCTATC GACAGTTGGG GTGACC-TTA GGGTTAATTC CG-GCAAGTA
GTGAGACCCC
M1 GGCTGCTATC GACAGTTGGG GTGACC-TTA GGGTTAATTC CG-GCAAGTA
GTGAGACCCC
M2 GGCTGCTATC GACAGTTGGG GTGACC-TTA GGGTTAATTC CG-GCAAGTA
GTGAGACCCC
M3 GGCTGCTATC GACAGTTGGG GTGACC-TTA GGGTTAATTC CG-GCAAGTA
GTGAGACCCC
M4 GGCTGCTATC GACAGTTGGG GTGACC-TTA GGGTTAATTC CG-GCAAGTA
GTGAGACCCC
M5 GGCTGCTATC GACAGTTGGG GTGACC-TTA GGGTTAATTC CG-GCAAGTA
GTGAGACCCC
M6 GGCTGCTATC GACAGTTGGG GTGACC-TTA GGGTTAATTC CG-GCAAGTA
GTGAGACCCC
M7 GGCTGCTATC GACAGTTGGG GTGACC-TTA GGGTTAATTC CG-GCAAGTA
GTGAGACCCC
M8 GGCTGCTATC GACAGTTGGG GTGACC-TTA GGGTTAATTC CG-GCAAGTA
GTGAGACCCC
M9 GGCTGCTATC GACAGTTGGG GTGACC-TTA GGGTTAATTC CG-GCAAGTA
GTGAGACCCC
M10 GGCTGCTATC GACAGTTGGG GTGACC-TTA GGGTTAATTC CG-GCAAGTA
GTGAGACCCC
M11 GGCTGCTATC GACAGTTGGG GTGACC-TTA GGGTTAATTC CG-GCAAGTA
GTGAGACCCC
M12 GGCTGCTATC GACAGTTGGG GTGACC-TTA GGGTTAATTC CG-GCAAGTA
GTGAGACCCC
M13 GGCTGCTATC GACAGTTGGG GTGACC-TTA GGGTTAATTC CG-GCAAGTA
GTGAGACCCC
M14 GGCTGCTATC GACAGTTGGG GTGACC-TTA GGGTTAATTC CG-GCAAGTA
GTGAGACCCC
M15 GGCTGCTATC GACAGTTGGG GTGACC-TTA GGGTTAATTC CG-GCAAGTA
GTGAGACCCC
M16 GGCTGCTATC GACAGTTGGG GTGACC-TTA GGGTTAATTC CG-GCAAGTA
GTGAGACCCC
M17 GGCTGCTATC GACAGTTGGG GTGACC-TTA GGGTTAATTC CG-GCAAGTA
GTGAGACCCC
M18 GGCTGCTATC GACAGTTGGG GTGACCTTTA GGGTTAATTC CG-GCAAGTA
GTGAGACCCC
M19 GGCTGCTATC GACAGTTGGG GTGACC-TTA GGGTTAATTC CG-GCAAGTA
GTGAGACCCC
M20 GGCTGCTATC GACAGTTGGG GTGACC-TTA GGGTTAATTC CG-GCAAGTA
GTGAGACCCC
M21 GGCTGCTATC GACAGTTGGG GTGACC-TTA GGGTTAATTC CG-GCAAGTA
GTGAGACCCC
M22 GGCTGCTATC GACAGTTGGG GTGACC-TTA GGGTTAATTC CG-GCAAGTA
GTGAGACCCC
M23 GGCTGCTATC GACAGTTGGG GTGACC-TTA GGGTTAATTC CG-GCAAGTA
GTGAGACCCC
B1 GGCTGCTATC GACAGTTGGG GTGACC-TTA GGGTTAATTC CG-GCAAGTA
GTGAGACCTC
B2 GGCTGCTATC GACAGTTGGG GTGACC-TTA GGGTTAATTC CG-GCAAGTA
GTGAGACCTC
B3 GGCTGCTATC GACAGTTGGG GTGACC-TTA GGGTTAATTC CG-GCAAGTA
GTGAGACCTC

```
B4             GGCTGCTATC GACAGTTGGG GTGACC-TTA GGGTTAATTC CG-GCAAGTA
GTGAGACCTC
B5             GGCTGCTATC GACAGTTGGG GTGACC-TTA GGGTTAATTC CG-GCAAGTA
GTGAGACCTC
B6             GGCTGCTATC GACAGTTGGG GTGACC-TTA GGGTTAATTC CG-GCAAGTA
GTGAGACCTC
B7             GGCTGCTATC GACAGTTGGG GTGACC-TTA GGGTTAATTC CG-GCAAGTA
GTGAGACCTC
B8             GGCTGCTATC GACAGTTGGG GTGACC-TTA GGGTTAATTC CG-GCAAGTA
GTGAGACCTC
B9             GGCTGCTATC GACAGTTGGG GTGACC-TTA GGGTTAATTC CG-GCAAGTA
CTGAGACCTC
B10            GGCTGCTATC GACAGTTGGG GTGACC-TTA GGGTTAATTC CG-GCAAGTA
GTGAGACCTC
B11            GGCTGCTATC GACAGTTGGG GTGACC-TTA GGGTTAATTC CG-GCAAGTA
GTGAGACCTC
B12            GGCTGCTATC GACAGTTGGG GTGACC-TTA GGGTTAATTC CG-GCAAGTA
GTGAGACCTC
B13            GGCTGCTATC GACAGTTGGG GTGACC-TTA GGGTTAATTC CG-GCAAGTA
GTGAGACCTC
B14            GGCTGCTATC GACAGTTGGG GTGACC-TTA GGGTTAATTC CG-GCAAGTA
GTGAGACCTC
B15            GGCTGCTATC GACAGTTGGG GTGACC-TTA GGGTTAATTC CG-GCAAGTA
GTGAGACCTC
B16            GGCTGCTATC GACAGTTGGG GTGACC-TTA GGGTTAATTC CG-GCAAGTA
GTGAGACCTC
KF537632_Ddi   GGCTGCTATC GACAGTTGGG GTGACC-TTA GGGTTAATTC CG-GCAAGTA
GTGAGACCCC
AJ438961_Dde   GGCTGCTATC GACAGTTGGG GTGACC-TTA GGGTTAATTC CG-GCAAGTA
GTGAGACCCC
AJ438960_Dca   GGCTGCTATC GACAGTTGGG GTGACC-TTA GGGTTAATTC CG-GCAAGTA
GTGAGACCCC
AJ438959_Dca   GGCTGCTATC GACAGTTGGG GTGACC-TTA GGGTTAATTC CG-GCAAGTA
GTGAGACCCC
AJ438958_Dga   GGCTGCTATC GACAGTTGGG GTGACC-TTA GGGTTAATTC CG-GCAAGTA
GTGAGACCCC

               ....|....| ....|....| ....|....| ....|....| ....|....|
....|....|
                   965        975        985        995        1005
1015
D1             TGCAGATAG- -TGGACAGGT ATTTTT-AAA ATACAGGAAG GAAGGGACAA
GAGCAGGTCA
D2             TGCAGATAG- -TGGACAGGT ATTTTT-AAA ATACAGGAAG GAAGGGACAA
GAGCAGGTCA
D3             TGCAGATAG- -TGGACAGGT ATTTTT-AAA ATACAGGAAG GAAGGGACAA
GAGCAGGTCA
R1             TGCAGATAG- -TGGACAGGT ATTTTT-AAG ATACAGGAAG GAAGGGACAA
GAGCAGGTCA
R2             TGCAGATAG- -TGGACAGGT ATTTTC-AAG ATACAGGAAG GAAGGGACAA
GAGCAGGTCA
R3             TGCAGATAG- -TGGACAGGT ATTTTC-AAG ATACAGGAAG GAAGGGACAA
GAGCAGGTCA
R4             TGCAGATAG- -TGGACAGGT ATTTTT-AAG ATACAGGAAG GAAGGGACAA
GAGCAGGTCA
R5             TGCAGATAG- -TGGACAGGT ATTTTT-AAG ATACAGGAAG GAAGGGACAA
GAGCAGGTCA
```

R6 TGCAGATAG- -TGGACAGGT ATTTTT-AAG ATACAGGAAG GAAGGGACAA
GAGCAGGTCA
R7 TGCAGATAG- -TGGACAGGT ATTTTT-AAG ATACAGGAAG GAAGGGACAA
GAGCAGGTCA
N1 TGCAGATAG- -TGGACAGGT ATTTTA-AAA ATACAGGAAG GAAGGGACAA
GAGCAGGTCA
N2 TGCAGATAG- -TGGACAGGT ATTTTA-AAA ATACAGGAAG GAAGGGACAA
GAGCAGGTCA
N3 TGCAGATAG- -TGGACAGGT ATTTTA-AAA ATACAGGAAG GAAGGGACAA
GAGCAGGTCA
M1 TGCAGATAG- -TGGACAGGT ATTTTT-AAA ATACAGGAAG GAAGGGACAA
GAGCAGGTCA
M2 TGCAGATAG- -TGGACAGGT ATTTTT-AAA ATACAGGAAG GAAGGGACAA
GAGCAGGTCA
M3 TGCAGATAG- -TGGACAGGT ATTTTT-AAA ATACAGGAAG GAAGGGACAA
GAGCAGGTCA
M4 TGCAGATAG- -TGGACAGGT ATTTTT-AAA ATACAGGAAG GAAGGGACAA
GAGCAGGTCA
M5 TGCAGATAG- -TGGACAGGT ATTTTT-AAA ATACAGGAAG GAAGGGACAA
GAGCAGGTCA
M6 TGCAGATAG- -TGGACAGGT ATTTTT-AAA ATACAGGAAG GAAGGGACAA
GAGCAGGTCA
M7 TGCAGATAG- -TGGACAGGT ATTTTT-AAA ATACAGGAAG GAAGGGACAA
GAGCAGGTCA
M8 TGCAGATAG- -TGGACAGGT ATTTTT-AAA ATACAGGAAG GAAGGGACAA
GAGCAGGTCA
M9 TGCAGATAG- -TGGACAGGT ATTTTT-AAA ATACAGGAAG GAAGGGACAA
GAGCAGGTCA
M10 TGCAGATAG- -TGGACAGGT ATTTTT-AAA ATACAGGAAG GAAGGGACAA
GAGCAGGTCA
M11 TGCAGATAG- -TGGACAGGT ATTTTT-AAA ATACAGGAAG GAAGGGACAA
GAGCAGGTCA
M12 TGCAGATAG- -TGGACAGGT ATTTTT-AAA ATACAGGAAG GAAGGGACAA
GAGCAGGTCA
M13 TGCAGATAG- -TGGACAGGT ATTTTT-AAA ATACAGGAAG GAAGGGACAA
GAGCAGGTCA
M14 TGCAGATAG- -TGGACAGGT ATTTTT-AAA ATACAGGAAG GAAGGGACAA
GAGCAGGTCA
M15 TGCAGATAG- -TGGACAGGT ATTTTT-AAA ATACAGGAAG GAAGGGACAA
GAGCAGGTCA
M16 TGCAGATAG- -TGGACAGGT ATTTTT-AAA ATACAGGAAG GAAGGGACAA
GAGCAGGTCA
M17 TGCAGATAG- -TGGACAGGT ATTTTT-AAA ATACAGGAAG GAAGGGACAA
GAGCAGGTCA
M18 TGCAGATAG- -TGGACAGGT ATTTTT-AAA ATACAGGAAG GAAGGGACAA
GAGCAGGTCA
M19 TGCAGATAG- -TGGACAGGT ATTTTT-AAA ATACAGGAAG GAAGGGACAA
GAGCAGGTCA
M20 TGCAGATAG- -TGGACAGGT ATTTTTCAAA ATACAGGAAG GAAGGGACAA
GAGCAGGTCA
M21 TGCAGATAG- -TGGACAGGT ATTTTTCAAA ATACAGGAAG GAAGGGACAA
GAGCAGGTCA
M22 TGCAGATAG- -TGGACAGGT ATTTTTCAAA ATACAGGAAG GAAGGGACAA
GAGCAGGTCA
M23 TGCAGATAG- -TGGACAGGT ATTTTTCAAA ATACAGGAAG GAAGGGACAA
GAGCAGGTCA
B1 TGCAGTTA-- -TGGACAGGT ATTTTT-AAG ATACAGGAAG GAAGAGACAA
GAGCAGGTCA

B2 TGCAGTTA-- -TGGACAGGT ATTTTT-AAG ATACAGGAAG GAAGAGACAA
GAGCAGGTCA
B3 TGCAGTTA-- -TGGACAGGT ATTTTT-AAG ATACAGGAAG GAAGAGACAA
GAGCAGGTCA
B4 TGCAGTTA-- -TGGACAGGT ATTTTT-AAG ATACAGGAAG GAAGAGACAA
GAGCAGGTCA
B5 TGCAGTTA-- -TGGACAGGT ATTTTT-AAG ATACAGGAAG GAAGAGACAA
GAGCAGGTCA
B6 TGCAGTTA-- -TGGACAGGT ATTTTT-AAG ATACAGGAAG GAAGAGACAA
GAGCAGGTCA
B7 TGCAGTTA-- -TGGACAGGT ATTTTT-AAG ATACAGGAAG GAAGAGACAA
GAGCAGGTCA
B8 TGCAGTTA-- -TGGACAGGT ATTTTT-AAG ATACAGGAAG GAAGAGACAA
GAGCAGGTCA
B9 TGCAGTTA-- -TGGACAGGT ATTTTT-AAG ATACAGGAAG GAAGAGACAA
GAGCAGGTCA
B10 TGCAGTTA-- -TGGACAGGT ATTTTT-AAG ATACAGGAAG GAAGAGACAA
GAGCAGGTCA
B11 TGCAGTTA-- -TGGACAGGT ATTTTT-AAG ATACAGGAAG GAAGAGACAA
GAGCAGGTCA
B12 TGCAGTTA-- -TGGACAGGT ATTTTT-AAG ATACAGGAAG GAAGAGACAA
GAGCAGGTCA
B13 TGCAGTTA-- -TGGACAGGT ATTTTT-AAG ATACAGGAAG GAAGAGACAA
GAGCAGGTCA
B14 TGCAGTTA-- -TGGACAGGT ATTTTT-AAG ATACAGGAAG GAAGAGACAA
GAGCAGGTCA
B15 TGCAGTTA-- -TGGACAGGT ATTTTT-AAG ATACAGGAAG GAAGAGACAA
GAGCAGGTCA
B16 TGCAGTTA-- -TGGACAGGT ATTTTT-AAG ATACAGGAAG GAAGAGACAA
GAGCAGGTCA
KF537632_Ddi TGCTATTAAA TAGGACAGGT ATTTTT-AAA ATACAGGAAG GAAGGGACAA
GAGCAGGTCG
AJ438961_Dde TGCAGGTA-- -TGGACAGGC ATTGTG-AAG ATGCAGGAAG GAGGGGACAA
GAGCAGGTCA
AJ438960_Dca CGCAGTAT-- -TGGACAGGC GTCGTG-AAG ATGCAGGAAG GAGGGGACAA
GAGCAGGTCA
AJ438959_Dca CGCAGTAT-- -TGGACAGGC GTCGTG-AAG ATGCAGGAAG GAGGGGACAA
GAGCAGGTCA
AJ438958_Dga CGTGTATA-- ---GACAGGC ATTGTT-AAG ATGCAGGAAG GAGGGGACAA
GAGCAGGTCA

....|....| ....|....| ....|....| ....|....| ....|....|
....|....|
1025 1035 1045 1055 1065
1075
D1 GTGATGCCCT TAGATGGCCT GGGCTGCACG CGCACTACAG TGGTCATTAT
AAGTAGAAGT
D2 GTGATGCCCT TAGATGGCCT GGGCTGCACG CGCACTACAG TGGTCATTAT
AAGTAGAAGT
D3 GTGATGCCCT TAGATGGCCT GGGCTGCACG CGCACTACAG TGGTCATTAT
AAGTAGAAGT
R1 GTGATGCCCT TAGATGGCCT GGGCTGCACG CGCACTACAG TGGTCATTAT
AAGTAGAAGT
R2 GTGATGCCCT TAGATGGCCT GGGCTGCACG CGCACTACAG TGGTCATTAT
AAGTAGAAGT
R3 GTGATGCCCT TAGATGGCCT GGGCTGCACG CGCACTACAG TGGTCATTAT
AAGTAGAAGT

R4 GTGATGCCCT TAGATGGCCT GGGCTGCACG CGCACTACAG TGGTCATTAT
AAGTAGAAGT
R5 GTGATGCCCT TAGATGGCCT GGGCTGCACG CGCACTACAG TGGTCATTAT
AAGTAGAAAT
R6 GTGATGCCCT TAGATGGCCT GGGCTGCACG CGCACTACAG TGGTCATTAT
AAGTAGAAGT
R7 GTGATGCCCT TAGATGGCCT GGGCTGCACG CGCACTACAG TGGTCATTAT
AAGTAGAAGT
N1 GTGATGCCCT TAGATGGCCT GGGCTGCACG CGCACTACAG TGGTCATTAT
AAGACGAAAG
N2 GTGATGCCCT TAGATGGCCT GGGCTGCACG CGCACTACAG TGGTCATTAT
AAGACGAAAG
N3 GTGATGCCCT TAGATGGCCT GGGCTGCACG CGCACTACAG TGGTCATTAT
AAGACGAAAG
M1 GTGATGCCCT TAGATGGCCT GGGCTGCACG CGCACTACAG TGGTCATTAT
AAGTAGAAAT
M2 GTGATGCCCT TAGATGGCCT GGGCTGCACG CGCACTACAG TGGTCATTAT
AAGTAGAAAT
M3 GTGATGCCCT TAGATGGCCT GGGCTGCACG CGCACTACAG TGGTCATTAT
AAGTAGAAAT
M4 GTGATGCCCT TAGATGGCCT GGGCTGCACG CGCACTACAG TGGTCATTAT
AAGTAGAAAT
M5 GTGATGCCCT TAGATGGCCT GGGCTGCACG CGCACTACAG TGGTCATTAT
AAGTAGAAAT
M6 GTGATGCCCT TAGATGGCCT GGGCTGCACG CGCACTACAG TGGTCATTAT
AAGTAGAAAT
M7 GTGATGCCCT TAGATGGCCT GGGCTGCACG CGCACTACAG TGGTCATTAT
AAGTAGAAAT
M8 GTGATGCCCT TAGATGGCCT GGGCTGCACG CGCACTACAG TGGTCATTAT
AAGTAGAAAT
M9 GTGATGCCCT TAGATGGCCT GGGCTGCACG CGCACTACAG TGGTCATTAT
AAGTAGAAAT
M10 GTGATGCCCT TAGATGGCCT GGGCTGCACG CGCACTACAG TGGTCATTAT
AAGTAGAAAT
M11 GTGATGCCCT TAGATGGCCT GGGCTGCACG CGCACTACAG TGGTCATTAT
AAGTAGAAAT
M12 GTGATGCCCT TAGATGGCCT GGGCTGCACG CGCACTACAG TGGTCATTAT
AAGTAGAAAT
M13 GTGATGCCCT TAGATGGCCT GGGCTGCACG CGCACTACAG TGGTCATTAT
AAGTAGAAAT
M14 GTGATGCCCT TAGATGGCCT GGGCTGCACG CGCACTACAG TGGTCATTAT
AAGTAGAAGT
M15 GTGATGCCCT TAGATGGCCT GGGCTGCACG CGCACTACAG TGGTCATTAT
AAGTAGAAGT
M16 GTGATGCCCT TAGATGGCCT GGGCTGCACG CGCACTACAG TGGTCATTAT
AAGTAGAAGT
M17 GTGATGCCCT TAGATGGCCT GGGCTGCACG CGCACTACAG TGGTCATTAT
AAGTAGAAGT
M18 GTGATGCCCT TAGATGGCCT GGGCTGCACG CGCACTACAG TGGTCATTAT
AAGTAGAAGT
M19 GTGATGCCCT TAGATGGCCT GGGCTGCACG CGCACTACAG TGGTCATTAT
AAGTAGAAGT
M20 GTGATGCCCT TAGATGGCCT GGGCTGCACG CGCACTACAG TGGTCATTAT
AAGTAGAAGT
M21 GTGATGCCCT TAGATGGCCT GGGCTGCACG CGCACTACAG TGGTCATTAT
AAGTAGAAGT
M22 GTGATGCCCT TAGATGGCCT GGGCTGCACG CGCACTACAG TGGTCATTAT
AAGTAGAAGT

M23 GTGATGCCCT TAGATGGCCT GGGCTGCACG CGCACTACAG TGGTCATTAT AAGTAGAAGT
B1 GTGATGCCCT TAGATGGCCT GGGCTGCACG CGCACTACAG TGGTCATTAT AAGATGAAGT
B2 GTGATGCCCT TAGATGGCCT GGGCTGCACG CGCACTACAG TGGTCATTAT AAGTAGAAGT
B3 GTGATGCCCT TAGATGGCCT GGGCTGCACG CGCACTACAG TGGTCATTAT AAGTAGAAGT
B4 GTGATGCCCT TAGATGGCCT GGGCTGCACG CGCACTACAG TGGTCATTAT AAGTAGAAGT
B5 GTGATGCCCT TAGATGGCCT GGGCTGCACG CGCACTACAG TGGTCATTAT AAGTAGAAGT
B6 GTGATGCCCT TAGATGGCCT GGGCTGCACG CGCACTACAG TGGTCATTAT AAGTAGAAGT
B7 GTGATGCCCT TAGATGGCCT GGGCTGCACG CGCACTACAG TGGTCATTAT AAGTAGAAGT
B8 GTGATGCCCT TAGATGGCCT GGGCTGCACG CGCACTACAG TGGTCATTAT AAGTAGAAGT
B9 GTGATGCCCT TAGATGGCCT GGGCTGCACG CGCACTACAG TGGTCATTAT AAGTAGAAGT
B10 GTGATGCCCT TAGATGGCCT GGGCTGCACG CGCACTACAG TGGTCATTAT AAGTAGAAGT
B11 GTGATGCCCT TAGATGGCCT GGGCTGCACG CGCACTACAG TGGTCATTAT AAGTAGAAGT
B12 GTGATGCCCT TAGATGGCCT GGGCTGCACG CGCACTACAG TGGTCATTAT AAGTAGAAGT
B13 GTGATGCCCT TAGATGGCCT GGGCTGCACG CGCACTACAG TGGTCATTAT AAGTAGAAGT
B14 GTGATGCCCT TAGATGGCCT GGGCTGCACG CGCACTACAG TGGTCATTAT AAGTAGAAGT
B15 GTGATGCCCT TAGATGGCCT GGGCTGCACG CGCACTACAG TGGTCATTAT AAGTAGAAGT
B16 GTGATGCCCT TAGATGGCCT GGGCTGCACG CGCACTACAG TGGTCATTAT AAGTAGAAGT
KF537632_Ddi GTGATGCCCT TAGATGGCCT GGGCTGCACG CGCACTACAG TGGTCATTAT AATGAGTAAA
AJ438961_Dde GTGATGCCCT TAGATGGCCT GGGCTGCACG CGCACTACAG TGGTCATTAT AGGAAGAGAG
AJ438960_Dca GTGATGCCCT TAGATGGCCT GGGCTGCACG CGCACTACAG TGGTCATTAT AAGGAGAAGT
AJ438959_Dca GTGATGCCCT TAGATGGCCT GGGCTGCACG CGCACTACAG TGGTCATTAT AAGAAGGAAT
AJ438958_Dga GTGATGCCCT TAGATGGCCT GGGCTGCACG CGCACTACAG TGGTCATAAT AAGAAGTAAG

....|....| ....|....| ....|....| ....|....| ....|....| ....|....|
1085 1095 1105 1115 1125 1135
D1 TAGA-TATAA AGATGATCGA GAGGGACTGA GCTTTGTAAG AGGCTCACGA ACGAGGAATT
D2 TAGA-TATAA AGATGATCGA GAGGGACTGA GCTTTGTAAG AGGCTCACGA ACGAGGAATT
D3 TAGA-TATAA AGATGATCGA GAGGGACTGA GCTTTGTAAG AGGCTCACGA ACGAGGAATT
R1 TAGA-TATAA AGATGATCGA GAGGGACTGA GCTTTGTAAG AGGCTCACGA ACGAGGAATT

R2 TAGA-TATAA AGATGATCGA GAGGGACTGA GCTTTGTAAG AGGCTCACGA
ACGAGGAATT
R3 TAGA-TATAA AGATGATCGA GAGGGACTGA GCTTTGTAAG AGGCTCACGA
ACGAGGAATT
R4 TAGA-TATAA AGATGATCGA GAGGGACTGA GCTTTGTAAG AGGCTCACGA
ACGAGGAATT
R5 TAGA-TATAA AGATGATCGA GAGGGACTGA GCTTTGTAAG AGGCTCACGA
ACGAGGAATT
R6 TAGA-TATAA AGATGATCGA GAGGGACTGA GCTTTGTAAG AGGCTCACGA
ACGAGGAATT
R7 TAGA-TATAA AGATGATCGA GAGGGACTGA GCTTTGTAAG AGGCTCACGA
ACGAGGAATT
N1 TAGA-TATAA AGATGATCGA GAGGGACTGA GCTTTGTAAG AGGCTCACGA
ACGAGGAATT
N2 TAGA-TATAA AGATGATCGA GAGGGACTGA GCTTTGTAAG AGGCTCACGA
ACGAGGAATT
N3 TAGA-TATAA AGATGATCGA GAGGGACTGA GCTTTGTAAG AGGCTCACGA
ACGAGGAATT
M1 TAGA-AGTAA AAATGATCGA GAGGGACTGA GCTTTGTAAG AGGCTCACGA
ACGAGGAATT
M2 TAGA-AGTAA AAATGATCGA GAGGGACTGA GCTTTGTAAG AGGCTCACGA
ACGAGGAATT
M3 TAGA-AATAA AGATGATCGA GAGGGACTGA GCTTTGTAAG AGGCTCACGA
ACGAGGAATT
M4 TAGA-AATAA AAATGATCGA GAGGGACTGA GCTTTGTAAG AGGCTCACGA
ACGAGGAATT
M5 TAGA-AATAA AAATGATCGA GAGGGACTGA GCTTTGTAAG AGGCTCACGA
ACGAGGAATT
M6 TAGA-AGTAA AAATGATCGA GAGGGACTGA GCTTTGTAAG AGGCTCACGA
ACGAGGAATT
M7 TAGA-AGTAA AAATGATCGA GAGGGACTGA GCTTTGTAAG AGGCTCACGA
ACGAGGAATT
M8 TAGA-AATAA AAATGATCGA GAGGGACTGA GCTTTGTAAG AGGCTCACGA
ACGAGGAATT
M9 TAGA-AATAA AAATGATCGA GAGGGACTGA GCTTTGTAAG AGGCTCACGA
ACGAGGAATT
M10 TAGA-AATAA AAATGATCGA GAGGGACTGA GCTTTGTAAG AGGCTCACGA
ACGAGGAATT
M11 TAGA-AGTAA AAATGATCGA GAGGGACTGA GCTTTGTAAG AGGCTCACGA
ACGAGGAATT
M12 TAGA-AGTAA AAATGATCGA GAGGGACTGA GCTTTGTAAG AGGCTCACGA
ACGAGGAATT
M13 TAGA-AGTAA AAATGATCGA GAGGGACTGA GCTTTGTAAG AGGCTCACGA
ACGAGGAATT
M14 TAGA-AGTAA AGATGATCGA GAGGGACTGA GCTTTGTAAG AGGCTCACGA
ACGAGGAATT
M15 TAGA-AGTAA AGATGATCGA GAGGGACTGA GCTTTGTAAG AGGCTCACGA
ACGAGGAATT
M16 TAGA-AGTAA AGATGATCGA GAGGGACTGA GCTTTGTAAG AGGCTCACGA
ACGAGGAATT
M17 TAGA-AGTAA AGATGATCGA GAGGGACTGA GCTTTGTAAG AGGCTCACGA
ACGAGGAATT
M18 TAGA-AGTAA AGATGATCGA GAGGGACTGA GCTTTGTAAG AGGCTCACGA
ACGAGGAATT
M19 TAGA-AGTAA AGATGATCGA GAGGGACTGA GCTTTGTAAG AGGCTCACGA
ACGAGGAATT
M20 TAGA-TATAA AGATGATCGA GAGGGACTGA GCTTTGTAAG AGGCTCACGA
ACGAGGAATT

M21 TAGA-TATAA AGATGATCGA GAGGGACTGA GCTTTGTAAG AGGCTCACGA
ACGAGGAATT
M22 TAGA-TATAA AGATGATCGA GAGGGACTGA GCTTTGTAAG AGGCTCACGA
ACGAGGAATT
M23 TAGA-TATAA AGATGATCGA GAGGGACTGA GCTTTGTAAG AGGCTCACGA
ACGAGGAATT
B1 TAGATTTTAA AGATGATCGA GAGGGACTGG GCTTTGTAAG AGGCCCAAGA
ACGAGGAATT
B2 TAGATTTTAA AGATGATCGA GAGGGACTGG GCTTTGTAAG AGGCCCAAGA
ACGAGGAATT
B3 TAGATTTTAA AGATGATCGA GAGGGACTGG GCTTTGTAAG AGGCCCAAGA
ACGAGGAATT
B4 TAGATTTTAA AGATGATCGA GAGGGACTGG GCTTTGTAAG AGGCCCAAGA
ACGAGGAATT
B5 TAGATTTTAA AGATGATCGA GAGGGACTGG GCTTTGTAAG AGGCCCAAGA
ACGAGGAATT
B6 TAGATTTTAA AAATGATCGA GAGGGACTGG GCTTTGTAAG AGGCCCAAGA
ACGAGGAATT
B7 TAGATTTTAA AGATGATCGA GAGGGACTGG GCTTTGTAAG AGGCCCAAGA
ACGAGGAATT
B8 TAGATTTTAA AGATGATCGA GAGGGACTGG GCTTTGTAAG AGGCCCAAGA
ACGAGGAATT
B9 TAGATTTTAA AGATGATCGA GAGGGACTGG GCTTTGTAAG AGGCCCAAGA
ACGAGGAATT
B10 TAGATTTTAA AGATGATCGA GAGGGACTGG GCTTTGTAAG AGGCCCAAGA
ACGAGGAATT
B11 TAGATTTTAA AGATGATCGA GAGGGACTGG GCTTTGTAAG AGGCCCAAGA
ACGAGGAATT
B12 TAGATTTTAA AAATGATCGA GAGGGACTGG GCTTTGTAAG AGGCCCAAGA
ACGAGGAATT
B13 TAGATTTTAA AGATGATCGA GAGGGACTGG GCTTTGTAAG AGGCCCAAGA
ACGAGGAATT
B14 TAGATTTTAA AGATGATCGA GAGGGACTGG GCTTTGTAAG AGGCCCAAGA
ACGAGGAATT
B15 TAGATTTTAA AGATGATCGA GAGGGACTGG GCTTTGTAAG AGGCCCAAGA
ACGAGGAATT
B16 TAGATTTTAA AAATGATCGA GAGGGACTGG GCTTTGTAAG AGGCCCAAGA
ACGAGGAATT
KF537632_Ddi TAGA-AGTAA AAATGATCGA GAGGGACTGG GCTTTGTAAG AGGCCCACGA
ACGAGGAATT
AJ438961_Dde TAGA-GACAA AGATGATCGA GAGGGACTGG GCTTTGTAAG AGGCCCACGA
ACGAGGAATT
AJ438960_Dca TAGA-AATAA AGATGATCGA GAGGGACTGG GCTTTGTAAG AGGCCCACGA
ACGAGGAATT
AJ438959_Dca TAAA-AGTAA AGATGATCGA GAGGGACTGG GCTTTGTAAG AGGCCCACGA
ACGAGGAATT
AJ438958_Dga TAGA-AGGAA TTATGATCGA GAGGGACTGG GCTTTGTAAT AGGCCCACGA
ACGAGGAATT

....|....| ....|....| ....|....| ....|....| ....|....|
....|....|
1145 1155 1165 1175 1185
1195
D1 GCTAGTAATC GTAGGCTCAT TAAGATACGA TGAATATGTC CCTGTACCTT
GTACACACCG
D2 GCTAGTAATC GTAGGCTCAT TAAGATACGA TGAATATGTC CCTGTACCTT
GTACACACCG

D3 GCTAGTAATC GTAGGCTCAT TAAGATACGA TGAATATGTC CCTGTACCTT
GTACACACCG
R1 GCTAGTAATC GTAGGCTCAT TAAGATACGA TGAATATGTC CCTGTACCTT
GTACACACCG
R2 GCTAGTAATC GTAGGCTCAT TAAGATACGA TGAATATGTC CCTGTACCTT
GTACACACCG
R3 GCTAGTAATC GTAGGCTCAT TAAGATACGA TGAATATGTC CCTGTACCTT
GTACACACCG
R4 GCTAGTAATC GTAGGCTCAT TAAGATACGA TGAATATGTC CCTGTACCTT
GTACACACCG
R5 GCTAGTAATC GTAGGCTCAT TAAGATACGA TGAATATGTC CCTGTACCTT
GTACACACCG
R6 GCTAGTAATC GTAGGCTCAT TAAGATACGA TGAATATGTC CCTGTACCTT
GTACACACCG
R7 GCTAGTAATC GTAGGCTCAT TAAGATACGA TGAATATGTC CCTGTACCTT
GTACACACCG
N1 GCTAGTAATC GTAGGCTCAT TAAGATACGA TGAATATGTC CCTGTACCTT
GTACACACCG
N2 GCTAGTAATC GTAGGCTCAT TAAGATACGA TGAATATGTC CCTGTACCTT
GTACACACCG
N3 GCTAGTAATC GTAGGCTCAT TAAGATACGA TGAATATGTC CCTGTACCTT
GTACACACCG
M1 GCTAGTAATC GTAGGCTCAT TAAGATACGA TGAATATGTC CCTGTACCTT
GTACACACCG
M2 GCTAGTAATC GTAGGCTCAT TAAGATACGA TGAATATGTC CCTGTACCTT
GTACACACCG
M3 GCTAGTAATC GTAGGCTCAT TAAGATACGA TGAATATGTC CCTGTACCTT
GTACACACCG
M4 GCTAGTAATC GTAGGCTCAT TAAGATACGA TGAATATGTC CCTGTACCTT
GTACACACCG
M5 GCTAGTAATC GTAGGCTCAT TAAGATACGA TGAATATGTC CCTGTACCTT
GTACACACCG
M6 GCTAGTAATC GTAGGCTCAT TAAGATACGA TGAATATGTC CCTGTACCTT
GTACACACCG
M7 GCTAGTAATC GTAGGCTCAT TAAGATACGA TGAATATGTC CCTGTACCTT
GTACACACCG
M8 GCTAGTAATC GTAGGCTCAT TAAGATACGA TGAATATGTC CCTGTACCTT
GTACACACCG
M9 GCTAGTAATC GTAGGCTCAT TAAGATACGA TGAATATGTC CCTGTACCTT
GTACACACCG
M10 GCTAGTAATC GTAGGCTCAT TAAGATACGA TGAATATGTC CCTGTACCTT
GTACACACCG
M11 GCTAGTAATC GTAGGCTCAT TAAGATACGA TGAATATGTC CCTGTACCTT
GTACACACCG
M12 GCTAGTAATC GTAGGCTCAT TAAGATACGA TGAATATGTC CCTGTACCTT
GTACACACCG
M13 GCTAGTAATC GTAGGCTCAT TAAGATACGA TGAATATGTC CCTGTACCTT
GTACACACCG
M14 GCTAGTAATC GTAGGCTCAT TAAGATACGA TGAATATGTC CCTGTACCTT
GTACACACCG
M15 GCTAGTAATC GTAGGCTCAT TAAGATACGA TGAATATGTC CCTGTACCTT
GTACACACCG
M16 GCTAGTAATC GTAGGCTCAT TAAGATACGA TGAATATGTC CCTGTACCTT
GTACACACCG
M17 GCTAGTAATC GTAGGCTCAT TAAGATACGA TGAATATGTC CCTGTACCTT
GTACACACCG
M18 GCTAGTAATC GTAGGCTCAT TAAGATACGA TGAATATGTC CCTGTACCTT
GTACACACCG

M19 GCTAGTAATC GTAGGCTCAT TAAGATACGA TGAATATGTC CCTGTACCTT
GTACACACCG
M20 GCTAGTAATC GTAGGCTCAT TAAGATACGA TGAATATGTC CCTGTACCTT
GTACACACCG
M21 GCTAGTAATC GTAGGCTCAT TAAGATACGA TGAATATGTC CCTGTACCTT
GTACACACCG
M22 GCTAGTAATC GTAGGCTCAT TAAGATACGA TGAATATGTC CCTGTACCTT
GTACACACCG
M23 GCTAGTAATC GTAGGCTCAT TAAGATACGA TGAATATGTC CCTGTACCTT
GTACACACCG
B1 GCTAGTAATC GTAGGCTCAT TAAGATACGA TGAATATGTC CCTGTACCTT
GTACACACCG
B2 GCTAGTAATC GTAGGCTCAT TAAGATACGA TGAATATGTC CCTGTACCTT
GTACACACCG
B3 GCTAGTAATC GTAGGCTCAT TAAGATACGA TGAATATGTC CCTGTACCTT
GTACACACCG
B4 GCTAGTAATC GTAGGCTCAT TAAGATACGA TGAATATGTC CCTGTACCTT
GTACACACCG
B5 GCTAGTAATC GTAGGCTCAT TAAGATACGA TGAATATGTC CCTGTACCTT
GTACACACCG
B6 GCTAGTAATC GTAGGCTCAT TAAGATACGA TGAATATGTC CCTGTACCTT
GTACACACCG
B7 GCTAGTAATC GTAGGCTCAT TAAGATACGA TGAATATGTC CCTGTACCTT
GTACACACCG
B8 GCTAGTAATC GTAGGCTCAT TAAGATACGA TGAATATGTC CCTGTACCTT
GTACACACCG
B9 GCTAGTAATC GTAGGCTCAT TAAGATACGA TGAATATGTC CCTGTACCTT
GTACACACCG
B10 GCTAGTAATC GTAGGCTCAT TAAGATACGA TGAATATGTC CCTGTACCTT
GTACACACCG
B11 GCTAGTAATC GTAGGCTCAT TAAGATACGA TGAATATGTC CCTGTACCTT
GTACACACCG
B12 GCTAGTAATC GTAGGCTCAT TAAGATACGA TGAATATGTC CCTGTACCTT
GTACACACCG
B13 GCTAGTAATC GTAGGCTCAT TAAGATACGA TGAATATGTC CCTGTACCTT
GTACACACCG
B14 GCTAGTAATC GTAGGGTCAT TAAAATACGA TGAATATGTC CCTGTACCTT
GTACACACCG
B15 GCTAGTAATC GTAGGCTCAT TAAGATACGA TGAATATGTC CCTGTACCTT
GTACACACCG
B16 GCTAGTAATC GTAGGCTCAT TAAGATACGA TGAATATGTC CCTGTACCTT
GTACACACCG
KF537632_Ddi GCTAGTAATC GTAGGCTCAT TAAGATACGA TGAATATGTC CCTGTACCTT
GTACACACCG
AJ438961_Dde GCTAGTAATC GCAGGCTCAT TAAGATGCGA TGAATATGTC CCTGTACCTT
GTACACACCG
AJ438960_Dca GCTAGTAATC GCAGGCTCAT TAGGATGCGA TGAATATGTC CCTGTACCTT
GTACACACCG
AJ438959_Dca GCTAGTAATC GCAGGCTCAT TAGGATGCGA TGAATATGTC CCTGTACCTT
GTACACACCG
AJ438958_Dga GCTAGTAATC GTAGGCTCAT TAGGATACGA TGAATATGTC CCTGTACCTT
GTACACACCG

....|....| ....|....| ....|....| ....|....| ....|....|
....|....|
1205 1215 1225 1235 1245
1255

D1 CCCGTCGTTA TCGAAGATGG AATTGTGTGC GAACGAGCAA CAAGCGAGTG
AGCGCATAGT
D2 CCCGTCGTTA TCGAAGATGG AATTGTGTGC GAACGAGCAA CAAGCGAGTG
AGCGCATAGT
D3 CCCGTCGTTA TCGAAGATGG AATTGTGTGC GAACGAGCAA CAAGCGAGTG
AGCGCATAGT
R1 CCCGTCGTTA TCGAAGATGG AATTGTGTGC GAACGAGCAT TAAGCGAGTG
AGCGCATAGT
R2 CCCGTCGTTA TCGAAGATGG AATTGTGTGC GAACAAGCAT TAAGCGAGTG
AGCGCATAGT
R3 CCCGTCGTTA TCGAAGATGG AATTGTGTGC GAACAAGCAT TAAGCGAGTG
AGCGCATAGT
R4 CCCGTCGTTA TCGAAGATGG AATTGTGTGC GAACGAGCAT TAAGCGAGTG
AGCGCATAGT
R5 CCCGTCGTTA TCGAAGATGG AATTGTGTGC GAACGAGCAT TAAGCGAGTG
AGCGCATAGT
R6 CCCGTCGTTA TCGAAGATGG AATTGTGTGC GAACGAGCAT TAAGCGAGTG
AGCGCATAGT
R7 CCCGTCGTTA TCGAAGATGG AATTGTGTGC GAACGAGCAT TAAGCGAGTG
AGCGCATAGT
N1 CCCGTCGTTA TCGAAGATGG AATTGTGTGC GAACGAGCAA TAAGCGAGTG
AGCGCATAGT
N2 CCCGTCGTTA TCGAAGATGG AATTGTGTGC GAACGAGCAA TAAGCGAGTG
AGCGCATAGT
N3 CCCGTCGTTA TCGAAGATGG AATTGTGTGC GAACGAGCAA TAAGCGAGTG
AGCGCATAGT
M1 CCCGTCGTTA TCGAAGATGG AATTGTGTGC GAACGAGCAA CAAGCGAGTG
AGCGCATAGT
M2 CCCGTCGTTA TCGAAGATGG AATTGTGTGC GAACGAGCAA CAAGCGAGTG
AGCGCATAGT
M3 CCCGTCGTTA TCGAAGATGG AATTGTGTGC GAACGAGCAA CAAGCGAGTG
AGCGCATAGT
M4 CCCGTCGTTA TCGAAGATGG AATTGTGTGC GAACGAGCAA CAAGCGAGTG
AGCGCATAGT
M5 CCCGTCGTTA TCGAAGATGG AATTGTGTGC GAACGAGCAA CAAGCGAGTG
AGCGCATAGT
M6 CCCGTCGTTA TCGAAGATGG AATTGTGTGC GAACGAGCAA CAAGCGAGTG
AGCGCATAGT
M7 CCCGTCGTTA TCGAAGATGG AATTGTGTGC GAACGAGCAA CAAGCGAGTG
AGCGCATAGT
M8 CCCGTCGTTA TCGAAGATGG AATTGTGTGC GAACGAGCAA CAAGCGAGTG
AGCGCATAGT
M9 CCCGTCGTTA TCGAAGATGG AATTGTGTGC GAACGAGCAA CAAGCGAGTG
AGCGCATAGT
M10 CCCGTCGTTA TCGAAGATGG AATTGTGTGC GAACGAGCAA CAAGCGAGTG
AGCGCATAGT
M11 CCCGTCGTTA TCGAAGATGG AATTGTGTGC GAACGAGCAA CAAGCGAGTG
AGCGCATAGT
M12 CCCGTCGTTA TCGAAGATGG AATTGTGTGC GAACGAGCAA CAAGCGAGTG
AGCGCATAGT
M13 CCCGTCGTTA TCGAAGATGG AATTGTGTGC GAACGAGCAA CAAGCGAGTG
AGCGCATAGT
M14 CCCGTCGTTA TCGAAGATGG AATTGTGTGC GAACGAGCAA CAAGCGAGTG
AGCGCATAGT
M15 CCCGTCGTTA TCGAAGATGG AATTGTGTGC GAACGAGCAA CAAGCGAGTG
AGCGCATAGT
M16 CCCGTCGTTA TCGAAGATGG AATTGTGTGC GAACGAGCAA CAAGCGAGTG
AGCGCATAGT

M17 CCCGTCGTTA TCGAAGATGG AATTGTGTGC GAACGAGCAA CAAGCGAGTG
AGCGCATAGT
M18 CCCGTCGTTA TCGAAGATGG AATTGTGTGC GAACGAGCAA CAAGCGAGTG
AGCGCATAGT
M19 CCCGTCGTTA TCGAAGATGG AATTGTGTGC GAACGAGCAA CAAGCGAGTG
AGCGCATAGT
M20 CCCGTCGTTA TCGAAGATGG AATTGTGTGC GAACGAGCAA CAAGCGAGTG
AGCGCATAGT
M21 CCCGTCGTTA TCGAAGATGG AATTGTGTGC GAACGAGCAA CAAGCGAGTG
AGCGCATAGT
M22 CCCGTCGTTA TCGAAGATGG AATTGTGTGC GAACGAGCAA CAAGCGAGTG
AGCGCATAGT
M23 CCCGTCGTTA TCGAAGATGG AATTGTGTGC GAACGAGCAA CAAGCGAGTG
AGCGCATAGT
B1 CCCGTCGTTA TCGAAGATGG AATTGTGTGC GAACGAGCAA CAAGCGAGTG
AGCGCATAGT
B2 CCCGTCGTTA TCGAAGATGG AATTGTGTGC GAACGAGCAA CAAGCGAGTG
AGCGCATAGT
B3 CCCGTCGTTA TCGAAGATGG AATTGTGTGC GAACGAGCAA CAAGCGAGTG
AGCGCATAGT
B4 CCCGTCGTTA TCGAAGATGG AATTGTGTGC GAACGAGCAA CAAGCGAGTG
AGCGCATAGT
B5 CCCGTCGTTA TCGAAGATGG AATTGTGTGC GAACGAGCAA CAAGCGAGTG
AGCGCATAGT
B6 CCCGTCGTTA TCGAAGATGG AATTGTGTGC GAACGAGCAA CAAGCGAGTG
AGCGCATAGT
B7 CCCGTCGTTA TCGAAGATGG AATTGTGTGC GAACGAGCAA CAAGCGAGTG
AGCGCATAGT
B8 CCCGTCGTTA TCGAAGATGG AATTGTGTGC GAACGAGCAA CAAGCGAGTG
AGCGCATAGT
B9 CCCGTCGTTA TCGAAGATGG AATTGTGTGC GAACGAGCAA CAAGCGAGTG
AGCGCATAGT
B10 CCCGTCGTTA TCGAAGATGG AATTGTGTGC GAACGAGCAA CAAGCGAGTG
AGCGCATAGT
B11 CCCGTCGTTA TCGAAGATGG AATTGTGTGC GAACGAGCAA CAAGCGAGTG
AGCGCATAGT
B12 CCCGTCGTTA TCGAAGATGG AATTGTGTGC GAACGAGCAA CAAGCGAGTG
AGCGCATAGT
B13 CCCGTCGTTA TCGAAGATGG AATTGTGTGC GAACGAGCAA CAAGCGAGTG
AGCGCATAGT
B14 CCCGTCGTTA TCGAAGATGG AATTGTGTGC GAACGAGCAA CAAGCGAGTG
AGCGCATAGT
B15 CCCGTCGTTA TCGAAGATGG AATTGTGTGC GAACGAGCAA CAAGCGAGTG
AGCGCATAGT
B16 CCCGTCGTTA TTGAAGATGG AATTGTGTGC GAACGAGCAA CAAGCGAGTG
AGCGCATAGT
KF537632_Ddi CCCGTCGTTA TCGAAGATGG AATTGTGTGC GAACGAGCAT TAAGCGAGTG
AGCGCATAGT
AJ438961_Dde CCCGTCGTTA TCGAAGATGG AATTGTATGC GAACGAGCAG CAAGCGAGTG
AGCGTATAGT
AJ438960_Dca CCCGTCGTTA TCGAAGATGG AATTGTATGC GAACGAGCAG CAAGCGAGTG
AGCGTATAGT
AJ438959_Dca CCCGTCGTTA TCGAAGATGG AATTGTATGC GAACGAGCAG CAAGCGAGTG
AGCGTATAGT
AJ438958_Dga CCCGTCGTTA TCGAAGATGG AATTGTATGC GAACGAGCGT GA-GCGAGTG
AGCGTGCGGT

```
              ....|....| ....|....| ....|....| ....|....| ....|....|
....|....|
                  1265       1275       1285       1295       1305
1315
D1            TCTAGATGTG ATAAAAGTCG TAACAAGGCA ACTGTAGGAG AACCTGTAGT
TGGATCATAC
D2            TCTAGATGTG ATAAAAGTCG TAACAAGGCA ACTGTAGGAG AACCTGTAGT
TGGATCATAC
D3            TCTAGATGTG ATAAAAGTCG TAACAAGGCA ACTGTAGGAG AACCTGTAGT
TGGATCATAC
R1            TCTAGATGTG ATAAAAGTCG TAACAAGGCA ACTGTAGGAG AACCTGTAGT
TGGATCATAC
R2            TCTAGATGTG ATAAAAGTCG TAACAAGGCA ACTGTAGGAG AACCTGTAGT
TGGATCATAC
R3            TCTAGATGTG ATAAAAGTCG TAACAAGGCA ACTGTAGGAG AACCTGTAGT
TGGATCATAC
R4            TCTAGATGTG ATAAAAGTCG TAACAAGGCA ACTGTAGGAG AACCTGTAGT
TGGATCATAC
R5            TCTAGATGTG ATAAAAGTCG TAACAAGGCA ACTGTAGGAG AACCTGTAGT
TGGATCATAC
R6            TCTAGATGTG ATAAAAGTCG TAACAAGGCA ACTGTAGGAG AACCTGTAGT
TGGATCATAC
R7            TCTAGATGTG ATAAAAGTCG TAACAAGGCA ACTGTAGGAG AACCTGTAGT
TGGATCATAC
N1            TCTAGATGTG ATAAAAGTCG TAACAAGGCA ACTGTAGGAG AACCTGTAGT
TGGATCATAC
N2            TCTAGATGTG ATAAAAGTCG TAACAAGGCA ACTGTAGGAG AACCTGTAGT
TGGATCATAC
N3            TCTAGATGTG ATAAAAGTCG TAACAAGGCA ACTGTAGGAG AACCTGTAGT
TGGATCATAC
M1            TCTAGATGTG ATAAAAGTCG TAACAAGGCA ACTGTAGGAG AACCTGTAGT
TGGATCATAC
M2            TCTAGATGTG ATAAAAGTCG TAACAAGGCA ACTGTAGGAG AACCTGTAGT
TGGATCATAC
M3            TCTAGATGTG ATAAAAGTCG TAACAAGGCA ACTGTAGGAG AACCTGTAGT
TGGATCATAC
M4            TCTAGATGTG ATAAAAGTCG TAACAAGGCA ACTGTAGGAG AACCTGTAGT
TGGATCATAC
M5            TCTAGATGTG ATAAAAGTCG TAACAAGGCA ACTGTAGGAG AACCTGTAGT
TGGATCATAC
M6            TCTAGATGTG ATAAAAGTCG TAACAAGGCA ACTGTAGGAG AACCTGTAGT
TGGATCATAC
M7            TCTAGATGTG ATAAAAGTCG TAACAAGGCA ACTGTAGGAG AACCTGTAGT
TGGATCATAC
M8            TCTAGATGTG ATAAAAGTCG TAACAAGGCA ACTGTAGGAG AACCTGTAGT
TGGATCATAC
M9            TCTAGATGTG ATAAAAGTCG TAACAAGGCA ACTGTAGGAG AACCTGTAGT
TGGATCATAC
M10           TCTAGATGTG ATAAAAGTCG TAACAAGGCA ACTGTAGGAG AACCTGTAGT
TGGATCATAC
M11           TCTAGATGTG ATAAAAGTCG TAACAAGGCA ACTGTAGGAG AACCTGTAGT
TGGATCATAC
M12           TCTAGATGTG ATAAAAGTCG TAACAAGGCA ACTGTAGGAG AACCTGTAGT
TGGATCATAC
M13           TCTAGATGTG ATAAAAGTCG TAACAAGGCA ACTGTAGGAG AACCTGTAGT
TGGATCATAC
M14           TCTAGATGTG ATAAAAGTCG TAACAAGGCA ACTGTAGGAG AACCTGTAGT
TGGATCATAC
```

M15 TCTAGATGTG ATAAAAGTCG TAACAAGGCA ACTGTAGGAG AACCTGCAGT
TGGATCATAC
M16 TCTAGATGTG ATAAAAGTCG TAACAAGGCA ACTGTAGGAG AACCTGCAGT
TGGATCATAC
M17 TCTAGATGTG ATAAAAGTCG TAACAAGGCA ACTGTAGGAG AACCTGCAGT
TGGATCATAC
M18 TCTAGATGTG ATAAAAGTCG TAACAAGGCA ACTGTAGGAG AACCTGTAGT
TGGATCATAC
M19 TCTAGATGTG ATAAAAGTCG TAACAAGGCA ACTGTAGGAG AACCTGTAGT
TGGATCATAC
M20 TCTAGATGTG ATAAAAGTCG TAACAAGGCA ACTGTAGGAG AACCTGTAGT
TGGATCATAC
M21 TCTAGATGTG ATAAAAGTCG TAACAAGGCA ACTGTAGGAG AACCTGTAGT
TGGATCATAC
M22 TCTAGATGTG ATAAAAGTCG TAACAAGGCA ACTGTAGGAG AACCTGTAGT
TGGATCATAC
M23 TCTAGATGTG ATAAAAGTCG TAACAAGGCA ACTGTAGGAG AACCTGTAGT
TGGATCATAC
B1 TCTAGATGTG ATAAAAGTCG TAACAAGGCA ACTGTAGGAG AACCTGTAGT
TGGATCATAC
B2 TCTAGATGTG ATAAAAGTCG TAACAAGGCA ACTGTAGGAG AACCTGTAGT
TGGATCATAC
B3 TCTAGATGTG ATAAAAGTCG TAACAAGGCA ACTGTAGGAG AACCTGTAGT
TGGATCATAC
B4 TCTAGATGTG ATAAAAGTCG TAACAAGGCA ACTGTAGGAG AACCTGTAGT
TGGATCATAC
B5 TCTAGATGTG ATAAAAGTCG TAACAAGGCA ACTGTAGGAG AACCTGTAGT
TGGATCATAC
B6 TCTAGATGTG ATAAAAGTCG TAACAAGGCA ACTGTAGGAG AACCTGTAGT
TGGATCATAC
B7 TCTAGATGTG ATAAAAGTCG TAACAAGGCA ACTGTAGGAG AACCTGTAGT
TGGATCATAC
B8 TCTAGATGTG ATAAAAGTCG TAACAAGGCA ACTGTAGGAG AACCTGTAGT
TGGATCATAC
B9 TCTAGATGTG ATAAAAGTCG TAACAAGGCA ACTGTAGGAG AACCTGTAGT
TGGATCATAC
B10 TCTAGATGTG ATAAAAGTCG TAACAAGGCA ACTGTAGGAG AACCTGTAGT
TGGATCATAC
B11 TCTAGATGTG ATAAAAGTCG TAACAAGGCA ACTGTAGGAG AACCTGTAGT
TGGATCATAC
B12 TCTAGATGTG ATAAAAGTCG TAACAAGGCA ACTGTAGGAG AACCTGTAGT
TGGATCATAC
B13 TCTAGATGTG ATAAAAGTCG TAACAAGGCA ACTGTAGGAG AACCTGTAGT
TGGATCATAC
B14 TCTAGATGTG ATAAAAGTCG TAACAAGGCA ACTGTAGGAG AACCTGTAGT
TGGATCATAC
B15 TCTAGATGTG ATAAAAGTCG TAACAAGGCA ACTGTAGGAG AACCTGTAGT
TGGATCATAC
B16 TCTAGATGTG ATAAAAGTCG TAACAAGGCA ACTGTAGGAG AACCTGTAGT
TGGATCATAC
KF537632_Ddi TCTAGATGTG ATAAAAGTCG TAACAAGGCA ACTGTAGGAG AACCTGTAGT
TGGATCATAC
AJ438961_Dde TCTAGATGTG ATAAAAGTCG TAACAAGGCA ACTGTAGGAG AACCTGTAGT
TGGATCAGAC
AJ438960_Dca TCTAGATGTG ATAAAAGTCG TAACAAGGCA ACTGTAGGAG AACCTGTAGT
TGGATCACAC
AJ438959_Dca TCTAGATGTG ATAAAAGTCG TAACAAGGCA ACTGTAGGAG AACCTGTAGT
TGGATCACAC

```
AJ438958_Dga    TCTAGATGTG ATAAAAGTCG TAACAAGGCA ACTGTAGGAG AACCTGTAGT
TGGATCACGA

                ....|....| ....|....| ....|....| ....|....| ....|....|
....|....|
                    1325       1335       1345       1355       1365
1375
D1              AGATTTTCTT A----ATAGA AGTTTG---A GAAGTATTCC TTACGCAAGG
GATCGTTTGG
D2              AGATTTTCTT A----ATAGA AGTTTG---A GAAGTATTCC TTACGCAAGG
GATCGTTTGG
D3              AGATTTTCTT A----ATAGA AGTTTG---A GAAGTATTCC TTACGCAAGG
GATCGTTTGG
R1              AGATTGTTTA T----CATAG TTTTTTT--- AGTATATTCC TTACGCAAGG
GATCGTTTGG
R2              AGATTGTTTT T----TAACA GTTTTATTTA TAAATATTCC TTACGCAAGG
GATCGTTTGG
R3              AGATTGTTTT T----TAACA GTTTTATTTA TAAATATTCC TTACGCAAGG
GATCGTTTGG
R4              AGATTGTTTA T----TAATA GTTTTTTA-A AGTATATTCC TTACGCAAGG
GATCGTTTGG
R5              AGATTGTTTA A----TAGAA AGTTTTT--A ATTTAATTCC TTACGCAAGG
GATCGTTTGG
R6              AGATTGTTTA T----TTAAA AAGTTTTA-A TTCTAATTCC TTACGCAAGG
GATCGTTTGG
R7              AGATTGTTTA T----TTGAA AAGTTTTA-A TTCTAATTCC TTACGCAAGG
GATCGTTTGG
N1              AGATATTGTC A----GCAAG AGTTT----T ATAGTATTCC TTACGCAAGG
GATCGTTTGG
N2              AGATATTGTC A----GCAAG AGTTT----T ATAGTATTCC TTACGCAAGG
GATCGTTTGG
N3              AGATATTGTC A----GCAAG AGTTT----T ATAGTATTCC TTACGCAAGG
GATCGTTTGG
M1              AGATTTATAT -----TAAGT AGTTTTTTAT AATAAATTCC TTACGCAAGG
GATCGTTTGG
M2              AGATTTATAT -----TAAGT AGTTTTTTAT AATAAATTCC TTACGCAAGG
GATCGTTTGG
M3              AGATTTATAT -----TAAGT AGTTTTTTAT ATTAAATTCC TTACGCAAGG
GATCGTTTGG
M4              AGATTTATAT -----TAAGT AGTTTTTTAT ATTAAATTCC TTACGCAAGG
GATCGTTTGG
M5              AGATTTATAT -----TAAGT AGTTTTTTAT ATTAAATTCC TTACGCAAGG
GATCGTTTGG
M6              AGATTTATAT -----TAAGT AGTTTTTTAT ATTAAATTCC TTACGCAAGG
GATCGTTTGG
M7              AGATTTATTT -----tAAGT AGTTTTTTAT ATTAAATTCC TTACGCAAGG
GATCGTTTGG
M8              AGATTTATAT -----TAAGT AGTTTTTTAT ATTAAATTCC TTACGCAAGG
GATCGTTTGG
M9              AGATTTATAT -----TAAGT AGTTTTTTAT ATTAAATTCC TTACGCAAGG
GATCGTTTGG
M10             AGATTTATAT -----TAAGT AGTTTTTTAT AATAAATTCC TTACGCAAGG
GATCGTTTGG
M11             AGATTTATAT -----TAAGT AGTTTTTTAT ATTAAATTCC TTACGCAAGG
GATCGTTTGG
M12             AGATTTATAT -----TAAGT AGTTTTTTAT ATTAAATTCC TTACGCAAGG
GATCGTTTGG
```

```
M13           AGATTTATAT -----TAAGT AGTTTTTTAT AATAAATTCC TTACGCAAGG
GATCGTTTGG
M14           AGATTTATTT -----CAAGT AGTTTG---T AATAAATTCC TTACGCAAGG
GATCGTTTGG
M15           AGATTTATTT -----TAGGT AGTTTA---T AAAAAATTCC TTACGCAAGG
GATCGTTTGG
M16           AGATTTATTT -----TAGGT AGTTTA---T AAAAAATTCC TTACGCAAGG
GATCGTTTGG
M17           AGATTTATTT -----TAGGT AGTTTA---T AAAAAATTCC TTACGCAAGG
GATCGTTTGG
M18           AGATTTAATT -----TAAGT AGTTTT---T AATAAATTCC TTACGCAAGG
GATCGTTTGG
M19           AGATTTAATT -----TAAGT AGTTTT---T AATAAATTCC TTACGCAAGG
GATCGTTTGG
M20           AGATTTATTT C----TAAGT AGTTTT---T TATAATTTCC TTACGCAAGG
GATCGTTTGG
M21           AGATTTATTT T----TAAGT AGTTTT---T TATAATTTCC TTACGCAAGG
GATCGTTTGG
M22           AGATTTATTT T----TAAGT AGTTTT---T TATAATTTCC TTACGCAAGG
GATCGTTTGG
M23           AGATTTATTT T----TAAGT AGTTTT---T TATAATTTCC TTACGCAAGG
GATCGTTTGG
B1            AGATATAATA A----AAA-G TAAGTTTGAT TTTAATTTCC CTGCGCAAGG
GATCGTTTGG
B2            AGATATAATA A----AAAGG TATGTTTGTT TTTAATTTCC CTGCGCAAGG
GATCGTTTGG
B3            AGATATAATA A----AAAGG TATGTTTGTT TTTAATTTCC CTGCGCAAGG
GATCGTTTGG
B4            AGATATAATA A----AAAGG TATGTTTGTT TTTAATTTCC CTGCGCAAGG
GATCGTTTGG
B5            AGATATAATA A----AAAGG TATGTTTGTT TTTAATTTCC CTGCGCAAGG
GATCGTTTGG
B6            AGATATAATA A----AAAGG TATGTTTGTT TTTAATTTCC CTGCGCAAGG
GATCGTTTGG
B7            AGATATAATA A----AAAGG TAGGTTTGTT TTTAATTTCC CTGCGCAAGG
GATCGTTTGG
B8            AGATATAATA A----AAAGG TAGGTTTGTT TTTAATTTCC CTGCGCAAGG
GATCGTTTGG
B9            AGATATAATA A----AAAGG TATGTTTGTT TTTAATTTCC CTGCGCAAGG
GATCGTTTGG
B10           AGATATAATA A----AAAGG TATGTTTGTT TTTAATTTCC CTGCGCAAGG
GATCGTTTGG
B11           AGATATAATA A----AAAGG TATGTTTGTT TTTAATTTCC CTGCGCAAGG
GATCGTTTGG
B12           AGATATAATA A----AAAGG TATGTTTGTT TTTAATTTCC CTGCGCAAGG
GATCGTTTGG
B13           AGATATAATA A----AAAGG TAGGTTTGTT TTTAATTTCC CTGCGCAAGG
GATCGTTTGG
B14           AGATATAATA A----AAAGG TATGTTTGTT TTTAATTTCC CTGCGCAAGG
GATCGTTTGG
B15           AGATATAATA A----AAAGG TATGTTTGTT TTTAATTTCC CTGCGCAAGG
GATCGTTTGG
B16           AGATATAATA A----AAAGG TATGTTTGTT TTTAATTTCC CTGCGCAAGG
GATCGTTTGG
KF537632_Ddi  AGATTTTATT A-AATTGACA AGTTTTTT-T ATAATTTTCC TTACGCAAGG
GATCGTTTGG
AJ438961_Dde  AGCTTTATTT A----TGTTT AGTTTTATAG TTAGTAATCC CTGCGCAAGG
GATCGTTAGG
```

```
AJ438960_Dca    AGATTTTATT TGAATTGTTT GTTTTT---- ------ATCT CTGCGCAAGG
GATCGTTAGG
AJ438959_Dca    AGATTTTATT TGAATTGTTT GTTTTT---- ------ATCT CTGCGCAAGG
GATCGTTAGG
AJ438958_Dga    TAGTTTATTA TTATTTAATT ATTATG---- ATATTTTTCC CTACGCAAGG
GATCGTTAGG

                ....|....| ....|....| ....|....| ....|....| ....|....|
....|....|
                    1385       1395       1405       1415       1425
1435
D1              TTCTATGTAC GATGAAGGTC GGAGCTGTAT TCGATAAAAT GAGATGATTG
TATAAATTGT
D2              TTCTATGTAC GATGAAGGTC GGAGCTGTAT TCGATAAAAT GAGATGATTG
TATAAATTGT
D3              TTCTATGTAC GATGAAGGTC GGAGCTGTAT TCGATAAAAT GAGATGATTG
TATAAATTGT
R1              TTCTATGTAC GATGAAGGTC GAAACTGTAT TCGATAAAAT AGGATGAAAG
TAAAAATTGT
R2              TTCTATGTAC GATGAAGGTC GAAACTGTAT TCGATAAAAT AGGATGAAAG
TAAAAATTGT
R3              TTCTATGTAC GATGAAGGTC GAAACTGTAT TCGATAAAAT AGGATGAAAG
TAAAAATTGT
R4              TTCTATGTAC GATGAAGGTC GAAACTGTAT TCGATAAAAT AGGATGAATG
TAAAAATTGT
R5              TTCTATGTAC GATGAAGGTC GACACTGTAT TCGATAAAAT AAGATGAAAG
TAAAAATTGT
R6              TTCTATGTAC GATGAAGGTC GAAACTGTAT TCGATAAAAT AGGATGAACG
TAAAAAATGT
R7              TTCTATGTAC GATGAAGGTC GAAACTGTAT TCGATAAAAT AGGATGAACG
TAAAAAATGT
N1              TTCTATGTTC GAAGAAGGTC GGAGCAGTAT TCGATAATGT AAGATGATTG
TAAAAGTTGT
N2              TTCTATGTTC GAAGAAGGTC GGAGCAGTAT TCGATAATGT AAGATGATTG
TAAAAGTTGT
N3              TTCTATGTTC GAAGAAGGTC GGAGCAGTAT TCGATAATGT AAGATGATTG
TAAAAGTTGT
M1              TTCTATGTAC GATGAAGGTC GGAGCAGTAT TCGATAATAT AAGATGATTG
TTTAAATTGT
M2              TTCTATGTAC GATGAAGGTC GGAGCAGTAT TCGATAATAT AAAATGATTG
TTTAAATTGT
M3              TTCTATGTAC GATGAAGGTC GGAGCAGTAT TCGATAATAT AAAATGATTG
TTTAAATTGT
M4              TTCTATGTAC GATGAAGGTC GGAGCAGTAT TCGATAATAT AAAATGATTG
TTTAAATTGT
M5              TTCTATGTAC GATGAAGGTC GGAGCAGTAT TCGATAATAT AAAATGATTG
TTTAAATTGT
M6              TTCTATGTAC GATGAAGGTC GGAGCAGTAT TCGATAATAT AAAATGATTG
TTTAAATTGT
M7              TTCTATGTAC GATGAAGGTC GGAGCAGTAT TCGATAATAT AAAATGATTG
TTTAAATTGT
M8              TTCTATGTAC GATGAAGGTC GGAGCAGTAT TCGATAATAT AAAATGATTG
TTTAAATTGT
M9              TTCTATGTAC GATGAAGGTC GGAGCAGTAT TCGATAATAT AAAATGATTG
TTTAAATTGT
M10             TTCTATGTAC GATGAAGGTC GGAGCAGTAT TCGATAATAT AAAATGATTG
TTTAAATTGT
```

M11 TTCTATGTAC GATGAAGGTC GGAGCAGTAT TCGATAATAT AAGATGATTG
TTTAAATTGT
M12 TTCTATGTAC GATGAAGGTC GGAGCAGTAT TCGATAATAT AAGATGATTG
TTTAAATTGT
M13 TTCTATGTAC GATGAAGGTC GGAGCAGTAT TCGATAATAT AAAATGATTG
TTTAAATTGT
M14 CTCTATGTGC GATGAAGGTC GGAGCAGTAT TCGATAATAT AAGATGATTG
TATAAATTGT
M15 CTCTATGTGC GATGAAGGTC GGAGCAGTAT TCGATAATAT AAGATGATTG
TATAAATTGT
M16 CTCTATGTGC GATGAAGGTC GGAGCAGTAT TCGATAATAT AAGATGATTG
TATAAATTGT
M17 CTCTATGTGC GATGAAGGTC GGAGCAGTAT TCGATAATAT AAGATGATTG
TATAAATTGT
M18 CTCTATGTGC GATGAAGGTC GGAGCAGTAT TCGATAATAT AAGATGATTG
TATAAATTGT
M19 CTCTATGTGC GATGAAGGTC GGAACAGTAT TCGATAATAT AAGATGATTG
TATAAATTGT
M20 TTCTATGTAC GATGAAGGTC GAAGCAGTAT TCGATAATGT AAGATGGTTG
TATAAATTGT
M21 TTCTATGTAC GATGAAGGTC GAAGCAGTAT TCGATAATGT AAGATGGTTG
TATAAATTGT
M22 TTCTATGTAC GATGAAGGTC GGAGCAGTAT TCGATAATGT AAGATGGTTG
TATAAATTGT
M23 TTCTATGTAC GATGAAGGTC GAAGCAGTAT TCGATAATGT AAGATGGTTG
TATAAATTGT
B1 TTCTATGTGC GATGAAGGTC GGAGCAGTAT CTGATAATGT AGAATGATTG
TAATAATTGT
B2 TTCTATGTGC GATGAAGGTC GGAGCAGTAT CTGATAATGT AGAATGATTG
TAATAATTGT
B3 TTCTATGTGC GATGAAGGTC GGAGCAGTAT CTGATAATGT AGAATGATTG
TAATAATTGT
B4 TTCTATGTGC GATGAAGGTC GGAGCAGTAT CTGATAATGT AGAATGATTG
TAATAATTGT
B5 TTCTATGTGC GATGAAGGTC GGAGCAGTAT CTGATAATGT AGAATGATTG
TAATAATTGT
B6 TTCTATGTGC GATGAAGGTC GGAGCAGTAT CTGATAATGT AGAATGATTG
TAATAATTGT
B7 TTCTATGTGC GATGAAGGTC GGAGCAGTAT CTGATAATGT AGAATGATTG
TAATAATTGT
B8 TTCTATGTGC GATGAAGGTC GGAGCAGTAT CTGATAATGT AGAATGATTG
TAATAATTGT
B9 TTCTATGTGC GATGAAGGTC GGAGCAGTAT CTGATAATGT AGAATGATTG
TAATAATTGT
B10 TTCTATGTGC GATGAAGGTC GGAGCAGTAT CTGATAATGT AGAATGATTG
TAATAATTGT
B11 TTCTATGTGC GATGAAGGTC GGAGCAGTAT CTGATAATGT AGAATGATTG
TAATAATTGT
B12 TTCTATGTGC GATGAAGGTC GGAGCAGTAT CTGATAATGT AGAATGATTG
TAATAATTGT
B13 TTCTATGTGC GATGAAGGTC GGAGCAGTAT CTGATAATGT AGAATGATTG
TAATAATTGT
B14 TTCTATGTGC GATGAAGGTC GGAGCAGTAT CTGATAATGT AGAATGATTG
TAATAATTGT
B15 TTCTATGTGC GATGAAGGTC GGAGCAGTAT CTGATAATGT AGAATGATTG
TAATAATTGT
B16 TTCTATGTGC GATGAAGGTC GGAGCAGTAT CTGATAATGT AGAATGATTG
TAATAATTGT

KF537632_Ddi   TTCTATGTAC GATGAAGGTC GGAGCAGTAT CTGATAATAT TAAATGATTG TA-
AAATTGT
AJ438961_Dde   CTCTATGTAC GATGAAGGCC GGGGCAGCAA CCGATATGGC GCAATGTATG
CAGATATCGT
AJ438960_Dca   TTCTATGTAC GACGAAGGCC GGAGTAGTAT CCGATATGGT GCTACAAGCG
CAGTTATTGT
AJ438959_Dca   TTCTATGTAC GATGAAGGTC GGAGTGGTAT CCGATATGGT GCTACAAGCG
CAGTTATTGT
AJ438958_Dga   TTCTGTGTTC GATGAAGACC GGGGCAGTAT CCGATACTGC ACAATGGATG CA-
-TGGGGT

....|....| ....|....| ....|....| ....|....| ....|....|
....|....|
1445 1455 1465 1475 1485
1495
D1 CTTATATTAC TGAATAGAGT A-GCGATGCT CGAATACTCC TT-GAA????
??????????
D2 CTTATATTAC TGAATAGAGT A-GCGATGCT CGAATACTCC TTTGAATTAA
GCATATGAGT
D3 CTTATATTAC TGAATAGAGT A-GCGATGCT CGAATACTCC TTTGAATTAA
GCATATGAGT
R1 CCTATATTAC TGAATAGAGT A-GCGATGCT CGAGTACTCC TTTGAATTAA
GCATATGAGT
R2 CCTATGTTAC TGAATAGAGT A-GCGATGCT CGAGTACTCC TTTGAATTAA
GCATATGAGT
R3 CCTATGTTAC TGAATAGAGT A-GCGATGCT CGAGTACTCC TTTGAATTAA
GCATATGAGT
R4 CCTATGTTAC TGAATAGAGT A-GCGATGCT CGGGTACTCC TTTGAATTAA
GCATATGAGT
R5 CTTATATTAC TGAATAGAGT A-GCGATGCT CGAATACTCC TTTGAATTAA
GCATATGAGT
R6 CCTATATTAC TGAATAGAGT A-GCGATGCT CGAATACTCC TTTGAATTAA
GCATATGAGT
R7 CCTATATTAC TGAATAGAGT A-GCGATGCT CGAATACTCC TTTGAATTAA
GCATATGAGT
N1 CTTATGTGAC TGAATTGAGT ACAAAATGCT CGGGTACTCC TTTGAATTAA
GCATATGAGT
N2 CTTATGTGAC TGAATTGAGT ACAAAATGCT CGGGTACTCC TTTGAATTAA
GCATATGAGT
N3 CTTATGTGAC TGAATTGAGT ACAAAATGCT CGGGTACTCC TTTGAATTAA
GCATATGAGT
M1 TTTATGTTAC TGAATAAAGT A-GTGATGCT CGATTACTCC TTTGAATTAA
GCATATGAGT
M2 TTTATGTTAC TGAATAGAGT A-GTGATGCT CGATTACTCC TTTGAATTAA
GCATATGAGT
M3 TTTATGTTAC TGAATAGAGT A-GTGATGCT CGATTACTCC TTTGAATTAA
GCATATGAGT
M4 TTTATGTTAC TGAATAGAGT A-GTGATGCT CGATTACTCC TTTGAATTAA
GCATATGAGT
M5 TTTATGTTAC TGAATAGAGT A-GTGATGCT CGATTACTCC TTTGAATTAA
GCATATGAGT
M6 TTTATGTTAC TGAATAGAGT A-GTGATGCT CGATTACTCC TTTGAATTAA
GCATATGAGT
M7 TTTATGTTAC TGAATAGAGT A-GTGATGCT CGATTACTCC TTTGAATTAA
GCATATGAGT
M8 TTTATGTTAC TGAATAGAGT A-GTGATGCT CGATTACTCC TTTGAATTAA
GCATATGAGT

M9 TTTATGTTAC TGAATAGAGT A-GTGATGCT CGATTACTCC TTTGAATTAA
GCATATGAGT
M10 TTTATGTTAC TGAATAGAGT A-GTGATGCT CGATTACTCC TTTGAATTAA
GCATATGAGT
M11 TTTATGTTAC TGAATAGAGT A-GTGATGCT CGATTACTCC TTTGAATTAA
GCATATGAGT
M12 TTTATGTTAC TGAATAGAGT A-GTGATGCT CGATTACTCC TTTGAATTAA
GCATATGAGT
M13 TTTATGTTAC TGAATAGAGT A-GTGATGCT CGATTACTCC TTTGAATTAA
GCATATGAGT
M14 CTTATGTTAC TGAATAGAGT A-GTGATGCT CGATTACTCC TTTGAATTAA
GCATA?????
M15 CTTATGTTAC TGAATAGAGT A-GTGATGCT CGATTACTCC TTTGAATTAA
GCATATGAGT
M16 CTTATGTTAC TGAATAGAGT A-GTGATGCT CGATTACTCC TTTGAATTAA
GCATATGAGT
M17 CTTATGTTAC TGAATAGAGT A-GTGATGCT CGATTACTCC TTTGAATTAA
GCATATGAGT
M18 CTTATGTTAC TGAATAGAGT A-GTGATGCT CGATTACTCC TTTGAATTAA
GCATATGAGT
M19 CTTATGTTTC TGAATAGAGT A-GTGATGCT CGATTACTCC TTTGAATTAA
GCATATGAGT
M20 CTTATGTTAC TGAATAGAGT A-GTGATGCT CGAATACTCC TTTGAATTAA
GCATATGAGT
M21 CTTATGTTAC TGAATAGAGT A-GTGATGCT CGAATACTCC TTTGAATTAA
GCATATGAGT
M22 CTTATGTTAC TGAATAGAGT A-GTGATGCT CGAATACTCC TTTGAATTAA
GCATATGAGT
M23 CTTATGTTAC TGAATAGAGT A-GTGATGCT CGAATACTCC TTTGAATTAA
GCATATGAGT
B1 TCTATGTGAC TGAATTGAAC A-GTGATGTT CGGGTACTCC TT-GAATAA?
??????????
B2 TCTATGTGAC TGAATTGAAC A-GTGATGTT CGGGTACTCC TTTGAATTAA
GCATATGAGT
B3 TCTATGTGAC TGAATTGAAC A-GTGATGTT CGGGTACTCC TTTGAATTAA
GCATATGAGT
B4 TCTATGTGAC TGAATTGAAC A-GTGATGTT CGGGTACTCC TTTGAATTAA
GCATATGAGT
B5 TCTATGTGAC TGAATTGAAC A-GTGATGTT CGGGTACTCC TTTGAATTAA
GCATATGAGT
B6 TCTATGTGAC TGAATTGAAC A-GTGATGTT CGGGTACTCC TTTGAATTAA
GCATATGAGT
B7 TCTATGTGAC TGAATTGAAC A-GTGATGTT CGGGTACTCC TTTGAATTAA
GCATATGAGT
B8 TCTATGTGAC TGAATTGAAC A-GTGATGTT CGGGTACTCC TTTGAATTAA
GCATATGAGT
B9 TCTATGTGAC TGAATTGAAC A-GTGATGTT CGGGTACTCC TTTGAATTAA
GCATATGAGT
B10 TCTATGTGAC TGAATTGAAC A-GTGATGTT CGGGTACTCC TTTGAATTAA
GCATATGAGT
B11 TCTATGTGAC TGAATTGAAC A-GTGATGTT CGGGTACTCC TTTGAATTAA
GCATATGAGT
B12 TCTATGTGAC TGAATTGAAC A-GTGATGTT CGGGTACTCC TTTGAATTAA
GCATATGAGT
B13 TCTATGTGAC TGAATTGAAC A-GTGATGTT CGGGTACTCC TTTGAATTAA
GCATATGAGT
B14 TCTATGTGAC TGAATTGAAC A-GTGATGTT CGGGTACTCC TTTGAATTAA
GCATATGAGT

```
B15             TCTATGTGAC TGAATTGAAC A-GTGATGTT CGGGTACTCC TTTGAATTAA
GCATATGAGT
B16             TCTATGTGAC TGAATTGAAC A-GTGATGTT CGGGTACTCC TTTGAATTAA
GCATATGAGT
KF537632_Ddi    TTAATGTTAC TGAATTAAGG TTAAAAACCT TGAATACTCC TTTGAATTAA
GCATATGAGT
AJ438961_Dde    TGTGCGTGGC CGAATTGAAC A-GAGATGTT CGGATACTCC TTTGAATTAA
GCA???????
AJ438960_Dca    TGCACGTTGC CTAATTGAAC A-GTGATGTT CGAATACTCC TTCTAATTAA
GCA???????
AJ438959_Dca    TGCACGTTGC CTAATTGAAC A-ATGATGTT CGAATACTCC TTTGAATTAA
GCA???????
AJ438958_Dga    TGTGCATTG- TTGAATGAAC A-GAGATGTT CGAGTACTCC TTTGAATTAA
GCA???????

                ....|....| ....|....| ....|....| ....|....| ....|....|
....|....|
                   1505       1515       1525       1535       1545
1555
D1              ?????????? ?????????? ?????????? ?????????? ??????????
??????????
D2              AAAGGAAGGA AAAGGAACTA ACAAGGATTC TCTTATTAGT GGCGAATGAA
CAGAGAAGAG
D3              AAAGGAAGGA AAAGGAACTA ACAAGGATTC TCTTATTAGT GGCGAATGAA
CAGAGAAGAG
R1              AAAGGAAGGA GAAGAAACTA ACAAGGATTC TCTTATTAGT GGCGAATGAA
CAGAGAAGAG
R2              AAAGGAAGGA GAAGAAACTA ACAAGGATTC TCTTATTAGT GGCGAATGAA
CAGAGAAGAG
R3              AAAGGAAGGA GAAGAAACTA ACAAGGATTC TCTTATTAGT GGCGAATGAA
CAGAGAAGAG
R4              AAAGGAAGGA GAAGAAACTA ACAAGGATTC TCTTATTAGT GGCGAATGAA
CAGAGAAGAG
R5              AAAGGAAGGA GAAGAAACTA ACAAGGATTC TCTTATTAGT GGCGAATGAA
CAGAGAATAG
R6              AAAGGAAGGA GAAGAAACTA ACAAGGATTC TCTTATTAGT GGCGAATGAA
CAGAGAACAG
R7              AAAGGAAGGA GAAGAAACTA ACAAGGATTC TCTTATTAGT GGCGAATGAA
CAGAGAACAG
N1              AAAGGAAGGA AAAGAAACTA ACAAGGATTC TCTTATTAGT GGCGAATGAA
CAGAGAAGAG
N2              AAAGGAAGGA AAAGAAACTA ACAAGGATTC TCTTATTAGT GGCGAATGAA
CAGAGAAGAG
N3              AAAGGAAGGA AAAGAAACTA ACAAGGATTC TCTTATTAGT GGCGAATGAA
CAGAGAAGAG
M1              AAAGGAGG?? ?????????? ?????????? ?????????? ??????????
??????????
M2              AAAGGAAGGA AAAGAAACTA ACAAGGATTC TCTTATTAGT GGCGAATGAA
CAGAGAAGAG
M3              AAAGGAAGGA AAAGAAACTA ACAAGGATTC TCTTATTAGT GGCGAATGAA
CAGAGAAGAG
M4              AAAGGAAGGA AAAGAAACTA ACAAGGATTC TCTTATTAGT GGCGAATGAA
CAGAGAAGAG
M5              AAAGGAAGGA GAAGAAACTA ACAAGGATTC TCTTATTAGT GGCGAATGAA
CAGAGAAGAG
M6              AAAGGAAGGA GAAGAAACTA ACAAGGATTC TCTTATTAGT GGCGAATGAA
CAGAGAAGAG
```

M7 AAAGGAAGGA AAAGAAACTA ACAAGGATTC TCTTATTAGT GGCGAATGAA
CAGAGAAGAG
M8 AAAGGAAGGA AAAGAAACTA ACAAGGATTC TCTTATTAGT GGCGAATGAA
CAGAGAAGAG
M9 AAAGGAAGGA AAAGAAACTA ACAAGGATTC TCTTATTAGT GGCGAATGAA
CAGAGAAGAG
M10 AAAGGAAGGA AAAGAAACTA ACAAGGATTC TCTTATTAGT GGCGAATGAA
CAGAGAAGAG
M11 AAAGGAAGGA AAAGAAACTA ACAAGGATTC TCTTATTAGT GGCGAATGAA
CAGAGAAGAG
M12 AAAGGAAGGA AAAGAAACTA ACAAGGATTC TCTTATTAGT GGCGAATGAA
CAGAGAAGAG
M13 AAAGGAAGGA AAAGAAACTA ACAAGGATTC TCTTATTAGT GGCGAATGAA
CAGAGAAGAG
M14 ?????????? ?????????? ?????????? ?????????? ??????????
??????????
M15 AAAGGAAGGA AAAGAAACCA ACGAGGATTC TCTTATTAGT GGCGAATGAA
CAGAGAACAG
M16 AAAGGAAGGA AAAGAAACCA ACGAGGATTC TCTTATTAGT GGCGAATGAA
CAGAGAACAG
M17 AAAGGAAGGA AAAGAAACCA ACGAGGATTC TCTTATTAGT GGCGAATGAA
CAGAGAACAG
M18 AAAGGA-GGA GAAGAAACTA ACAAGGATTC TCTTATTAGT GGCGAATGAA
CAGAGAACAG
M19 AAAGGAAGGA GAAGAAACTA ACAAGGATTC TCTTATTAGT GGCGAATGAA
CAGAGAAGAG
M20 AAAGGAAGGA GAAGAAACTA ACAAGGATTC TCTTATTAGT GGCGAATGAA
CAGAGAATAG
M21 AAAGGAAGGA GAAGAAACTA ACAAGGATTC TCTTATTAGT GGCGAATGAA
CAGAGAATAG
M22 AAAGGAAGGA GAAGAAACTA ACAAGGATTC TCTTATTAGT GGCGAATGAA
CAGAGAATAG
M23 AAAGGAAGGA GAAGAAACTA ACAAGGATTC TCTTATTAGT GGCGAATGAA
CAGAGAATAG
B1 ?????????? ?????????? ?????????? ?????????? ??????????
??????????
B2 AAAGGAAGGA AAAGAAACTA ACTAGGATTC TCTTATTAGT GGCGAATGAA
CAGAGAATAG
B3 AAAGGAAGGA AAAGAAACTA ACTAGGATTC TCTTATTAGT GGCGAATGAA
CAGAGAATAG
B4 AAAGGAAGGA AAAGAAACTA ACTAGGATTC TCTTATTAGT GGCGAATGAA
CAGAGAATAG
B5 AAAGGAAGGA AAAGAAACTA ACTAGGATTC TCTTATTAGT GGCGAATGAA
CAGAGAATAG
B6 AAAGGAAGGA AAAGAAACTA ACTAGGATTC TCTTATTAGT GGCGAATGAA
CAGAGAATAG
B7 AAAGGAAGGA AAAGAAACTA ACTAGGATTC TCTTATTAGT GGCGAATGAA
CAGAGAATAG
B8 AAAGGAAGGA AAAGAAACTA ACTAGGATTC TCTTATTAGT GGCGAATGAA
CAGAGAATAG
B9 AAAGGAAGGA AAAGAAACTA ACTAGGATTC TCTTATTAGT GGCGAATGAA
CAGAGAATAG
B10 AAAGGAAGGA AAAGAAACTA ACTAGGATTC TCTTATTAGT GGCGAATGAA
CAGAGAATAG
B11 AAAGGAAGGA AAAGAAACTA ACTAGGATTC TCTTATTAGT GGCGAATGAA
CAGAGAATAG
B12 AAAGGAAGGA AAAGAAACTA ACTAGGATTC TCTTATTAGT GGCGAATGAA
CAGAGAATAG

```
B13            AAAGGAAGGA AAAGAAACTA ACTAGGATTC TCTTATTAGT GGCGAATGAA
CAGAGAATAG
B14            AAAGGAAGGA AAAGAAACTA ACTAGGATTC TCTTATTAGT GGCGAATGAA
CAGAGAATAG
B15            AAAGGAAGGA AAAGAAACTA ACTAGGATTC TCTTATTAGT GGCGAATGAA
CAGAGAATAG
B16            AAAGGAAGGA AAAGAAACTA ACTAGGATTC TCTTATTAGT GGCGAATGAA
CAGAGAATAG
KF537632_Ddi   AAAGGAAGGA AAAGAAACTA ACAAGGATTC TCTTATTAGT GGCGAATGAA
CAGAGAAGAG
AJ438961_Dde   ?????????? ?????????? ?????????? ?????????? ??????????
??????????
AJ438960_Dca   ?????????? ?????????? ?????????? ?????????? ??????????
??????????
AJ438959_Dca   ?????????? ?????????? ?????????? ?????????? ??????????
??????????
AJ438958_Dga   ?????????? ?????????? ?????????? ?????????? ??????????
??????????

               ....|....| ....|....| ....|....| ....|....| ....|....|
....|....|
                  1565       1575       1585       1595       1605
1615
D1             ?????????? ?????????? ?????????? ?????????? ??????????
??????????
D2             CCCTAAGTGT AATCAAATTC TCTGAAT--T TGAATTGTCA GGCAT-AGAG
GATGCGAATA
D3             CCCTAAGTGT AATCAAATTC TCTGAAT--T TGAATTGTCA GGCAT-GGAG
GATGCGAATA
R1             CCCTAAGTGT AATCAAATTC TTGTAAT--T TGAGATGTCA GGCATTAGAT
AGTGTGAATA
R2             CCCTAAGTGT AATCAAATTC TTGCAAT--T TGAGATGTCA GGCATTAGAT
AGTGTGAATA
R3             CCCTAAGTGT AATCAAATTC TTGCAAT--T TGAGATGTCA GGCATTAGAT
AGTGTGAATA
R4             CCCTAAGTGT AATCAAATTC TTGCAAT--T TGAGATGTCA GGCATTAGGT
AGTGTGAATA
R5             CCCTAAGTGT AATCAAATTC TAGCAAT--T TGAGATGTCA GGCATTGAAT
AGTGTGAATA
R6             CCCTAAGTGT AATCAAATTC ATGTAAT--T TGAGATGTCA GGCATTGAAT
AGTGTGAATA
R7             CCCTAAGTGT AATCAAATTC ATGTAAT--T TGAGATGTCA GGCATTGAAT
AGTGTGAATA
N1             CCCTAAGTGT AATCAAATTC TTGTAAT--T TGAGATGTCT AGTATTATAC
AGTGTGAATA
N2             CCCTAAGTGT AATCAAATTC TTGTAAT--T TGAGATGTCT AGTATTATAT
AGTGTGAATA
N3             CCCTAAGTGT AATCAAATTC TTGTAAT--T TGAGATGTCA GGTATTATAT
AGTGTGAATA
M1             ?????????? ?????????? ?????????? ?????????? ??????????
??????????
M2             CCCTAAGTGT AATCAAATTC TATGAAT--T TGAGATGTCA GGTATAATGT
GGTGTGAATG
M3             CCCTAAGTGT AATCAAATTC TATGAAT--T TGAGATGTCA GGTATAATGT
GGTGTGAATG
M4             CCCTAAGTGT AATCAAATTC TATGAAT--T TGAGATGTCA GGTATAATGT
GGTGTGAATG
```

M5 CCCTAAGTGT AATCAAATTC TATGAAT--T TGAGATGTCA GGTATAATGT
GGTGTGAATG
M6 CCCTAAGTGT AATCAAATTC TATGAAT--T TGAGATGTCA GGTATAAATT
GGTGTGAATG
M7 CCCTAAGTGT AATCAAATTC TATGAAT--T TGAGATGTCA GGTATAAAGT
GGTGTGAATG
M8 CCCTAAGTGT AATCAAATTC TATGAAT--T TGAGATGTCA GGTATAAAGT
GGTGTGAATG
M9 CCCTAAGTGT AATCAAATTC TATGAAT--T TGAGATGTCA GGTATAAAGT
GGTGTGAATG
M10 CCCTAAGTGT AATCAAATTC TATGAAT--T TGAGATGTCA GGTATAAATT
GGTGTGAATG
M11 CCCTAAGTGT AATCAAATTC TATGAAT--T TGAGATGTCA GGTATAATGT
GGTGTGAATG
M12 CCCTAAGTGT AATCAAATTC TATGAAT--T TGAGATGTCA GGTATAATGT
GGTGTGAATG
M13 CCCTAAGTGT AATCAAATTC TATGAAT--T TGAGATGTCA GGTATCATGT
GGTGTGAATG
M14 ?????????? ?????????? ?????????? ?????????? ??????????
??????????
M15 CCCTAAGTGT AATCAAATTC GTTGAAT--T TGAGATGTCA GGTATCATGT
GGTGTGAATG
M16 CCCTAAGTGT AATCAAATTC GTTGAAT--T TGAGATGTCA GGTATCATGT
GGTGTGAATG
M17 CCCTAAGTGT AATCAAATTC GTTGAAT--T TGAGATGTCA GGTATCATGT
GGTGTGAATG
M18 CCCTAAGTGT AATCAAATTC TATGAAT--T TGAGATGTCA GGTATCATGT
GGTGTGAATG
M19 CCCTAAGTGT AATCAAATTC TAAGAAT--T TGAGATGTCA GGTATTATGT
GGTGTGAATG
M20 CCCTAAGTGT AATCAAATTC ATTGAAT--T TGAGATGTCA GGTATCATGT
AGTGTGAATA
M21 CCCTAAGTGT AATCAAATTC ATTGAAT--T TGAGATGTCA GGTATCATGT
AGTGTGAATA
M22 CCCTAAGTGT AATCAAATTC ATTGAAT--T TGAGATGTCA GGTATCATGT
AGTGTGAATA
M23 CCCTAAGTGT AATCAAATTC ATTGAAT--T TGAGATGTCA GGTATCATGT
AGTGTGAATA
B1 ?????????? ?????????? ?????????? ?????????? ??????????
??????????
B2 CCC-AAGTGT AATCAATAAT AAATAATTAT TGAGATGTCT AGTAT--ATT
GACGTGAAAA
B3 CCC-AAGTGT AATCAATAAT AAATAATTAT TGAGATGTCT AGTAT--ATT
GACGTGAAAA
B4 CCC-AAGTGT AATCAATAAT AAATAATTAT TGAGATGTCT AGTAT--ATT
GACGTGAAAA
B5 CCC-AAGTGT AATCAATAAT AAATAATTAT TGAGATGTCT AGTAT--ATT
GACGTGAAAA
B6 CCC-AAGTGT AATCAATAAT AAATAATTAT TGAGATGTCT AGTAT--ATT
GACGTGAAAA
B7 CCC-AAGTGT AATCAATAAT AAATAATTAT TGAGATGTCT AGTAT--ATT
GACGTGAAAA
B8 CCC-AAGTGT AATCAATAAT AAATAATTAT TGAGATGTCT AGTAT--ATT
GACGTGAAAA
B9 CCC-AAGTGT AATCAATAAT AAATAATTAT TGAGATGTCT AGTAT--ATT
GACGTGAAAA
B10 CCC-AAGTGT AATCAATAAT AAATAATTAT TGAGATGTCT AGTAT--ATT
GACGTGAAAA

B11 CCC-AAGTGT AATCAATAAT AAATAATTAT TGAGATGTCT AGTAT--ATT
GACGTGAAAA
B12 CCC-AAGTGT AATCAATAAT AAATAATTAT TGAGATGTCT AGTAT--ATT
GACGTGAAAA
B13 CCC-AAGTGT AATCAATAAT AAATAATTAT TGAGATGTCT AGTAT--ATT
GACGTGAAAA
B14 CCC-AAGTGT AATCAATAAT AAATAATTAT TGAGATGTCT AGTAT--ATT
GACGTGAAAA
B15 CCC-AAGTGT AATCAATAAT AAATAATTAT TGAGATGTCT AGTAT--ATT
GACGTGAAAA
B16 CCC-AAGTGT AATCAATAAT AAATAATTAT TGAGATGTCT AGTAT--ATT
GACGTGAAAA
KF537632_Ddi CCC-CAGTGT AATCACTTTA AATTTATAAG TGAGTTGTCT AGCAG-ATAG
GACGTGAAAA
AJ438961_Dde ?????????? ?????????? ?????????? ?????????? ??????????
??????????
AJ438960_Dca ?????????? ?????????? ?????????? ?????????? ??????????
??????????
AJ438959_Dca ?????????? ?????????? ?????????? ?????????? ??????????
??????????
AJ438958_Dga ?????????? ?????????? ?????????? ?????????? ??????????
??????????

....|....| ....|....| ....|....| ....|....| ....|....|
....|....|
1625 1635 1645 1655 1665
1675
D1 ?????????? ?????????? ?????????? ?????????? ??????????
??????????
D2 TATTGGAAAA TATAGCCATA GAGAGTTAAA GCCTCGTAGC A--TCTTAAA
TTAGGGATGA
D3 TATTGGAAAA TATAGCCATA GAGAGTTAAA GCCTCGTAGC A--TCTTAAA
TTAGGGATGA
R1 TACTGGAAAG TATAGTCGTA GAGAGTTATA GCCTCGTAGC A--CTATATG
ATAGGGATGA
R2 TACTGGAAAG TATTGCCGTA GAGAGTTATA GCCTCGTAGC A--CTATATG
ATAGGGATGA
R3 TACTGGAAAG TATTGCCGTA GAGAGTTATA GCCTCGTAGC A--CTATATG
ATAGGGATGA
R4 TACTGGAAAG TATAGCCGTA GAGAGTTATA GCCTCGTAGC A--CTATATG
ATAGGGATGA
R5 TACTGGAAAG TATAGCCAAA GAGAGTTATA GCCTCGTAAC A--CTATATG
ATAGGGATGA
R6 TACTGGAAAG TATAGCCCAA GAGAGTTATA GCCTCGTAGC A--CTATATG
ATAGGGATGA
R7 TACTGGAAAG TATAGCCCAA GAGAGTTATA GCCTCGTAGC A--CTATATG
ATAGGGATGA
N1 TGCTGGAAAG CATTGCCGTA GAGAGTTATA GCCTCGTAGC A--CTA-GTT
ATAGGGATGA
N2 TGCTGGAAAG CATTGCCGTA GAGAGTTATA GCCTCGTAGC A--CTA-GTT
ATAGGGATGA
N3 TGCTGGAAAG CATTGCCGTA GAGAGTTATA GCCTCGTAGC A--CTA-GTT
ATAGGGATGA
M1 ?????????? ?????????? ?????????? ?????????? ??????????
??????????
M2 TATTGGAAAA TACAGCCTAA GAGAGTTATA GCCTCGTAAC A--CCATTTT
GTAGGAATGA

M3 TATTGGAAAA TACAGCCTAA GAGAGTTATA GCCTCGTAAC A--CCATTTT
GTAGGAATGA
M4 TATTGGAAAA TACAGCCTAA GAGAGTTATA GCCTCGTAAC A--CCATTTT
GTAGGAATGA
M5 TATTGGAAAA TACAGCCTAA GAGAGTTATA GCCTCGTAAC A--CCATTTT
GTAGGAATGA
M6 TATTGGAAAA TACAGCCTAA GAGAGTTATA GCCTCGTAAC A--CCATTTT
GTAGGAATGA
M7 TATTGGAAAA TACAGCCTAA GAGAGTTATA GCCTCGTAAC A--CCATTTT
GTAGGAATGA
M8 TATTGGAAAA TACAGCCTAA GAGAGTTATA GCCTCGTAAC A--CCATTTT
GTAGGAATGA
M9 TATTGGAAAA TACAGCCTAA GAGAGTTATA GCCTCGTAAC A--CCATTTT
GTAGGAATGA
M10 TATTGGAAAA TACAGCCTAA GAGAGTTATA GCCTCGTAAC A--CCATTTT
GTAGGAATGA
M11 TATTGGAAAA TACAGCCTAA GAGAGTTATA GCCTCGTAAC A--CCATTTT
GTAGGAATGA
M12 TATTGGAAAA TACAGCCTAA GAGAGTTATA GCCTCGTAAC A--CCATTTT
GTAGGAATGA
M13 TATTGGAAAA TACAGCCTAA GAGAGTTATA GCCTCGTAAC A--CCATTTT
GTAGGAATGA
M14 ?????????? ?????????? ?????????? ?????????? ??????????
??????????
M15 TACTGGAAAG TACTGCCGAA GAGAGTTATA GCCTCGTAGC A--CCATTTT
TTAGGTATGA
M16 TACTGGAAAG TACTGCCGAA GAGAGTTATA GCCTCGTAGC A--CCATTTT
TTAGGTATGA
M17 TACTGGAAAG TACTGCCGAA GAGAGTTATA GCCTCGTAGC A--CCATTTT
TTAGGTATGA
M18 TACTGGAAAG TACTGCCGAA GAGAGTTATA GCCTCGTAAC A--CCATTTT
GTAGGTATGA
M19 TACTGGAAAG TACTGCCGAA GAGAGTTATA GCCTCGTAAC A--CCATTTT
GTAGGTATGA
M20 TACTGGAAAG TATTGCCAGA GAGAGTTATA GCCTCGTAGC A--CTATTTT
GTAGGGATGA
M21 TACTGGAAAG TATTGCCAGA GAGAGTTATA GCCTCGTAGC A--CTATTTT
GTAGGGATGA
M22 TACTGGAAAG TATTGCCAGA GAGAGTTATA GCCTCGTAGC A--CTATTTT
GTAGGGATGA
M23 TACTGGAAAG TATTGCCAGA GAGAGTTATA GCCTCGTAGC A--CTATTTT
GTAGGGATGA
B1 ?????????? ?????????? ?????????? ?????????? ??????????
??????????
B2 CAATGGAAAG TGTTGCCGTA GAGAGTTATA GCCTCGTAGC GT-CTAAATT
TAAAAAGGGA
B3 CAATGGAAAG TGTTGTCGTA GAGAGTTATA GCCTCGTAGC GT-CTAAATT
TAAAAAGGGA
B4 CAATGGAAAG TGTTGCCGTA GAGAGTTATA GCCTCGTAGC GT-CTAAATT
TAAAAAGGGA
B5 CAATGGAAAG TGTTGCCGTA GAGAGTTATA GCCTCGTAGC GT-CTAAATT
TAATAAGGGA
B6 CAATGGAAAG TGTTGCCGTA GAGAGTTATA GCCTCGTAGC GT-CTAAATT
TAAAAAGGGA
B7 CAATGGAAAG TGTTGCCGTA GAGAGTTATA GCCTCGTAGC GT-CTAAATT
TAAAAAGGGA
B8 CAATGGAAAG TGTTGCCGTA GAGAGTTATA GCCTCGTAGC GT-CTAAATT
TAAAAAGGGA

B9 CAATGGAAAG TGTTGCCGTA GAGAGTTATA GCCTCGTAGC GT-CTAAATT
TAAAAAGGGA
B10 CAATGGAAAG TGTTGCCGTA GAGAGTTATA GCCTCGTAGC GT-CTAAATT
TAATAAGGGA
B11 CAATGGAAAG TGTTGCCGTA GAGAGTTATA GCCTCGTAGC GT-CTAAATT
TAAAAAGGGA
B12 CAATGGAAAG TGTTGCCGTA GAGAGTTATA GCCTCGTAGC GT-CTAAATT
TAAAAAGGGA
B13 CAATGGAAAG TGTTGCCGTA GAGAGTTATA GCCTCGTAGC GT-CTAAATT
TAAAAAGGGA
B14 CAATGGAAAG TGTTGCCGTA GAGAGTTATA GCCTCGTAGC GT-CTAAATT
TAAAAAGGGA
B15 CAATGGAAAG TGTTGCCGTA GAGAGTTATA GCCTCGTAGC GT-CTAAATT
TAAAAAGGGA
B16 CAATGGAAAG TGTTGCCGTA GAGAGTTATA GCCTCGTAGC GT-CTAAATT
TAAAAAGGGA
KF537632_Ddi TAATGGAAAG TATTGCCGCA GAGAGTGATA GCCTCGTAAC GTCTAATGAA
TTGGGATTGA
AJ438961_Dde ?????????? ?????????? ?????????? ?????????? ??????????
??????????
AJ438960_Dca ?????????? ?????????? ?????????? ?????????? ??????????
??????????
AJ438959_Dca ?????????? ?????????? ?????????? ?????????? ??????????
??????????
AJ438958_Dga ?????????? ?????????? ?????????? ?????????? ??????????
??????????

....|....| ....|....| ....|....| ....|....| ....|....|
....|....|
1685 1695 1705 1715 1725
1735
D1 ?????????? ?????????? ?????????? ?????????? ??????????
??????????
D2 GTAGTTGTGC TCGGTAATGC ACAATGAAAT AGGTGGTAGT GTCCATCTAA
GGCTAAATAT
D3 GTAGTTGTGC TCGGTAATGC ACAATGAAAT AGGTGGTAGT GTCCATCTAA
GGCTAAATAT
R1 GTAGTTGTGC TCGGTAATGC ACAATGAAAT AGGTGGTAGT GTCCATCTAA
GGCTAAATAT
R2 GTAGTTGTGC TCGGTAATGC ACAATGAAAT AGGTGGTAGT GTCCATCTAA
GGCTAAATAT
R3 GTAGTTGTGC TCGGTAATGC ACAATGAAAT AGGTGGTAGT GTCCATCTAA
GGCTAAATAT
R4 GTAGTTGTGC TCGGTAATGC ACAATGAAAT AGGTGGTAGT GTCCATCTAA
GGCTAAATAT
R5 GTAGTTGTGC TCGGTAATGC ACAATGAAAT AGGTGGTAGT GTCCATCTAA
GGCTAAATAT
R6 GTAGTTGTGC TCGGTAATGC ACAATGAAAT AGGTGGTAGT GTCCATCTAA
GGCTAAATAT
R7 GTAGTTGTGC TCGGTAATGC ACAATGAAAT AGGTGGTAGT GTCCATCTAA
GGCTAAATAT
N1 GTAGTTGTGC TCGGTAATGC ACAATGAAAT AGGTGGTAGT GTCCATCTAA
GGCTAAATAT
N2 GTAGTTGTGC TCGGTAATGC ACAATGAAAT AGGTGGTAGT GTCCATCTAA
GGCTAAATAT
N3 GTAGTTGTGC TCGGTAATGC ACAATGAAAT AGGTGGTAGT GTCCATCTAA
GGCTAAATAT

M1 ?????????? ?????????? ?????????? ?????????? ??????????
??????????
M2 GTAGTTGTGC TCGGTAATGC ACAATGAAAT AGGTGGTAGT GTCCATCTAA
GGCTAAATAT
M3 GTAGTTGTGC TCGGTAATGC ACAATGAAAT AGGTGGTAGT GTCCATCTAA
GGCTAAATAT
M4 GTAGTTGTGC TCGGTAATGC ACAATGAAAT AGGTGGTAGT GTCCATCTAA
GGCTAAATAT
M5 GTAGTTGTGC TCGGTAATGC ACAATGAAAT AGGTGGTAGT GTCCATCTAA
GGCTAAATAT
M6 GTAGTTGTGC TCGGTAATGC ACAATGAAAT AGGTGGTAGT GTCCATCTAA
GGCTAAATAT
M7 GTAGTTGTGC TCGGTAATGC ACAATGAAAT AGGTGGTAGT GTCCATCTAA
GGCTAAATAT
M8 GTAGTTGTGC TCGGTAATGC ACAATGAAAT AGGTGGTAGT GTCCATCTAA
GGCTAAATAT
M9 GTAGTTGTGC TCGGTAATGC ACAATGAAAT AGGTGGTAGT GTCCATCTAA
GGCTAAATAT
M10 GTAGTTGTGC TCGGTAATGC ACAATGAAAT AGGTGGTAGT GTCCATCTAA
GGCTAAATAT
M11 GTAGTTGTGC TCGGTAATGC ACAATGAAAT AGGTGGTAGT GTCCATCTAA
GGCTAAATAT
M12 GTAGTTGTGC TCGGTAATGC ACAATGAAAT AGGTGGTAGT GTCCATCTAA
GGCTAAATAT
M13 GTAGTTGTGC TCGGTAATGC ACAATGAAAT AGGTGGTAGT GTCCATCTAA
GGCTAAATAT
M14 ?????????? ?????????? ?????????? ?????????? ??????????
??????????
M15 GTAGTTGTGC TCGGTAATGC ACAATGAAAT AGGTGGTAGT GTCCATCTAA
GGCTAAATAT
M16 GTAGTTGTGC TCGGTAATGC ACAATGAAAT AGGTGGTAGT GTCCATCTAA
GGCTAAATAT
M17 GTAGTTGTGC TCGGTAATGC ACAATGAAAT AGGTGGTAGT GTCCATCTAA
GGCTAAATAT
M18 GTAGTTGTGC TCGGTAATGC ACAATGAAAT AGGTGGTAGT GTCCATCTAA
GGCTAAATAT
M19 GTAGTTGTGC TCGGTAATGC ACAATGAAAT AGGTGGTAGT GTCCATCTAA
GGCTAAATAT
M20 GTAGTTGTGC TCGGTAATGC ACAATGAAAT AGGTGGTAGT GTCCATCTAA -
GCTAAATAT
M21 GTAGTTGTGC TCGGTAATGC ACAATGAAAT AGGTGGTAGT GTCCATCTAA
GGCTAAATAT
M22 GTAGTTGTGC TCGGTAATGC ACAATGAAAT AGGTGGTAGT GTCCATCTAA
GGCTAAATAT
M23 GTAGTTGTGC TCGGTAATGC ACAATGAAAT AGGTGGTAGT GTCCATCTAA
GGCTAAATAT
B1 ?????????? ?????????? ?????????? ?????????? ??????????
??????????
B2 GTAGTTGTGC TCGGTAATGC ACAATG-AAT AGGTGGTAGT GTCCATCTAA
GGCTAAATAT
B3 GTAGTTGTGC TCGGTAATGC ACAATG-AAT AGGTGGTAGT GTCCATCTAA
GGCTAAATAT
B4 GTAGTTGTGC TCGGTAATGC ACAATG-AAT AGGTGGTAGT GTCCATCTAA
GGCTAAATAT
B5 GTAGTTGTGC TCGGTAATGC ACAATG-AAT AGGTGGTAGT GTCCATCTAA
GGCTAAATAT
B6 GTAGTTGTGC TCGGTAATGC ACAATG-AAT AGGTGGTAGT GTCCATCTAA
GGCTAAATAT

B7 GTAGTTGTGC TCGGTAATGC ACAATG-AAT AGGTGGTAGT GTCCATCTAA
GGCTAAATAT
B8 GTAGTTGTGC TCGGTAATGC ACAATG-AAT AGGTGGTAGT GTCCATCTAA
GGCTAAATAT
B9 GTAGTTGTGC TCGGTAATGC ACAATG-AAT AGGTGGTAGT GTCCATCTAA
GGCTAAATAT
B10 GTAGTTGTGC TCGGTAATGC ACAATG-AAT AGGTGGTAGT GTCCATCTAA
GGCTAAATAT
B11 GTAGTTGTGC TCGGTAATGC ACAATG-AAT AGGTGGTAGT GTCCATCTAA
GGCTAAATAT
B12 GTAGTTGTGC TCGGTAATGC ACAATG-AAT AGGTGGTAGT GTCCATCTAA
GGCTAAATAT
B13 GTAGTTGTGC TCGGTAATGC ACAATG-AAT AGGTGGTAGT GTCCATCTAA
GGCTAAATAT
B14 GTAGTTGTGC TCGGTAATGC ACAATG-AAT AGGTGGTAGT GTCCATCTAA
GGCTAAATAT
B15 GTAGTTGTGC TCGGTAATGC ACAATG-AAT AGGTGGTAGT GTCCATCTAA
GGCTAAATAT
B16 GTAGTTGTGC TCGGTAATGC ACAATG-AAT AGGTGGTAGT GTCCATCTAA
GGCTAAATAT
KF537632_Ddi GTAGTTGTGC TCGGTAATGC ACAATGAATT TGGTGGTAGT GTCCATCAAA
GGCTAAATAT
AJ438961_Dde ?????????? ?????????? ?????????? ?????????? ??????????
??????????
AJ438960_Dca ?????????? ?????????? ?????????? ?????????? ??????????
??????????
AJ438959_Dca ?????????? ?????????? ?????????? ?????????? ??????????
??????????
AJ438958_Dga ?????????? ?????????? ?????????? ?????????? ??????????
??????????

....|....| ....|....| ....|....| ....|....| ....|....
1745 1755 1765 1775 1785
D1 ?????????? ?????????? ?????????? ?????????? ?????????
D2 GACATAGAAA CCGATAGTGA AGAAGTAAAG TGATCGAAAA TGGAATAGA
D3 GACATAGAAA CCGATAGTGA AGAAGTAAAG TGATCGAAAA TGGAATAGA
R1 GACATAGAAA CCGATAGTGA ATAAGTAAAG TGATCGAAAA TGGAATAGA
R2 GACATAGAAA CCGATAGTGG ATAAGTAAAG TGATCGAAAA TGGAATAGA
R3 GACATAGAAA CCGATAGTGG ATAAGTAAAG TGATCGAAAA TGGAATAGA
R4 GACATAGAAA CCGATAGTGG ATAAGTAAAG TGATCGAAAA TGGAATAGA
R5 GACATAGAGA CCGATAGTGA ATAAGTAAAG TGATCGAAAA TGGAATAGA
R6 GACATAGAAA CCGATAGTGG AAAAGTAAAG TGATCGAAAA TGGAATAGA
R7 GACATAGAAA CCGATAGTGG AAAAGTAAAG TGATCGAAAA TGGAATAGA
N1 GACATAGAAA CCGATAGTGA ATAAGTAAAG TGATCGAAAA TGGAATAGA
N2 GACATAGAAA CCGATAGTGA ATAAGTAAAG TGATCGAAAA TGGAATAGA
N3 GACATAGAAA CCGATAGTGA ATAAGTAAAG TGATCGAAAA TGGAATAGA
M1 ?????????? ?????????? ?????????? ?????????? ?????????
M2 GACATAGAGA CCGATAGTGA ATAAGTAAAG TGATCGAAAA TGGAATAGA
M3 GACATAGAGA CCGATAGTGA ATAAGTAAAG TGATCGAAAA TGGAATAGA
M4 GACATAGAGA CCGATAGTGA ATAAGTAAAG TGATCGAAAA TGGAATAGA
M5 GACATAGAGA CCGATAGTGA ATAAGTAAAG TGATCGAAAA TGGAATAGA
M6 GACATAGAGA CCGATAGTGA ATAAGTAAAG TGATCGAAAA TGGAATAGA
M7 GACATAGAGA CCGATAGTGA ATAAGTAAAG TGATCGAAAA TGGAATAGA
M8 GACATAGAGA CCGATAGTGA ATAAGTAAAG TGATCGAAAA TGGAATAGA
M9 GACATAGAGA CCGATAGTGA ATAAGTAAAG TGATCGAAAA TGGAATAGA
M10 GACATAGAGA CCGATAGTGA ATAAGTAAAG TGATCGAAAA TGGAATAGA
M11 GACATAGAGA CCGATAGTGA ATAAGTAAAG TGATCGAAAA TGGAATAGA
M12 GACATAGAGA CCGATAGTGA ATAAGTAAAG TGATCGAAAA TGGAATAGA

M13	GACATAGAGA	CCGATAGTGA	ATAAGTAAAG	TGATCGAAAA	TGGAATAGA
M14	??????????	??????????	??????????	??????????	?????????
M15	GACATAGAGA	CCGATAGTAA	ATAAGTAAAG	TGATCGAAAA	TGGAATAGA
M16	GACATAGAGA	CCGATAGTAA	ATAAGTAAAG	TGATCGAAAA	TGGAATAGA
M17	GACATAGAGA	CCGATAGTAA	ATAAGTAAAG	TGATCGAAAA	TGGAATAGA
M18	GACATAGAGA	CCGATAGTGA	ATAAGTAAAG	TGATCGAAAA	TGGAATAGA
M19	GACATAGAGA	CCGATAGTGA	ATAAGTAAAG	TGATCGAAAA	TGGAATAGA
M20	GACATAGAGA	CCGATAGTGG	ATAAGTAAAG	TGATCGAAAA	TGGAATAGA
M21	GACATAGAGA	CCGATAGTGG	ATAAGTAAAG	TGATCGAAAA	TGGAATAGA
M22	GACATAGAGA	CCGATAGTGG	ATAAGTAAAG	TGATCGAAAA	TGGAATAGA
M23	GACATAGAGA	CCGATAGTGG	ATAAGTAAAG	TGATCGAAAA	TGGAATAGA
B1	??????????	??????????	??????????	??????????	?????????
B2	GACATAGAGA	CCGATAGTGA	ATAAGTAGAG	TGATCGAATA	TGGAATAGA
B3	GACATAGAGA	CCGATAGTGA	ATAAGTAGAG	TGATCGAATA	TGGAATAGA
B4	GACATAGAGA	CCGATAGTGA	ATAAGTAGAG	TGATCGAATA	TGGAATAGA
B5	GACATAGAGA	CCGATAGTGA	ATAAGTAGAG	TGATCGAATA	TGGAATAGA
B6	GACATAGAGA	CCGATAGTGA	ATAAGTAGAG	TGATCGAATA	TGGAATAGA
B7	GACATAGAGA	CCGATAGTGA	ATAAGTAGAG	TGATCGAATA	TGGAATAGA
B8	GTCATAGAGA	CCGATAGTGA	ATAAGTAGAG	TGATCGAATA	TGGAATAGA
B9	GACATAGAGA	CCGATAGTGA	ATAAGTAGAG	TGATCGAATA	TGGAATAGA
B10	GACATAGAGA	CCGATAGTGA	ATAAGTAGAG	TGATCGAATA	TGGAATAGA
B11	GACATAGAGA	CCGATAGTGA	ATAAGTAGAG	TGATCGAATA	TGGAATAGA
B12	GACATAGAGA	CCGATAGTGA	ATAAGTAGAG	TGATCGAATA	TGGAATAGA
B13	GACATAGAGA	CCGATAGTGA	ATAAGTAGAG	TGATCGAATA	TGGAATAGA
B14	GACATAGAGA	CCGATAGTGA	ATAAGTAGAG	TGATCGAATA	TGGAATAGA
B15	GACATAGAGA	CCGATAGTGA	ATAAGTAGAG	TGATCGAATA	TGGAATAGA
B16	GACATAGAGA	CCGATAGTGA	ATAAGTAGAG	TGATCGAATA	TGGAATAGA
KF537632_Ddip	GACATAGAGA	CCGATAGAGA	AAAAGTAGAG	TGATCGAAAA	TGAAG-AGA
AJ438961_Ddes	??????????	??????????	??????????	??????????	?????????
AJ438960_Dcav	??????????	??????????	??????????	??????????	?????????
AJ438959_Dcav	??????????	??????????	??????????	??????????	?????????
AJ438958_Dgam	??????????	??????????	??????????	??????????	?????????
